# Antioxidant Potential of Santowhite as Synthetic and Ascorbic Acid as Natural Polymer Additives

**DOI:** 10.3390/polym14173518

**Published:** 2022-08-27

**Authors:** Dalal K. Thbayh, Edina Reizer, Mousumi U. Kahaly, Béla Viskolcz, Béla Fiser

**Affiliations:** 1 Institute of Chemistry, University of Miskolc, 3515 Miskolc, Hungary; 2 Polymer Research Center, University of Basrah, 61004 Basrah, Iraq; 3 Higher Education and Industrial Cooperation Centre, University of Miskolc, 3515 Miskolc, Hungary; 4 ELI-ALPS, ELI-HU Non-Profit Ltd., Wolfgang Sandner utca 3, 6728 Szeged, Hungary; 5 Institute of Physics, University of Szeged, Dóm tér 9, 6720 Szeged, Hungary; 6 Ferenc Rakoczi II Transcarpathian Hungarian College of Higher Education, 90200 Beregszász, Ukraine

**Keywords:** DFT, BDE, natural additive, IP, HAT

## Abstract

A wide variety of additives are used to improve specific characteristics of the final polymeric product. Antioxidant additives (AAs) can prevent oxidative stress and thus the damage of polymeric materials. In this work, the antioxidant potential and thus the applicability of Santowhite (SW) as synthetic and ascorbic acid (Asc) as natural AAs were explored by using computational tools. Two density functional theory (DFT) methods, M05-2X and M06-2X, have been applied in combination with the 6-311++G(2d,2p) basis set in gas phase. Three antioxidant mechanisms have been considered: hydrogen atom transfer (HAT), single electron transfer-proton transfer (SET-PT), and sequential proton loss electron transfer (SPLET). Bond dissociation enthalpy (BDE), ionization potential (IP), proton dissociation enthalpy (PDE), proton affinity (PA), and electron transfer enthalpy (ETE) have been computed for each potential hydrogen donor site. The results indicate that the antioxidant potential of Asc is higher than SW. Furthermore, some of the C-H bonds, depending on their position in the structures, are potent radical scavengers, but O-H groups are more prone to donate H-atoms to free radicals. Nonetheless, both additives can be potentially applied to safeguard common polymers and prohibit oxidative stress-induced material deterioration.

## 1. Introduction

At present, polymers and plastic materials pervade in all aspects of our lives, but these are not pure polymeric materials because they are not suitable for most applications, and thus, it would lead to their commercial failure. The characteristics of polymeric materials can be improved through incorporating additives into their formulations that will enhance the processability and properties of the final product [1,2]. In the absence of additives, most polymers probably would not be applicable as widely as they are. One example is polypropylene, which would decay in just a few weeks due to its weak thermal oxidative stability [3]. As a result, additives play a critical role in the processing and improvement of polymers used in a wide range of applications, such as automotive, construction, packaging, electronics, and telecommunications fields [4].

**Figure 1:**
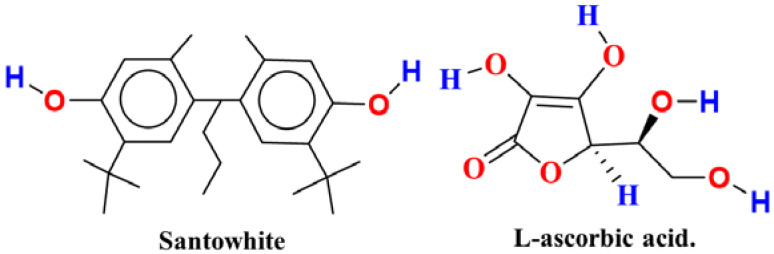
2D chemical structure of Santowhite and L-ascorbic acid.

There are many types of additives that are used to improve specific characteristics of the final polymeric product. Polymer additives have been used as plasticizers (e.g., dioctylphthalate (DOP) and tricresyl phosphate (TCP)) [5], flame retardants (e.g., polybrominated diphenylethers (PBDES) and tris (chloropropyl) phosphate (TCPP)) [1], colorants (e.g., anthraquinone and carbon blacks) [6], stabilizers (e.g., barium-zinc and calcium-zinc) [7], antimicrobial agents (e.g., 5-chloro-2-(2,4-dichlorophenoxy) phenol and 4,5-dichloro-2-noctylisothiazolinone) [6], antioxidants (e.g., butylated hydroxytoluene (BHT), *tert*-butylhydroquinone (TBHQ), ascorbic acid (Asc) [8], curcumin [9,10], vitamin E, and Santowhite) [11]. Antioxidants can protect other molecules from oxidative stress-related damage [12,13]. Oxidative stress is a process that can produce free radicals [14], which are characterized by having an unpaired electron in their outer shell, making them unstable and highly reactive [15,16]. Free radicals in the body have the capability to damage DNA, cells, and tissues through the oxidative stress, because they can react swiftly with non-radical species to produce new radicals and propagate free-radical chain reactions [17]. Similar processes can occur in the case of polymeric materials. These harmful reactions can be prevented by antioxidant species, which can donate an electron to free radicals and consequently stabilize and detoxify them. There are various factors that must be considered to choose the best possible antioxidant compounds for a given system. The most important factors are the following: (1) radicals—the antioxidant should be capable of scavenging the appropriate radicals, (2) timing—the radical scavenging should occur at a specific location during the most suitable time within which the antioxidant additives are able to give an electron to the free radicals and make them more stable, (3) activity—the antioxidants must remain active and not degrade as long as possible [18]. Antioxidant species are divided into two categories: synthetic and natural compounds [19]. Due to the significant environmental concerns, manufacturers have been pushed to investigate natural alternatives along with regularly used synthetic materials. There are several synthetic antioxidants, such as butylated hydroxytoluene (BHT), *tert*-butylhydroquinone (TBHQ), octyl gallate (OG), butylated hydroxyanisole (BHA), propyl gallate (PG), and Santowhite (SW), which are routinely applied as additives. The latter, SW, is a hindered phenolic antioxidant with two phenolic groups per molecule (Figure 1).

Santowhite has the ability to inhibit the oxidation of polymers through donating a labile hydrogen atom to the polymeric hydroperoxide radical ROO^•^ or the polymeric radical R^•^, resulting in the formation of a stable phenoxy radical [11]. SW is considered a highly effective antioxidant due to its stable phenoxy radical forms and its ability to end multiple chains [20]. Whilst the raw polymer is easily oxidized, efficient antioxidants such as SW have proved that they are well-suited to improving durability when employed in the polymer formulation. The molecular structure for the antioxidant and the dose of exposure have a considerable influence on the efficacy of stabilization [21]. Environmental issues significantly becoming more prevalent due to the increasing number of synthetic materials, and the replacement of synthetic additives with environmentally friendly (e.g., natural) substances is desired. Thus, finding ecologically safe and effective alternatives to commonly used additives is a top priority for the major players of the chemical industry, but on the other hand, it is difficult and may lead to a significant increase in production costs [15]. Natural antioxidants are derived from a variety of natural products (e.g., fruits, leaves, and flowers) [16,22,23]. Vitamin C or L-ascorbic acid (Asc) is one of the most well-known natural antioxidants of all time. Asc has gained widespread recognition as a powerful antioxidant and free-radical scavenger since its discovery by Albert Szent-Györgyi and Walter Norman Haworth (Figure 1) [24,25]. It can be oxidized via losing two protons and two electrons, but it usually only loses one electron at a time [26]. Asc is present mainly in fresh fruits including citrus fruits, kiwifruit, strawberries, papaya, blackcurrant, and vegetables such as tomato, carrot, coriander, broccoli, cauliflower, cabbage, and others [8,27]. The promising experimental results of Asc and SW used as antioxidant additives in polymeric formulations [11,28,29,30] encouraged us to study their radical scavenging mechanisms and compare their antioxidant potential.

## 2. Materials and Methods

All calculations have been carried out by using the Gaussian 09 software package [31]. The studied species, SW and Asc, and the corresponding radicals, radical cations, and anions were optimized by employing two global hybrid functionals, M05-2X [32] and M06-2X [33], in combination with the 6-311++G(2d,2p) basis set in the gas phase (Figures S1 and S2). Both of the applied methods have been effectively utilized before to study thermochemistry, kinetics, and non-covalent interactions, particularly of free radical processes [34,35,36,37]. However, the M06-2X/6-311++G(2d,2p) level of theory has been chosen to use during the discussion as it was successfully utilized in the study of similar systems and processes before [38].

### Computational Details

**Figure 2:**
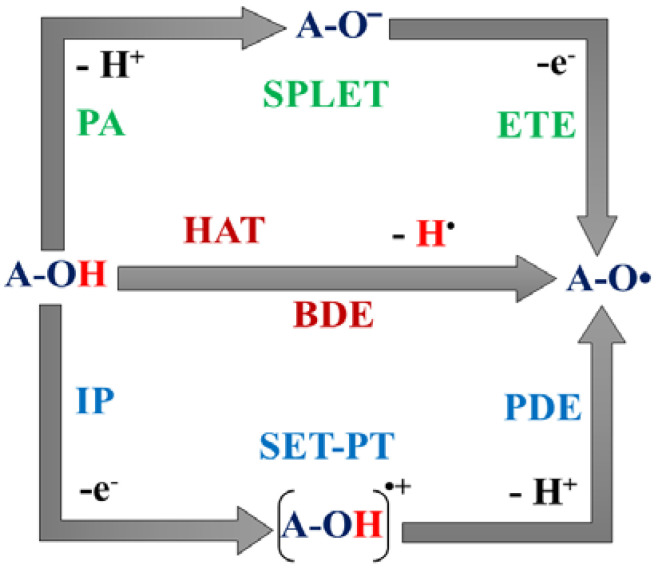
Schematic representation of the studied radical scavenging mechanisms through a hydroxyl group: hydrogen atom transfer (HAT), single electron transfer-proton transfer (SET-PT), and sequential proton loss electron transfer (SPLET). A—antioxidant, OH—hydroxyl group. BDE: bond dissociation enthalpy, IP: ionization potential, PDE: proton dissociation enthalpy, PA: proton affinity, and ETE: electron transfer enthalpy.

Three major free-radical scavenging (RS) mechanisms were considered to examine H^•^ transfers from all unique X-H (X = C, or O) positions of the studied molecules: hydrogen atom transfer (HAT), single electron transfer-proton transfer (SET-PT), and sequential proton loss electron transfer (SPLET) [8,36,39,40,41,42,43,44] (Figure 2).

The antioxidant potential of the studied species was determined and compared. In the case of the HAT mechanism (Figure 2), bond dissociation enthalpy (BDE) is the most essential parameter to evaluate the corresponding antioxidant potential as a hydrogen atom (H^•^) is transferred from the antioxidant (A) to the radical. BDE is calculated as follows

BDE = *H*(A^•^) + *H*(H^•^) − *H*(A) (1)

The SET-PT mechanism includes two steps (Figure 2): electron transfer followed by a proton transfer from the antioxidant (A) to the radical. Thus, two parameters determine the antioxidant potential, ionization potential (IP) and proton dissociation enthalpy (PDE) which were calculated according to the following equations:

IP = *H*(A^•+^) + *H*(e^−^) − *H*(A) (2)

PDE = *H*(A^•^) + *H*(H^+^) − *H*(A^•+^) (3)

The SPLET mechanism also includes two steps (Figure 2): a proton transfer followed by an electron transfer from the antioxidant (A) to the free radical. To compare the antioxidant potential of the unique X-H positions of the species, proton affinity (PA) and electron transfer enthalpy (ETE) values were computed as follows:

PA = *H*(A^−^) + *H*(H^+^) − *H*(A) (4)

ETE = *H*(A^•^) + *H*(e^−^) − *H*(A^−^) (5)

*H*(e^−^) and *H*(H^+^) in gas phase are 3.1351 kJ/mol and 6.1398 kJ/mol, respectively [38,45,46,47].

## 3. Results and Discussion

### 3.1. Structural Properties

**Figure 3:**
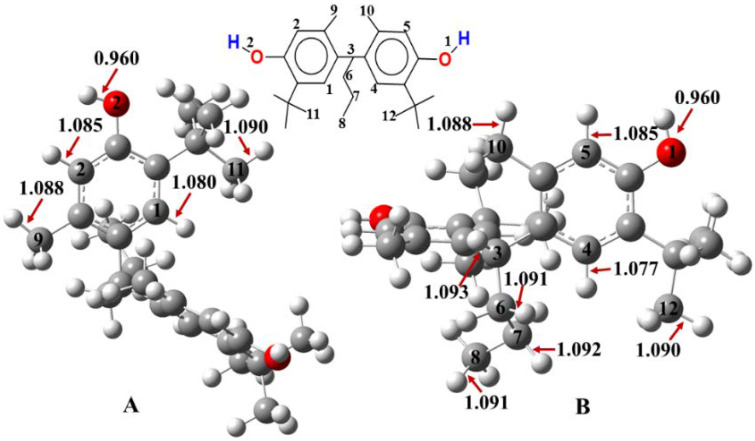
2D and 3D structures of the studied synthetic antioxidant additive, Santowhite. (**A**,**B**) are the same 3D structure from different points of view. Geometry optimizations have been carried out at the M06-2X/6-311++G(2d,2p) level of theory in gas phase, and the corresponding bond lengths are also shown in Å.

The structure of Santowhite has been optimized by employing two global hybrid functionals: M05-2X and M06-2X (Figure 3 and Figure S1). The X-H bonds of SW optimized at the M05-2X//6-311++G(2d,2p) level of theory are shorter compared to the M06-2X results, but the trends are the same. O1-H and O2-H are equal to 0.960 Å at the M06-2X//6-311++G(2d,2p) level of theory. As for C-H bonds, the longest is C3-H (1.093 Å) whilst the shortest is a benzylic carbon–hydrogen bond, C4-H with 1.077 Å (Figure 3).

**Figure 4:**
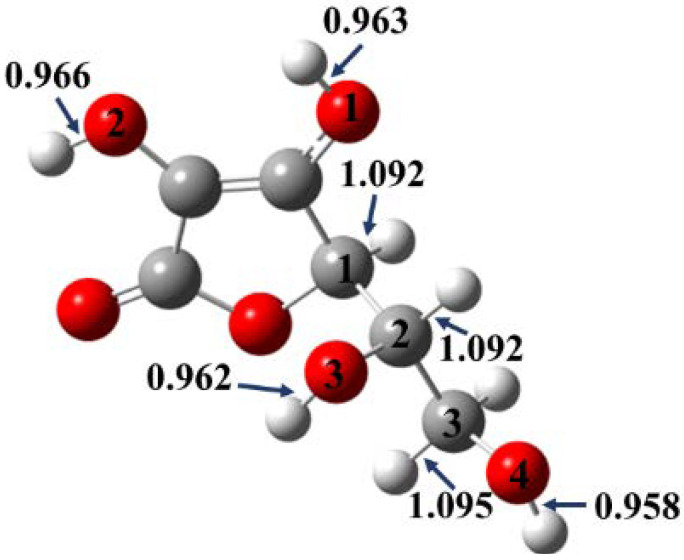
Optimized geometry of the studied natural antioxidant additive, L-ascorbic acid (Asc). Geometry optimizations have been carried out at the M06-2X/6-311++G(2d,2p) level of theory in gas phase, and the corresponding bond lengths are also shown in Å.

In the case of Asc, the structure includes four hydroxyl groups and three unique C-H positions (Figure 4 and Figure S2).

The shortest O-H bond is O4-H with a length equal to 0.958 Å, while the longest value belongs to O2-H with a length equal to 0.966 Å at the M06-2X//6-311++G(2d,2p) level of theory (Figure 4). The C-H bonds cover a range from 1.092 to 1.095 Å, where the longest is C3-H, whereas the shortest value belongs to C1-H and C2-H with a length equal to 1.092 Å (Figure 4). All in all, the trend is the same, but the bonds are longer compared to the results obtained at the M05-2X//6-311++G(2d,2p) level of theory (Figure S2).

### 3.2. Antioxidant Mechanisms

#### 3.2.1. Hydrogen Atom Transfer (HAT)

**Figure 5:**
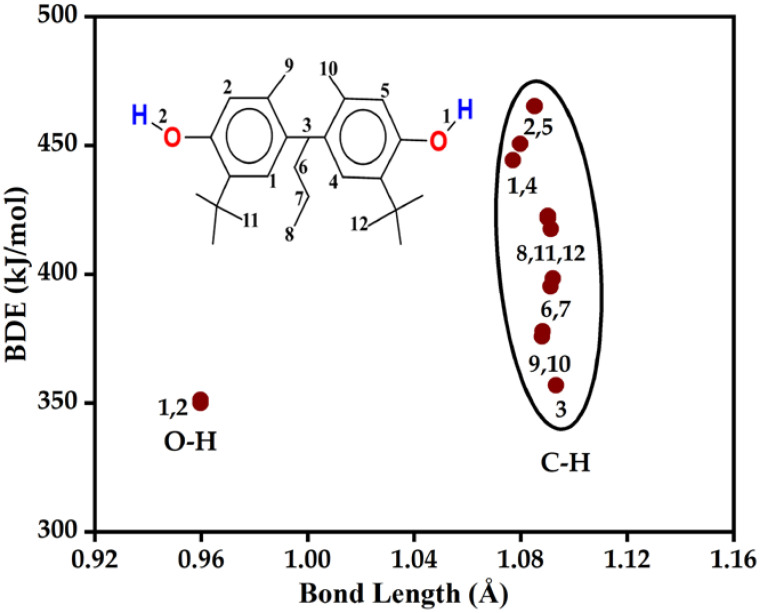
Bond dissociation enthalpy (BDE) vs. bond length plot for Santowhite (SW). Calculations have been carried out at the M06-2X/6-311++G(2d,2p) level of theory in gas phase. The numbers represent the X-H (X = C, H) bonds in the structure.

In the case of the HAT mechanism, the most active bonds are those with the lowest BDE value [48]. The higher the antioxidant potential, the lower the BDE value, since it is easier to donate the hydrogen atom to free radicals [38,49,50]. BDE values of O-H and C-H bonds for SW have been computed and compared to determine the most potent antioxidant sites and also verify the applicability of the studied additives in polymer formulations (Table 1 and Table S1 and Figure 5).

SW has two O-H bonds, O1-H and O2-H, and their BDE values cover a narrow range of 350.0 to 351.3 kJ/mol (Table 1, Figure 5). The BDE of O2-H was found to be slightly lower than O1-H, but both are smaller compared to the bond dissociation enthalpy of the C-H bonds within the structure determined by using both functionals (Table 1 and Table S1).

As for the BDE values of the C-H bonds of SW, these cover a range between 357.0 to 465.3 kJ/mol (Figure 5). The weakest bond is C3-H with a BDE = 357.0 kJ/mol and it is located between the two phenolic groups, while the strongest one is a benzylic hydrogen (C2-H, BDE = 465.3 kJ/mol). Similar results were obtained at the M05-2X/6-311++G(2d,2p) level of theory (Table S1). The C-H bonds are mostly stronger as the corresponding BDE values are higher than their O-H counterparts, but we noticed that some C-H bonds are close to the O-H bonds in terms of their antioxidant potential, including C3-H, C6-H, C7-H, C9-H, and C10-H, where C3-H is between the two phenolic groups in the molecule, whilst C6-H and C7-H are located on the propyl group in the middle, and C9-H and C10-H are located on methyl groups (Figure 5). The BDE values of these C-H bonds can be arranged in the following order: C3-H < C9-H < C10-H < C6-H < C7-H with BDE values equal to 357.0 < 375.9 < 377.9 < 395.4 < 398.4 kJ/mol. All in all, based on the results, the O-H bonds are more potent antioxidant sites in SW, and to donate an H atom from these to a free radical is easier than from their C-H counterparts. However, there are some potentially good free radical scavengers within the C-H sites as well.

**Figure 6:**
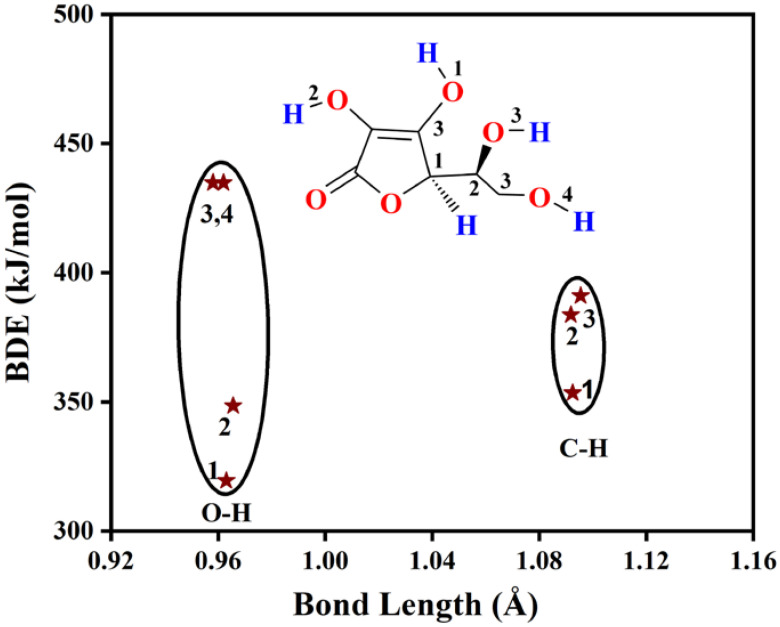
Bond dissociation enthalpy (BDE) vs. bond length plot for L-ascorbic acid (Asc) as natural antioxidant additive. The calculations have been carried out at the M06-2X/6-311++G(2d,2p) level of theory in gas phase.

In the case of Asc, there are four O-H and three C-H bonds for which the bond dissociation enthalpies have been computed (Table 2 and Table S2 and Figure 6). 

The corresponding computed X-H bond dissociation enthalpy values of Asc are similar in the case of both M05-2X and M06-2X functionals (Table 2 and Table S2). According to the calculations, O1-H has the highest antioxidant potential within Asc and its BDE is lower by 29 kJ/mol and 115.3 kJ/mol than that of O2-H and O3-H≈O4-H, respectively (Table 2, Figure 6). These results are in good agreement with previous studies [8,48,51,52]. The BDEs of the C-H bonds are higher than O1-H and O2-H but lower than O3-H and O4-H and cover a range from 353.4 to 391.0 kJ/mol, where the weakest bond is C1-H, while the strongest one is C3-H (Table 2). Thus, it can be concluded that the O1-H followed by O2-H has the highest contribution to the antioxidant activity of Asc in the case of the HAT mechanism. 

By comparing Asc with SW, the natural antioxidant has a higher antioxidant potential than its synthetic counterpart. To test the applicability of the studied antioxidant additives, BDE values of commonly used polymers including polyethylene (PE), polysulfone (PS), polycarbonate (PC), and polypropylene (PP) have been collected from the literature and compared to the calculated data (Table 3). It was found that the BDE values of commonly used polymers cover a range between 393.7 and 406.2 kJ/mol [53] (Table 3).

It can be seen that the lowest BDE value of both studied structures is well below the corresponding bond dissociation enthalpies of commonly used polymers. This indicates that the studied molecules are applicable to prevent oxidative stress-induced deterioration of polymeric products.

#### 3.2.2. Single Electron Transfer-Proton Transfer (SET-PT)

In the SET-PT mechanism, the first step is an electron transfer from the antioxidant (**A**) to the radical. The **A** then becomes a radical cation (**A^•+^**), which in turn deprotonates through the second step to form a radical (**A**^•^) [8,38,43]. To measure the antioxidant activity of the corresponding X-H site, first the ionization potential (IP) is calculated and then the proton dissociation enthalpy (PDE) has to be determined (Figure 2) [8,38,54]. The IP for SW was found to be 715.8 kJ/mol at the M06-2X/6-311++G(2d,2p) level of theory (Table 4). PDEs indicate that O-H bonds are more prone to deprotonation in the case of the second step of the SET-PT mechanism of SW than C-H bonds, but C3-H is a close competitor of O-H groups. Similar results were obtained at the M05-2X/6-311++G(2d,2p) level of theory (Table S3).

In the case of Asc, IP was found to be 816.7 kJ/mol (Table 5). The PDEs for all C-H and O-H sites cover a range between 813.9 to 929.2 kJ/mol, where the weakest one is O1-H and the strongest one is O3-H. The PDEs of these C-H and O-H bonds can be arranged in the following order: O1-H < O2-H < C1-H < C2-H < C3-H < O4-H < O3-H with PDE values equal to 813.9 < 833.0 < 838.3 < 878.1 < 892.0 < 921.8 < 929.2 kJ/mol, respectively (Table 5). The results corresponding to O-H bonds are in good agreement with previous studies [8,52] and the M05-2X results (Table S4).

All in all, the IP + PDE values of both SW and Asc indicate that the antioxidant activity of ascorbic acid is higher than Santowhite, which is in agreement with the findings of the HAT mechanism.

#### 3.2.3. Sequential Proton Loss Electron Transfer (SPLET)

In the case of the SPLET mechanism, the first step includes the dissociation of a proton from the antioxidant (**A**), which led to the formation of an anion (**A^−^**). Thereafter, the (**A^−^**) anion transfers an electron to the scavenged radical and became a radical (**A^•^**) itself. To determine the antioxidant potential in the case of the SPLET mechanism, PA and ETE values have been computed for SW (Table 6 and Table S5).

The proton affinities of SW indicate that proton transfer is more probable from the hydroxyl groups than from the C-H sites. The best proton transfer ability corresponds to the O2-H site with a PA equal to 1427.2 kJ/mol and it is lower by about 7.2 kJ/mol than the corresponding value of O1-H (1434.4 kJ/mol). The antioxidant potential of the different X-H bonds of SW in the case of the SPLET mechanism was determined by calculating the sum of PA and ETE, and the best O-H and C-H sites were ranked as follows: O2-H>O1-H>C3-H (Table 6). A similar trend was obtained at both levels of theory (Table 6 and Table S5).

**Figure 7:**
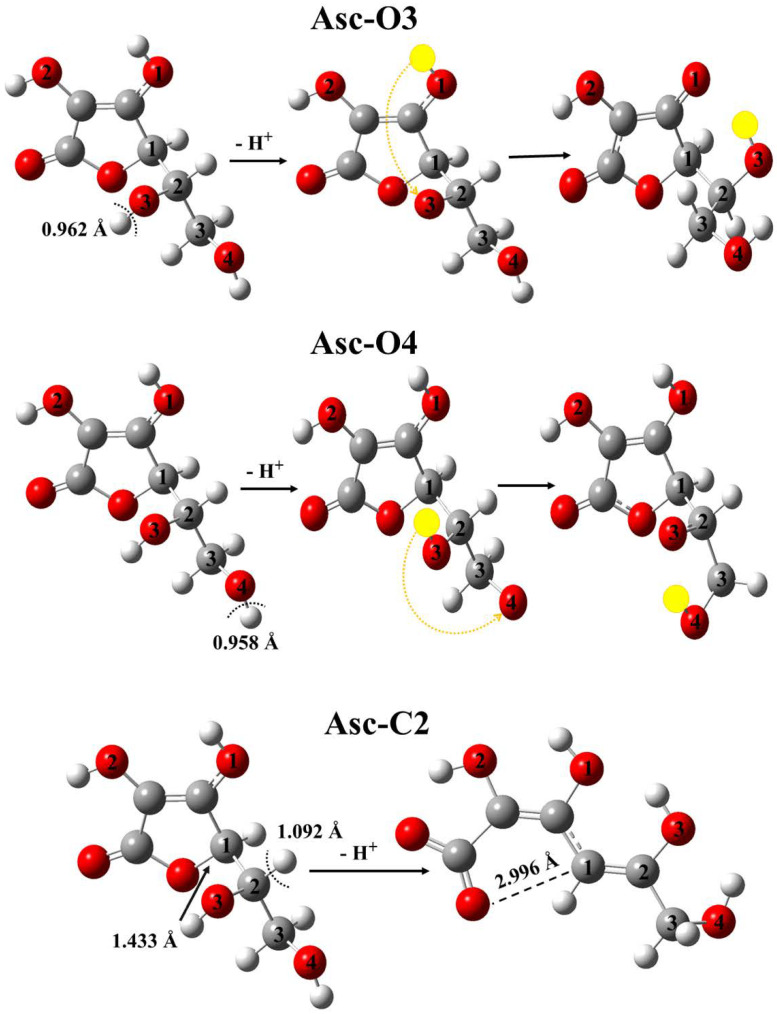
Optimized structures of ascorbic acid (Asc) and the corresponding anionic species after proton loss in the case of the O3-H, O4-H, and C2-H sites. The species have been computed at the M06-2X/6-311++G(2d,2p) level of theory in the gas phase and the corresponding bond lengths and distances (in Å) are also shown. Intramolecular proton transfers are indicated with orange arrows and the transferred protons are highlighted as yellow spheres.

As for Asc, the values of PA and ETE have been computed only for four sites (O1-H, O2-H, C1-H, and C3-H) in the case of both M05-2X and M06-2X functional (Table 7 and Table S6), because in the case of the other three X-H sites (O3-H, O4-H, and C2-H), intramolecular rearrangement is occurred after the proton transfer and thus the SPLET was not comparable (Figure 7).

Similarly, in the case of Asc, proton transfer is more probable from the hydroxyl groups compared to the C-H sites. The best proton donor is O1-H with a proton affinity equal to 1336.1 kJ/mol and it is lower by about 52.5 kJ/mol than O2-H (1388.6 kJ/mol) (Table 7 and Table S6). Regarding the C-H sites, it can be noticed that the PA of C1-H (1455.6 kJ/mol) is lower by about 168.4 kJ/mol than that of C3-H (1624.0 kJ/mol). The antioxidant potential of the X-H sites within Asc for the SPLET mechanism was determined as in the case of SW. It was found that the PA+ETE values of the X-H sites are in the following order: O1-H<C1-H < O2-H < C3-H with 1631.9 < 1649.7 < 1655.8 < 1693.7 kJ/mol, respectively (Table 7). Thus, O1-H has the highest, while C3-H has the lowest antioxidant potential. The order is the same in the case of the M05-2X functional with slight deviations in the values (Table S6). All in all, the antioxidant activity of ascorbic acid is higher considering the SPLET mechanism as well compared to Santowhite.

In case of the abovementioned three X-H sites, O3-H, O4-H, and C2-H, intramolecular rearrangement is experienced after the proton transfer step (Figure 7).

The O3-H and O4-H sites are gaining back the proton from nearby positions such as O1-H and O3-H, respectively (Figure 7). In the case of C2-H, the proton loss initiates the opening of the ring and led to an acyclic compound (Figure 7, bottom). Although the rearrangements prevent us from comparing these sites with the others in the case of the SPLET mechanism, it does not mean that O3-H, O4-H, and C2-H do not contribute to the antioxidant activity of ascorbic acid.

All in all, the antioxidant potential of Asc and SW have been studied and compared. To the best of our knowledge, Santowhite has not been studied before, and thus the current results are important and novel contributions to the field. Furthermore, there are some C-H sites that are almost as important as the O-H sites in terms of antioxidant potential. This information could also be important in synthetic antioxidant additive design.

## 4. Conclusions

In the current study, the antioxidant potential of two additives, one synthetic and one natural, Santowhite and vitamin C, have been determined and compared. Computational chemical tools have been used to compare the ability of these additives to donate H atoms to free radicals. The most probable radical scavenging sites have been explored based on DFT calculations carried out by using the M05-2X and M06-2X functional in combination with the 6-311++G(2d,2p) basis set in the gas phase. Three major free-radical scavenging (RS) mechanisms—hydrogen atom transfer (HAT), single electron transfer-proton transfer (SET-PT), and sequential proton loss electron transfer (SPLET)—are considered to be able to describe how these antioxidants donate atomic hydrogen from their X-H (X = C, or O) sites and the corresponding bond dissociation enthalpy (BDE), ionization potential (IP), proton dissociation enthalpy (PDE), proton affinity (PA), and electron transfer enthalpy (ETE) values were computed. Antioxidant properties of Santowhite have been successfully described at the molecular level for the first time. In the case of ascorbic acid, after the proton loss in the SPLET mechanism, three X-H sites displayed interesting behaviour and intramolecular rearrangements are experienced. All in all, the natural antioxidant, Asc, has a higher antioxidant potential than its synthetic counterpart, SW. Furthermore, in both structures, C-H sites were identified that are almost as important as the O-H sites in terms of their antioxidant potential, depending on their position in the structures. By comparing bond dissociation enthalpy values of commonly used polymers from the literature with the lowest BDE values of the studied antioxidant additives, it was found that there is at least one X-H bond in the investigated species that has a lower bond dissociation enthalpy value than the polymeric materials. Therefore, it was revealed that these additives can be used to safeguard the polymers and prohibit oxidative stress-induced material deterioration.

## Figures and Tables

**Table 1:** Bond dissociation enthalpy (BDE) (in kJ/mol) for all unique C-H and O-H bonds in Santowhite (SW) calculated at the M06-2X/6-311++G(2d,2p) level of theory in gas phase.

SW	
X-H Positions	BDE (kJ/mol)
O1-H	351.3
O2-H	350.0
C1-H	450.7
C2-H	465.3
C3-H	357.0
C4-H	444.3
C5-H	465.2
C6-H	395.4
C7-H	398.4
C8-H	417.6
C9-H	375.9
C10-H	377.9
C11-H	421.8
C12-H	422.7

**Table 2:** Bond dissociation enthalpy (BDE) values (in kJ/mol) for all C-H and O-H bonds of ascorbic acid (Asc) at the M06-2X/6-311++G(2d,2p) level of theory in gas phase.

Asc	
X-H Positions	BDE (kJ/mol)
O1-H	319.5
O2-H	348.5
O3-H	434.7
O4-H	434.8
C1-H	353.4
C2-H	383.6
C3-H	391.0

**Table 3:** Bond dissociation enthalpy (BDE) values of commonly used polymers such as polyethylene (PE), polysulfone (PS), polycarbonate (PC), and polypropylene (PP) along with the lowest BDE values (in kJ/mol) of the studied antioxidants, Santowhite (SW) and L-ascorbic acid (Asc) computed at the M06-2X/6-311++G(2d,2p) level of theory in the gas phase.

Compound	BDE (kJ/mol)	Ref.
Synthetic additive		- *
SW	350.0	
Natural additive		
Asc	319.5	
Polymers		
PE	393.7	[53] **
PP	403.7	
PC	405.0	
PS	406.2	

* This work ** Literature value.

**Table 4:** Calculated ionization potential (IP) and lowest proton dissociation enthalpy (PDE) values in kJ/mol for Santowhite (SW) determined at the M06-2X/6-311++G(2d,2p) level of theory in gas phase.

Compound	IP	PDE	IP + PDE
SW	715.8		
O1-H		946.6	1662.4
O2-H		945.3	1661.1
C3-H		952.3	1668.1

**Table 5:** Calculated ionization potential (IP) and proton dissociation enthalpy (PDE) values in kJ/mol for ascorbic acid (Asc) determined at the M06-2X/6-311++G(2d,2p) levels of theory in gas phase.

Compound	IP	PDE	IP + PDE
Asc	816.7		
O1-H		813.9	1630.6
O2-H		833.0	1649.7
O3-H		929.2	1745.9
O4-H		921.8	1738.5
C1-H		838.3	1655.0
C2-H		878.1	1694.8
C3-H		892.0	1708.7

**Table 6:** Lowest proton affinities (PAs) and electron transfer enthalpies (ETE) in kJ/mol for Santowhite (SW) calculated at the M06-2X/6-311++G(2d,2p) level of theory in gas phase.

SW	PA	ETE	PA + ETE
O1-H	1434.4	228.0	1662.4
O2-H	1427.2	233.9	1661.1
C3-H	1554.1	114.0	1668.1

**Table 7:** Proton affinities (PAs) and electron transfer enthalpies (ETEs) in kJ/mol for ascorbic acid (Asc) calculated at the M06-2X/6-31 G(2d,2p) level of theory in the gas phase.

Asc	PA	ETE	PA + ETE
O1-H	1336.1	295.8	1631.9
O2-H	1388.6	267.2	1655.8
O3-H	-*	-*	-*
O4-H	-*	-*	-*
C1-H	1455.6	194.1	1649.7
C2-H	-*	-*	-*
C3-H	1624.0	69.7	1693.7

-* intramolecular rearrangement after proton transfer.

## Data Availability

Structures, figures, and additional tables are available in the supporting information.

## Supplementary Materials

The following supporting information can be downloaded at: https://www.mdpi.com/article/10.3390/polym14173518/s1; Optimized geometries of the studied species. Figure S1: 2D and 3D structures of the studied synthetic antioxidant additive santowhite. Figure S2: 3D structure of the studied natural antioxidant additive ascorbic acid (Asc). Table S1–S6: Antioxidant properties of the studied species calculated at the M05-2X/6-311++G(2d,2p) level of theory in gas phase. Cartesian coordinates of the optimized structures.
