# Supplementary material for: Antioxidant Potential of Santowhite as Synthetic and Ascorbic Acid as Natural Polymer Additives
Source: Polymers (Basel). 2022 Aug 27;14(17):3518. doi: 10.3390/polym14173518 (PMC9460313; doi:10.3390/polym14173518)
Supplement: Supplementary file 1 [file polymers-14-03518-s001.zip › polymers-1844048-supplementary.pdf]

Antioxidant potential of santowhite as synthetic and ascorbic acid as natural polymer additives

Dalal K. Thbayh[1,2,3], Edina Reizer[1], Mousumi U. Kahaly[4,5], Béla Viskolcz[1,3], Béla Fiser[1,3,6,*]

[1]Institute of Chemistry, University of Miskolc, 3515 Miskolc-Egyetemváros, Hungary
[2]Polymer Research Center, University of Basrah, 61004 Basrah, Iraq
[3]Higher Education and Industrial Cooperation Centre, University of Miskolc, 3515 Miskolc-Egyetemváros, Hungary
[4]ELI-ALPS, ELI-HU Non-Profit Ltd., Wolfgang Sandner utca 3, 6728 Szeged, Hungary
[5]Institute of Physics, University of Szeged, Dóm tér 9, 6720 Szeged, Hungary
[6]Ferenc Rákóczi II. Transcarpathian Hungarian College of Higher Education, 90200 Beregszász, Transcarpathia, Ukraine

*kemfiser@uni-miskolc.hu

Table of Contents

List of Abbreviations	2
Figure S1	2
Figure S2	3
1.Antioxidant Mechanisms	3
1.1 Hydrogen atom transfer (HAT)	3
Table S1	4
Table S2	5
1.2 Single electron transfer-proton transfer (SET-PT)	5
Table S3	5
Table S4	6
1.3 Sequential proton loss electron transfer (SPLET)	6
Table S5	7
Table S6	7
Structure of L-ascorbic acid (**Asc**) and the corresponding radical species in the HAT mechanism optimized at the **M06-2X/6-311++G(2d,2p)** level of theory in gas phase.	8
SET-PT (SET step) mechanism	9
SET-PT (PT step) mechanism	9
SPLET (SP step) mechanism	10
SPLET (ETE step) mechanism	11
Structures of santowhite (**SW**) and the corresponding radical species in the HAT mechanism optimized at the **M06-2X/6-311++G(2d,2p)** level of theory in gas phase.	12
SET-PT (SET step) mechanism	18
SET-PT (PT step) mechanism	19
SPLET (SP step) mechanism	25
SPLET (ETE step) mechanism	31
Structure of L-ascorbic acid (**Asc**) and the corresponding radical species in the HAT mechanism optimized at the **M05-2X/6-311++G(2d,2p)** level of theory in gas phase.	37

SET-PT (SET step) mechanism	38
SET-PT (PT step) mechanism	38
SPLET (SP step) mechanism	40
SPLET (ETE step) mechanism	41
Structures of santowhite (**SW**) and the corresponding radical species in the HAT mechanism optimized at the **M05-2X/6-311++G(2d,2p)** level of theory in gas phase.	42
SET-PT (SET step) mechanism	48
SET-PT (PT step) mechanism	49
SPLET (SP step) mechanism	55
SPLET (ETE step) mechanism	61

List of abbreviations

HAT: hydrogen atom transfer
SET-PT: single electron transfer-proton transfer
SPLET: sequential proton loss electron transfer
Asc: L-ascorbic acid
SW: santowhite

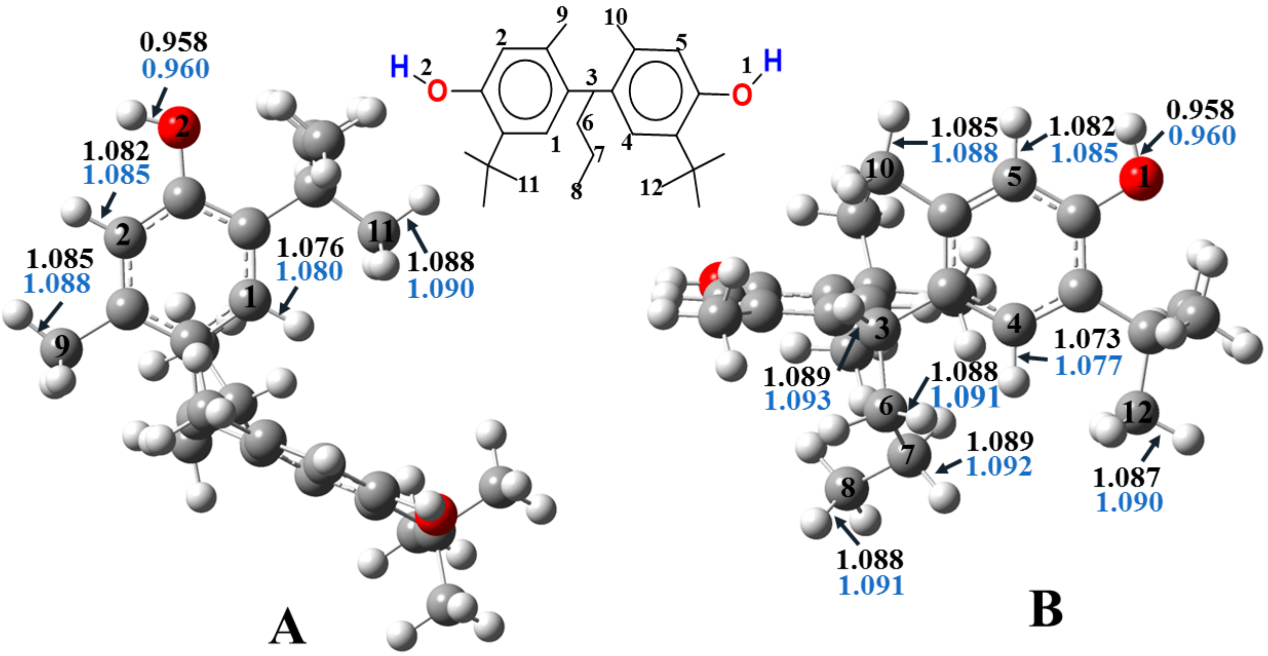

Figure S1. 2D and 3D structures of the studied synthetic antioxidant additive, santowhite. **A** and **B** are the same 3D structure from different point of views. Geometry optimizations have been carried out at the M05-2X/6-311++G(2d,2p) and M06-2X/6-311++G(2d,2p) levels of theory in gas phase, and the corresponding bond lengths (in Å) are also shown in **black** and **blue**, respectively.

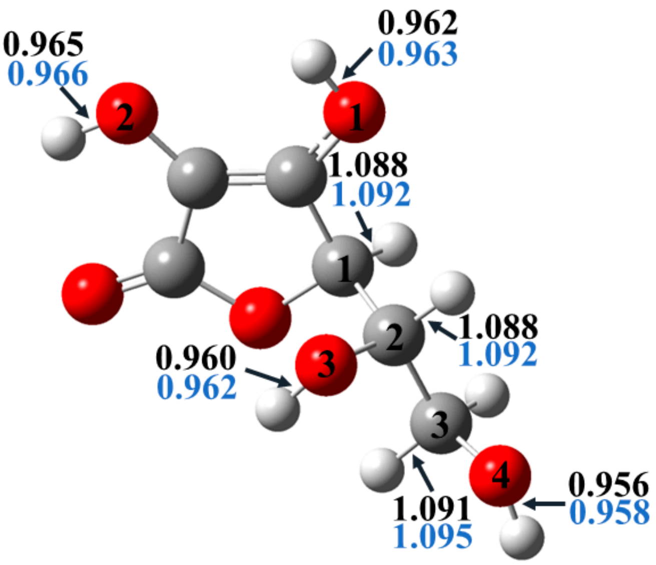

Figure S2. 3D structure of the studied natural antioxidant additive, ascorbic acid (Asc). Geometry optimizations have been carried out at the M05-2X/6-311++G(2d,2p) and M06-2X/6-311++G(2d,2p) levels of theory in gas phase, and the corresponding bond lengths (in Å) are also shown in **black** and **blue**, respectively.

1. Antioxidant Mechanisms

1.1 Hydrogen atom transfer (HAT)

SW has two O-H bonds, O1-H and O2-H, which have lower BDE values compare to C-H bonds. The BDE of O2-H was found to be slightly lower than O1-H at both theoretical levels but covered a narrow range of 351.2 to 351.5 kJ/mol (**Table S1**).
As for the BDE values of the C-H bonds of SW calculated at the M05-2X/6-311++G(2d,2p) level of theory, these cover a range between 356.2 to 474.8 kJ/mol (**Table S1**). The weakest bond is C3-H with a BDE=356.2 kJ/mol and it is located between the two phenolic groups while the strongest one is a benzylic hydrogen (C2-H, BDE=474.8 kJ/mol).
The C-H bonds are mostly stronger as the corresponding BDE values are higher than their O-H counterparts, but we noticed that some C-H bonds are close to the O-H bonds in terms of their antioxidant potential, including C3-H, C6-H, C7-H, C9-H, and C10-H, where C3-H link the two phenolic groups together in the molecule, whilst C6-H and C7-H are located on the propyl group in the middle, whereas C9-H and C10-H are located on methyl groups (**Figure S1**). The BDE values of these C-H bonds can be arranged in the following order: C3-H < C10-H < C9-H < C6-H < C7-H with BDEs equal to 356.2 < 369.2 <

372.0 < 395.7 < 397.2 kJ/mol, respectively (**Table S1**). All in all, based on the results, the O-H bonds are more potent in SW, and easier donate H atom from these to a free radical than from the C-H bonds. However, there are some potentially good free radical scavengers within the C-H sites as well.

Table S1. Bond dissociation enthalpy (BDE) (in kJ/mol) for all unique C-H and O-H bonds in santowhite (SW) calculated at the M05-2X/6-311++G(2d,2p) level of theory in gas phase.

**X-H positions**	**BDE (kJ/mol)**
**SW**	**M05-2X/ 6-311++G(2d,2p)**
O1-H	351.5
O2-H	351.2
C1-H	461.2
C2-H	474.8
C3-H	356.2
C4-H	456.8
C5-H	472.2
C6-H	395.7
C7-H	397.2
C8-H	418.2
C9-H	372.0
C10-H	369.2
C11-H	425.6
C12-H	420.1

In case of Asc, there are four O-H bonds and three C-H bonds for which the bond dissociation enthalpies have been computed (**Table S2**). The BDEs of the O1-H has the highest antioxidant potential within Asc and its BDE is lower by 30 kJ/mol and 114.5 kJ/mol, than that of O2-H, and O3-H≈O4-H, respectively. The BDEs of the C-H bonds are higher than O1-H and O2-H, but lower than O3-H and O4-H and covers a range from 351.2 to 387.7 kJ/mol where the weakest bond is C1-H, while the strongest one is C3-H. Thus, it can be concluded that the O1-H followed by O2-H have the highest contribution to the antioxidant activity of Asc in case of the HAT mechanism. By comparing Asc with SW, the natural antioxidant has higher antioxidant potential than its synthetic counterpart.

Table S2. Bond dissociation enthalpy (BDE) values (in kJ/mol) for all C-H and O-H bonds of ascorbic acid (Asc) at the M05-2X/6-311++G(2d,2p) level of theory in gas phase.

**X-H positions**	**BDE (kJ/mol)**
**Asc**	**M05-2X/ 6-311++G(2d,2p)**
O1-H	318.7
O2-H	349.1
O3-H	433.2
O4-H	433.3
C1-H	351.2
C2-H	381.7
C3-H	387.7

1.2 Single electron transfer-proton transfer (SET-PT)

The IP for SW was computed, and it was found that it is 717.7 kJ/mol at the M05-2X/6-311++G(2d,2p) level of theory (***Table** S3*). PDEs indicates that O-H bonds are more prone to deprotonation in case of the second step of the SET-PT mechanism of SW than C-H bonds, but C3-H is a close competitor of O-H groups. In the case of Asc, IP was found to be 818.6 kJ/mol (***Table** S4*). The PDEs for all C-H and O-H sites cover a range between 814.0 and 928.6 kJ/mol, where the weakest one is O1-H and the strongest one is O3-H. The value of PDE for these C-H and O-H bonds can be arranged in the following order: O1-H < O2-H < C1-H < C2-H < C3-H < O4-H < O3-H with PDE values equal to 814.0 < 834.2 < 836.6 < 877.1 < 884.8 < 921.6 < 928.6 kJ/mol, respectively.

Table S3. Calculated ionization potential (IP) and lowest proton dissociation enthalpy (PDE) values in kJ/mol for santowhite (SW) determined at the M05-2X/6-311++G(2d,2p) level of theory in gas phase.

**M05-2X/6-311++G(2d,2p)**			
**Compound**	**IP**	**PDE**	**IP+PDE**
SW	717.7		
**O1-H**		947.8	1665.5
**O2-H**		947.5	1665.2
**C3-H**		952.4	1670.1

Table S4. Calculated ionization potential (IP) and proton dissociation enthalpy (PDE) values in kJ/mol for ascorbic acid (Asc) determined at the M05-2X/6-311++G(2d,2p) level of theory in gas phase.

M05-2X/6-311++G(2d,2p)			
**Compound**	**IP**	**PDE**	**IP+PDE**
**Asc**	818.6		
O1-H		814.0	1632.6
O2-H		834.2	1652.7
O3-H		928.6	1747.2
O4-H		921.6	1740.2
C1-H		836.6	1655.2
C2-H		877.1	1695.7
C3-H		884.8	1703.4

1.3 Sequential proton loss electron transfer (SPLET)

To determine the antioxidant potential in case of the SPLET mechanism, PA and ETE values have been computed for SW (***Table S5***).

Table S5. Lowest proton affinities (PAs) and electron transfer enthalpies (ETE) in kJ/mol for santowhite (SW) calculated at the M05-2X/6-311++G(2d,2p) level of theory in gas phase.

M05-2X/6-311++G(2d,2p)			
**SW**	**PA**	**ETE**	**PA+ETE**
O1-H	1438.4	227.1	1665.5
O2-H	1431.6	233.6	1665.2
C3-H	1563.6	106.6	1670.1

The best proton transfer ability corresponds to O2-H with PA equal to 1431.6 kJ/mol and it is lower by about 6.8 kJ/mol than O1-H (1438.4 kJ/mol) according to the M05-2X/6-311++G(2d,2p). The antioxidant potential of the different X-H bonds of SW in case of the SPLET mechanism was determined by calculating the sum of PA and ETE and the O-H and C-H sites were ranked as follows: O2-H > O1-H> C3-H at both levels of theory. As for Asc, only four sites (O1-H, O2-H, C1-H, and C3-H) were considered in the comparison (***Table S6***), because in case of others (O3-H, O4-H, and C2-H) intramolecular

rearrangement is occurred after the proton transfer and thus, the SPLET was not comparable.

Table S6. Proton affinities (PAs) and electron transfer enthalpies (ETEs) in kJ/mol for ascorbic acid (Asc) calculated at the M05-2X/6-311++G(2d,2p) level of theory in the gas phase.

**M05-2X/6-311++G(2d,2p)**			
**Asc**	**PA**	**ETE**	**PA+ETE**
O1-H	1329.3	304.0	1633.3
O2-H	1382.3	276.6	1658.9
O3-H	**-***	-*	-*
O4-H	**-***	-*	-*
C1-H	1450.5	199.4	1650.0
C2-H	**-***	-*	-*
C3-H	1624.9	69.4	1694.3

-* intramolecular rearrangement after proton transfer

Optimized structures

Structure of L-ascorbic acid (Asc) and the corresponding radical species in the HAT mechanism optimized at the M06-2X/6-311++G(2d,2p) level of theory in gas phase.

M06-2X/6-311++G(2d,2p)

Asc

C	0.26373400	0.19921000	-0.77160900
O	-0.18494300	-1.15485100	-0.63411300
C	-1.45792000	-1.16174300	-0.14698400
C	-1.90021800	0.21754200	0.03149000
C	-0.90391200	1.02236800	-0.32757700
O	-0.79434400	2.35645000	-0.33292300
H	-1.59141100	2.74913800	0.03845200
O	-3.12461800	0.52963200	0.50540400
H	-3.58325000	-0.29970000	0.69063600
O	-2.07973800	-2.15702700	0.09031100
C	1.51091900	0.40586000	0.09027600
H	1.78596800	1.46101800	0.03520700
C	2.66511400	-0.42661400	-0.43926400
H	2.43393900	-1.48806700	-0.29801200
H	2.77632700	-0.24143100	-1.51327900
O	1.23040500	0.14349900	1.44063900
H	1.07484800	-0.80013400	1.54435800
O	3.82794300	-0.04996700	0.26507200
H	4.54413700	-0.64258400	0.03326300
H	0.49550500	0.38013600	-1.82374700

Asc (O1•)

C	0.232115	0.267406	-0.891882
O	-0.236826	-1.093246	-0.867959
C	-1.433049	-1.173255	-0.250386
C	-1.894121	0.173607	0.076449
C	-0.913927	1.127832	-0.348040
O	-0.916827	2.337448	-0.296442
O	-3.045179	0.385877	0.661304
H	-3.459921	-0.477789	0.824431
O	-2.034522	-2.188124	-0.034783
C	1.459032	0.409888	-0.011593
H	1.776589	1.457787	-0.068965
C	2.593062	-0.486243	-0.469220
H	2.248295	-1.522118	-0.482236
H	2.916016	-0.194903	-1.472850
O	1.060785	0.080973	1.298790
H	1.861179	-0.027257	1.822299
O	3.635052	-0.304117	0.477428
H	4.272609	-1.014564	0.396176
H	0.459533	0.534395	-1.923372

Asc (O2•)

C	0.272526	0.233934	-0.804262
O	-0.190300	-1.116732	-0.674549
C	-1.455998	-1.187852	-0.145820
C	-1.945554	0.231577	0.070553
C	-0.879849	1.046817	-0.342821
O	-0.862952	2.355194	-0.305510
H	-1.709545	2.653309	0.067377
O	-3.029319	0.607140	0.514226
O	-2.000320	-2.216070	0.074993
C	1.519552	0.427332	0.066163
H	1.822373	1.474259	-0.005937
C	2.654568	-0.446513	-0.435143
H	2.381153	-1.498966	-0.302958
H	2.797424	-0.263314	-1.505729
O	1.202890	0.197964	1.414417
H	1.166780	-0.749868	1.573406
O	3.808176	-0.110647	0.301823
H	4.514424	-0.719882	0.081893
H	0.505705	0.424753	-1.855158

Asc (O3•)

C	0.250630	0.187902	-0.858935
O	-0.201391	-1.159819	-0.718011
C	-1.431757	-1.163200	-0.135546
C	-1.848095	0.220791	0.091794
C	-0.877022	1.020652	-0.336848
O	-0.748761	2.356163	-0.340705
H	-1.520013	2.757378	0.073569
O	-3.031213	0.533124	0.655999
H	-3.479682	-0.296653	0.864733
O	-2.051066	-2.151439	0.128422
C	1.541008	0.368051	-0.054961
H	1.854479	1.424667	-0.103484
C	2.712420	-0.467352	-0.606293
H	2.436940	-1.523900	-0.557835
H	2.880959	-0.198996	-1.648416
O	1.372926	0.090900	1.270541
O	3.892545	-0.193015	0.096367
H	3.722626	-0.345887	1.030413
H	0.438104	0.383502	-1.917560

Asc (O4•)

C	0.316283	0.207363	-0.764548
O	-0.129986	-1.146276	-0.622863
C	-1.406613	-1.152510	-0.138784
C	-1.849344	0.227658	0.032897
C	-0.852542	1.031821	-0.327642
O	-0.741766	2.365278	-0.339524
H	-1.541444	2.762072	0.022183
O	-3.073787	0.543069	0.502306
H	-3.536352	-0.283590	0.689657
O	-2.025871	-2.147549	0.101038
C	1.561305	0.409536	0.100392
H	1.841571	1.463038	0.045018
C	2.713934	-0.442023	-0.430508
H	2.460067	-1.512345	-0.327063
H	2.868039	-0.290742	-1.509362
O	1.290682	0.145858	1.450132
H	1.074829	-0.786757	1.549137
O	3.890054	-0.267911	0.236616
H	0.552679	0.384939	-1.816671

Asc (C1•)

C	0.198663	0.107725	0.274989
O	-0.244817	-1.178783	0.399222
C	-1.609046	-1.182461	0.137311
C	-1.973724	0.174113	-0.163843
C	-0.836721	0.956909	-0.071007
O	-0.697404	2.284673	-0.267935
H	-1.559509	2.671677	-0.445973
O	-3.212523	0.577093	-0.472103
H	-3.795239	-0.193162	-0.443513
O	-2.293733	-2.173188	0.171016
C	1.641368	0.351625	0.546449
H	1.821269	1.425600	0.500353
C	2.499066	-0.331399	-0.512304
H	2.375662	-1.416652	-0.420613
H	2.142540	-0.027251	-1.502001
O	1.985455	-0.052542	1.854329
H	1.814638	-0.995103	1.940766
O	3.835583	0.061342	-0.300776
H	4.401450	-0.397616	-0.923021

Asc (C2•)

C	0.182976	0.086378	-0.796385
O	-0.391511	-1.253190	-0.781378
C	-1.651022	-1.194391	-0.287145
C	-1.977208	0.196247	0.022597
C	-0.925240	0.953191	-0.272101
O	-0.725340	2.273142	-0.168056
H	-1.501445	2.682707	0.228249
O	-3.176056	0.558947	0.525348

Asc (C3•)

C	0.285633	0.309648	-0.776983
O	-0.124482	-1.062697	-0.719437
C	-1.400583	-1.133415	-0.248027
C	-1.885009	0.220207	0.002486
C	-0.908777	1.072222	-0.297833
O	-0.837587	2.406885	-0.224189
H	-1.650371	2.754417	0.157904
O	-3.122926	0.469123	0.479018

H	-3.697580	-0.247919	0.627235	H	-3.559263	-0.382540	0.608453
O	-2.370622	-2.142523	-0.144227	O	-1.995720	-2.157873	-0.073303
C	1.419657	0.146014	0.010147	C	1.521722	0.498651	0.103696
C	2.671665	-0.485716	-0.493455	H	1.774774	1.560927	0.107867
H	2.566441	-1.579954	-0.499390	C	2.661444	-0.273799	-0.447835
H	2.869636	-0.153490	-1.515642	H	2.529027	-1.289557	-0.799658
O	1.234419	0.148303	1.346942	O	1.218226	0.180042	1.455053
H	2.107416	0.108663	1.759316	H	1.089890	-0.771779	1.514455
O	3.714971	-0.103285	0.393849	O	3.878563	0.095074	0.023272
H	4.436743	-0.729366	0.324450	H	4.551654	-0.518985	-0.276294
H	0.414619	0.317959	-1.837963	H	0.528331	0.554250	-1.813033

SET-PT (SET step) mechanism

M06-2X/6-311++G(2d,2p)			
Asc$^{•+}$			
C	0.24144500	0.29045300	-0.97749000
O	-0.18060500	-1.08015100	-0.92389800
C	-1.29456900	-1.23274500	-0.19226900
C	-1.80387300	0.12990000	0.14296600
C	-0.90666400	1.05967600	-0.39378400
O	-0.92118200	2.34164000	-0.37027700
H	-1.68148500	2.72124800	0.10211400
O	-2.87883900	0.36462300	0.80486800
H	-3.30690200	-0.47403100	1.07346100
O	-1.83430700	-2.23939500	0.12057300
C	1.45395900	0.50583100	-0.06827200
H	1.76805800	1.55052500	-0.17492600
C	2.61844200	-0.40646500	-0.41393100
H	2.28515500	-1.44630000	-0.42223400
H	3.02077800	-0.14120000	-1.39564100
O	0.97037200	0.25641300	1.23008800
H	1.73267900	0.11793000	1.80732200
O	3.54608600	-0.16505400	0.62143800
H	4.27130700	-0.79237900	0.57782700
H	0.44576900	0.55970800	-2.01358500

SET-PT (PT step) mechanism

M06-2X/6-311++G(2d,2p)											
Asc$^{•+}$				Asc (O1$^{•}$)				Asc (O2$^{•}$)			
C	0.24144500	0.29045300	-0.97749000	C	0.252814	0.266727	-0.876929	C	0.256229	0.286093	-0.866552
O	-0.18060500	-1.08015100	-0.92389800	O	-0.171519	-1.105626	-0.783274	O	-0.156227	-1.081870	-0.797185
C	-1.29456900	-1.23274500	-0.19226900	C	-1.362883	-1.193289	-0.157424	C	-1.393760	-1.227376	-0.224964
C	-1.80387300	0.12990000	0.14296600	C	-1.866938	0.152593	0.103301	C	-1.934920	0.161463	0.061604
C	-0.90666400	1.05967600	-0.39378400	C	-0.919497	1.115649	-0.372488	C	-0.920758	1.037104	-0.358188
O	-0.92118200	2.34164000	-0.37027700	O	-0.961832	2.325612	-0.381796	O	-0.960145	2.344028	-0.279508
H	-1.68148500	2.72124800	0.10211400	O	-3.022933	0.356239	0.681534	H	-1.807935	2.589619	0.127066
O	-2.87883900	0.36462300	0.80486800	H	-3.408528	-0.511310	0.889491	O	-3.018418	0.476008	0.550144
H	-3.30690200	-0.47403100	1.07346100	O	-1.930376	-2.215001	0.111412	O	-1.895394	-2.283505	-0.038479
O	-1.83430700	-2.23939500	0.12057300	C	1.476265	0.492704	-0.009409	C	1.496103	0.519527	-0.012205

C	1.45395900	0.50583100	-0.06827200	H	1.759502	1.546145	-0.120249	H	1.816272	1.556239	-0.175974
H	1.76805800	1.55052500	-0.17492600	C	2.637964	-0.387942	-0.425822	C	2.623904	-0.414889	-0.405833
C	2.61844200	-0.40646500	-0.41393100	H	2.327227	-1.433825	-0.385299	H	2.284125	-1.447020	-0.305572
H	2.28515500	-1.44630000	-0.42223400	H	2.948970	-0.137260	-1.444100	H	2.922008	-0.226235	-1.441375
H	3.02077800	-0.14120000	-1.39564100	O	1.091641	0.217088	1.317240	O	1.123158	0.322393	1.328340
O	0.97037200	0.25641300	1.23008800	H	1.896108	0.162608	1.842849	H	1.933436	0.231358	1.840404
H	1.73267900	0.11793000	1.80732200	O	3.675523	-0.124428	0.506432	O	3.684381	-0.131796	0.494021
O	3.54608600	-0.16505400	0.62143800	H	4.336224	-0.816509	0.458076	H	4.322577	-0.846112	0.474178
H	4.27130700	-0.79237900	0.57782700	H	0.469377	0.488804	-1.921311	H	0.477890	0.529229	-1.909033
H	0.44576900	0.55970800	-2.01358500								

Asc (O3$^{\bullet}$)				Asc (O4$^{\bullet}$)				Asc (C1$^{\bullet}$)			
C	0.245569	0.195121	-0.836945	C	0.239865	0.272171	-0.825099	C	0.172793	0.117446	0.263124
O	-0.175098	-1.164877	-0.718629	O	-0.123778	-1.104952	-0.725705	O	-0.196369	-1.194426	0.325854
C	-1.407162	-1.206191	-0.141130	C	-1.361880	-1.205537	-0.168050	C	-1.560684	-1.258771	0.098482
C	-1.856656	0.163950	0.106016	C	-1.878350	0.140238	0.073550	C	-2.011880	0.090735	-0.112015
C	-0.903076	0.992937	-0.306209	C	-0.956676	1.015182	-0.315526	C	-0.917093	0.927888	-0.008182
O	-0.806313	2.330999	-0.288696	O	-0.925261	2.354612	-0.292257	O	-0.855879	2.270113	-0.137024
H	-1.588154	2.707355	0.129048	H	-1.721431	2.688950	0.133733	H	-1.742440	2.615362	-0.276477
O	-3.048848	0.439762	0.670273	O	-3.092156	0.360388	0.618411	O	-3.280313	0.436565	-0.363340
H	-3.478646	-0.403413	0.864039	H	-3.485218	-0.501703	0.805265	H	-3.815211	-0.368407	-0.363641
O	-2.004031	-2.212682	0.104881	O	-1.914926	-2.240867	0.061587	O	-2.192333	-2.285160	0.085361
C	1.528598	0.392921	-0.025515	C	1.492157	0.547214	-0.005613	C	1.597390	0.463736	0.481090
H	1.817701	1.457103	-0.057158	H	1.772878	1.592497	-0.184675	H	1.714917	1.528995	0.256876
C	2.721420	-0.406343	-0.584794	C	2.638969	-0.364833	-0.447763	C	2.498606	-0.338316	-0.448727
H	2.471114	-1.469738	-0.552504	H	2.293189	-1.411759	-0.358225	H	2.330296	-1.402710	-0.272471
H	2.886919	-0.118810	-1.622278	H	2.928747	-0.212204	-1.494836	H	2.267920	-0.097196	-1.489452
O	1.362534	0.091874	1.294959	O	1.194208	0.334024	1.346463	O	1.930051	0.198018	1.828843
O	3.892236	-0.114101	0.126205	H	2.030067	0.232691	1.814282	H	2.891949	0.218408	1.881289
H	3.723005	-0.285090	1.057214	O	3.707307	-0.285274	0.396402	O	3.826215	0.035261	-0.114507
H	0.431835	0.411519	-1.891748	H	0.436605	0.501768	-1.875419	H	4.441920	-0.597022	-0.487148

Asc (C2$^{\bullet}$)				Asc (C3$^{\bullet}$)			
C	0.210300	0.104590	-0.753429	C	0.267457	0.283352	-0.791973
O	-0.314907	-1.254258	-0.701738	O	-0.092464	-1.094144	-0.693590
C	-1.576397	-1.227920	-0.209785	C	-1.340866	-1.196552	-0.169794
C	-1.953612	0.157879	0.061375	C	-1.871543	0.147831	0.051514
C	-0.929607	0.944375	-0.253692	C	-0.945126	1.024909	-0.319562
O	-0.778136	2.273142	-0.185970	O	-0.923691	2.365701	-0.305886
H	-1.569486	2.664887	0.198013	H	-1.731206	2.695667	0.101432
O	-3.165656	0.490044	0.553499	O	-3.102339	0.360900	0.561380
H	-3.657281	-0.332374	0.677413	H	-3.489367	-0.505188	0.742937
O	-2.261130	-2.197381	-0.041020	O	-1.900558	-2.231835	0.050836
C	1.442786	0.231571	0.051706	C	1.496259	0.579678	0.059084
C	2.717915	-0.367023	-0.434023	H	1.741142	1.641797	-0.101119
H	2.653150	-1.464155	-0.410375	C	2.633265	-0.283760	-0.349364
H	2.905041	-0.055436	-1.464710	H	2.769106	-0.613558	-1.370131
O	1.255743	0.263803	1.387834	O	1.150312	0.389859	1.416451
H	2.129016	0.267568	1.801507	H	1.945654	0.141286	1.894964
O	3.745108	0.077467	0.443163	O	3.734895	-0.118235	0.438737
H	4.489924	-0.522716	0.390413	H	4.427432	-0.728656	0.178335
H	0.434797	0.315635	-1.800906	H	0.492105	0.507475	-1.837668

SPLET (SP step) mechanism

M06-2X/6-311++G(2d,2p)

Asc			
C	0.26373400	0.19921000	-0.77160900
O	-0.18494300	-1.15485100	-0.63411300
C	-1.45792000	-1.16174300	-0.14698400
C	-1.90021800	0.21754200	0.03149000
C	-0.90391200	1.02236800	-0.32757700
O	-0.79434400	2.35645000	-0.33292300
H	-1.59141100	2.74913800	0.03845200
O	-3.12461800	0.52963200	0.50540400
H	-3.58325000	-0.29970000	0.69063600
O	-2.07973800	-2.15702700	0.09031100
C	1.51091900	0.40586000	0.09027600
H	1.78596800	1.46101800	0.03520700
C	2.66511400	-0.42661400	-0.43926400
H	2.43393900	-1.48806700	-0.29801200
H	2.77632700	-0.24143100	-1.51327900
O	1.23040500	0.14349900	1.44063900
H	1.07484800	-0.80013400	1.54435800
O	3.82794300	-0.04996700	0.26507200
H	4.54413700	-0.64258400	0.03326300
H	0.49550500	0.38013600	-1.82374700

Asc (O1$^-$)			
C	0.291006	0.198753	-0.867734
O	-0.157527	-1.155240	-0.834327
C	-1.375902	-1.173844	-0.182320
C	-1.745765	0.136604	0.207497
C	-0.815228	1.065198	-0.236518
O	-0.716345	2.300476	-0.176249
O	-2.949724	0.353505	0.845063
H	-3.349845	-0.521841	0.907408
O	-1.989019	-2.215327	-0.013708
C	1.588178	0.355172	-0.090394
H	1.851288	1.418209	-0.123457
C	2.714942	-0.479590	-0.663277
H	2.381365	-1.518110	-0.738138
H	2.977944	-0.117570	-1.657621
O	1.445536	-0.057407	1.262509
H	0.522511	0.061399	1.517853
O	3.881355	-0.382869	0.140602
H	3.573997	-0.472326	1.048260
H	0.451131	0.498406	-1.906303

Asc (O2$^-$)			
C	0.300067	0.219068	-0.848964
O	-0.128695	-1.143394	-0.807552
C	-1.369623	-1.230957	-0.194099
C	-1.825820	0.149336	0.209244
C	-0.813566	0.955715	-0.211289
O	-0.805981	2.323176	-0.087237
H	-1.651181	2.509451	0.346603
O	-2.910902	0.456165	0.783111
O	-1.901665	-2.296818	-0.073801
C	1.628104	0.375469	-0.104151
H	1.931521	1.425252	-0.205008
C	2.717281	-0.518004	-0.659099
H	2.365137	-1.552276	-0.646060
H	2.948109	-0.233705	-1.686385
O	1.496985	0.052227	1.267492
H	0.563756	0.168985	1.497241
O	3.915235	-0.382813	0.091432
H	3.646691	-0.428703	1.014351
H	0.466281	0.494262	-1.898866

Asc (O3$^-$)			
C	0.064913	0.584988	-1.158724
O	-0.362593	-0.676130	-1.665491
C	-1.395022	-1.117420	-0.862065
C	-1.637520	-0.189625	0.183967
C	-0.758251	0.865849	0.101496
O	-0.516253	1.873448	0.814336
H	1.171227	2.143275	0.218559
O	-2.604253	-0.451745	1.129471
H	-2.966945	-1.307893	0.873809
O	-1.960085	-2.176046	-1.078366
C	1.558821	0.589989	-0.851590
H	2.129081	0.423475	-1.769488
C	1.962548	-0.468053	0.164330
H	1.353112	-0.354997	1.066462
H	1.809639	-1.467289	-0.240262
O	1.918652	1.872820	-0.360584
O	3.342260	-0.327636	0.471255
H	3.474751	0.621527	0.571531
H	-0.135400	1.355452	-1.907349

Asc (O4$^-$)			
C	0.156941	0.207107	-0.892737
O	-0.220614	-1.146056	-0.578826
C	-1.414576	-1.144551	0.028942
C	-1.955950	0.206040	0.009110
C	-1.066295	0.997242	-0.581447
O	-1.099071	2.326451	-0.806859
H	-1.801124	2.704422	-0.269607
O	-3.167837	0.495405	0.558034
H	-3.461308	-0.327329	0.970406
O	-1.971661	-2.121151	0.477279
C	1.344316	0.670647	0.016952
H	1.586893	1.675504	-0.396812
C	2.589833	-0.273035	-0.237289
H	2.436652	-0.923553	-1.107553
H	3.480039	0.341507	-0.428517
O	1.017053	0.655470	1.310754
H	2.080350	-0.569942	1.502716
O	2.756947	-1.038564	0.919798
H	0.434714	0.237731	-1.949126

Asc (C1$^-$)			
C	0.278320	0.137346	-0.091363
O	-0.177792	-1.181177	0.030575
C	-1.567492	-1.173233	0.081379
C	-1.958080	0.162476	0.038963
C	-0.815706	0.959849	-0.065491
O	-0.797584	2.327965	-0.120218
H	-1.715511	2.611038	-0.112508
O	-3.273999	0.604385	-0.016927
H	-3.791521	-0.201256	-0.124171
O	-2.235667	-2.218689	0.109734
C	1.700098	0.372297	0.174713
H	1.903402	1.439553	0.076546
C	2.619068	-0.411028	-0.755785
H	2.299895	-1.457981	-0.767202
H	2.539778	-0.013045	-1.766329
O	2.097314	0.016774	1.535192
H	1.465936	-0.651696	1.815645
O	3.976823	-0.301934	-0.351500
H	3.945700	-0.359045	0.610093

Asc (C2$^-$)			
C	0.587509	-0.280204	-0.231608
O	-1.546862	-1.847210	-1.634125
C	-2.367749	-1.130190	-1.053930
C	-1.882344	-0.031634	-0.105466
C	-0.638593	0.323339	0.241635
O	-0.488224	1.377664	1.145092
H	-1.379187	1.677938	1.357338
O	-2.921811	0.661228	0.444478
H	-3.675112	0.179564	0.010081
O	-3.627986	-1.139772	-1.104959
C	1.826809	0.062250	0.134446
C	3.064916	-0.627223	-0.338769
H	3.506430	-1.186869	0.495137
H	2.818147	-1.322888	-1.136417
O	2.163039	1.072257	1.006088
H	1.343244	1.499879	1.290614

Asc (C3$^-$)			
C	0.272384	0.226017	-0.798365
O	-0.247457	-1.122487	-0.796384
C	-1.486788	-1.127904	-0.295072
C	-1.892731	0.238205	0.002418
C	-0.883579	1.043110	-0.310047
O	-0.775272	2.381618	-0.213529
H	-1.538607	2.712860	0.267965
O	-3.118081	0.516499	0.526548
H	-3.534227	-0.343972	0.667663
O	-2.172715	-2.112318	-0.134296
C	1.520192	0.354995	0.082576
H	1.835029	1.406057	-0.004033
C	2.621567	-0.582169	-0.299337
H	2.480503	-0.929544	-1.328836
O	1.104429	0.168699	1.453504
H	1.547440	-0.671263	1.644353

O	4.027356	0.294398	-0.841252	O	3.894532	0.149882	-0.282029
H	3.948520	1.082731	-0.296429	H	4.395155	-0.251813	0.420977
H	0.444415	-1.083542	-0.939561	H	0.532773	0.474331	-1.828466

SPLET (ETE step) mechanism

M06-2X/6-311++G(2d,2p)											
Asc (O1•)				Asc (O2•)				Asc (C1•)			
C	0.226975	0.303394	-0.858163	C	0.184059	0.193676	-0.871290	C	0.172862	0.139574	0.193580
O	-0.230361	-1.062942	-0.849179	O	-0.213010	-1.174286	-0.737172	O	-0.263221	-1.146437	0.344947
C	-1.463162	-1.155597	-0.317478	C	-1.454382	-1.311074	-0.175317	C	-1.639605	-1.151812	0.143732
C	-1.929794	0.182524	0.044078	C	-2.006994	0.084033	0.061632	C	-2.016084	0.203176	-0.151567
C	-0.910182	1.137184	-0.264523	C	-0.997516	0.950501	-0.388769	C	-0.876127	0.986831	-0.112546
O	-0.865163	2.333102	-0.059270	O	-1.039744	2.261924	-0.326441	O	-0.743978	2.312891	-0.321489
O	-3.099213	0.378727	0.594845	H	-1.904033	2.510799	0.043196	H	-1.610772	2.699193	-0.477619
H	-3.532536	-0.486667	0.690019	O	-3.087177	0.413281	0.546975	O	-3.266282	0.606014	-0.406206
O	-2.085830	-2.169502	-0.170992	O	-1.952844	-2.361222	0.046752	H	-3.848942	-0.162799	-0.350451
C	1.489524	0.459200	-0.022424	C	1.442044	0.473960	-0.044769	O	-2.321016	-2.141270	0.219342
H	1.857779	1.476832	-0.177322	H	1.751225	1.504841	-0.241693	C	1.627190	0.377278	0.355770
C	2.571945	-0.528653	-0.416660	C	2.577457	-0.460631	-0.423670	H	1.800133	1.450704	0.430215
H	2.200807	-1.544121	-0.256755	H	2.269168	-1.489883	-0.225356	C	2.422437	-0.174074	-0.829138
H	2.815122	-0.410077	-1.472379	H	2.799397	-0.363053	-1.486199	H	2.203069	-1.243359	-0.934830
O	1.210572	0.232715	1.350216	O	1.189497	0.276145	1.333669	H	2.121806	0.331917	-1.746001
H	0.910324	1.050986	1.753439	H	0.902140	1.096453	1.738961	O	2.118684	-0.196109	1.559151
O	3.751622	-0.287686	0.312580	O	3.748821	-0.121434	0.278471	H	1.759894	-1.085870	1.643062
H	3.527830	-0.354226	1.245144	H	3.561621	-0.225569	1.215475	O	3.795658	0.057059	-0.646947
H	0.415457	0.588638	-1.893933	H	0.390976	0.392608	-1.926804	H	4.014642	-0.212616	0.250638
Asc (C3•)											
C	0.338181	0.075840	-0.769988								
O	-0.151201	-1.234426	-0.472596								
C	-1.410087	-1.140690	0.043024								
C	-1.797643	0.266786	0.084845								
C	-0.784767	0.988560	-0.386501								
O	-0.628950	2.309550	-0.543872								
H	-1.409775	2.769846	-0.218092								
O	-2.995114	0.675305	0.551336								
H	-3.480358	-0.110398	0.834186								
O	-2.060704	-2.079025	0.400343								
C	1.621741	0.328106	0.020789								
H	1.834657	1.403943	-0.029286								
C	2.754873	-0.478764	-0.514689								
H	2.921167	-0.592995	-1.575362								
O	1.441436	0.059174	1.400416								
H	1.164045	-0.862180	1.476673								
O	3.882639	-0.513276	0.230731								
H	3.638400	-0.318231	1.144379								
H	0.548901	0.134322	-1.840495								

Structures of santowhite (SW) and the corresponding radical species in the HAT mechanism optimized at the M06-2X/6-311++G(2d,2p) level of theory in gas phase.

M06-2X/6-311++G(2d,2p)											
SW				SW (O1•)				SW (O2•)			
C	-2.67469700	-0.88809800	-0.06533000	C	2.658923	0.882200	-0.076842	C	2.701272	0.890911	-0.075165
C	-1.49051600	-0.15717800	-0.02475800	C	1.471560	0.156821	-0.030254	C	1.517741	0.159126	-0.031340

C	-1.41417100	1.22111900	-0.21044200	C	1.391252	-1.224856	-0.186868	C	1.441998	-1.219346	-0.215972
C	-2.58638600	1.92518400	-0.49075900	C	2.562896	-1.939239	-0.442369	C	2.614079	-1.922706	-0.498606
C	-3.78020800	1.21581600	-0.55533800	C	3.760009	-1.235961	-0.512415	C	3.807211	-1.212481	-0.566446
C	-3.83614600	-0.15458700	-0.34159200	C	3.819652	0.138506	-0.327744	C	3.862619	0.158099	-0.353699
H	-0.56422600	-0.68096000	0.15836000	H	0.545903	0.687937	0.133965	H	0.591490	0.682327	0.153629
H	-4.70095500	1.74561100	-0.77700400	H	4.680412	-1.773897	-0.715088	H	4.727832	-1.741724	-0.789947
C	-0.08417000	1.94094100	-0.03587700	C	0.057033	-1.935699	-0.007755	C	0.112955	-1.940057	-0.037819
H	-0.11639200	2.84401300	-0.65122900	H	0.090656	-2.851933	-0.603256	H	0.144408	-2.843495	-0.652674
C	1.08885300	1.12039600	-0.55451400	C	-1.108482	-1.122072	-0.553509	C	-1.061905	-1.120724	-0.554208
C	1.16100900	0.79742800	-1.91592000	C	-1.168320	-0.828234	-1.922092	C	-1.137512	-0.798674	-1.915645
C	2.11018100	0.67754000	0.28152600	C	-2.134786	-0.657438	0.264422	C	-2.081594	-0.678107	0.283956
C	2.23976300	0.04566800	-2.35968800	C	-2.240450	-0.082120	-2.390745	C	-2.217875	-0.048007	-2.357344
C	3.19890400	-0.08696300	-0.13896100	C	-3.217022	0.101955	-0.181312	C	-3.171881	0.085310	-0.134447
H	2.05975100	0.94168100	1.32445800	H	-2.093858	-0.899241	1.313172	H	-2.028508	-0.941548	1.326933
C	3.23757100	-0.39732800	-1.50128700	C	-3.243415	0.383047	-1.550269	C	-3.213991	0.394782	-1.496874
H	2.31275100	-0.20270100	-3.41369200	H	-2.303907	0.143762	-3.450409	H	-2.293533	0.199639	-3.411330
C	0.07631600	2.42996200	1.42138500	C	-0.117197	-2.392603	1.458325	C	-0.043729	-2.428275	1.420126
H	1.06319300	2.88574500	1.51686900	H	-1.106617	-2.842476	1.555507	H	-1.030034	-2.884740	1.518224
H	-0.64355200	3.23527900	1.58323200	H	0.598133	-3.196973	1.643345	H	0.677126	-3.232947	1.580786
C	-0.13989500	1.37000700	2.51921000	C	0.094316	-1.310074	2.534831	C	0.174269	-1.367462	2.516769
H	0.58518500	1.53223900	3.31962500	H	-0.637865	-1.452274	3.332586	H	-0.548800	-1.529732	3.318992
H	0.06298300	0.37290100	2.12532200	H	-0.101404	-0.320921	2.117939	H	-0.030288	-0.370759	2.122730
C	-1.54749400	1.39404400	3.11219800	C	1.496957	-1.326672	3.139703	C	1.583279	-1.390064	3.106453
H	-1.66369500	0.63048600	3.88153600	H	1.609949	-0.547165	3.893365	H	1.700717	-0.625931	3.875032
H	-1.75316600	2.36329200	3.56957100	H	1.695066	-2.286616	3.619487	H	1.790759	-2.358867	3.563953
H	-2.30196700	1.21701700	2.34508000	H	2.258318	-1.169075	2.375158	H	2.335809	-1.212956	2.337447
C	-2.59016000	3.41059700	-0.74282400	C	2.562816	-3.429740	-0.662386	C	2.618379	-3.408275	-0.749740
H	-2.13947400	3.96279200	0.08169800	H	2.103275	-3.962332	0.170105	H	2.170054	-3.960286	0.076191
H	-2.02523000	3.65692400	-1.64418600	H	2.004236	-3.693281	-1.562836	H	2.051513	-3.655599	-1.649612
H	-3.60591800	3.77696300	-0.87640100	H	3.578161	-3.802750	-0.779730	H	3.634096	-3.773962	-0.885479
C	0.11503800	1.23942700	-2.90510300	C	-0.116130	-1.295404	-2.892942	C	-0.093542	-1.240512	-2.907010
H	-0.81417400	0.68382700	-2.77235900	H	0.814174	-0.740603	-2.764576	H	0.835561	-0.684129	-2.776807
H	-0.12563400	2.29658600	-2.78454700	H	0.119359	-2.350626	-2.747708	H	0.148211	-2.297413	-2.786353
H	0.46384700	1.08774400	-3.92474000	H	-0.456060	-1.164398	-3.918427	H	-0.444866	-1.089738	-3.925918
O	4.28022400	-1.13970500	-1.99211400	O	-4.279101	1.118629	-2.065471	O	-4.258357	1.136063	-1.985712
H	4.15038500	-1.27447200	-2.93347100	O	5.025359	0.785734	-0.398260	H	-4.130838	1.270331	-2.927458
O	-5.03877100	-0.80796300	-0.40791800	H	5.715155	0.143103	-0.578146	O	5.064592	0.812337	-0.423272
H	-5.72950400	-0.17209300	-0.60719000	C	2.693737	2.396327	0.144922	C	2.731615	2.400227	0.177847
C	-2.70558000	-2.39723100	0.18870900	C	3.563571	2.721347	1.371136	C	3.612878	2.703685	1.401423
C	-3.58418500	-2.69925100	1.41455000	H	4.589017	2.388049	1.236104	H	4.638330	2.378119	1.248727
H	-4.60974900	-2.37300800	1.26406400	H	3.569740	3.799559	1.540801	H	3.615990	3.778139	1.593531
H	-3.58765200	-3.77358000	1.60734600	H	3.155805	2.239246	2.261172	H	3.216705	2.201197	2.285397
H	-3.18555200	-2.19650300	2.29727000	C	1.293525	2.958414	0.410852	C	1.331776	2.950032	0.470112
C	-1.30547000	-2.94790600	0.47802400	H	0.619125	2.787079	-0.429138	H	0.649321	2.793085	-0.366176
H	-0.62487000	-2.79200200	-0.35996900	H	0.847203	2.523911	1.306694	H	0.896896	2.494827	1.361290
H	-0.86814900	-2.49246600	1.36788600	H	1.369144	4.035001	0.565001	H	1.404176	4.023512	0.645948
H	-1.37826300	-4.02122000	0.65471000	C	3.250976	3.104699	-1.100710	C	3.272479	3.137062	-1.058457
C	-3.24991000	-3.13443900	-1.04585000	H	4.268248	2.792160	-1.320404	H	4.288770	2.833977	-1.295272
H	-4.26652800	-2.83073800	-1.28046200	H	2.626103	2.890438	-1.969024	H	2.639442	2.938024	-1.924477
H	-2.61876600	-2.93642600	-1.91348500	H	3.249562	4.184204	-0.939629	H	3.267912	4.212951	-0.874824
H	-3.24571900	-4.21021500	-0.86154600	C	-4.307329	0.597434	0.772499	C	-4.254273	0.555627	0.840874
C	4.28323700	-0.55747900	0.83410600	C	-5.672249	0.021894	0.359950	C	-5.620871	-0.017610	0.430680
C	5.64929600	0.01446800	0.42033000	H	-6.441140	0.370270	1.052047	H	-6.383970	0.312616	1.137920
H	6.41381100	-0.31588600	1.12597900	H	-5.950480	0.328211	-0.644750	H	-5.911070	0.308293	-0.564426
H	5.93690400	-0.31228200	-0.57525100	H	-5.650259	-1.068116	0.396559	H	-5.593594	-1.108030	0.444249
H	5.62287100	1.10491700	0.43327100	C	-4.357884	2.134565	0.758669	C	-4.311883	2.092456	0.859731
C	4.33973500	-2.09433900	0.85380100	H	-4.588670	2.520202	-0.230629	H	-4.554834	2.497563	-0.118815
H	4.58007500	-2.50024700	-0.12505800	H	-5.125180	2.483138	1.452408	H	-5.073371	2.422853	1.568600
H	5.10264300	-2.42486100	1.56108300	H	-3.399407	2.547283	1.077358	H	-3.351960	2.502950	1.176937

H	3.38025400	-2.50390800	1.17352800	C	-4.038711	0.159159	2.216134	C	-3.968458	0.088591	2.272136
C	4.00114700	-0.08932300	2.26574100	H	-4.026554	-0.927242	2.314940	H	-3.950373	-0.999566	2.348063
H	3.98406000	0.99889500	2.34102000	H	-3.092273	0.552269	2.591066	H	-3.019891	0.478251	2.645287
H	3.05317000	-0.47803200	2.64137200	H	-4.834627	0.541653	2.855410	H	-4.759291	0.453902	2.927566
H	4.79324800	-0.45481600	2.91953700								
**SW (C1$^{\bullet}$)**				**SW (C2$^{\bullet}$)**				**SW (C3$^{\bullet}$)**			
C	2.677145	0.892165	-0.063842	C	2.702422	0.863584	-0.068503	C	2.675465	0.874565	-0.058522
C	1.493171	0.160879	-0.023809	C	1.513829	0.139693	-0.031026	C	1.491107	0.143516	-0.026299
C	1.417232	-1.217320	-0.210381	C	1.428475	-1.236517	-0.227930	C	1.414414	-1.232534	-0.227824
C	2.589665	-1.920869	-0.491080	C	2.595606	-1.945470	-0.517001	C	2.586442	-1.933615	-0.516284
C	3.783288	-1.211118	-0.555133	C	3.793647	-1.242990	-0.578687	C	3.780435	-1.223843	-0.572761
C	3.838820	0.159164	-0.340505	C	3.858614	0.125219	-0.353736	C	3.836720	0.143996	-0.343265
H	4.704200	-1.740507	-0.777084	H	0.591260	0.667658	0.158741	H	0.564954	0.665384	0.162882
C	0.087427	-1.937635	-0.036357	C	0.094463	-1.949520	-0.056009	H	4.701041	-1.751278	-0.800556
H	0.119944	-2.840303	-0.652286	H	0.119570	-2.847636	-0.678899	C	0.084245	-1.953991	-0.061481
C	-1.085800	-1.117094	-0.554537	C	-1.074711	-1.117457	-0.564895	C	-1.088602	-1.127247	-0.570582
C	-1.157968	-0.793273	-1.915740	C	-1.148196	-0.782761	-1.923394	C	-1.160747	-0.788620	-1.928179
C	-2.107305	-0.675067	0.281726	C	-2.091219	-0.675244	0.277337	C	-2.109780	-0.693788	0.270551
C	-2.236911	-0.041538	-2.359090	C	-2.223346	-0.020683	-2.358219	C	-2.239339	-0.031540	-2.363211
C	-3.196222	0.089394	-0.138335	C	-3.176202	0.099444	-0.134077	C	-3.198336	0.075768	-0.141055
H	-2.056861	-0.939862	1.324491	H	-2.039875	-0.948339	1.317916	H	-2.059363	-0.969923	1.310373
C	-3.234897	0.400621	-1.500464	C	-3.216279	0.421338	-1.493684	C	-3.236995	0.401795	-1.499718
H	-2.309908	0.207486	-3.412939	H	-2.297373	0.236872	-3.409943	H	-2.312318	0.228957	-3.414284
C	-0.073005	-2.427636	1.420582	C	-0.065486	-2.449617	1.397549	C	-0.076288	-2.459706	1.390066
H	-1.059757	-2.883763	1.515715	H	-1.054936	-2.900062	1.491722	H	-1.063272	-2.916316	1.480352
H	0.647084	-3.232851	1.581955	H	0.649764	-3.260688	1.550918	H	0.643390	-3.267008	1.542600
C	0.142837	-1.368324	2.519099	C	0.159990	-1.400168	2.503573	C	0.140238	-1.412505	2.500002
H	-0.582244	-1.531277	3.319367	H	-0.564120	-1.564548	3.304428	H	-0.584842	-1.583757	3.298536
H	-0.060303	-0.371024	2.125839	H	-0.037659	-0.398591	2.118468	H	-0.062415	-0.410883	2.117622
C	1.550408	-1.392338	3.112155	C	1.568861	-1.437834	3.092819	C	1.547860	-1.443707	3.092600
H	1.666344	-0.629240	3.881990	H	1.691684	-0.681421	3.868163	H	1.664287	-0.689079	3.870665
H	1.756331	-2.361820	3.568919	H	1.769632	-2.412099	3.541631	H	1.753317	-2.418203	3.538781
H	2.304875	-1.214603	2.345196	H	2.322537	-1.259118	2.325309	H	2.302339	-1.258051	2.327530
C	2.593880	-3.406119	-0.744097	C	2.589542	-3.428735	-0.781372	C	2.589839	-3.416030	-0.785422
H	2.143303	-3.958972	0.080044	H	2.137458	-3.984955	0.039672	H	2.139059	-3.977564	0.032716
H	2.029074	-3.652030	-1.645651	H	2.020887	-3.664072	-1.683328	H	2.024804	-3.651833	-1.689528
H	3.609751	-3.772108	-0.877849	H	3.602676	-3.800257	-0.920520	H	3.605501	-3.781084	-0.923259
C	-0.111812	-1.234338	-2.905144	C	-0.107415	-1.222998	-2.918817	C	-0.114934	-1.219465	-2.922436
H	0.817233	-0.678557	-2.771989	H	0.825550	-0.674278	-2.783800	H	0.814421	-0.665657	-2.783358
H	0.129156	-2.291505	-2.785252	H	0.126984	-2.282590	-2.807626	H	0.125485	-2.278000	-2.814063
H	-0.460604	-1.082101	-3.924704	H	-0.457773	-1.060702	-3.936287	H	-0.463756	-1.055976	-3.940242
O	-4.277734	1.143014	-1.990877	O	-4.255503	1.174200	-1.975735	O	-4.279490	1.150025	-1.981917
H	-4.147878	1.278422	-2.932140	H	-4.127136	1.315970	-2.916265	H	-4.149665	1.295582	-2.921668
O	5.041262	0.812927	-0.406340	O	5.065106	0.771670	-0.417655	O	5.039502	0.797798	-0.402133
H	5.732189	0.177383	-0.605979	H	5.751388	0.133214	-0.623915	H	5.730069	0.164093	-0.608744
C	2.707580	2.401143	0.191167	C	2.743294	2.370336	0.197951	C	2.706731	2.380668	0.212861
C	3.586026	2.702629	1.417254	C	3.626758	2.656730	1.424051	C	3.585471	2.668349	1.442050
H	4.611692	2.376776	1.266619	H	4.649905	2.325408	1.268303	H	4.610948	2.343608	1.287766
H	3.589174	3.776835	1.610739	H	3.637366	3.729380	1.625731	H	3.589212	3.740388	1.647193
H	3.187485	2.199200	2.299627	H	3.227175	2.149149	2.303570	H	3.186759	2.155577	2.318948
C	1.307295	2.951231	0.480752	C	1.347343	2.927243	0.495319	C	1.306771	2.928321	0.508566
H	0.626790	2.795670	-0.357381	H	0.663737	2.782514	-0.342232	H	0.626091	2.782234	-0.331129
H	0.870052	2.495095	1.370296	H	0.909384	2.467148	1.382469	H	0.869382	2.462780	1.393153
H	1.379770	4.024453	0.658131	H	1.427230	3.998583	0.680708	H	1.379836	3.999513	0.697585
C	3.251772	3.139299	-1.042887	C	3.289164	3.114384	-1.031816	C	3.251180	3.131897	-1.013163
H	4.268491	2.836040	-1.277633	H	4.303300	2.806356	-1.271478	H	4.267712	2.830666	-1.251307
H	2.620736	2.941661	-1.910686	H	2.654680	2.927487	-1.899480	H	2.619944	2.944034	-1.882986
H	3.247262	4.214955	-0.857893	H	3.292103	4.188598	-0.838596	H	3.247263	4.205482	-0.816495
C	-4.280747	0.558975	0.834969	C	-4.255206	0.568567	0.845563	C	-4.282505	0.535323	0.837418

C	-5.646618	-0.013098	0.420745	C	-5.625799	0.008537	0.430481	C	-5.648725	-0.031491	0.417161
H	-6.411269	0.316584	1.126560	H	-6.386518	0.337747	1.140753	H	-6.413124	0.290910	1.126605
H	-5.934261	0.314208	-0.574643	H	-5.913811	0.345312	-0.561636	H	-5.936303	0.306762	-0.574579
H	-5.619881	-1.103547	0.432988	H	-5.606112	-1.082124	0.434322	H	-5.622567	-1.122023	0.417555
C	-4.337687	2.095806	0.855646	C	-4.302115	2.105531	0.878131	C	-4.338625	2.071869	0.874796
H	-4.578085	2.502273	-0.122967	H	-4.542328	2.521028	-0.096728	H	-4.578914	2.489071	-0.099316
H	-5.100732	2.425655	1.563095	H	-5.061221	2.434885	1.590033	H	-5.101416	2.394419	1.585874
H	-3.378342	2.505445	1.175693	H	-3.339329	2.506488	1.198839	H	-3.379027	2.477497	1.199163
C	-3.998608	0.089982	2.266320	C	-3.972522	0.086809	2.272561	C	-4.000459	0.050659	2.263557
H	-3.981214	-0.998279	2.340902	H	-3.962006	-1.002081	2.338779	H	-3.983635	-1.038358	2.326311
H	-3.050765	0.478721	2.642257	H	-3.021233	0.466514	2.649027	H	-3.052367	0.434788	2.643586
H	-4.790852	0.454828	2.920303	H	-4.760735	0.451757	2.931341	H	-4.792437	0.408800	2.921557
**SW (C4·)**				**SW (C5·)**				**SW (C6·)**			
C	2.663447	0.886958	-0.070362	C	2.664330	0.887872	-0.071205	C	2.667552	0.878399	-0.084577
C	1.480402	0.154658	-0.022242	C	1.479699	0.157503	-0.033990	C	1.485080	0.146180	-0.023019
C	1.405719	-1.225080	-0.197612	C	1.403842	-1.221475	-0.214759	C	1.411671	-1.236783	-0.171455
C	2.578443	-1.929512	-0.474859	C	2.577257	-1.927000	-0.486249	C	2.585186	-1.945506	-0.434037
C	3.771117	-1.218927	-0.546844	C	3.771646	-1.218278	-0.547338	C	3.777323	-1.235456	-0.519024
C	3.825456	0.153096	-0.343318	C	3.826947	0.152920	-0.338584	C	3.830372	0.140326	-0.342301
H	0.553687	0.678447	0.158692	H	0.552650	0.682290	0.142280	H	0.557796	0.672622	0.146975
H	4.692228	-1.749021	-0.766270	H	4.693351	-1.749225	-0.762161	H	4.699036	-1.768958	-0.727384
C	0.077080	-1.945503	-0.015319	C	0.072699	-1.940182	-0.044356	C	0.083496	-1.954628	0.023912
H	0.109497	-2.853045	-0.624049	H	0.107812	-2.845629	-0.656049	H	0.117118	-2.873861	-0.566949
C	-1.098067	-1.130493	-0.537861	C	-1.097320	-1.121256	-0.572256	C	-1.091939	-1.150974	-0.515305
C	-1.173155	-0.817687	-1.901479	C	-1.162269	-0.803513	-1.935253	C	-1.166274	-0.864930	-1.884828
C	-2.118521	-0.682945	0.296747	C	-2.122835	-0.674847	0.256739	C	-2.113383	-0.688069	0.309659
C	-2.253798	-0.070773	-2.348818	C	-2.238435	-0.053115	-2.387529	C	-2.247204	-0.127809	-2.347457
C	-3.209110	0.076869	-0.127388	C	-3.209086	0.088388	-0.172358	C	-3.204287	0.062394	-0.130029
C	-3.250692	0.377114	-1.491894	H	-2.077932	-0.934983	1.300929	H	-2.061246	-0.923929	1.359265
H	-2.329053	0.169704	-3.404491	C	-3.240547	0.393513	-1.536052	C	-3.245105	0.335891	-1.500170
C	-0.080060	-2.423984	1.445801	C	-0.095547	-2.423531	1.413923	H	-2.321876	0.091936	-3.407681
H	-1.066101	-2.880466	1.546442	H	-1.083067	-2.878618	1.506017	C	-0.074331	-2.404607	1.493986
H	0.641266	-3.227050	1.612275	H	0.623187	-3.228454	1.582620	H	0.647537	-3.203659	1.676640
C	0.136601	-1.355645	2.535380	C	0.115311	-1.359424	2.508769	C	0.140635	-1.315005	2.562643
H	-0.586793	-1.513005	3.338289	H	-0.613986	-1.518332	3.306013	H	-0.583225	-1.457261	3.367947
H	-0.068434	-0.361766	2.134515	H	-0.085165	-0.363777	2.109991	H	-0.064927	-0.329320	2.142290
C	1.545305	-1.373283	3.125966	C	1.519793	-1.381638	3.109172	C	1.548913	-1.319914	3.154483
H	1.661791	-0.603901	3.889436	H	1.632250	-0.615159	3.876158	H	1.664195	-0.535666	3.902862
H	1.753208	-2.338833	3.590103	H	1.722741	-2.349184	3.571341	H	1.757274	-2.276036	3.637545
H	2.298131	-1.200841	2.356190	H	2.278314	-1.207816	2.345320	H	2.302167	-1.161906	2.382033
C	2.583914	-3.416738	-0.715963	C	2.581826	-3.413374	-0.732568	C	2.592069	-3.437153	-0.646037
H	2.135521	-3.963478	0.113430	H	2.126658	-3.962240	0.091715	H	2.143515	-3.967965	0.193553
H	2.017711	-3.670522	-1.614453	H	2.021514	-3.662987	-1.635906	H	2.026744	-3.708919	-1.539809
H	3.599958	-3.782621	-0.848682	H	3.598139	-3.780586	-0.859438	H	3.608515	-3.804701	-0.770846
C	-0.128336	-1.265474	-2.889276	C	-0.111313	-1.249664	-2.917262	C	-0.120351	-1.331045	-2.862930
H	0.800308	-0.707576	-2.762327	H	0.817388	-0.693862	-2.781818	H	0.807736	-0.769990	-2.746230
H	0.114085	-2.321367	-2.761361	H	0.128359	-2.306430	-2.791382	H	0.122851	-2.384033	-2.714236
H	-0.479209	-1.121822	-3.909368	H	-0.454752	-1.101797	-3.939280	H	-0.470586	-1.207645	-3.885888
O	-4.295306	1.114350	-1.986287	O	-4.280370	1.134336	-2.035162	O	-4.289964	1.062447	-2.009629
H	-4.167366	1.242351	-2.928847	H	-4.145581	1.265432	-2.976342	H	-4.161431	1.172118	-2.954417
O	5.027011	0.807690	-0.416652	O	5.030129	0.805641	-0.401158	O	5.031437	0.794377	-0.427537
H	5.718303	0.171359	-0.612484	H	5.721669	0.168781	-0.594377	H	5.723402	0.154925	-0.610398
C	2.692603	2.397967	0.172478	C	2.694415	2.397962	0.177176	C	2.695274	2.393888	0.128719
C	3.572987	2.710289	1.394456	C	3.566727	2.704416	1.406407	C	3.574491	2.730765	1.345001
H	4.598748	2.384417	1.244515	H	4.592947	2.377258	1.262525	H	4.600633	2.402891	1.202199
H	3.575246	3.786015	1.579313	H	3.569564	3.779478	1.595079	H	3.575720	3.809899	1.508809
H	3.176681	2.213498	2.281586	H	3.163325	2.205204	2.288967	H	3.177939	2.251065	2.241379
C	1.292223	2.948752	0.460270	C	1.293009	2.950208	0.457068	C	1.294224	2.949008	0.404680

H	0.610334	2.785690	-0.375310	H	0.616730	2.791299	-0.383854	H	0.613091	2.769078	-0.428050
H	0.857173	2.499266	1.354263	H	0.850899	2.498344	1.346386	H	0.858884	2.516704	1.306967
H	1.363780	4.023445	0.628899	H	1.365254	4.024171	0.629995	H	1.364764	4.026849	0.552333
C	3.233629	3.126823	-1.068476	C	3.245426	3.130229	-1.057360	C	3.236616	3.098820	-1.125844
H	4.250260	2.822859	-1.302691	H	4.263147	2.825292	-1.285499	H	4.253672	2.791196	-1.353338
H	2.601204	2.921505	-1.933476	H	2.618742	2.929081	-1.927503	H	2.605003	2.876109	-1.987126
H	3.228213	4.203923	-0.892107	H	3.240650	4.206708	-0.877225	H	3.230175	4.179154	-0.970555
C	-4.292361	0.552998	0.844153	C	-4.298310	0.563006	0.793226	C	-4.288654	0.556497	0.831238
C	-5.658336	-0.023948	0.437096	C	-5.662394	-0.010083	0.374543	C	-5.653847	-0.029444	0.434539
H	-6.422051	0.310508	1.141679	H	-6.430460	0.323239	1.074920	H	-6.418362	0.318067	1.131898
H	-5.948219	0.295031	-0.560344	H	-5.944697	0.312923	-0.623775	H	-5.943254	0.269744	-0.569152
H	-5.630308	-1.114233	0.458035	H	-5.636417	-1.100482	0.391821	H	-5.624930	-1.119088	0.476793
C	-4.351050	2.089879	0.852610	C	-4.354375	2.099949	0.806706	C	-4.348624	2.093199	0.809627
H	-4.593749	2.488205	-0.128778	H	-4.589470	2.502161	-0.174948	H	-4.590925	2.472073	-0.179531
H	-5.113155	2.424517	1.558822	H	-5.120842	2.433445	1.508725	H	-5.111531	2.440926	1.508613
H	-3.391587	2.503174	1.167560	H	-3.396430	2.510432	1.129849	H	-3.389738	2.513378	1.117139
C	-4.007003	0.095828	2.278688	C	-4.023846	0.100278	2.228104	C	-4.003981	0.127678	2.274637
H	-3.988203	-0.991779	2.361963	H	-4.007531	-0.987647	2.307662	H	-3.984340	-0.958078	2.379157
H	-3.058912	0.488659	2.649714	H	-3.077703	0.490120	2.607170	H	-3.056492	0.528483	2.638607
H	-4.798448	0.464999	2.931210	H	-4.819215	0.468548	2.876351	H	-4.796216	0.508858	2.919248
**SW (C7$^{\bullet}$)**				**SW (C8$^{\bullet}$)**				**SW (C9$^{\bullet}$)**			
C	2.671247	0.880661	-0.078128	C	2.685931	0.865795	-0.078819	C	2.700487	0.844096	-0.056989
C	1.487604	0.150462	-0.015092	C	1.499362	0.141048	-0.008296	C	1.509039	0.124466	-0.029428
C	1.411936	-1.232638	-0.161097	C	1.419564	-1.244341	-0.128042	C	1.418328	-1.248668	-0.244561
C	2.584259	-1.943714	-0.422638	C	2.590759	-1.964381	-0.369319	C	2.582752	-1.958330	-0.542672
C	3.777527	-1.235740	-0.509067	C	3.787001	-1.262341	-0.462747	C	3.783571	-1.259826	-0.594720
C	3.832829	0.140261	-0.334757	C	3.846254	0.116567	-0.314680	C	3.853855	0.105018	-0.351654
H	0.561201	0.678698	0.154138	H	0.573936	0.675652	0.145578	H	0.588495	0.653507	0.167058
H	4.698341	-1.771094	-0.716649	H	4.707025	-1.804782	-0.654860	H	4.698431	-1.793992	-0.829862
C	0.082636	-1.947994	0.035748	C	0.086661	-1.951162	0.075168	C	0.081460	-1.958619	-0.082464
H	0.114672	-2.868311	-0.553510	H	0.118645	-2.882780	-0.496060	H	0.103234	-2.848501	-0.717185
C	-1.091591	-1.143385	-0.504674	C	-1.081707	-1.153069	-0.487169	C	-1.084259	-1.115286	-0.580611
C	-1.165699	-0.859615	-1.874683	C	-1.147288	-0.895522	-1.862781	C	-1.155978	-0.762345	-1.934579
C	-2.112145	-0.677389	0.319651	C	-2.105098	-0.667731	0.322345	C	-2.099293	-0.680257	0.267122
C	-2.245516	-0.121559	-2.338418	C	-2.221906	-0.162815	-2.346640	C	-2.227975	0.009657	-2.359573
C	-3.201911	0.074066	-0.121164	C	-3.189742	0.078849	-0.138912	C	-3.181080	0.104083	-0.134299
H	-2.060209	-0.911498	1.369659	H	-2.059757	-0.881721	1.376942	H	-2.049367	-0.967306	1.304007
C	-3.242522	0.345233	-1.491775	C	-3.221869	0.323667	-1.514697	C	-3.219442	0.444106	-1.489536
H	-2.320014	0.096453	-3.399012	H	-2.289808	0.034955	-3.411635	H	-2.300640	0.281404	-3.407815
C	-0.075666	-2.395147	1.506633	C	-0.081299	-2.369311	1.553495	C	-0.080934	-2.477281	1.364301
H	-1.062117	-2.848624	1.616419	H	-1.069949	-2.817170	1.666519	H	-1.072183	-2.925028	1.452228
H	0.644942	-3.195043	1.690563	H	0.635432	-3.168000	1.756801	H	0.631063	-3.293127	1.507114
C	0.141243	-1.304027	2.573348	C	0.133647	-1.258576	2.600174	C	0.148311	-1.443465	2.484186
H	-0.062798	-0.318747	2.151307	H	-0.595204	-1.380182	3.404358	H	-0.576704	-1.615577	3.282593
C	1.549613	-1.310177	3.164958	H	-0.064561	-0.280920	2.158100	H	-0.045263	-0.436106	2.112321
H	1.666290	-0.524809	3.911945	C	1.538721	-1.258187	3.199604	C	1.556829	-1.494486	3.073270
H	1.756512	-2.265788	3.649656	H	1.654099	-0.458966	3.931955	H	1.682377	-0.748895	3.858596
H	2.302989	-1.154737	2.392106	H	2.296873	-1.120291	2.428094	H	1.753613	-2.475392	3.509198
C	2.588696	-3.435738	-0.632031	C	2.591023	-3.460174	-0.549896	H	2.311455	-1.308598	2.308402
H	2.139429	-3.964357	0.208561	H	2.135270	-3.970922	0.298219	C	2.570930	-3.437929	-0.826661
H	2.022780	-3.708152	-1.525231	H	2.029046	-3.747805	-1.440809	H	2.116388	-4.003184	-0.013179
H	3.604526	-3.805146	-0.756368	H	3.606195	-3.835424	-0.661450	H	2.001649	-3.659060	-1.731811
C	-0.120698	-1.329129	-2.852145	C	-0.098623	-1.387415	-2.825198	C	-0.116613	-1.193466	-2.935457
H	0.808314	-0.769371	-2.736582	H	0.831732	-0.828751	-2.715287	H	0.818463	-0.650266	-2.792928
H	0.120829	-2.382247	-2.701651	H	0.138316	-2.438265	-2.653101	H	0.113573	-2.255354	-2.838241
H	-0.470908	-1.206951	-3.875258	H	-0.442779	-1.283783	-3.852404	H	-0.465994	-1.016333	-3.950786
O	-4.286292	1.072584	-2.002327	O	-4.260222	1.044643	-2.044957	O	-4.255533	1.207377	-1.961862
H	-4.157744	1.180397	-2.947327	H	-4.126110	1.133762	-2.991131	H	-4.126300	1.361079	-2.900397
O	5.034935	0.792220	-0.421339	O	5.051134	0.762571	-0.407133	O	5.062905	0.747495	-0.406674

H	5.725834	0.151333	-0.603199	H	5.740731	0.115908	-0.572771	H	5.746734	0.109124	-0.621180
C	2.701454	2.396474	0.132513	C	2.720388	2.385275	0.102740	C	2.747208	2.347016	0.229405
C	3.581422	2.734054	1.348056	C	3.594875	2.743191	1.316419	C	3.631391	2.613665	1.459433
H	4.607009	2.404275	1.205655	H	4.620045	2.407179	1.186095	H	4.653276	2.280401	1.299594
H	3.584422	3.813470	1.509977	H	3.600838	3.825512	1.457526	H	3.646159	3.683500	1.675301
H	3.184249	2.256563	2.245338	H	3.191087	2.284474	2.220516	H	3.229522	2.096064	2.332044
C	1.301349	2.954338	0.407739	C	1.320794	2.953186	0.359388	C	1.353364	2.905439	0.533735
H	0.619784	2.774054	-0.424560	H	0.643165	2.759241	-0.473054	H	0.669469	2.774508	-0.305848
H	0.865466	2.524316	1.310854	H	0.878433	2.542174	1.268187	H	0.913305	2.435369	1.414594
H	1.373656	4.032321	0.553496	H	1.396145	4.033522	0.484746	H	1.437411	3.973907	0.733315
C	3.243719	3.098336	-1.123371	C	3.272033	3.060902	-1.163405	C	3.296410	3.105121	-0.990252
H	4.260238	2.788672	-1.350498	H	4.288671	2.743399	-1.378876	H	4.309402	2.796296	-1.233691
H	2.611599	2.875141	-1.984157	H	2.643852	2.823334	-2.023226	H	2.661478	2.932233	-1.860490
H	3.239050	4.178949	-0.969970	H	3.270377	4.144283	-1.030883	H	3.303517	4.176664	-0.782825
C	-4.285313	0.571599	0.839421	C	-4.276615	0.598563	0.805878	C	-4.258549	0.564447	0.851169
C	-5.651519	-0.012828	0.443976	C	-5.642708	0.011349	0.414189	C	-5.631202	0.015372	0.428330
H	-6.415352	0.337137	1.140856	H	-6.409098	0.377331	1.099945	H	-6.390850	0.338147	1.142688
H	-5.940614	0.285073	-0.560188	H	-5.925227	0.290824	-0.597118	H	-5.917559	0.366386	-0.559321
H	-5.624354	-1.102441	0.488130	H	-5.619672	-1.077297	0.479481	H	-5.615813	-1.075314	0.417734
C	-4.342805	2.108357	0.815133	C	-4.328491	2.134756	0.751665	C	-4.299412	2.101018	0.904072
H	-4.584663	2.485892	-0.174645	H	-4.563569	2.493965	-0.246532	H	-4.537666	2.530330	-0.065266
H	-5.105028	2.458537	1.513639	H	-5.093280	2.500980	1.439051	H	-5.057449	2.423910	1.620063
H	-3.383188	2.527522	1.121748	H	-3.369081	2.556434	1.055469	H	-3.335160	2.493894	1.230327
C	-4.001086	0.144845	2.283519	C	-4.001836	0.198762	2.259487	C	-3.978234	0.062727	2.271741
H	-3.983181	-0.940757	2.389934	H	-3.988387	-0.884642	2.386898	H	-3.972030	-1.026978	2.323538
H	-3.052888	0.544754	2.646627	H	-3.054223	0.602317	2.620081	H	-3.025580	0.433660	2.653464
H	-4.792594	0.528431	2.927596	H	-4.795491	0.597436	2.891649	H	-4.765219	0.422027	2.935078
**SW (C10•)**				**SW (C11•)**				**SW (C12•)**			
C	2.672247	0.885536	-0.060274	C	2.677594	0.919206	-0.044773	C	2.652627	0.886466	-0.069434
C	1.488291	0.153461	-0.038190	C	1.496389	0.182871	-0.017590	C	1.467677	0.157122	-0.023331
C	1.412681	-1.220155	-0.256075	C	1.425792	-1.192320	-0.227056	C	1.387330	-1.219596	-0.218779
C	2.585491	-1.917084	-0.551335	C	2.601042	-1.886660	-0.518691	C	2.555710	-1.923346	-0.515424
C	3.779125	-1.205991	-0.597910	C	3.791991	-1.171420	-0.570243	C	3.750040	-1.215385	-0.585787
C	3.834298	0.159071	-0.352179	C	3.842197	0.195311	-0.332869	C	3.810231	0.153258	-0.362147
H	0.561588	0.672508	0.155986	H	0.567856	0.699635	0.173916	H	0.544004	0.680949	0.172444
H	4.700334	-1.730152	-0.830770	H	4.715028	-1.693555	-0.800399	H	4.667799	-1.744922	-0.820101
C	0.082740	-1.944312	-0.099951	C	0.098643	-1.920464	-0.065805	C	0.057839	-1.938635	-0.037241
H	0.116033	-2.832769	-0.736168	H	0.134923	-2.812654	-0.696610	H	0.082814	-2.837036	-0.659726
C	-1.089968	-1.112293	-0.600711	C	-1.077419	-1.095892	-0.570997	C	-1.118636	-1.112313	-0.538598
C	-1.160620	-0.757672	-1.954296	C	-1.150090	-0.749798	-1.926681	C	-1.203002	-0.778855	-1.896770
C	-2.112458	-0.689417	0.244207	C	-2.101044	-0.671685	0.271877	C	-2.131364	-0.674241	0.310326
C	-2.239123	0.003867	-2.381699	C	-2.231648	0.005094	-2.358138	C	-2.284625	-0.022035	-2.324670
C	-3.200963	0.084314	-0.159636	C	-3.192636	0.095506	-0.136090	C	-3.222716	0.095160	-0.094105
H	-2.063174	-0.977804	1.280751	H	-2.050151	-0.953559	1.310134	H	-2.071608	-0.946410	1.350698
C	-3.238120	0.426369	-1.514395	C	-3.231767	0.429151	-1.492889	C	-3.273623	0.415969	-1.453607
H	-2.310946	0.276736	-3.429707	H	-2.305027	0.271297	-3.407753	H	-2.367067	0.234481	-3.376022
C	-0.079302	-2.467258	1.345310	C	-0.060703	-2.435186	1.382705	C	-0.089803	-2.438506	1.417696
H	-1.066123	-2.925485	1.428947	H	-1.045764	-2.896570	1.469665	H	-1.076491	-2.893451	1.518953
H	0.640672	-3.275884	1.489187	H	0.662357	-3.240231	1.531123	H	0.630223	-3.246166	1.566652
C	0.135204	-1.433133	2.467827	C	0.150521	-1.393442	2.498780	C	0.138418	-1.387298	2.521484
H	-0.590771	-1.614247	3.263366	H	-0.574362	-1.572406	3.295800	H	-0.579403	-1.554490	3.327402
H	-0.067575	-0.427178	2.097068	H	-0.056200	-0.390530	2.122004	H	-0.066498	-0.386898	2.137118
C	1.542103	-1.470515	3.061783	C	1.557856	-1.421957	3.092198	C	1.551465	-1.418073	3.101052
H	1.657101	-0.725076	3.848867	H	1.670478	-0.671308	3.874660	H	1.676118	-0.660590	3.875058
H	1.747590	-2.450100	3.496663	H	1.767220	-2.398099	3.532931	H	1.759815	-2.391104	3.549085
H	2.297425	-1.275377	2.299912	H	2.312052	-1.228655	2.328745	H	2.299014	-1.236386	2.328266
C	2.590119	-3.396209	-0.837985	C	2.611041	-3.367479	-0.796321	C	2.554681	-3.406797	-0.778815
H	2.138655	-3.967648	-0.027114	H	2.162130	-3.935641	0.018262	H	2.110825	-3.964556	0.045667

H	2.026357	-3.621632	-1.745528	H	2.047656	-3.600533	-1.702169	H	1.980933	-3.645351	-1.676692
H	3.606172	-3.759009	-0.978854	H	3.628368	-3.727341	-0.935521	H	3.568540	-3.773731	-0.924692
C	-0.113305	-1.176109	-2.952265	C	-0.101735	-1.170408	-2.922643	C	-0.167065	-1.214941	-2.899050
H	0.815541	-0.623438	-2.805477	H	0.825118	-0.613393	-2.779727	H	0.764262	-0.661832	-2.770795
H	0.127617	-2.235710	-2.856120	H	0.143187	-2.228497	-2.820169	H	0.072986	-2.273366	-2.788807
O	-4.280463	1.179638	-1.989019	H	-0.450559	-1.002593	-3.939745	H	-0.525153	-1.054935	-3.914188
H	-4.149551	1.336378	-2.926818	O	-4.277157	1.175620	-1.971537	O	-4.319603	1.163708	-1.928973
O	5.036758	0.814231	-0.401797	H	-4.147313	1.327124	-2.910345	H	-4.198360	1.305448	-2.870452
H	5.727965	0.183421	-0.615021	O	5.042178	0.854634	-0.387127	O	5.013255	0.805227	-0.434766
C	2.702265	2.388341	0.228946	H	5.735624	0.225117	-0.596873	H	5.701046	0.169810	-0.645339
C	3.579294	2.661978	1.462555	C	2.702154	2.423849	0.235262	C	2.688369	2.393567	0.195787
H	4.605158	2.339689	1.305730	C	3.578791	2.708272	1.466708	C	3.578906	2.684844	1.415602
H	3.582131	3.731518	1.680370	H	4.605770	2.388858	1.311300	H	4.602478	2.358145	1.253031
H	3.179795	2.138633	2.332824	H	3.577750	3.779123	1.677996	H	3.585944	3.757664	1.616531
C	1.301606	2.931633	0.529348	H	3.181696	2.188758	2.340367	H	3.187727	2.176004	2.298167
H	0.622064	2.795090	-0.312872	C	1.299633	2.963738	0.533099	C	1.291936	2.944221	0.502397
H	0.863393	2.455403	1.407815	H	0.620172	2.819529	-0.307907	H	0.603266	2.795786	-0.330343
H	1.373788	4.000557	0.731117	H	0.863652	2.491244	1.414689	H	0.862215	2.482695	1.392825
C	3.247793	3.154342	-0.987421	C	3.244194	3.184445	-0.986046	H	1.368145	4.016040	0.686559
H	4.264804	2.856548	-1.227833	H	4.262185	2.888984	-1.225185	C	3.222330	3.139314	-1.038172
H	2.617759	2.976411	-1.860198	H	2.614379	2.998847	-1.857382	H	4.236207	2.835812	-1.284608
H	3.242982	4.225523	-0.778071	H	3.235494	4.256858	-0.783228	H	2.582767	2.948919	-1.901335
C	-4.286632	0.531624	0.822841	C	-4.279460	0.544749	0.844225	H	3.221634	4.213658	-0.845651
C	-5.651983	-0.030983	0.394191	C	-5.642923	-0.025547	0.419747	C	-4.297117	0.559944	0.892637
H	-6.417462	0.282553	1.106437	H	-6.409200	0.289471	1.130480	C	-5.667929	-0.006679	0.487340
H	-5.938524	0.318809	-0.593840	H	-5.931276	0.317142	-0.570244	H	-6.425260	0.319485	1.202619
H	-5.625167	-1.121428	0.381717	H	-5.612046	-1.115941	0.413907	H	-5.964306	0.328108	-0.502984
C	-4.343725	2.067586	0.878322	C	-4.342255	2.080798	0.890371	H	-5.643178	-1.097235	0.491731
H	-4.583048	2.496141	-0.091088	H	-4.583674	2.502537	-0.081505	C	-4.350901	2.096696	0.924561
H	-5.107600	2.381252	1.592206	H	-5.106927	2.395962	1.602740	H	-4.599725	2.510438	-0.048884
H	-3.384779	2.469914	1.208671	H	-3.384647	2.488704	1.217745	H	-5.106610	2.423013	1.641458
C	-4.006076	0.030288	2.243501	C	-3.996304	0.053136	2.267764	H	-3.387796	2.502304	1.238389
H	-3.988674	-1.059385	2.293388	H	-3.974811	-1.036140	2.324285	C	-4.002409	0.080437	2.317959
H	-3.058693	0.410453	2.629238	H	-3.050147	0.439175	2.650666	H	-3.986408	-1.008349	2.384787
H	-4.799093	0.380153	2.904694	H	-4.790280	0.404069	2.927238	H	-3.050319	0.464777	2.687636

SET-PT (SET step)

M06-2X/6-311++G(2d,2p)			
SW $^{•+}$			
C	2.674697	0.888098	-0.065330
C	1.490516	0.157178	-0.024758
C	1.414171	-1.221119	-0.210442
C	2.586386	-1.925184	-0.490759
C	3.780208	-1.215816	-0.555338
C	3.836146	0.154587	-0.341592
H	0.564226	0.680960	0.158360
H	4.700955	-1.745611	-0.777004
C	0.084170	-1.940941	-0.035877
H	0.116392	-2.844013	-0.651229
C	-1.088853	-1.120396	-0.554514
C	-1.161009	-0.797428	-1.915920
C	-2.110181	-0.677540	0.281526
C	-2.239763	-0.045668	-2.359688
C	-3.198904	0.086963	-0.138961
H	-2.059751	-0.941681	1.324458
C	-3.237571	0.397328	-1.501287

H	-2.312751	0.202701	-3.413692
C	-0.076316	-2.429962	1.421385
H	-1.063193	-2.885745	1.516869
H	0.643552	-3.235279	1.583232
C	0.139895	-1.370007	2.519210
H	-0.585185	-1.532239	3.319625
H	-0.062983	-0.372901	2.125322
C	1.547494	-1.394044	3.112198
H	1.663695	-0.630486	3.881536
H	1.753166	-2.363292	3.569571
H	2.301967	-1.217017	2.345080
C	2.590160	-3.410597	-0.742824
H	2.139474	-3.962792	0.081698
H	2.025230	-3.656924	-1.644186
H	3.605918	-3.776963	-0.876401
C	-0.115038	-1.239427	-2.905103
H	0.814174	-0.683827	-2.772359
H	0.125634	-2.296586	-2.784547
H	-0.463847	-1.087744	-3.924740
O	-4.280224	1.139705	-1.992114
H	-4.150385	1.274472	-2.933471
O	5.038771	0.807963	-0.407918
H	5.729504	0.172093	-0.607190
C	2.705580	2.397231	0.188709
C	3.584185	2.699251	1.414550
H	4.609749	2.373008	1.264064
H	3.587652	3.773580	1.607346
H	3.185552	2.196503	2.297270
C	1.305470	2.947906	0.478024
H	0.624870	2.792002	-0.359969
H	0.868149	2.492466	1.367886
H	1.378263	4.021220	0.654710
C	3.249910	3.134439	-1.045850
H	4.266528	2.830738	-1.280462
H	2.618766	2.936426	-1.913485
H	3.245719	4.210215	-0.861546
C	-4.283237	0.557479	0.834106
C	-5.649296	-0.014468	0.420330
H	-6.413811	0.315886	1.125979
H	-5.936904	0.312282	-0.575251
H	-5.622871	-1.104917	0.433271
C	-4.339735	2.094339	0.853801
H	-4.580075	2.500247	-0.125058
H	-5.102643	2.424861	1.561083
H	-3.380254	2.503908	1.173528
C	-4.001147	0.089323	2.265741
H	-3.984060	-0.998895	2.341020
H	-3.053170	0.478032	2.641372
H	-4.793248	0.454816	2.919537

SET-PT (PT step) mechanism

M06-2X/6-311++G(2d,2p)											
SW $^{\bullet+}$				SW (O1$^{\bullet}$)				SW (O2$^{\bullet}$)			
C	2.674697	0.888098	-0.065330	C	2.526451	0.930440	0.047402	C	2.565015	0.942474	0.051479
C	1.490516	0.157178	-0.024758	C	1.394362	0.133490	0.106632	C	1.439502	0.135834	0.104544
C	1.414171	-1.221119	-0.210442	C	1.378075	-1.233816	-0.199626	C	1.429827	-1.224130	-0.233078

C	2.586386	-1.925184	-0.490759	C	2.565262	-1.863023	-0.602506	C	2.617062	-1.834887	-0.663281
C	3.780208	-1.215816	-0.555338	C	3.709502	-1.089314	-0.665590	C	3.754853	-1.051301	-0.720870
C	3.836146	0.154587	-0.341592	C	3.705406	0.274842	-0.358790	C	3.744176	0.305347	-0.382558
H	0.564226	0.680960	0.158360	H	0.466671	0.585513	0.425255	H	0.512106	0.573365	0.443601
H	4.700955	-1.745611	-0.777004	H	4.646404	-1.548921	-0.960268	H	4.691779	-1.496896	-1.036263
C	0.084170	-1.940941	-0.035877	C	0.078556	-2.007608	-0.088199	C	0.137305	-2.010087	-0.125478
H	0.116392	-2.844013	-0.651229	H	0.149119	-2.847237	-0.790007	H	0.206252	-2.832751	-0.847253
C	-1.088853	-1.120396	-0.554514	C	-1.024728	-1.137174	-0.618334	C	-0.978092	-1.136024	-0.623313
C	-1.161009	-0.797428	-1.915920	C	-0.966675	-0.661916	-1.978459	C	-0.938523	-0.629103	-1.972626
C	-2.110181	-0.677540	0.281526	C	-2.105557	-0.749392	0.195713	C	-2.052677	-0.775297	0.211182
C	-2.239763	-0.045668	-2.359688	C	-1.970892	0.162692	-2.409952	C	-1.953438	0.197621	-2.373988
C	-3.198904	0.086963	-0.138961	C	-3.118126	0.090519	-0.207713	C	-3.075745	0.066015	-0.161677
H	-2.059751	-0.941681	1.324458	H	-2.134022	-1.134354	1.200294	H	-2.067243	-1.183527	1.206836
C	-3.237571	0.397328	-1.501287	C	-3.020432	0.559991	-1.563905	C	-2.996454	0.567361	-1.507623
H	-2.312751	0.202701	-3.413692	H	-1.968380	0.525821	-3.431104	H	-1.964847	0.584210	-3.386430
C	-0.076316	-2.429962	1.421385	C	-0.159486	-2.624934	1.304291	C	-0.080842	-2.661140	1.254857
H	-1.063193	-2.885745	1.516869	H	-1.157074	-3.068290	1.308261	H	-1.075060	-3.111998	1.259402
H	0.643552	-3.235279	1.583232	H	0.532666	-3.461500	1.413577	H	0.618568	-3.494748	1.337230
C	0.139895	-1.370007	2.519210	C	0.031811	-1.683691	2.507185	C	0.116834	-1.746467	2.477062
H	-0.585185	-1.532239	3.319625	H	-0.663878	-1.981966	3.293241	H	-0.567941	-2.068039	3.263515
H	-0.062983	-0.372901	2.125322	H	-0.233881	-0.658007	2.240373	H	-0.159273	-0.716941	2.236958
C	1.547494	-1.394044	3.112198	C	1.450441	-1.702367	3.073664	C	1.541727	-1.767477	3.027513
H	1.663695	-0.630486	3.881536	H	1.535750	-1.029978	3.926200	H	1.631522	-1.114300	3.894405
H	1.753166	-2.363292	3.569571	H	1.712528	-2.704847	3.412865	H	1.814858	-2.775502	3.340559
H	2.301967	-1.217017	2.345080	H	2.185244	-1.394758	2.329557	H	2.266008	-1.437245	2.282787
C	2.590160	-3.410597	-0.742824	C	2.623043	-3.325250	-0.951471	C	2.681671	-3.288200	-1.046598
H	2.139474	-3.962792	0.081698	H	2.181954	-3.941136	-0.167919	H	2.253765	-3.925307	-0.272763
H	2.025230	-3.656924	-1.644186	H	2.086505	-3.538415	-1.878300	H	2.136510	-3.483985	-1.972228
H	3.605918	-3.776963	-0.876401	H	3.651986	-3.647620	-1.089148	H	3.711359	-3.599522	-1.202866
C	-0.115038	-1.239427	-2.905103	C	0.129252	-1.035526	-2.933937	C	0.149495	-0.972286	-2.948342
H	0.814174	-0.683827	-2.772359	H	0.986572	-0.372672	-2.806075	H	1.003308	-0.306095	-2.814494
H	0.125634	-2.296586	-2.784547	H	0.479716	-2.053012	-2.775404	H	0.509111	-1.990478	-2.817213
H	-0.463847	-1.087744	-3.924740	H	-0.222939	-0.947201	-3.958877	H	-0.214619	-0.863014	-3.967077
O	-4.280224	1.139705	-1.992114	O	-3.959427	1.378075	-2.008213	O	-3.946246	1.388351	-1.922655
H	-4.150385	1.274472	-2.933471	O	4.848752	0.986434	-0.440016	H	-3.803635	1.664608	-2.836534
O	5.038771	0.807963	-0.407918	H	5.580387	0.421266	-0.705084	O	4.881327	1.027245	-0.459708
H	5.729504	0.172093	-0.607190	C	2.501463	2.414484	0.417878	C	2.533276	2.417349	0.456448
C	2.705580	2.397231	0.188709	C	3.445918	2.674818	1.603831	C	3.488831	2.657397	1.637779
C	3.584185	2.699251	1.414550	H	4.478487	2.443671	1.358846	H	4.520295	2.439772	1.376309
H	4.609749	2.373008	1.264064	H	3.391036	3.726636	1.886365	H	3.429386	3.701975	1.945157
H	3.587652	3.773580	1.607346	H	3.149868	2.076564	2.466776	H	3.206699	2.037182	2.489814
H	3.185552	2.196503	2.297270	C	1.096890	2.859074	0.838667	C	1.130219	2.841487	0.902645
C	1.305470	2.947906	0.478024	H	0.368817	2.726084	0.035737	H	0.394323	2.721547	0.104808
H	0.624870	2.792002	-0.359969	H	0.750029	2.321426	1.723213	H	0.797077	2.280978	1.778227
H	0.868149	2.492466	1.367886	H	1.123096	3.919201	1.087473	H	1.151423	3.895763	1.175617
H	1.378263	4.021220	0.654710	C	2.920139	3.265339	-0.793148	C	2.932336	3.299043	-0.739040
C	3.249910	3.134439	-1.045850	H	3.930765	3.037449	-1.119408	H	3.940939	3.086383	-1.081445
H	4.266528	2.830738	-1.280462	H	2.238229	3.102633	-1.629335	H	2.242448	3.150513	-1.571312
H	2.618766	2.936426	-1.913485	H	2.879533	4.321555	-0.524955	H	2.886975	4.348457	-0.446052
H	3.245719	4.210215	-0.861546	C	-4.262015	0.500872	0.708522	C	-4.212423	0.446472	0.776180
C	-4.283237	0.557479	0.834106	C	-5.607041	0.050630	0.103946	C	-5.560712	0.000136	0.175992
C	-5.649296	-0.014468	0.420330	H	-6.405066	0.302588	0.801916	H	-6.352807	0.229894	0.888232
H	-6.413811	0.315886	1.125979	H	-5.821448	0.539622	-0.841627	H	-5.789106	0.509169	-0.755628
H	-5.936904	0.312282	-0.575251	H	-5.620653	-1.029201	-0.046118	H	-5.568090	-1.076019	0.001136
H	-5.622871	-1.104917	0.433271	C	-4.247870	2.030242	0.903678	C	-4.207299	1.970998	1.006510
C	-4.339735	2.094339	0.853801	H	-4.420912	2.567005	-0.024489	H	-4.394489	2.527698	0.092947
H	-4.580075	2.500247	-0.125058	H	-5.039776	2.299688	1.602127	H	-4.993398	2.218273	1.719567
H	-5.102643	2.424861	1.561083	H	-3.298335	2.356883	1.329397	H	-3.255536	2.294904	1.429334
H	-3.380254	2.503908	1.173528	C	-4.125210	-0.151407	2.087590	C	-4.055647	-0.236381	2.138216

C	-4.001147	0.089323	2.265741	H	-4.157761	-1.239990	2.027918	H	-4.080901	-1.323514	2.053727
H	-3.984060	-0.998895	2.341020	H	-3.206148	0.149175	2.592756	H	-3.133289	0.059410	2.640190
H	-3.053170	0.478032	2.641372	H	-4.960935	0.168156	2.707590	H	-4.886793	0.062473	2.774487
H	-4.793248	0.454816	2.919537								
**SW (C1·)**				**SW (C2·)**				**SW (C3·)**			
C	2.542266	0.937724	0.068170	C	2.568174	0.911519	0.058160	C	2.541399	0.919015	0.072254
C	1.415002	0.133153	0.115122	C	1.436125	0.113609	0.103629	C	1.413630	0.114663	0.109893
C	1.403710	-1.225708	-0.226861	C	1.415162	-1.242714	-0.247843	C	1.401371	-1.240006	-0.248307
C	2.591088	-1.837447	-0.655269	C	2.597154	-1.858736	-0.684925	C	2.588242	-1.847340	-0.684322
C	3.730598	-1.055939	-0.706779	C	3.741289	-1.083931	-0.735158	C	3.728242	-1.066003	-0.726797
C	3.721530	0.299645	-0.364181	C	3.741874	0.269236	-0.383019	C	3.720138	0.285394	-0.368016
H	4.667634	-1.502385	-1.020640	H	0.512502	0.555224	0.447654	H	0.486406	0.549479	0.452889
C	0.109306	-2.009439	-0.125835	C	0.116305	-2.019135	-0.147538	H	4.664906	-1.509265	-1.046229
H	0.178907	-2.829939	-0.850007	H	0.178173	-2.834927	-0.877706	C	0.106491	-2.024059	-0.156303
C	-1.002789	-1.131588	-0.624390	C	-0.992143	-1.130948	-0.635801	C	-1.005171	-1.139598	-0.644025
C	-0.957965	-0.620458	-1.971950	C	-0.949077	-0.610624	-1.979895	C	-0.960376	-0.612425	-1.985391
C	-2.079286	-0.771388	0.207864	C	-2.063340	-0.769983	0.202936	C	-2.081215	-0.788683	0.192769
C	-1.969976	0.209549	-2.373867	C	-1.957387	0.228424	-2.372230	C	-1.971959	0.222977	-2.377081
C	-3.099512	0.073133	-0.165531	C	-3.079670	0.083427	-0.160744	C	-3.100996	0.060895	-0.170224
H	-2.097797	-1.182752	1.202160	H	-2.080766	-1.188219	1.194382	H	-2.099727	-1.211892	1.182082
C	-3.014987	0.578598	-1.509616	C	-2.996927	0.597817	-1.501550	C	-3.016503	0.582337	-1.508194
H	-1.977429	0.599377	-3.385104	H	-1.966120	0.625404	-3.380669	H	-1.979429	0.624870	-3.383583
C	-0.114476	-2.664446	1.251725	C	-0.106497	-2.682437	1.226207	C	-0.117348	-2.695343	1.213389
H	-1.109591	-3.113344	1.251704	H	-1.104368	-3.125174	1.226713	H	-1.112751	-3.143571	1.208273
H	0.583024	-3.499696	1.333643	H	0.586109	-3.522532	1.299678	H	0.579637	-3.531960	1.285125
C	0.081148	-1.754058	2.477455	C	0.099243	-1.781930	2.457567	C	0.079188	-1.799798	2.449861
H	-0.606737	-2.076771	3.260720	H	-0.587762	-2.105903	3.241085	H	-0.608697	-2.131412	3.229400
H	-0.192169	-0.723228	2.239765	H	-0.168548	-0.747784	2.228134	H	-0.193528	-0.766026	2.224585
C	1.504254	-1.779643	3.032319	C	1.524180	-1.820205	3.006972	C	1.502424	-1.832927	3.003990
H	1.592602	-1.129406	3.901565	H	1.619733	-1.176665	3.880430	H	1.591422	-1.193184	3.880924
H	1.774407	-2.789199	3.343008	H	1.789206	-2.833572	3.309564	H	1.772010	-2.846297	3.302516
H	2.231529	-1.448481	2.290931	H	2.250780	-1.488323	2.265244	H	2.229716	-1.493391	2.266416
C	2.654038	-3.289658	-1.043010	C	2.649686	-3.308540	-1.083083	C	2.650154	-3.294852	-1.089412
H	2.222439	-3.928373	-0.272559	H	2.216955	-3.949992	-0.315546	H	2.218348	-3.942456	-0.326534
H	2.111415	-3.481418	-1.970971	H	2.102498	-3.490406	-2.010355	H	2.107161	-3.475157	-2.019451
H	3.683599	-3.602523	-1.197027	H	3.676719	-3.626659	-1.243100	H	3.679472	-3.606513	-1.247440
C	0.132446	-0.962692	-2.945325	C	0.135628	-0.952714	-2.959674	C	0.129556	-0.943696	-2.963085
H	0.987145	-0.298624	-2.806668	H	0.994926	-0.294927	-2.819519	H	0.984719	-0.281881	-2.816733
H	0.489638	-1.982008	-2.816310	H	0.486965	-1.975098	-2.839142	H	0.486126	-1.964710	-2.846362
H	-0.228237	-0.849460	-3.964848	H	-0.228070	-0.830079	-3.977037	H	-0.231325	-0.818053	-3.981083
O	-3.961845	1.402786	-1.925017	O	-3.940170	1.430738	-1.907650	O	-3.962940	1.412039	-1.913455
H	-3.815808	1.681665	-2.837558	H	-3.795744	1.715132	-2.818743	H	-3.816966	1.701713	-2.822636
O	4.860341	1.019528	-0.435451	O	4.884855	0.982562	-0.453443	O	4.859392	1.005349	-0.430987
H	5.592922	0.465630	-0.720913	H	5.612941	0.426179	-0.745494	H	5.591541	0.454434	-0.723249
C	2.512158	2.411364	0.477735	C	2.548695	2.382394	0.478171	C	2.512349	2.387673	0.499414
C	3.464455	2.645760	1.662827	C	3.506751	2.602555	1.661350	C	3.465111	2.607274	1.686963
H	4.496308	2.426923	1.403913	H	4.536274	2.379184	1.397092	H	4.496754	2.390887	1.425170
H	3.406102	3.689471	1.973343	H	3.456001	3.644392	1.979401	H	3.407512	3.647236	2.009950
H	3.178415	2.023399	2.511989	H	3.219964	1.976004	2.507173	H	3.178896	1.974990	2.528703
C	1.108541	2.836862	0.920863	C	1.149370	2.813414	0.929462	C	1.109124	2.808742	0.947980
H	0.374929	2.720920	0.120335	H	0.412134	2.707643	0.130859	H	0.375225	2.702848	0.146324
H	0.771534	2.274232	1.793602	H	0.812075	2.246740	1.799469	H	0.771986	2.235936	1.814023
H	1.130964	3.890220	1.197258	H	1.179330	3.864640	1.213163	H	1.132298	3.858706	1.236942
C	2.916726	3.296064	-0.713675	C	2.954381	3.272952	-0.708486	C	2.917170	3.286292	-0.681446
H	3.925982	3.082494	-1.053580	H	3.961045	3.055558	-1.053611	H	3.926198	3.076154	-1.024155
H	2.229174	3.151549	-1.548584	H	2.262900	3.138567	-1.541840	H	2.229303	3.152209	-1.517834
H	2.872511	4.344628	-0.417489	H	2.917747	4.319657	-0.404782	H	2.873709	4.331269	-0.372733
C	-4.238389	0.452858	0.769951	C	-4.212746	0.463573	0.781586	C	-4.239380	0.430139	0.770041
C	-5.585657	0.011107	0.164103	C	-5.564930	0.034424	0.177637	C	-5.587093	-0.003474	0.159324

H	-6.379539	0.240168	0.874575	H	-6.354776	0.263369	0.892631	H	-6.380639	0.217586	0.872700
H	-5.810107	0.523552	-0.766604	H	-5.789600	0.554787	-0.748612	H	-5.811460	0.520203	-0.765130
H	-5.594606	-1.064470	-0.014203	H	-5.581196	-1.039795	-0.008183	H	-5.596782	-1.076837	-0.031826
C	-4.230985	1.976631	1.005153	C	-4.195040	1.985576	1.027453	C	-4.230932	1.950986	1.023439
H	-4.414194	2.536603	0.092784	H	-4.378108	2.553082	0.119722	H	-4.414023	2.521943	0.117880
H	-5.018839	2.223195	1.716517	H	-4.978747	2.231980	1.743439	H	-5.018439	2.189533	1.737915
H	-3.279923	2.297302	1.432001	H	-3.240456	2.297358	1.453022	H	-3.279551	2.265923	1.453830
C	-4.087258	-0.234633	2.130291	C	-4.060908	-0.234404	2.136497	C	-4.088330	-0.273662	2.122024
H	-4.114389	-1.321439	2.042259	H	-4.095095	-1.320374	2.040937	H	-4.116185	-1.359320	2.021014
H	-3.165905	0.057731	2.636106	H	-3.135919	0.048699	2.640942	H	-3.166655	0.012044	2.631046
H	-4.919815	0.063843	2.764892	H	-4.889275	0.064727	2.776252	H	-4.920527	0.017741	2.760375
**SW (C4•)**				**SW (C5•)**				**SW (C6•)**			
C	2.528308	0.934577	0.058373	C	2.531348	0.935434	0.050863	C	2.532266	0.928138	0.047637
C	1.402632	0.128392	0.114784	C	1.402903	0.132763	0.101780	C	1.407187	0.121921	0.114535
C	1.393179	-1.233499	-0.214985	C	1.391556	-1.229923	-0.224630	C	1.399091	-1.244505	-0.195944
C	2.580838	-1.846875	-0.640265	C	2.580176	-1.847918	-0.640434	C	2.587588	-1.862873	-0.611545
C	3.718794	-1.063771	-0.701052	C	3.720860	-1.068374	-0.695586	C	3.724968	-1.079786	-0.682482
C	3.707894	0.294811	-0.370597	C	3.711840	0.291065	-0.368619	C	3.712713	0.283324	-0.371287
H	0.474895	0.567986	0.450223	H	0.474276	0.575973	0.429915	H	0.478825	0.565464	0.442972
H	4.656031	-1.511293	-1.012771	H	4.658806	-1.519481	-0.999920	H	4.662818	-1.530914	-0.987082
C	0.100432	-2.018666	-0.104355	C	0.095747	-2.010941	-0.120664	C	0.106881	-2.029075	-0.075265
H	0.170118	-2.845496	-0.821283	H	0.167726	-2.839784	-0.835036	H	0.177815	-2.865906	-0.780369
C	-1.014272	-1.147362	-0.608535	C	-1.012947	-1.137585	-0.634455	C	-1.008104	-1.165892	-0.592620
C	-0.973064	-0.648251	-1.960708	C	-0.961225	-0.642019	-1.987570	C	-0.966186	-0.685923	-1.951684
C	-2.089785	-0.781690	0.222606	C	-2.092874	-0.766602	0.188568	C	-2.084589	-0.789371	0.232395
C	-1.987405	0.176262	-2.368025	C	-1.970345	0.184490	-2.403715	C	-1.980850	0.131925	-2.371443
C	-3.112311	0.057576	-0.156303	C	-3.110324	0.074756	-0.199260	C	-3.107473	0.043624	-0.159173
C	-3.031385	0.551126	-1.505030	H	-2.116516	-1.166498	1.187425	H	-2.100866	-1.177695	1.235953
H	-1.997583	0.556994	-3.382697	C	-3.018924	0.564652	-1.548650	C	-3.025835	0.518113	-1.514681
C	-0.119408	-2.661706	1.279463	C	-0.135246	-2.649823	1.263263	H	-1.990500	0.498259	-3.391407
H	-1.113687	-3.112412	1.285452	H	-1.130894	-3.097538	1.263745	C	-0.113580	-2.652656	1.317332
H	0.579800	-3.494908	1.367469	H	0.560852	-3.484885	1.358023	H	0.586220	-3.483963	1.417680
C	0.076957	-1.740009	2.496592	C	0.055769	-1.725644	2.479362	C	0.081051	-1.713677	2.521461
H	-0.608776	-2.056950	3.284090	H	-0.636150	-2.038543	3.263057	H	-0.605075	-2.020002	3.312808
H	-0.198739	-0.711855	2.250221	H	-0.215188	-0.697296	2.228579	H	-0.195263	-0.689333	2.260348
C	1.501204	-1.758009	3.048820	C	1.476241	-1.746505	3.041128	C	1.504858	-1.722711	3.075040
H	1.590070	-1.099844	3.912027	H	1.561327	-1.086429	3.903256	H	1.592491	-1.052331	3.928922
H	1.773842	-2.764241	3.368004	H	1.743720	-2.752737	3.364646	H	1.778038	-2.724107	3.408645
H	2.226395	-1.432172	2.303044	H	2.207364	-1.424722	2.299394	H	2.230401	-1.406868	2.325316
C	2.645710	-3.302385	-1.015102	C	2.643171	-3.304558	-1.011180	C	2.653928	-3.323487	-0.965704
H	2.216821	-3.934959	-0.238098	H	2.207212	-3.933882	-0.235473	H	2.224908	-3.945354	-0.180177
H	2.101606	-3.503451	-1.940222	H	2.104645	-3.506334	-1.939404	H	2.110744	-3.538058	-1.888327
H	3.675541	-3.614729	-1.168366	H	3.673058	-3.620364	-1.156783	H	3.684134	-3.637133	-1.113701
C	0.116048	-0.997196	-2.933152	C	0.133294	-0.996673	-2.951846	C	0.124002	-1.047706	-2.918214
H	0.969789	-0.330345	-2.802159	H	0.988142	-0.332048	-2.816829	H	0.977103	-0.378379	-2.795976
H	0.475382	-2.014657	-2.795722	H	0.488644	-2.014853	-2.809470	H	0.484035	-2.062831	-2.766115
H	-0.246863	-0.893770	-3.952927	H	-0.222497	-0.894740	-3.974276	H	-0.238155	-0.959008	-3.939643
O	-3.980588	1.369819	-1.925908	O	-3.962835	1.385112	-1.977905	O	-3.975346	1.330002	-1.947865
H	-3.836876	1.640773	-2.841201	H	-3.812210	1.653322	-2.892895	H	-3.831100	1.588099	-2.866782
O	4.845225	1.016115	-0.450590	O	4.851842	1.008762	-0.442826	O	4.849534	1.004345	-0.460555
H	5.578264	0.461025	-0.732539	H	5.585073	0.450773	-0.718483	H	5.583246	0.445916	-0.734031
C	2.496282	2.411771	0.454775	C	2.501120	2.413715	0.443339	C	2.498736	2.410765	0.423079
C	3.450489	2.658528	1.635815	C	3.448168	2.660602	1.630099	C	3.451776	2.674974	1.601281
H	4.482230	2.439272	1.376812	H	4.480949	2.437610	1.378539	H	4.483904	2.452912	1.346240
H	3.390817	3.704872	1.937081	H	3.389634	3.707878	1.928337	H	3.391023	3.725426	1.887669
H	3.167284	2.043281	2.491089	H	3.157419	2.048367	2.485003	H	3.168361	2.071657	2.464943
C	1.092760	2.838649	0.896875	C	1.095970	2.845901	0.874988	C	1.094511	2.842717	0.857961
H	0.357780	2.714189	0.098885	H	0.365956	2.721622	0.072425	H	0.360285	2.706388	0.061217

H	0.758525	2.283248	1.775292	H	0.754218	2.293723	1.752544	H	0.759999	2.299526	1.743875
H	1.113778	3.894482	1.163775	H	1.118382	3.902337	1.139375	H	1.114469	3.902236	1.109916
C	2.896849	3.286495	-0.745321	C	2.912307	3.284203	-0.756246	C	2.899589	3.268751	-0.788946
H	3.905824	3.071739	-1.085313	H	3.922876	3.065574	-1.088956	H	3.909013	3.050024	-1.125050
H	2.207914	3.133240	-1.577526	H	2.228478	3.130905	-1.592644	H	2.211459	3.103183	-1.619457
H	2.851279	4.337590	-0.458455	H	2.867984	4.336151	-0.472326	H	2.852945	4.323761	-0.517013
C	-4.250034	0.443583	0.778011	C	-4.253086	0.466521	0.726468	C	-4.246270	0.441886	0.768666
C	-5.597678	-0.006053	0.178839	C	-5.598051	0.019402	0.119442	C	-5.593064	-0.017270	0.174824
H	-6.390575	0.227911	0.888814	H	-6.394956	0.257528	0.823520	H	-6.386728	0.226070	0.880777
H	-5.824921	0.497615	-0.755973	H	-5.817540	0.521383	-0.818125	H	-5.819941	0.472940	-0.767202
H	-5.604988	-1.083199	0.010196	H	-5.607473	-1.058140	-0.046545	H	-5.599375	-1.096700	0.021428
C	-4.244987	1.969413	0.999534	C	-4.244930	1.992884	0.944194	C	-4.242622	1.970700	0.968588
H	-4.431039	2.520848	0.082552	H	-4.423207	2.542553	0.024611	H	-4.428363	2.508959	0.043747
H	-5.031886	2.220899	1.710232	H	-5.035791	2.248515	1.648991	H	-5.030304	2.231576	1.675021
H	-3.293677	2.295643	1.421591	H	-3.295479	2.317334	1.371772	H	-3.291919	2.303637	1.386757
C	-4.094935	-0.231407	2.144153	C	-4.109129	-0.205476	2.095303	C	-4.091753	-0.213588	2.144344
H	-4.120228	-1.319007	2.065918	H	-4.137168	-1.293190	2.019627	H	-4.116114	-1.302206	2.081481
H	-3.173125	0.067169	2.645486	H	-3.189789	0.091610	2.602028	H	-3.170594	0.092795	2.642149
H	-4.926785	0.071218	2.777715	H	-4.944271	0.101234	2.722537	H	-4.924364	0.097295	2.772889
**SW (C7$^{\bullet}$)**				**SW (C8$^{\bullet}$)**				**SW (C9$^{\bullet}$)**			
C	2.536401	0.929324	0.044020	C	2.654493	0.873046	-0.202794	C	2.567968	0.887301	0.072455
C	1.409869	0.126123	0.121853	C	1.478971	0.143654	-0.041704	C	1.431835	0.094568	0.104682
C	1.399146	-1.244228	-0.170733	C	1.418210	-1.247873	-0.017154	C	1.404022	-1.255715	-0.268876
C	2.586348	-1.870163	-0.578637	C	2.594015	-1.972752	-0.202156	C	2.582964	-1.870574	-0.715723
C	3.725137	-1.090166	-0.660211	C	3.778776	-1.265150	-0.378919	C	3.731066	-1.100928	-0.753089
C	3.715503	0.276917	-0.366858	C	3.817693	0.122364	-0.370391	C	3.738484	0.246293	-0.378919
H	0.482448	0.575631	0.444794	H	0.549254	0.676212	0.085099	H	0.510391	0.535240	0.455690
H	4.662040	-1.546962	-0.959220	H	4.705610	-1.809477	-0.528503	H	4.661935	-1.550414	-1.080661
C	0.105544	-2.024773	-0.039346	C	0.102269	-1.940677	0.308037	C	0.101178	-2.027002	-0.181500
H	0.174673	-2.870880	-0.733474	H	0.133633	-2.938286	-0.137346	H	0.159068	-2.831079	-0.924869
C	-1.008051	-1.166376	-0.567545	C	-1.093128	-1.238867	-0.323183	C	-1.002572	-1.125297	-0.655419
C	-0.965768	-0.704294	-1.932782	C	-1.222818	-1.225592	-1.714468	C	-0.956472	-0.583342	-1.990837
C	-2.083529	-0.777121	0.252861	C	-2.079698	-0.609626	0.433806	C	-2.072136	-0.772578	0.188890
C	-1.979088	0.109863	-2.362836	C	-2.318023	-0.574681	-2.268850	C	-1.960363	0.267152	-2.369623
C	-3.105031	0.052565	-0.149201	C	-3.179729	0.065793	-0.104725	C	-3.083983	0.091849	-0.161012
H	-2.100138	-1.152262	1.261416	H	-1.994288	-0.647136	1.504525	H	-2.091978	-1.206838	1.173376
C	-3.023037	0.509142	-1.510825	C	-3.274789	0.062681	-1.489619	C	-2.998237	0.627615	-1.493232
H	-1.988453	0.462850	-3.387499	H	-2.436399	-0.564167	-3.347488	H	-1.966785	0.680571	-3.371454
C	-0.115532	-2.629688	1.361363	C	-0.017816	-2.170625	1.832976	C	-0.125396	-2.711476	1.181197
H	-1.110279	-3.079048	1.379177	H	-1.016909	-2.557542	2.039189	H	-1.125521	-3.149042	1.174288
H	0.582778	-3.460899	1.472322	H	0.676626	-2.966525	2.110765	H	0.562878	-3.556197	1.241084
C	0.081281	-1.675408	2.553044	C	0.280429	-0.963669	2.738107	C	0.084614	-1.832244	2.427125
H	-0.193252	-0.654058	2.278659	H	-0.261577	-1.102268	3.682879	H	-0.604257	-2.165431	3.205121
C	1.505279	-1.679825	3.106186	H	-0.135164	-0.045626	2.309228	H	-0.177813	-0.793134	2.214543
H	1.594467	-0.998502	3.951200	C	1.726529	-0.755423	3.030680	C	1.509186	-1.886780	2.976105
H	1.776746	-2.677275	3.452757	H	2.045540	0.093626	3.616005	H	1.607792	-1.258074	3.859965
H	2.231115	-1.375147	2.352139	H	2.470219	-1.479251	2.733744	H	1.768935	-2.906294	3.262174
C	2.649872	-3.335402	-0.913693	C	2.614080	-3.479317	-0.218686	H	2.237680	-1.546570	2.240030
H	2.220008	-3.946155	-0.119951	H	2.274727	-3.898032	0.729966	C	2.628180	-3.313940	-1.137473
H	2.105943	-3.561016	-1.833238	H	1.960473	-3.873966	-0.998636	H	2.191958	-3.965602	-0.380586
H	3.679443	-3.652852	-1.057944	H	3.619651	-3.849837	-0.406907	H	2.080322	-3.477847	-2.067693
C	0.123386	-1.080688	-2.894888	C	-0.234462	-1.919872	-2.613286	C	0.126737	-0.914958	-2.975860
H	0.977760	-0.411390	-2.781716	H	0.773806	-1.528393	-2.475662	H	0.989354	-0.263957	-2.824826
H	0.481613	-2.094398	-2.729659	H	-0.198843	-2.990536	-2.400053	H	0.472800	-1.940959	-2.871956
H	-0.238996	-1.004683	-3.917261	H	-0.511917	-1.797176	-3.658109	H	-0.236048	-0.773879	-3.991155
O	-3.971221	1.317045	-1.954248	O	-4.338509	0.689187	-2.080429	O	-3.937090	1.471874	-1.885869
H	-3.826850	1.562841	-2.876512	H	-4.262649	0.597187	-3.026549	H	-3.790958	1.770350	-2.792173
O	4.853611	0.994615	-0.465950	O	5.008006	0.778039	-0.536701	O	4.885123	0.954806	-0.437477
H	5.586193	0.431309	-0.732363	H	5.705161	0.134002	-0.632281	H	5.610431	0.399537	-0.738427

C	2.505736	2.416792	0.400058	C	2.684902	2.401933	-0.165413	C	2.555908	2.351209	0.516404
C	3.459708	2.694627	1.574366	C	3.581034	2.870717	0.993033	C	3.514752	2.547124	1.703202
H	4.491329	2.467350	1.321882	H	4.619706	2.590017	0.835325	H	4.543191	2.322820	1.435532
H	3.400991	3.748843	1.847017	H	3.526044	3.957018	1.083895	H	3.469251	3.583881	2.038198
H	3.175514	2.103174	2.445943	H	3.241182	2.431702	1.932638	H	3.224527	1.908336	2.538634
C	1.102472	2.856977	0.829755	C	1.288508	2.989005	0.066867	C	1.158684	2.781975	0.974400
H	0.367694	2.711595	0.035124	H	0.591555	2.708730	-0.723694	H	0.421136	2.693027	0.174035
H	0.767300	2.326030	1.722815	H	0.872748	2.670199	1.024296	H	0.818251	2.202920	1.834981
H	1.124470	3.919661	1.067830	H	1.360107	4.076784	0.078935	H	1.193949	3.828266	1.275220
C	2.907703	3.258120	-0.823223	C	3.209674	2.957269	-1.498736	C	2.966475	3.258916	-0.655487
H	3.916596	3.033161	-1.156798	H	4.198764	2.570012	-1.731920	H	3.972108	3.042025	-1.003920
H	2.218954	3.082976	-1.651253	H	2.531643	2.694212	-2.311757	H	2.274545	3.141691	-1.491055
H	2.863099	4.316680	-0.565090	H	3.269241	4.045919	-1.444889	H	2.935120	4.300701	-0.334753
C	-4.242742	0.465019	0.773759	C	-4.232196	0.761171	0.762481	C	-4.215356	0.462375	0.787177
C	-5.590602	0.000619	0.186450	C	-5.603177	0.100311	0.548034	C	-5.569554	0.050071	0.176048
H	-6.383550	0.254628	0.889444	H	-6.341798	0.561972	1.206043	H	-6.358414	0.271366	0.894530
H	-5.816937	0.478889	-0.761824	H	-5.941729	0.209733	-0.480295	H	-5.791301	0.586621	-0.741632
H	-5.598954	-1.080711	0.047181	H	-5.553190	-0.962955	0.786091	H	-5.591270	-1.020875	-0.027278
C	-4.236211	1.996307	0.953675	C	-4.306754	2.254934	0.406097	C	-4.189923	1.980053	1.057847
H	-4.421315	2.522770	0.021942	H	-4.589810	2.406518	-0.633160	H	-4.369832	2.563223	0.159461
H	-5.023144	2.267845	1.656917	H	-5.046453	2.746014	1.040884	H	-4.972554	2.218757	1.777607
H	-3.284740	2.332931	1.367117	H	-3.341467	2.734527	0.576414	H	-3.233872	2.279953	1.488629
C	-4.088906	-0.172698	2.157833	C	-3.896394	0.664446	2.254829	C	-4.067468	-0.258383	2.130549
H	-4.115289	-1.261998	2.109216	H	-3.913066	-0.366869	2.609733	H	-4.107190	-1.342461	2.017277
H	-3.166996	0.138471	2.651262	H	-2.918602	1.093522	2.477980	H	-3.141180	0.011707	2.639722
H	-4.920705	0.147908	2.782557	H	-4.641400	1.223138	2.821470	H	-4.894468	0.034513	2.774940
**SW (C10$^{\bullet}$)**				**SW (C11$^{\bullet}$)**				**SW (C12$^{\bullet}$)**			
C	2.538881	0.929900	0.062627	C	2.542035	0.960073	0.098021	C	2.517527	0.934078	0.053418
C	1.411540	0.124786	0.096621	C	1.417471	0.150775	0.123668	C	1.390398	0.130086	0.112091
C	1.400325	-1.228646	-0.266255	C	1.411067	-1.199061	-0.252476	C	1.374982	-1.228910	-0.229194
C	2.587876	-1.833804	-0.703446	C	2.600861	-1.795794	-0.695114	C	2.557670	-1.841416	-0.669321
C	3.727464	-1.051681	-0.742347	C	3.737752	-1.009396	-0.725888	C	3.696987	-1.060495	-0.732665
C	3.718317	0.298466	-0.378915	C	3.723806	0.337079	-0.349229	C	3.692049	0.295234	-0.390558
H	0.483805	0.557893	0.440399	H	0.488120	0.577281	0.471313	H	0.466475	0.568974	0.458790
H	4.664625	-1.493311	-1.062582	H	4.676550	-1.444623	-1.050070	H	4.630584	-1.507536	-1.055791
C	0.105819	-2.013743	-0.177955	C	0.119251	-1.989453	-0.172396	C	0.081279	-2.011957	-0.114770
H	0.175724	-2.822997	-0.914643	H	0.192220	-2.791192	-0.916962	H	0.143156	-2.832789	-0.839266
C	-1.005968	-1.128234	-0.663482	C	-0.995415	-1.103066	-0.649650	C	-1.035363	-1.133758	-0.602431
C	-0.960440	-0.596413	-2.002988	C	-0.951241	-0.557971	-1.983855	C	-1.003903	-0.623206	-1.950587
C	-2.082855	-0.780813	0.173686	C	-2.073800	-0.767599	0.190461	C	-2.103220	-0.772680	0.240505
C	-1.972194	0.239763	-2.392576	C	-1.965740	0.278489	-2.365619	C	-2.019514	0.207139	-2.342607
C	-3.102838	0.069435	-0.187163	C	-3.096585	0.082622	-0.162431	C	-3.126750	0.072194	-0.122915
H	-2.101889	-1.207441	1.161520	H	-2.091716	-1.203964	1.174053	H	-2.111888	-1.183625	1.235110
C	-3.017611	0.595536	-1.523262	C	-3.012694	0.622101	-1.493270	C	-3.055559	0.577063	-1.467996
H	-1.979118	0.645120	-3.397692	H	-1.973701	0.693664	-3.366712	H	-2.036991	0.596554	-3.353879
C	-0.118693	-2.689872	1.189242	C	-0.103417	-2.679738	1.188012	C	-0.128898	-2.666284	1.265253
H	-1.113838	-3.138642	1.181813	H	-1.097001	-3.131847	1.175786	H	-1.124183	-3.114688	1.275472
H	0.578710	-3.526338	1.258631	H	0.596849	-3.514431	1.249466	H	0.568984	-3.501847	1.340468
C	0.076383	-1.798485	2.428945	C	0.088128	-1.799892	2.436479	C	0.079549	-1.755489	2.488565
H	-0.611914	-2.133172	3.206805	H	-0.599288	-2.144572	3.210745	H	-0.600548	-2.077538	3.278874
H	-0.196746	-0.764096	2.207024	H	-0.188495	-0.764324	2.224611	H	-0.195648	-0.724621	2.253218
C	1.499211	-1.832721	2.984055	C	1.510865	-1.834659	2.991789	C	1.508174	-1.781553	3.029034
H	1.587171	-1.195955	3.863257	H	1.596305	-1.206253	3.877232	H	1.605620	-1.131001	3.897073
H	1.769141	-2.846962	3.279293	H	1.784194	-2.850811	3.277167	H	1.780955	-2.791114	3.337400
H	2.226877	-1.490233	2.248217	H	2.237609	-1.482456	2.259636	H	2.228086	-1.451057	2.280199
C	2.650920	-3.279875	-1.113476	C	2.669053	-3.237546	-1.119289	C	2.615985	-3.293817	-1.057072
H	2.218895	-3.930349	-0.353168	H	2.239003	-3.896937	-0.365572	H	2.191876	-3.932000	-0.282035
H	2.108746	-3.457279	-2.044549	H	2.127832	-3.407675	-2.052273	H	2.063920	-3.485689	-1.979424
H	3.680536	-3.590408	-1.271783	H	3.699793	-3.542952	-1.280228	H	3.643782	-3.607257	-1.221352

C	0.130432	-0.923696	-2.980977	C	0.141109	-0.871871	-2.964577	C	0.076450	-0.966382	-2.934786
H	0.985107	-0.261907	-2.831684	H	0.993437	-0.208632	-2.808459	H	0.932833	-0.302682	-2.805047
H	0.487491	-1.944905	-2.867499	H	0.501655	-1.892905	-2.860984	H	0.434424	-1.985822	-2.808961
O	-3.964206	1.426095	-1.926390	H	-0.219135	-0.734184	-3.981242	H	-0.294459	-0.853390	-3.950660
H	-3.817696	1.718984	-2.834454	O	-3.962009	1.453302	-1.888603	O	-4.006159	1.401550	-1.874154
O	4.857212	1.019276	-0.438525	H	-3.816185	1.755594	-2.793691	H	-3.869211	1.679981	-2.788240
H	5.589897	0.469786	-0.732120	O	4.860223	1.062369	-0.401312	O	4.830435	1.014522	-0.473630
C	2.508670	2.397059	0.494825	H	5.594910	0.518320	-0.699995	H	5.559822	0.460143	-0.766247
C	3.460393	2.613102	1.683859	C	2.506595	2.422807	0.544583	C	2.492284	2.407901	0.462652
H	4.492359	2.398202	1.422117	C	3.457135	2.630456	1.736056	C	3.456621	2.642311	1.637965
H	3.401955	3.651911	2.010384	H	4.489933	2.421699	1.472627	H	4.485697	2.422855	1.368731
H	3.173887	1.977758	2.523192	H	3.394988	3.665802	2.072736	H	3.401921	3.686179	1.948620
C	1.104861	2.815787	0.943756	H	3.172525	1.985920	2.569001	H	3.178867	2.020442	2.490233
H	0.371639	2.712243	0.141174	C	1.101184	2.832258	0.997047	C	1.093426	2.834279	0.919759
H	0.767382	2.239808	1.807559	H	0.368616	2.734064	0.193197	H	0.351707	2.718371	0.126732
H	1.127217	3.864762	1.236354	H	0.765386	2.246667	1.855023	H	0.764975	2.272176	1.796093
C	2.913889	3.299973	-0.682618	C	2.909116	3.338626	-0.623782	H	1.119158	3.887739	1.195475
H	3.923300	3.091588	-1.025270	H	3.919365	3.137101	-0.968064	C	2.885231	3.291908	-0.733154
H	2.226743	3.168386	-1.519994	H	2.222732	3.212891	-1.462681	H	3.890896	3.077697	-1.083148
H	2.869598	4.343855	-0.370338	H	2.861103	4.379234	-0.301292	H	2.189208	3.147391	-1.561013
C	-4.242155	0.434793	0.753490	C	-4.237499	0.434794	0.781311	H	2.844527	4.340617	-0.436972
C	-5.589151	0.002527	0.140242	C	-5.582770	0.003918	0.163318	C	-4.255931	0.452870	0.823866
H	-6.383371	0.220678	0.853764	H	-6.377998	0.212312	0.878632	C	-5.609468	0.011539	0.231844
H	-5.813104	0.529262	-0.782573	H	-5.808209	0.538907	-0.754373	H	-6.396019	0.241287	0.950204
H	-5.598084	-1.070175	-0.054614	H	-5.587924	-1.066845	-0.042058	H	-5.843056	0.523712	-0.696762
C	-4.234765	1.954762	1.012137	C	-4.235457	1.952168	1.054863	H	-5.620747	-1.064106	0.054085
H	-4.417482	2.528734	0.108410	H	-4.419831	2.534347	0.156740	C	-4.245400	1.976735	1.058346
H	-5.022956	2.190399	1.726823	H	-5.024716	2.178057	1.771515	H	-4.437541	2.536423	0.147641
H	-3.283894	2.268749	1.444345	H	-3.285833	2.265181	1.490508	H	-5.025906	2.223984	1.777530
C	-4.091747	-0.273580	2.103155	C	-4.085130	-0.286266	2.124020	H	-3.289916	2.297110	1.475430
H	-4.118908	-1.358899	1.998382	H	-4.108501	-1.370593	2.008591	C	-4.091403	-0.234136	2.182896
H	-3.170626	0.010890	2.613870	H	-3.165183	-0.003643	2.637866	H	-4.119957	-1.320965	2.095596
H	-4.924599	0.015150	2.741864	H	-4.919207	-0.006697	2.765202	H	-3.164843	0.057978	2.679255

SPLET (SP step) mechanism

**M06-2X/6-311++G(2d,2p)**											
**SW**				**SW ($O1^{-}$)**				**SW ($O2^{-}$)**			
C	-2.67469700	-0.88809800	-0.06533000	C	2.658923	0.882200	-0.076842	C	2.701272	0.890911	-0.075165
C	-1.49051600	-0.15717800	-0.02475800	C	1.471560	0.156821	-0.030254	C	1.517741	0.159126	-0.031340
C	-1.41417100	1.22111900	-0.21044200	C	1.391252	-1.224856	-0.186868	C	1.441998	-1.219346	-0.215972
C	-2.58638600	1.92518400	-0.49075900	C	2.562896	-1.939239	-0.442369	C	2.614079	-1.922706	-0.498606
C	-3.78020800	1.21581600	-0.55533800	C	3.760009	-1.235961	-0.512415	C	3.807211	-1.212481	-0.566446
C	-3.83614600	-0.15458700	-0.34159200	C	3.819652	0.138506	-0.327744	C	3.862619	0.158099	-0.353699
H	-0.56422600	-0.68096000	0.15836000	H	0.545903	0.687937	0.133965	H	0.591490	0.682327	0.153629
H	-4.70095500	1.74561100	-0.77700400	H	4.680412	-1.773897	-0.715088	H	4.727832	-1.741724	-0.789947
C	-0.08417000	1.94094100	-0.03587700	C	0.057033	-1.935699	-0.007755	C	0.112955	-1.940057	-0.037819
H	-0.11639200	2.84401300	-0.65122900	H	0.090656	-2.851933	-0.603256	H	0.144408	-2.843495	-0.652674
C	1.08885300	1.12039600	-0.55451400	C	-1.108482	-1.122072	-0.553509	C	-1.061905	-1.120724	-0.554208
C	1.16100900	0.79742800	-1.91592000	C	-1.168320	-0.828234	-1.922092	C	-1.137512	-0.798674	-1.915645
C	2.11018100	0.67754000	0.28152600	C	-2.134786	-0.657438	0.264422	C	-2.081594	-0.678107	0.283956
C	2.23976300	0.04566800	-2.35968800	C	-2.240450	-0.082120	-2.390745	C	-2.217875	-0.048007	-2.357344
C	3.19890400	-0.08696300	-0.13896100	C	-3.217022	0.101955	-0.181312	C	-3.171881	0.085310	-0.134447
H	2.05975100	0.94168100	1.32445800	H	-2.093858	-0.899241	1.313172	H	-2.028508	-0.941548	1.326933
C	3.23757100	-0.39732800	-1.50128700	C	-3.243415	0.383047	-1.550269	C	-3.213991	0.394782	-1.496874
H	2.31275100	-0.20270100	-3.41369200	H	-2.303907	0.143762	-3.450409	H	-2.293533	0.199639	-3.411330
C	0.07631600	2.42996200	1.42138500	C	-0.117197	-2.392603	1.458325	C	-0.043729	-2.428275	1.420126
H	1.06319300	2.88574500	1.51686900	H	-1.106617	-2.842476	1.555507	H	-1.030034	-2.884740	1.518224

H	-0.64355200	3.23527900	1.58323200	H	0.598133	-3.196973	1.643345	H	0.677126	-3.232947	1.580786
C	-0.13989500	1.37000700	2.51921000	C	0.094316	-1.310074	2.534831	C	0.174269	-1.367462	2.516769
H	0.58518500	1.53223900	3.31962500	H	-0.637865	-1.452274	3.332586	H	-0.548800	-1.529732	3.318992
H	0.06298300	0.37290100	2.12532200	H	-0.101404	-0.320921	2.117939	H	-0.030288	-0.370759	2.122730
C	-1.54749400	1.39404400	3.11219800	C	1.496957	-1.326672	3.139703	C	1.583279	-1.390064	3.106453
H	-1.66369500	0.63048600	3.88153600	H	1.609949	-0.547165	3.893365	H	1.700717	-0.625931	3.875032
H	-1.75316600	2.36329200	3.56957100	H	1.695066	-2.286616	3.619487	H	1.790759	-2.358867	3.563953
H	-2.30196700	1.21701700	2.34508000	H	2.258318	-1.169075	2.375158	H	2.335809	-1.212956	2.337447
C	-2.59016000	3.41059700	-0.74282400	C	2.562816	-3.429740	-0.662386	C	2.618379	-3.408275	-0.749740
H	-2.13947400	3.96279200	0.08169800	H	2.103275	-3.962332	0.170105	H	2.170054	-3.960286	0.076191
H	-2.02523000	3.65692400	-1.64418600	H	2.004236	-3.693281	-1.562836	H	2.051513	-3.655599	-1.649612
H	-3.60591800	3.77696300	-0.87640100	H	3.578161	-3.802750	-0.779730	H	3.634096	-3.773962	-0.885479
C	0.11503800	1.23942700	-2.90510300	C	-0.116130	-1.295404	-2.892942	C	-0.093542	-1.240512	-2.907010
H	-0.81417400	0.68382700	-2.77235900	H	0.814174	-0.740603	-2.764576	H	0.835561	-0.684129	-2.776807
H	-0.12563400	2.29658600	-2.78454700	H	0.119359	-2.350626	-2.747708	H	0.148211	-2.297413	-2.786353
H	0.46384700	1.08774400	-3.92474000	H	-0.456060	-1.164398	-3.918427	H	-0.444866	-1.089738	-3.925918
O	4.28022400	-1.13970500	-1.99211400	O	-4.279101	1.118629	-2.065471	O	-4.258357	1.136063	-1.985712
H	4.15038500	-1.27447200	-2.93347100	O	5.025359	0.785734	-0.398260	H	-4.130838	1.270331	-2.927458
O	-5.03877100	-0.80796300	-0.40791800	H	5.715155	0.143103	-0.578146	O	5.064592	0.812337	-0.423272
H	-5.72950400	-0.17209300	-0.60719000	C	2.693737	2.396327	0.144922	C	2.731615	2.400227	0.177847
C	-2.70558000	-2.39723100	0.18870900	C	3.563571	2.721347	1.371136	C	3.612878	2.703685	1.401423
C	-3.58418500	-2.69925100	1.41455000	H	4.589017	2.388049	1.236104	H	4.638330	2.378119	1.248727
H	-4.60974900	-2.37300800	1.26406400	H	3.569740	3.799559	1.540801	H	3.615990	3.778139	1.593531
H	-3.58765200	-3.77358000	1.60734600	H	3.155805	2.239246	2.261172	H	3.216705	2.201197	2.285397
H	-3.18555200	-2.19650300	2.29727000	C	1.293525	2.958414	0.410852	C	1.331776	2.950032	0.470112
C	-1.30547000	-2.94790600	0.47802400	H	0.619125	2.787079	-0.429138	H	0.649321	2.793085	-0.366176
H	-0.62487000	-2.79200200	-0.35996900	H	0.847203	2.523911	1.306694	H	0.896896	2.494827	1.361290
H	-0.86814900	-2.49246600	1.36788600	H	1.369144	4.035001	0.565001	H	1.404176	4.023512	0.645948
H	-1.37826300	-4.02122000	0.65471000	C	3.250976	3.104699	-1.100710	C	3.272479	3.137062	-1.058457
C	-3.24991000	-3.13443900	-1.04585000	H	4.268248	2.792160	-1.320404	H	4.288770	2.833977	-1.295272
H	-4.26652800	-2.83073800	-1.28046200	H	2.626103	2.890438	-1.969024	H	2.639442	2.938024	-1.924477
H	-2.61876600	-2.93642600	-1.91348500	H	3.249562	4.184204	-0.939629	H	3.267912	4.212951	-0.874824
H	-3.24571900	-4.21021500	-0.86154600	C	-4.307329	0.597434	0.772499	C	-4.254273	0.555627	0.840874
C	4.28323700	-0.55747900	0.83410600	C	-5.672249	0.021894	0.359950	C	-5.620871	-0.017610	0.430680
C	5.64929600	0.01446800	0.42033000	H	-6.441140	0.370270	1.052047	H	-6.383970	0.312616	1.137920
H	6.41381100	-0.31588600	1.12597900	H	-5.950480	0.328211	-0.644750	H	-5.911070	0.308293	-0.564426
H	5.93690400	-0.31228200	-0.57525100	H	-5.650259	-1.068116	0.396559	H	-5.593594	-1.108030	0.444249
H	5.62287100	1.10491700	0.43327100	C	-4.357884	2.134565	0.758669	C	-4.311883	2.092456	0.859731
C	4.33973500	-2.09433900	0.85380100	H	-4.588670	2.520202	-0.230629	H	-4.554834	2.497563	-0.118815
H	4.58007500	-2.50024700	-0.12505800	H	-5.125180	2.483138	1.452408	H	-5.073371	2.422853	1.568600
H	5.10264300	-2.42486100	1.56108300	H	-3.399407	2.547283	1.077358	H	-3.351960	2.502950	1.176937
H	3.38025400	-2.50390800	1.17352800	C	-4.038711	0.159159	2.216134	C	-3.968458	0.088591	2.272136
C	4.00114700	-0.08932300	2.26574100	H	-4.026554	-0.927242	2.314940	H	-3.950373	-0.999566	2.348063
H	3.98406000	0.99889500	2.34102000	H	-3.092273	0.552269	2.591066	H	-3.019891	0.478251	2.645287
H	3.05317000	-0.47803200	2.64137200	H	-4.834627	0.541653	2.855410	H	-4.759291	0.453902	2.927566
H	4.79324800	-0.45481600	2.91953700								
**SW ($C1^{-}$)**				**SW ($C2^{-}$)**				**SW ($C3^{-}$)**			
C	2.677145	0.892165	-0.063842	C	2.702422	0.863584	-0.068503	C	2.675465	0.874565	-0.058522
C	1.493171	0.160879	-0.023809	C	1.513829	0.139693	-0.031026	C	1.491107	0.143516	-0.026299
C	1.417232	-1.217320	-0.210381	C	1.428475	-1.236517	-0.227930	C	1.414414	-1.232534	-0.227824
C	2.589665	-1.920869	-0.491080	C	2.595606	-1.945470	-0.517001	C	2.586442	-1.933615	-0.516284
C	3.783288	-1.211118	-0.555133	C	3.793647	-1.242990	-0.578687	C	3.780435	-1.223843	-0.572761
C	3.838820	0.159164	-0.340505	C	3.858614	0.125219	-0.353736	C	3.836720	0.143996	-0.343265
H	4.704200	-1.740507	-0.777084	H	0.591260	0.667658	0.158741	H	0.564954	0.665384	0.162882
C	0.087427	-1.937635	-0.036357	C	0.094463	-1.949520	-0.056009	H	4.701041	-1.751278	-0.800556
H	0.119944	-2.840303	-0.652286	H	0.119570	-2.847636	-0.678899	C	0.084245	-1.953991	-0.061481
C	-1.085800	-1.117094	-0.554537	C	-1.074711	-1.117457	-0.564895	C	-1.088602	-1.127247	-0.570582
C	-1.157968	-0.793273	-1.915740	C	-1.148196	-0.782761	-1.923394	C	-1.160747	-0.788620	-1.928179
C	-2.107305	-0.675067	0.281726	C	-2.091219	-0.675244	0.277337	C	-2.109780	-0.693788	0.270551

C	-2.236911	-0.041538	-2.359090	C	-2.223346	-0.020683	-2.358219	C	-2.239339	-0.031540	-2.363211
C	-3.196222	0.089394	-0.138335	C	-3.176202	0.099444	-0.134077	C	-3.198336	0.075768	-0.141055
H	-2.056861	-0.939862	1.324491	H	-2.039875	-0.948339	1.317916	H	-2.059363	-0.969923	1.310373
C	-3.234897	0.400621	-1.500464	C	-3.216279	0.421338	-1.493684	C	-3.236995	0.401795	-1.499718
H	-2.309908	0.207486	-3.412939	H	-2.297373	0.236872	-3.409943	H	-2.312318	0.228957	-3.414284
C	-0.073005	-2.427636	1.420582	C	-0.065486	-2.449617	1.397549	C	-0.076288	-2.459706	1.390066
H	-1.059757	-2.883763	1.515715	H	-1.054936	-2.900062	1.491722	H	-1.063272	-2.916316	1.480352
H	0.647084	-3.232851	1.581955	H	0.649764	-3.260688	1.550918	H	0.643390	-3.267008	1.542600
C	0.142837	-1.368324	2.519099	C	0.159990	-1.400168	2.503573	C	0.140238	-1.412505	2.500002
H	-0.582244	-1.531277	3.319367	H	-0.564120	-1.564548	3.304428	H	-0.584842	-1.583757	3.298536
H	-0.060303	-0.371024	2.125839	H	-0.037659	-0.398591	2.118468	H	-0.062415	-0.410883	2.117622
C	1.550408	-1.392338	3.112155	C	1.568861	-1.437834	3.092819	C	1.547860	-1.443707	3.092600
H	1.666344	-0.629240	3.881990	H	1.691684	-0.681421	3.868163	H	1.664287	-0.689079	3.870665
H	1.756331	-2.361820	3.568919	H	1.769632	-2.412099	3.541631	H	1.753317	-2.418203	3.538781
H	2.304875	-1.214603	2.345196	H	2.322537	-1.259118	2.325309	H	2.302339	-1.258051	2.327530
C	2.593880	-3.406119	-0.744097	C	2.589542	-3.428735	-0.781372	C	2.589839	-3.416030	-0.785422
H	2.143303	-3.958972	0.080044	H	2.137458	-3.984955	0.039672	H	2.139059	-3.977564	0.032716
H	2.029074	-3.652030	-1.645651	H	2.020887	-3.664072	-1.683328	H	2.024804	-3.651833	-1.689528
H	3.609751	-3.772108	-0.877849	H	3.602676	-3.800257	-0.920520	H	3.605501	-3.781084	-0.923259
C	-0.111812	-1.234338	-2.905144	C	-0.107415	-1.222998	-2.918817	C	-0.114934	-1.219465	-2.922436
H	0.817233	-0.678557	-2.771989	H	0.825550	-0.674278	-2.783800	H	0.814421	-0.665657	-2.783358
H	0.129156	-2.291505	-2.785252	H	0.126984	-2.282590	-2.807626	H	0.125485	-2.278000	-2.814063
H	-0.460604	-1.082101	-3.924704	H	-0.457773	-1.060702	-3.936287	H	-0.463756	-1.055976	-3.940242
O	-4.277734	1.143014	-1.990877	O	-4.255503	1.174200	-1.975735	O	-4.279490	1.150025	-1.981917
H	-4.147878	1.278422	-2.932140	H	-4.127136	1.315970	-2.916265	H	-4.149665	1.295582	-2.921668
O	5.041262	0.812927	-0.406340	O	5.065106	0.771670	-0.417655	O	5.039502	0.797798	-0.402133
H	5.732189	0.177383	-0.605979	H	5.751388	0.133214	-0.623915	H	5.730069	0.164093	-0.608744
C	2.707580	2.401143	0.191167	C	2.743294	2.370336	0.197951	C	2.706731	2.380668	0.212861
C	3.586026	2.702629	1.417254	C	3.626758	2.656730	1.424051	C	3.585471	2.668349	1.442050
H	4.611692	2.376776	1.266619	H	4.649905	2.325408	1.268303	H	4.610948	2.343608	1.287766
H	3.589174	3.776835	1.610739	H	3.637366	3.729380	1.625731	H	3.589212	3.740388	1.647193
H	3.187485	2.199200	2.299627	H	3.227175	2.149149	2.303570	H	3.186759	2.155577	2.318948
C	1.307295	2.951231	0.480752	C	1.347343	2.927243	0.495319	C	1.306771	2.928321	0.508566
H	0.626790	2.795670	-0.357381	H	0.663737	2.782514	-0.342232	H	0.626091	2.782234	-0.331129
H	0.870052	2.495095	1.370296	H	0.909384	2.467148	1.382469	H	0.869382	2.462780	1.393153
H	1.379770	4.024453	0.658131	H	1.427230	3.998583	0.680708	H	1.379836	3.999513	0.697585
C	3.251772	3.139299	-1.042887	C	3.289164	3.114384	-1.031816	C	3.251180	3.131897	-1.013163
H	4.268491	2.836040	-1.277633	H	4.303300	2.806356	-1.271478	H	4.267712	2.830666	-1.251307
H	2.620736	2.941661	-1.910686	H	2.654680	2.927487	-1.899480	H	2.619944	2.944034	-1.882986
H	3.247262	4.214955	-0.857893	H	3.292103	4.188598	-0.838596	H	3.247263	4.205482	-0.816495
C	-4.280747	0.558975	0.834969	C	-4.255206	0.568567	0.845563	C	-4.282505	0.535323	0.837418
C	-5.646618	-0.013098	0.420745	C	-5.625799	0.008537	0.430481	C	-5.648725	-0.031491	0.417161
H	-6.411269	0.316584	1.126560	H	-6.386518	0.337747	1.140753	H	-6.413124	0.290910	1.126605
H	-5.934261	0.314208	-0.574643	H	-5.913811	0.345312	-0.561636	H	-5.936303	0.306762	-0.574579
H	-5.619881	-1.103547	0.432988	H	-5.606112	-1.082124	0.434322	H	-5.622567	-1.122023	0.417555
C	-4.337687	2.095806	0.855646	C	-4.302115	2.105531	0.878131	C	-4.338625	2.071869	0.874796
H	-4.578085	2.502273	-0.122967	H	-4.542328	2.521028	-0.096728	H	-4.578914	2.489071	-0.099316
H	-5.100732	2.425655	1.563095	H	-5.061221	2.434885	1.590033	H	-5.101416	2.394419	1.585874
H	-3.378342	2.505445	1.175693	H	-3.339329	2.506488	1.198839	H	-3.379027	2.477497	1.199163
C	-3.998608	0.089982	2.266320	C	-3.972522	0.086809	2.272561	C	-4.000459	0.050659	2.263557
H	-3.981214	-0.998279	2.340902	H	-3.962006	-1.002081	2.338779	H	-3.983635	-1.038358	2.326311
H	-3.050765	0.478721	2.642257	H	-3.021233	0.466514	2.649027	H	-3.052367	0.434788	2.643586
H	-4.790852	0.454828	2.920303	H	-4.760735	0.451757	2.931341	H	-4.792437	0.408800	2.921557
**SW ($C4^{-}$)**				**SW ($C5^{-}$)**				**SW ($C6^{-}$)**			
C	2.663447	0.886958	-0.070362	C	2.664330	0.887872	-0.071205	C	2.667552	0.878399	-0.084577
C	1.480402	0.154658	-0.022242	C	1.479699	0.157503	-0.033990	C	1.485080	0.146180	-0.023019
C	1.405719	-1.225080	-0.197612	C	1.403842	-1.221475	-0.214759	C	1.411671	-1.236783	-0.171455
C	2.578443	-1.929512	-0.474859	C	2.577257	-1.927000	-0.486249	C	2.585186	-1.945506	-0.434037

C	3.771117	-1.218927	-0.546844	C	3.771646	-1.218278	-0.547338	C	3.777323	-1.235456	-0.519024
C	3.825456	0.153096	-0.343318	C	3.826947	0.152920	-0.338584	C	3.830372	0.140326	-0.342301
H	0.553687	0.678447	0.158692	H	0.552650	0.682290	0.142280	H	0.557796	0.672622	0.146975
H	4.692228	-1.749021	-0.766270	H	4.693351	-1.749225	-0.762161	H	4.699036	-1.768958	-0.727384
C	0.077080	-1.945503	-0.015319	C	0.072699	-1.940182	-0.044356	C	0.083496	-1.954628	0.023912
H	0.109497	-2.853045	-0.624049	H	0.107812	-2.845629	-0.656049	H	0.117118	-2.873861	-0.566949
C	-1.098067	-1.130493	-0.537861	C	-1.097320	-1.121256	-0.572256	C	-1.091939	-1.150974	-0.515305
C	-1.173155	-0.817687	-1.901479	C	-1.162269	-0.803513	-1.935253	C	-1.166274	-0.864930	-1.884828
C	-2.118521	-0.682945	0.296747	C	-2.122835	-0.674847	0.256739	C	-2.113383	-0.688069	0.309659
C	-2.253798	-0.070773	-2.348818	C	-2.238435	-0.053115	-2.387529	C	-2.247204	-0.127809	-2.347457
C	-3.209110	0.076869	-0.127388	C	-3.209086	0.088388	-0.172358	C	-3.204287	0.062394	-0.130029
C	-3.250692	0.377114	-1.491894	H	-2.077932	-0.934983	1.300929	H	-2.061246	-0.923929	1.359265
H	-2.329053	0.169704	-3.404491	C	-3.240547	0.393513	-1.536052	C	-3.245105	0.335891	-1.500170
C	-0.080060	-2.423984	1.445801	C	-0.095547	-2.423531	1.413923	H	-2.321876	0.091936	-3.407681
H	-1.066101	-2.880466	1.546442	H	-1.083067	-2.878618	1.506017	C	-0.074331	-2.404607	1.493986
H	0.641266	-3.227050	1.612275	H	0.623187	-3.228454	1.582620	H	0.647537	-3.203659	1.676640
C	0.136601	-1.355645	2.535380	C	0.115311	-1.359424	2.508769	C	0.140635	-1.315005	2.562643
H	-0.586793	-1.513005	3.338289	H	-0.613986	-1.518332	3.306013	H	-0.583225	-1.457261	3.367947
H	-0.068434	-0.361766	2.134515	H	-0.085165	-0.363777	2.109991	H	-0.064927	-0.329320	2.142290
C	1.545305	-1.373283	3.125966	C	1.519793	-1.381638	3.109172	C	1.548913	-1.319914	3.154483
H	1.661791	-0.603901	3.889436	H	1.632250	-0.615159	3.876158	H	1.664195	-0.535666	3.902862
H	1.753208	-2.338833	3.590103	H	1.722741	-2.349184	3.571341	H	1.757274	-2.276036	3.637545
H	2.298131	-1.200841	2.356190	H	2.278314	-1.207816	2.345320	H	2.302167	-1.161906	2.382033
C	2.583914	-3.416738	-0.715963	C	2.581826	-3.413374	-0.732568	C	2.592069	-3.437153	-0.646037
H	2.135521	-3.963478	0.113430	H	2.126658	-3.962240	0.091715	H	2.143515	-3.967965	0.193553
H	2.017711	-3.670522	-1.614453	H	2.021514	-3.662987	-1.635906	H	2.026744	-3.708919	-1.539809
H	3.599958	-3.782621	-0.848682	H	3.598139	-3.780586	-0.859438	H	3.608515	-3.804701	-0.770846
C	-0.128336	-1.265474	-2.889276	C	-0.111313	-1.249664	-2.917262	C	-0.120351	-1.331045	-2.862930
H	0.800308	-0.707576	-2.762327	H	0.817388	-0.693862	-2.781818	H	0.807736	-0.769990	-2.746230
H	0.114085	-2.321367	-2.761361	H	0.128359	-2.306430	-2.791382	H	0.122851	-2.384033	-2.714236
H	-0.479209	-1.121822	-3.909368	H	-0.454752	-1.101797	-3.939280	H	-0.470586	-1.207645	-3.885888
O	-4.295306	1.114350	-1.986287	O	-4.280370	1.134336	-2.035162	O	-4.289964	1.062447	-2.009629
H	-4.167366	1.242351	-2.928847	H	-4.145581	1.265432	-2.976342	H	-4.161431	1.172118	-2.954417
O	5.027011	0.807690	-0.416652	O	5.030129	0.805641	-0.401158	O	5.031437	0.794377	-0.427537
H	5.718303	0.171359	-0.612484	H	5.721669	0.168781	-0.594377	H	5.723402	0.154925	-0.610398
C	2.692603	2.397967	0.172478	C	2.694415	2.397962	0.177176	C	2.695274	2.393888	0.128719
C	3.572987	2.710289	1.394456	C	3.566727	2.704416	1.406407	C	3.574491	2.730765	1.345001
H	4.598748	2.384417	1.244515	H	4.592947	2.377258	1.262525	H	4.600633	2.402891	1.202199
H	3.575246	3.786015	1.579313	H	3.569564	3.779478	1.595079	H	3.575720	3.809899	1.508809
H	3.176681	2.213498	2.281586	H	3.163325	2.205204	2.288967	H	3.177939	2.251065	2.241379
C	1.292223	2.948752	0.460270	C	1.293009	2.950208	0.457068	C	1.294224	2.949008	0.404680
H	0.610334	2.785690	-0.375310	H	0.616730	2.791299	-0.383854	H	0.613091	2.769078	-0.428050
H	0.857173	2.499266	1.354263	H	0.850899	2.498344	1.346386	H	0.858884	2.516704	1.306967
H	1.363780	4.023445	0.628899	H	1.365254	4.024171	0.629995	H	1.364764	4.026849	0.552333
C	3.233629	3.126823	-1.068476	C	3.245426	3.130229	-1.057360	C	3.236616	3.098820	-1.125844
H	4.250260	2.822859	-1.302691	H	4.263147	2.825292	-1.285499	H	4.253672	2.791196	-1.353338
H	2.601204	2.921505	-1.933476	H	2.618742	2.929081	-1.927503	H	2.605003	2.876109	-1.987126
H	3.228213	4.203923	-0.892107	H	3.240650	4.206708	-0.877225	H	3.230175	4.179154	-0.970555
C	-4.292361	0.552998	0.844153	C	-4.298310	0.563006	0.793226	C	-4.288654	0.556497	0.831238
C	-5.658336	-0.023948	0.437096	C	-5.662394	-0.010083	0.374543	C	-5.653847	-0.029444	0.434539
H	-6.422051	0.310508	1.141679	H	-6.430460	0.323239	1.074920	H	-6.418362	0.318067	1.131898
H	-5.948219	0.295031	-0.560344	H	-5.944697	0.312923	-0.623775	H	-5.943254	0.269744	-0.569152
H	-5.630308	-1.114233	0.458035	H	-5.636417	-1.100482	0.391821	H	-5.624930	-1.119088	0.476793
C	-4.351050	2.089879	0.852610	C	-4.354375	2.099949	0.806706	C	-4.348624	2.093199	0.809627
H	-4.593749	2.488205	-0.128778	H	-4.589470	2.502161	-0.174948	H	-4.590925	2.472073	-0.179531
H	-5.113155	2.424517	1.558822	H	-5.120842	2.433445	1.508725	H	-5.111531	2.440926	1.508613
H	-3.391587	2.503174	1.167560	H	-3.396430	2.510432	1.129849	H	-3.389738	2.513378	1.117139
C	-4.007003	0.095828	2.278688	C	-4.023846	0.100278	2.228104	C	-4.003981	0.127678	2.274637
H	-3.988203	-0.991779	2.361963	H	-4.007531	-0.987647	2.307662	H	-3.984340	-0.958078	2.379157

H	-3.058912	0.488659	2.649714	H	-3.077703	0.490120	2.607170	H	-3.056492	0.528483	2.638607
H	-4.798448	0.464999	2.931210	H	-4.819215	0.468548	2.876351	H	-4.796216	0.508858	2.919248
**SW (C7^{-})**				**SW (C8^{-})**				**SW (C9^{-})**			
C	2.671247	0.880661	-0.078128	C	2.685931	0.865795	-0.078819	C	2.700487	0.844096	-0.056989
C	1.487604	0.150462	-0.015092	C	1.499362	0.141048	-0.008296	C	1.509039	0.124466	-0.029428
C	1.411936	-1.232638	-0.161097	C	1.419564	-1.244341	-0.128042	C	1.418328	-1.248668	-0.244561
C	2.584259	-1.943714	-0.422638	C	2.590759	-1.964381	-0.369319	C	2.582752	-1.958330	-0.542672
C	3.777527	-1.235740	-0.509067	C	3.787001	-1.262341	-0.462747	C	3.783571	-1.259826	-0.594720
C	3.832829	0.140261	-0.334757	C	3.846254	0.116567	-0.314680	C	3.853855	0.105018	-0.351654
H	0.561201	0.678698	0.154138	H	0.573936	0.675652	0.145578	H	0.588495	0.653507	0.167058
H	4.698341	-1.771094	-0.716649	H	4.707025	-1.804782	-0.654860	H	4.698431	-1.793992	-0.829862
C	0.082636	-1.947994	0.035748	C	0.086661	-1.951162	0.075168	C	0.081460	-1.958619	-0.082464
H	0.114672	-2.868311	-0.553510	H	0.118645	-2.882780	-0.496060	H	0.103234	-2.848501	-0.717185
C	-1.091591	-1.143385	-0.504674	C	-1.081707	-1.153069	-0.487169	C	-1.084259	-1.115286	-0.580611
C	-1.165699	-0.859615	-1.874683	C	-1.147288	-0.895522	-1.862781	C	-1.155978	-0.762345	-1.934579
C	-2.112145	-0.677389	0.319651	C	-2.105098	-0.667731	0.322345	C	-2.099293	-0.680257	0.267122
C	-2.245516	-0.121559	-2.338418	C	-2.221906	-0.162815	-2.346640	C	-2.227975	0.009657	-2.359573
C	-3.201911	0.074066	-0.121164	C	-3.189742	0.078849	-0.138912	C	-3.181080	0.104083	-0.134299
H	-2.060209	-0.911498	1.369659	H	-2.059757	-0.881721	1.376942	H	-2.049367	-0.967306	1.304007
C	-3.242522	0.345233	-1.491775	C	-3.221869	0.323667	-1.514697	C	-3.219442	0.444106	-1.489536
H	-2.320014	0.096453	-3.399012	H	-2.289808	0.034955	-3.411635	H	-2.300640	0.281404	-3.407815
C	-0.075666	-2.395147	1.506633	C	-0.081299	-2.369311	1.553495	C	-0.080934	-2.477281	1.364301
H	-1.062117	-2.848624	1.616419	H	-1.069949	-2.817170	1.666519	H	-1.072183	-2.925028	1.452228
H	0.644942	-3.195043	1.690563	H	0.635432	-3.168000	1.756801	H	0.631063	-3.293127	1.507114
C	0.141243	-1.304027	2.573348	C	0.133647	-1.258576	2.600174	C	0.148311	-1.443465	2.484186
H	-0.062798	-0.318747	2.151307	H	-0.595204	-1.380182	3.404358	H	-0.576704	-1.615577	3.282593
C	1.549613	-1.310177	3.164958	H	-0.064561	-0.280920	2.158100	H	-0.045263	-0.436106	2.112321
H	1.666290	-0.524809	3.911945	C	1.538721	-1.258187	3.199604	C	1.556829	-1.494486	3.073270
H	1.756512	-2.265788	3.649656	H	1.654099	-0.458966	3.931955	H	1.682377	-0.748895	3.858596
H	2.302989	-1.154737	2.392106	H	2.296873	-1.120291	2.428094	H	1.753613	-2.475392	3.509198
C	2.588696	-3.435738	-0.632031	C	2.591023	-3.460174	-0.549896	H	2.311455	-1.308598	2.308402
H	2.139429	-3.964357	0.208561	H	2.135270	-3.970922	0.298219	C	2.570930	-3.437929	-0.826661
H	2.022780	-3.708152	-1.525231	H	2.029046	-3.747805	-1.440809	H	2.116388	-4.003184	-0.013179
H	3.604526	-3.805146	-0.756368	H	3.606195	-3.835424	-0.661450	H	2.001649	-3.659060	-1.731811
C	-0.120698	-1.329129	-2.852145	C	-0.098623	-1.387415	-2.825198	C	-0.116613	-1.193466	-2.935457
H	0.808314	-0.769371	-2.736582	H	0.831732	-0.828751	-2.715287	H	0.818463	-0.650266	-2.792928
H	0.120829	-2.382247	-2.701651	H	0.138316	-2.438265	-2.653101	H	0.113573	-2.255354	-2.838241
H	-0.470908	-1.206951	-3.875258	H	-0.442779	-1.283783	-3.852404	H	-0.465994	-1.016333	-3.950786
O	-4.286292	1.072584	-2.002327	O	-4.260222	1.044643	-2.044957	O	-4.255533	1.207377	-1.961862
H	-4.157744	1.180397	-2.947327	H	-4.126110	1.133762	-2.991131	H	-4.126300	1.361079	-2.900397
O	5.034935	0.792220	-0.421339	O	5.051134	0.762571	-0.407133	O	5.062905	0.747495	-0.406674
H	5.725834	0.151333	-0.603199	H	5.740731	0.115908	-0.572771	H	5.746734	0.109124	-0.621180
C	2.701454	2.396474	0.132513	C	2.720388	2.385275	0.102740	C	2.747208	2.347016	0.229405
C	3.581422	2.734054	1.348056	C	3.594875	2.743191	1.316419	C	3.631391	2.613665	1.459433
H	4.607009	2.404275	1.205655	H	4.620045	2.407179	1.186095	H	4.653276	2.280401	1.299594
H	3.584422	3.813470	1.509977	H	3.600838	3.825512	1.457526	H	3.646159	3.683500	1.675301
H	3.184249	2.256563	2.245338	H	3.191087	2.284474	2.220516	H	3.229522	2.096064	2.332044
C	1.301349	2.954338	0.407739	C	1.320794	2.953186	0.359388	C	1.353364	2.905439	0.533735
H	0.619784	2.774054	-0.424560	H	0.643165	2.759241	-0.473054	H	0.669469	2.774508	-0.305848
H	0.865466	2.524316	1.310854	H	0.878433	2.542174	1.268187	H	0.913305	2.435369	1.414594
H	1.373656	4.032321	0.553496	H	1.396145	4.033522	0.484746	H	1.437411	3.973907	0.733315
C	3.243719	3.098336	-1.123371	C	3.272033	3.060902	-1.163405	C	3.296410	3.105121	-0.990252
H	4.260238	2.788672	-1.350498	H	4.288671	2.743399	-1.378876	H	4.309402	2.796296	-1.233691
H	2.611599	2.875141	-1.984157	H	2.643852	2.823334	-2.023226	H	2.661478	2.932233	-1.860490
H	3.239050	4.178949	-0.969970	H	3.270377	4.144283	-1.030883	H	3.303517	4.176664	-0.782825
C	-4.285313	0.571599	0.839421	C	-4.276615	0.598563	0.805878	C	-4.258549	0.564447	0.851169
C	-5.651519	-0.012828	0.443976	C	-5.642708	0.011349	0.414189	C	-5.631202	0.015372	0.428330
H	-6.415352	0.337137	1.140856	H	-6.409098	0.377331	1.099945	H	-6.390850	0.338147	1.142688
H	-5.940614	0.285073	-0.560188	H	-5.925227	0.290824	-0.597118	H	-5.917559	0.366386	-0.559321

H	-5.624354	-1.102441	0.488130	H	-5.619672	-1.077297	0.479481	H	-5.615813	-1.075314	0.417734
C	-4.342805	2.108357	0.815133	C	-4.328491	2.134756	0.751665	C	-4.299412	2.101018	0.904072
H	-4.584663	2.485892	-0.174645	H	-4.563569	2.493965	-0.246532	H	-4.537666	2.530330	-0.065266
H	-5.105028	2.458537	1.513639	H	-5.093280	2.500980	1.439051	H	-5.057449	2.423910	1.620063
H	-3.383188	2.527522	1.121748	H	-3.369081	2.556434	1.055469	H	-3.335160	2.493894	1.230327
C	-4.001086	0.144845	2.283519	C	-4.001836	0.198762	2.259487	C	-3.978234	0.062727	2.271741
H	-3.983181	-0.940757	2.389934	H	-3.988387	-0.884642	2.386898	H	-3.972030	-1.026978	2.323538
H	-3.052888	0.544754	2.646627	H	-3.054223	0.602317	2.620081	H	-3.025580	0.433660	2.653464
H	-4.792594	0.528431	2.927596	H	-4.795491	0.597436	2.891649	H	-4.765219	0.422027	2.935078
**SW ($C10^{-}$)**				**SW ($C11^{-}$)**				**SW ($C12^{-}$)**			
C	2.672247	0.885536	-0.060274	C	2.677594	0.919206	-0.044773	C	2.652627	0.886466	-0.069434
C	1.488291	0.153461	-0.038190	C	1.496389	0.182871	-0.017590	C	1.467677	0.157122	-0.023331
C	1.412681	-1.220155	-0.256075	C	1.425792	-1.192320	-0.227056	C	1.387330	-1.219596	-0.218779
C	2.585491	-1.917084	-0.551335	C	2.601042	-1.886660	-0.518691	C	2.555710	-1.923346	-0.515424
C	3.779125	-1.205991	-0.597910	C	3.791991	-1.171420	-0.570243	C	3.750040	-1.215385	-0.585787
C	3.834298	0.159071	-0.352179	C	3.842197	0.195311	-0.332869	C	3.810231	0.153258	-0.362147
H	0.561588	0.672508	0.155986	H	0.567856	0.699635	0.173916	H	0.544004	0.680949	0.172444
H	4.700334	-1.730152	-0.830770	H	4.715028	-1.693555	-0.800399	H	4.667799	-1.744922	-0.820101
C	0.082740	-1.944312	-0.099951	C	0.098643	-1.920464	-0.065805	C	0.057839	-1.938635	-0.037241
H	0.116033	-2.832769	-0.736168	H	0.134923	-2.812654	-0.696610	H	0.082814	-2.837036	-0.659726
C	-1.089968	-1.112293	-0.600711	C	-1.077419	-1.095892	-0.570997	C	-1.118636	-1.112313	-0.538598
C	-1.160620	-0.757672	-1.954296	C	-1.150090	-0.749798	-1.926681	C	-1.203002	-0.778855	-1.896770
C	-2.112458	-0.689417	0.244207	C	-2.101044	-0.671685	0.271877	C	-2.131364	-0.674241	0.310326
C	-2.239123	0.003867	-2.381699	C	-2.231648	0.005094	-2.358138	C	-2.284625	-0.022035	-2.324670
C	-3.200963	0.084314	-0.159636	C	-3.192636	0.095506	-0.136090	C	-3.222716	0.095160	-0.094105
H	-2.063174	-0.977804	1.280751	H	-2.050151	-0.953559	1.310134	H	-2.071608	-0.946410	1.350698
C	-3.238120	0.426369	-1.514395	C	-3.231767	0.429151	-1.492889	C	-3.273623	0.415969	-1.453607
H	-2.310946	0.276736	-3.429707	H	-2.305027	0.271297	-3.407753	H	-2.367067	0.234481	-3.376022
C	-0.079302	-2.467258	1.345310	C	-0.060703	-2.435186	1.382705	C	-0.089803	-2.438506	1.417696
H	-1.066123	-2.925485	1.428947	H	-1.045764	-2.896570	1.469665	H	-1.076491	-2.893451	1.518953
H	0.640672	-3.275884	1.489187	H	0.662357	-3.240231	1.531123	H	0.630223	-3.246166	1.566652
C	0.135204	-1.433133	2.467827	C	0.150521	-1.393442	2.498780	C	0.138418	-1.387298	2.521484
H	-0.590771	-1.614247	3.263366	H	-0.574362	-1.572406	3.295800	H	-0.579403	-1.554490	3.327402
H	-0.067575	-0.427178	2.097068	H	-0.056200	-0.390530	2.122004	H	-0.066498	-0.386898	2.137118
C	1.542103	-1.470515	3.061783	C	1.557856	-1.421957	3.092198	C	1.551465	-1.418073	3.101052
H	1.657101	-0.725076	3.848867	H	1.670478	-0.671308	3.874660	H	1.676118	-0.660590	3.875058
H	1.747590	-2.450100	3.496663	H	1.767220	-2.398099	3.532931	H	1.759815	-2.391104	3.549085
H	2.297425	-1.275377	2.299912	H	2.312052	-1.228655	2.328745	H	2.299014	-1.236386	2.328266
C	2.590119	-3.396209	-0.837985	C	2.611041	-3.367479	-0.796321	C	2.554681	-3.406797	-0.778815
H	2.138655	-3.967648	-0.027114	H	2.162130	-3.935641	0.018262	H	2.110825	-3.964556	0.045667
H	2.026357	-3.621632	-1.745528	H	2.047656	-3.600533	-1.702169	H	1.980933	-3.645351	-1.676692
H	3.606172	-3.759009	-0.978854	H	3.628368	-3.727341	-0.935521	H	3.568540	-3.773731	-0.924692
C	-0.113305	-1.176109	-2.952265	C	-0.101735	-1.170408	-2.922643	C	-0.167065	-1.214941	-2.899050
H	0.815541	-0.623438	-2.805477	H	0.825118	-0.613393	-2.779727	H	0.764262	-0.661832	-2.770795
H	0.127617	-2.235710	-2.856120	H	0.143187	-2.228497	-2.820169	H	0.072986	-2.273366	-2.788807
O	-4.280463	1.179638	-1.989019	H	-0.450559	-1.002593	-3.939745	H	-0.525153	-1.054935	-3.914188
H	-4.149551	1.336378	-2.926818	O	-4.277157	1.175620	-1.971537	O	-4.319603	1.163708	-1.928973
O	5.036758	0.814231	-0.401797	H	-4.147313	1.327124	-2.910345	H	-4.198360	1.305448	-2.870452
H	5.727965	0.183421	-0.615021	O	5.042178	0.854634	-0.387127	O	5.013255	0.805227	-0.434766
C	2.702265	2.388341	0.228946	H	5.735624	0.225117	-0.596873	H	5.701046	0.169810	-0.645339
C	3.579294	2.661978	1.462555	C	2.702154	2.423849	0.235262	C	2.688369	2.393567	0.195787
H	4.605158	2.339689	1.305730	C	3.578791	2.708272	1.466708	C	3.578906	2.684844	1.415602
H	3.582131	3.731518	1.680370	H	4.605770	2.388858	1.311300	H	4.602478	2.358145	1.253031
H	3.179795	2.138633	2.332824	H	3.577750	3.779123	1.677996	H	3.585944	3.757664	1.616531
C	1.301606	2.931633	0.529348	H	3.181696	2.188758	2.340367	H	3.187727	2.176004	2.298167
H	0.622064	2.795090	-0.312872	C	1.299633	2.963738	0.533099	C	1.291936	2.944221	0.502397
H	0.863393	2.455403	1.407815	H	0.620172	2.819529	-0.307907	H	0.603266	2.795786	-0.330343
H	1.373788	4.000557	0.731117	H	0.863652	2.491244	1.414689	H	0.862215	2.482695	1.392825

C	3.247793	3.154342	-0.987421	C	3.244194	3.184445	-0.986046	H	1.368145	4.016040	0.686559
H	4.264804	2.856548	-1.227833	H	4.262185	2.888984	-1.225185	C	3.222330	3.139314	-1.038172
H	2.617759	2.976411	-1.860198	H	2.614379	2.998847	-1.857382	H	4.236207	2.835812	-1.284608
H	3.242982	4.225523	-0.778071	H	3.235494	4.256858	-0.783228	H	2.582767	2.948919	-1.901335
C	-4.286632	0.531624	0.822841	C	-4.279460	0.544749	0.844225	H	3.221634	4.213658	-0.845651
C	-5.651983	-0.030983	0.394191	C	-5.642923	-0.025547	0.419747	C	-4.297117	0.559944	0.892637
H	-6.417462	0.282553	1.106437	H	-6.409200	0.289471	1.130480	C	-5.667929	-0.006679	0.487340
H	-5.938524	0.318809	-0.593840	H	-5.931276	0.317142	-0.570244	H	-6.425260	0.319485	1.202619
H	-5.625167	-1.121428	0.381717	H	-5.612046	-1.115941	0.413907	H	-5.964306	0.328108	-0.502984
C	-4.343725	2.067586	0.878322	C	-4.342255	2.080798	0.890371	H	-5.643178	-1.097235	0.491731
H	-4.583048	2.496141	-0.091088	H	-4.583674	2.502537	-0.081505	C	-4.350901	2.096696	0.924561
H	-5.107600	2.381252	1.592206	H	-5.106927	2.395962	1.602740	H	-4.599725	2.510438	-0.048884
H	-3.384779	2.469914	1.208671	H	-3.384647	2.488704	1.217745	H	-5.106610	2.423013	1.641458
C	-4.006076	0.030288	2.243501	C	-3.996304	0.053136	2.267764	H	-3.387796	2.502304	1.238389
H	-3.988674	-1.059385	2.293388	H	-3.974811	-1.036140	2.324285	C	-4.002409	0.080437	2.317959
H	-3.058693	0.410453	2.629238	H	-3.050147	0.439175	2.650666	H	-3.986408	-1.008349	2.384787
H	-4.799093	0.380153	2.904694	H	-4.790280	0.404069	2.927238	H	-3.050319	0.464777	2.687636

SPLET (ETE step) mechanism

M06-2X/6-311++G(2d,2p)											
SW (O1•)				SW (O2•)				SW (C1•)			
C	2.514260	0.976316	-0.103447	C	2.724847	0.870742	0.038151	C	2.763682	0.793255	0.028629
C	1.390528	0.154277	-0.057745	C	1.534889	0.162025	0.013995	C	1.484601	0.207955	0.045441
C	1.427106	-1.233126	-0.173165	C	1.428115	-1.193857	-0.312100	C	1.402624	-1.156971	-0.288706
C	2.660247	-1.847764	-0.398228	C	2.606632	-1.871308	-0.655764	C	2.511776	-1.931094	-0.650501
C	3.797525	-1.048729	-0.463942	C	3.813119	-1.195338	-0.645364	C	3.757964	-1.311510	-0.664770
C	3.737792	0.329013	-0.308781	C	3.962435	0.189163	-0.300818	C	3.884103	0.029292	-0.326416
H	0.412892	0.598700	0.061096	H	0.615784	0.682770	0.255449	H	4.644004	-1.876821	-0.944030
H	4.763981	-1.511743	-0.642564	H	4.726050	-1.715552	-0.913133	C	0.038937	-1.854517	-0.192768
C	0.138947	-2.034367	-0.001310	C	0.093202	-1.915337	-0.225043	H	0.027299	-2.703435	-0.887133
H	0.251397	-2.951051	-0.588072	H	0.112995	-2.737957	-0.948952	C	-1.112216	-0.962818	-0.631548
C	-1.075517	-1.293456	-0.534349	C	-1.069371	-1.029498	-0.655612	C	-1.138754	-0.440432	-1.932327
C	-1.198732	-1.053716	-1.915124	C	-1.098921	-0.501982	-1.956262	C	-2.186101	-0.672034	0.203107
C	-2.056555	-0.743243	0.296857	C	-2.122887	-0.715311	0.197660	C	-2.219941	0.341583	-2.312918
C	-2.245306	-0.287890	-2.389527	C	-2.168828	0.304546	-2.317754	C	-3.278081	0.124624	-0.151902
C	-3.115893	0.037035	-0.144674	C	-3.203798	0.103720	-0.141162	H	-2.163583	-1.072121	1.202365
H	-1.985067	-0.939235	1.357112	H	-2.098949	-1.126333	1.192810	C	-3.265823	0.633247	-1.448796
C	-3.250758	0.313155	-1.561014	C	-3.197365	0.614937	-1.438104	H	-2.249283	0.748559	-3.319972
H	-2.346443	-0.112246	-3.454928	H	-2.205375	0.712495	-3.323752	C	-0.147839	-2.460119	1.215395
C	0.005630	-2.497315	1.470688	C	-0.106559	-2.581408	1.154887	H	-1.127552	-2.943270	1.272955
H	-0.982648	-2.946349	1.578593	H	-1.103369	-3.030392	1.195654	H	0.588428	-3.260345	1.329569
H	0.730094	-3.297330	1.650739	H	0.599642	-3.412100	1.222251	C	0.037292	-1.459717	2.375121
C	0.217897	-1.416645	2.548748	C	0.123836	-1.664409	2.370930	H	-0.736255	-1.623926	3.131835
H	-0.490628	-1.580924	3.363937	H	-0.574540	-1.940528	3.166609	H	-0.077570	-0.445273	1.988370
H	-0.016837	-0.433674	2.139891	H	-0.102672	-0.630396	2.103837	C	1.413760	-1.560970	3.028976
C	1.635495	-1.395513	3.118854	C	1.551552	-1.726039	2.913129	H	1.528047	-0.821183	3.823383
H	1.741130	-0.627887	3.887099	H	1.669411	-1.072375	3.778763	H	1.570804	-2.552174	3.463137
H	1.882720	-2.358358	3.571907	H	1.799512	-2.743602	3.223977	H	2.193109	-1.380121	2.287822
H	2.371098	-1.193035	2.339324	H	2.270067	-1.416252	2.153964	C	2.422525	-3.387644	-1.049764
C	2.782339	-3.336260	-0.596674	C	2.583299	-3.327251	-1.062681	H	1.927300	-3.994936	-0.290334
H	2.377789	-3.890570	0.250484	H	2.122166	-3.962784	-0.303780	H	1.855509	-3.513525	-1.976555
H	2.229074	-3.652939	-1.483267	H	2.020291	-3.476357	-1.988893	H	3.417425	-3.804203	-1.212182
H	3.824532	-3.625141	-0.725719	H	3.597061	-3.687685	-1.230129	C	-0.024472	-0.675345	-2.914507
C	-0.202681	-1.633296	-2.893155	C	-0.010139	-0.771389	-2.959871	H	0.843675	-0.074349	-2.639333
H	0.804022	-1.240560	-2.729930	H	0.892448	-0.213128	-2.707814	H	0.302232	-1.716262	-2.910568
H	-0.140493	-2.721788	-2.803079	H	0.271396	-1.825051	-2.969008	H	-0.341409	-0.414319	-3.924514

H	-0.496584	-1.399279	-3.915328	H	-0.337272	-0.491836	-3.960997	O	-4.309056	1.430274	-1.886093
O	-4.178841	1.018521	-2.054931	O	-4.226932	1.432951	-1.856097	H	-4.082857	1.749843	-2.761542
O	4.897614	1.072218	-0.373414	H	-4.020549	1.739519	-2.741310	O	5.157760	0.595508	-0.358869
H	5.628440	0.471955	-0.533377	O	5.094259	0.753544	-0.307318	H	5.769446	-0.093911	-0.625531
C	2.409049	2.494578	0.059786	C	2.773789	2.351755	0.410700	C	2.942110	2.279913	0.404138
C	3.223748	2.945725	1.283812	C	3.668470	2.540705	1.647654	C	3.862730	2.420168	1.628365
H	4.278987	2.708222	1.174183	H	4.660666	2.140939	1.450169	H	4.859066	2.030181	1.432355
H	3.122462	4.025320	1.415577	H	3.745690	3.602724	1.901281	H	3.950818	3.471983	1.917421
H	2.848766	2.456577	2.184521	H	3.237156	2.014608	2.502661	H	3.436078	1.870902	2.470528
C	0.958319	2.936457	0.278639	C	1.391996	2.922739	0.741653	C	1.597986	2.913805	0.767663
H	0.317233	2.667274	-0.561000	H	0.707248	2.841104	-0.105017	H	0.891369	2.829841	-0.055928
H	0.534711	2.496662	1.182620	H	0.939824	2.413558	1.595569	H	1.142805	2.401480	1.614049
H	0.931950	4.021481	0.389051	H	1.491607	3.980916	0.994426	H	1.755520	3.968422	1.016824
C	2.926638	3.199989	-1.204427	C	3.348831	3.164015	-0.762072	C	3.521264	3.072233	-0.779873
H	3.968458	2.958048	-1.400330	H	4.330235	2.780412	-1.031442	H	4.496387	2.694725	-1.080061
H	2.330821	2.904984	-2.069042	H	2.686847	3.088844	-1.627849	H	2.844270	3.000394	-1.633639
H	2.838251	4.282057	-1.083988	H	3.433090	4.219878	-0.486111	H	3.623637	4.129228	-0.515252
C	-4.143741	0.621467	0.823760	C	-4.322375	0.426547	0.853867	C	-4.411900	0.434116	0.830204
C	-5.549438	0.111904	0.461788	C	-5.674259	-0.061506	0.306107	C	-5.752067	-0.074230	0.272345
H	-6.297753	0.561585	1.122287	H	-6.467064	0.158289	1.025084	H	-6.555987	0.139802	0.981269
H	-5.776683	0.365196	-0.571686	H	-5.918683	0.421243	-0.636649	H	-5.991503	0.399374	-0.676505
H	-5.596563	-0.972994	0.579963	H	-5.648310	-1.140520	0.146148	H	-5.710065	-1.153882	0.119198
C	-4.123774	2.156982	0.728846	C	-4.379158	1.941976	1.109150	C	-4.492175	1.949650	1.078863
H	-4.318560	2.463254	-0.296640	H	-4.588329	2.491318	0.194903	H	-4.702233	2.490770	0.159769
H	-4.881647	2.588452	1.390494	H	-5.163570	2.164971	1.836277	H	-5.284607	2.165772	1.799996
H	-3.146049	2.537911	1.033552	H	-3.428355	2.292204	1.513201	H	-3.547727	2.313218	1.485555
C	-3.868961	0.240039	2.281530	C	-4.095082	-0.260131	2.204534	C	-4.188226	-0.242751	2.186475
H	-3.900808	-0.841755	2.427238	H	-4.070976	-1.346106	2.105168	H	-4.145763	-1.328671	2.091533
H	-2.894493	0.601590	2.617931	H	-3.162740	0.063766	2.668594	H	-3.264441	0.096993	2.655909
H	-4.632708	0.687969	2.921653	H	-4.914668	-0.001195	2.876510	H	-5.018576	0.006849	2.849384

SW (C2·)				SW (C3·)				SW (C4·)			
C	2.731887	0.839133	-0.004765	C	-3.063189	-0.635226	0.267214	C	2.437371	0.974437	-0.367882
C	1.512412	0.156305	-0.003432	C	-1.755564	-0.199520	0.099826	C	1.351413	0.156603	-0.068320
C	1.424812	-1.201024	-0.287173	C	-1.354306	1.008782	-0.526236	C	1.421756	-1.231266	0.036158
C	2.598932	-1.907384	-0.617472	C	-2.416618	1.807995	-1.028731	C	2.646307	-1.854446	-0.209299
C	3.853871	-1.291233	-0.644201	C	-3.731803	1.397716	-0.838050	C	3.744076	-1.060729	-0.531542
C	3.856432	0.064586	-0.321735	C	-4.071780	0.214919	-0.204590	C	3.653074	0.321639	-0.601617
H	0.600591	0.692250	0.221194	H	-0.954575	-0.809051	0.491338	H	0.379989	0.593881	0.116428
C	0.079081	-1.910020	-0.163723	H	-4.532676	2.014742	-1.240622	H	4.704323	-1.531015	-0.725079
H	0.094077	-2.761564	-0.850607	C	0.051186	1.366012	-0.586310	C	0.188299	-2.000741	0.484821
C	-1.086142	-1.042154	-0.617350	C	1.075769	0.360792	-0.661502	H	0.295289	-3.023344	0.116123
C	-1.132110	-0.578278	-1.941789	C	0.963998	-0.845308	-1.427723	C	-1.091902	-1.417837	-0.129080
C	-2.129562	-0.687923	0.232614	C	2.346473	0.549239	-0.041634	C	-1.389696	-1.790794	-1.447021
C	-2.209977	0.204681	-2.330557	C	2.018448	-1.747433	-1.439238	C	-1.867750	-0.506531	0.608629
C	-3.217456	0.110080	-0.133611	C	3.412377	-0.343813	-0.074011	C	-2.531340	-1.254277	-2.031732
H	-2.092711	-1.050586	1.246236	H	2.483438	1.455527	0.521684	C	-3.005737	0.027620	-0.024997
C	-3.230688	0.552423	-1.455750	C	3.216211	-1.536464	-0.774561	C	-3.331569	-0.362148	-1.329668
H	-2.259940	0.562378	-3.355009	H	1.919812	-2.648207	-2.042268	H	-2.802578	-1.527285	-3.049007
C	-0.098398	-2.514683	1.246499	C	0.471029	2.752028	-0.147961	C	0.124747	-2.123613	2.029117
H	-1.092415	-2.965334	1.315654	H	1.475205	2.962901	-0.529748	H	-0.900730	-2.410606	2.260582
H	0.613968	-3.336862	1.339651	H	-0.166613	3.524836	-0.583200	H	0.779017	-2.946192	2.343906
C	0.138622	-1.547128	2.421992	C	0.470427	3.007938	1.376573	C	0.483483	-0.884283	2.860896
H	-0.556612	-1.787794	3.231982	H	0.982711	3.956674	1.580181	H	-0.078531	-0.934722	3.797216
H	-0.086822	-0.524649	2.113803	H	1.040576	2.224500	1.881698	H	0.110751	0.000010	2.346516
C	1.569465	-1.592335	2.958008	C	-0.935193	3.060463	1.972002	C	1.970446	-0.754086	3.196290
H	1.706887	-0.874836	3.768700	H	-0.909694	3.309174	3.035079	H	2.149792	0.103242	3.848652
H	1.800513	-2.586930	3.346020	H	-1.539829	3.816360	1.463730	H	2.329981	-1.645925	3.717659
H	2.288089	-1.361462	2.171167	H	-1.444733	2.104957	1.855316	H	2.582922	-0.622886	2.303645

C	2.512685	-3.378979	-0.983253	C	-2.168551	3.038453	-1.855559	C	2.803999	-3.350248	-0.120625
H	2.034789	-3.987886	-0.210102	H	-2.052279	3.942814	-1.251213	H	2.508866	-3.719127	0.862554
H	1.946129	-3.538504	-1.908278	H	-1.246961	2.914262	-2.429243	H	2.173753	-3.854765	-0.856123
H	3.522854	-3.749214	-1.138812	H	-2.997909	3.208934	-2.543696	H	3.838070	-3.643039	-0.299930
C	-0.051499	-0.894992	-2.939924	C	-0.200615	-1.118875	-2.338122	C	-0.523367	-2.732323	-2.252705
H	0.857894	-0.337203	-2.712757	H	-1.059498	-1.560330	-1.825575	H	0.515276	-2.392512	-2.279130
H	0.223113	-1.950009	-2.909315	H	-0.557990	-0.182825	-2.773233	H	-0.521407	-3.741162	-1.830584
H	-0.379573	-0.651086	-3.949884	H	0.101116	-1.795568	-3.139057	H	-0.881317	-2.804051	-3.280477
O	-4.273210	1.338335	-1.903785	O	4.229880	-2.494805	-0.842961	O	-4.467117	0.138532	-1.967183
H	-4.086364	1.588690	-2.810847	H	3.880340	-3.237072	-1.338445	H	-4.487825	-0.240466	-2.848243
O	5.102427	0.687729	-0.332305	O	-5.411916	-0.142235	-0.067027	O	4.775788	1.063975	-0.912567
H	5.670451	-0.055041	-0.583486	H	-5.933502	0.567422	-0.445462	H	5.509357	0.456805	-1.026722
C	2.818330	2.332286	0.328486	C	-3.390252	-1.974081	0.936118	C	2.301499	2.497915	-0.423516
C	3.701443	2.539421	1.569965	C	-4.251668	-1.748739	2.189577	C	3.224123	3.143912	0.623530
H	4.700785	2.144987	1.404661	H	-5.191812	-1.261799	1.943331	H	4.269472	2.912365	0.433359
H	3.773047	3.604607	1.809815	H	-4.467830	-2.706126	2.671416	H	3.099029	4.229173	0.605562
H	3.263705	2.022862	2.426696	H	-3.713570	-1.121517	2.902145	H	2.964458	2.786850	1.621421
C	1.444219	2.936808	0.637493	C	-2.126131	-2.720961	1.372864	C	0.866884	2.941025	-0.116781
H	0.762061	2.841932	-0.208965	H	-1.467114	-2.927505	0.528501	H	0.153538	2.533595	-0.833661
H	0.981646	2.458367	1.502635	H	-1.560198	-2.155615	2.113427	H	0.550501	2.629099	0.879414
H	1.560682	3.999608	0.861337	H	-2.414063	-3.674007	1.821314	H	0.814089	4.030107	-0.165211
C	3.407785	3.107488	-0.860876	C	-4.138911	-2.878916	-0.056977	C	2.660816	3.013206	-1.826814
H	4.393199	2.728571	-1.119543	H	-5.065882	-2.419361	-0.391147	H	3.683378	2.760129	-2.097194
H	2.757494	3.004685	-1.731814	H	-3.512925	-3.071124	-0.930267	H	1.986681	2.581040	-2.567616
H	3.488133	4.170879	-0.616302	H	-4.372829	-3.837509	0.413895	H	2.551196	4.099788	-1.860268
C	-4.323726	0.481116	0.858344	C	4.738453	-0.045427	0.634168	C	-3.896764	1.050885	0.712141
C	-5.678359	-0.052792	0.363650	C	5.884068	-0.024157	-0.392242	C	-5.319898	0.495234	0.891230
H	-6.462365	0.201592	1.080868	H	6.832546	0.189317	0.108493	H	-5.940661	1.210571	1.439691
H	-5.942812	0.369798	-0.602384	H	5.969474	-0.977680	-0.908152	H	-5.793078	0.289360	-0.066688
H	-5.642154	-1.139127	0.268212	H	5.705604	0.755387	-1.134855	H	-5.282322	-0.433061	1.464663
C	-4.392427	2.008793	1.022568	C	5.020002	-1.114219	1.703541	C	-3.953281	2.380743	-0.058815
H	-4.612822	2.499912	0.078514	H	5.091413	-2.105152	1.262319	H	-4.383197	2.253768	-1.050020
H	-5.172900	2.269110	1.741458	H	5.958853	-0.891032	2.217761	H	-4.555235	3.110950	0.490870
H	-3.441286	2.389771	1.397108	H	4.217421	-1.121593	2.442748	H	-2.945121	2.786024	-0.168116
C	-4.066949	-0.118756	2.244763	C	4.727756	1.315599	1.337566	C	-3.341510	1.353705	2.105438
H	-4.037847	-1.208644	2.213884	H	4.553483	2.129146	0.632868	H	-3.269324	0.445861	2.701688
H	-3.127858	0.237771	2.669928	H	3.959509	1.363528	2.110307	H	-2.333223	1.760743	2.041126
H	-4.875467	0.177806	2.914667	H	5.697561	1.478141	1.812518	H	-3.997410	2.075133	2.603971
**SW (C5$^{\bullet}$)**				**SW (C6$^{\bullet}$)**				**SW (C7$^{\bullet}$)**			
C	2.516206	0.977223	-0.126211	C	2.664462	0.864328	-0.120639	C	2.714668	0.761754	-0.334694
C	1.390233	0.159299	-0.062070	C	1.467485	0.148218	-0.076341	C	1.503173	0.105745	-0.118147
C	1.422445	-1.228473	-0.165840	C	1.368408	-1.239019	-0.210861	C	1.391561	-1.269162	0.090392
C	2.650483	-1.848920	-0.401253	C	2.549089	-1.950800	-0.471509	C	2.543825	-2.052103	0.013255
C	3.789744	-1.054903	-0.480268	C	3.747300	-1.256269	-0.552215	C	3.755841	-1.427618	-0.259839
C	3.735742	0.324241	-0.333762	C	3.819388	0.119330	-0.367104	C	3.850076	-0.049953	-0.404154
H	0.415777	0.608564	0.061510	H	0.545886	0.684892	0.094116	H	0.591817	0.678851	-0.067882
H	4.752860	-1.522196	-0.665285	H	4.665524	-1.801999	-0.754714	H	4.662293	-2.022983	-0.322379
C	0.140794	-2.026236	0.040454	C	0.049625	-1.954335	0.073976	C	0.071908	-1.838521	0.544769
H	0.236039	-2.950079	-0.535353	H	0.061649	-2.894665	-0.484712	H	0.060682	-2.912130	0.321905
C	-1.079435	-1.290640	-0.501099	C	-1.116085	-1.145657	-0.512760	C	-1.115975	-1.239967	-0.194377
C	-1.226511	-1.106759	-1.895277	C	-1.193845	-0.847230	-1.876969	C	-1.255497	-1.402253	-1.578578
C	-2.031991	-0.738735	0.346919	C	-2.134446	-0.694954	0.318770	C	-2.106474	-0.533145	0.487577
C	-2.271759	-0.360197	-2.442952	C	-2.286317	-0.117809	-2.337447	C	-2.362889	-0.844968	-2.207464
C	-3.106510	0.022252	-0.130263	C	-3.227341	0.058203	-0.112986	C	-3.222130	0.041122	-0.124840
H	-1.944312	-0.900705	1.409585	H	-2.038593	-1.012168	1.348529	H	-1.981321	-0.414929	1.553919
C	-3.158813	0.188981	-1.518089	C	-3.279158	0.336230	-1.480968	C	-3.324135	-0.130702	-1.506894
C	0.033359	-2.464555	1.522664	H	-2.373493	0.100144	-3.398913	H	-2.484357	-0.969996	-3.280087
H	-0.955709	-2.904812	1.657473	C	-0.089995	-2.332072	1.543829	C	-0.057090	-1.683284	2.113850

H	0.754816	-3.268375	1.699173	H	0.390614	-3.290855	1.751935	H	-1.014810	-2.173343	2.347003
C	0.275567	-1.373999	2.584341	C	0.286897	-1.282309	2.572617	H	0.705439	-2.344307	2.545548
H	-0.397477	-1.544327	3.428280	H	-0.315406	-1.411251	3.484233	C	0.101457	-0.314230	2.738915
H	0.011539	-0.396741	2.179113	H	0.017274	-0.283219	2.204471	H	-0.223846	0.496524	2.081521
C	1.713722	-1.328727	3.100186	C	1.761530	-1.231133	3.037275	C	1.513274	-0.051655	3.237625
H	1.832075	-0.564088	3.869723	H	1.918005	-0.490417	3.832223	H	1.613055	0.980938	3.588409
H	1.996733	-2.289164	3.537201	H	2.049426	-2.208471	3.432206	H	1.762201	-0.685378	4.098693
H	2.415873	-1.106145	2.295842	H	2.438163	-0.995295	2.213908	H	2.338692	-0.212194	2.505086
C	2.760763	-3.337556	-0.601904	C	2.538928	-3.447717	-0.611031	C	2.509699	-3.542141	0.235901
H	2.363481	-3.890353	0.249797	H	2.113150	-3.898682	0.288011	H	2.082971	-3.781819	1.210542
H	2.192781	-3.645923	-1.482129	H	1.922723	-3.767645	-1.454141	H	1.897972	-4.042807	-0.517781
H	3.799147	-3.633302	-0.745416	H	3.546968	-3.834298	-0.760171	H	3.514023	-3.961591	0.189201
C	-0.228868	-1.755038	-2.838293	C	-0.140644	-1.292991	-2.858121	C	-0.246997	-2.163381	-2.398670
H	0.790910	-1.386198	-2.689582	H	0.753939	-0.671979	-2.784119	H	0.702810	-1.629037	-2.447457
H	-0.196394	-2.844142	-2.716619	H	0.174060	-2.318125	-2.658982	H	-0.036811	-3.137555	-1.954084
H	-0.532255	-1.533958	-3.858588	H	-0.518520	-1.238698	-3.879512	H	-0.613730	-2.319833	-3.412960
O	-4.188400	0.961649	-2.052507	O	-4.338988	1.065629	-1.994051	O	-4.396017	0.413140	-2.188893
H	-3.987228	0.893557	-2.997260	H	-4.184292	1.180177	-2.933520	H	-4.283740	0.211843	-3.119835
O	4.898331	1.062610	-0.411087	O	5.049418	0.755944	-0.446571	O	5.087977	0.524212	-0.629625
H	5.623729	0.458778	-0.581945	H	5.719871	0.077372	-0.544411	H	5.746024	-0.171048	-0.573378
C	2.416447	2.497837	0.016719	C	2.711466	2.377283	0.111376	C	2.798742	2.286760	-0.433153
C	3.240118	2.964751	1.228621	C	3.580279	2.685102	1.342868	C	3.663687	2.823158	0.720282
H	4.293939	2.722606	1.115408	H	4.600337	2.334509	1.206647	H	4.682138	2.447503	0.659234
H	3.142878	4.046563	1.345478	H	3.601121	3.763149	1.522512	H	3.691837	3.915315	0.685992
H	2.870022	2.489528	2.138858	H	3.161697	2.197276	2.224424	H	3.237216	2.514842	1.675860
C	0.967972	2.946263	0.238035	C	1.316911	2.952767	0.379274	C	1.415471	2.932928	-0.307656
H	0.322206	2.664204	-0.593859	H	0.643566	2.792186	-0.463434	H	0.746752	2.610251	-1.106779
H	0.550106	2.521428	1.151985	H	0.864545	2.507570	1.266095	H	0.949718	2.691664	0.648590
H	0.944787	4.033092	0.331591	H	1.402685	4.028338	0.545335	H	1.523421	4.017116	-0.373642
C	2.926532	3.183402	-1.261359	C	3.280920	3.092806	-1.124395	C	3.396194	2.712132	-1.784274
H	3.966681	2.936634	-1.459950	H	4.293959	2.764409	-1.343322	H	4.394774	2.305660	-1.925743
H	2.324855	2.875755	-2.117334	H	2.655481	2.890707	-1.995597	H	2.761786	2.365079	-2.601851
H	2.841040	4.267463	-1.156579	H	3.293478	4.172579	-0.955720	H	3.453179	3.802376	-1.837252
C	-4.139302	0.639170	0.819693	C	-4.305573	0.546083	0.858315	C	-4.260943	0.828763	0.678908
C	-5.546301	0.117365	0.484596	C	-5.674833	-0.037532	0.472151	C	-5.651220	0.188871	0.526698
H	-6.285148	0.564769	1.156414	H	-6.436017	0.305289	1.177757	H	-6.378625	0.745089	1.122726
H	-5.811494	0.354944	-0.542611	H	-5.970149	0.264664	-0.529841	H	-5.983183	0.185678	-0.508757
H	-5.582357	-0.966640	0.608307	H	-5.640351	-1.127281	0.509575	H	-5.631406	-0.840153	0.888740
C	-4.116708	2.171654	0.693927	C	-4.372686	2.082362	0.846766	C	-4.302263	2.285991	0.188599
H	-4.331370	2.477250	-0.327025	H	-4.623364	2.463724	-0.140345	H	-4.572992	2.347405	-0.863172
H	-4.858018	2.617595	1.364006	H	-5.129472	2.427080	1.556170	H	-5.035130	2.850665	0.769953
H	-3.131486	2.554006	0.968797	H	-3.410067	2.500406	1.145687	H	-3.326240	2.753732	0.325208
C	-3.859471	0.293920	2.286692	C	-4.001654	0.104502	2.293608	C	-3.918144	0.848117	2.172117
H	-3.885857	-0.783861	2.455695	H	-3.960874	-0.981418	2.380354	H	-3.902042	-0.156644	2.595336
H	-2.886075	0.666945	2.610717	H	-3.048440	0.502213	2.643196	H	-2.943757	1.295823	2.369210
H	-4.623310	0.753524	2.918087	H	-4.789196	0.472873	2.954185	H	-4.677401	1.427033	2.701608
**SW (C8·)**				**SW (C9·)**				**SW (C10·)**			
C	-2.460222	1.085522	-0.092164	C	-2.708173	0.847136	-0.168381	C	2.709362	0.879913	-0.010148
C	-1.366459	0.242873	0.105889	C	-1.516972	0.122795	-0.013130	C	1.513650	0.166775	-0.058853
C	-1.460431	-1.107020	0.432082	C	-1.420468	-1.167001	0.468372	C	1.425395	-1.197133	-0.315970
C	-2.735458	-1.670779	0.563609	C	-2.602131	-1.895742	0.873363	C	2.600424	-1.897513	-0.594532
C	-3.839544	-0.840382	0.403681	C	-3.818295	-1.149681	0.702583	C	3.807796	-1.208297	-0.559965
C	-3.717087	0.500880	0.079002	C	-3.861409	0.132060	0.212140	C	3.871782	0.145299	-0.264024
H	-0.377119	0.656080	-0.010272	H	-0.587606	0.613104	-0.274954	H	0.575655	0.682239	0.088417
H	-4.834376	-1.264922	0.502051	H	-4.754745	-1.630397	0.974640	H	4.730823	-1.738613	-0.777135
C	-0.205016	-1.959232	0.621915	C	-0.076226	-1.860591	0.563708	C	0.075285	-1.902963	-0.223130
H	-0.366493	-2.540336	1.535656	H	-0.120057	-2.502127	1.451886	H	0.104783	-2.737565	-0.934820
C	1.007241	-1.083521	0.896047	C	1.067294	-0.893801	0.824742	C	-1.088112	-1.034588	-0.650468
C	1.173197	-0.471213	2.143207	C	1.060813	-0.112443	1.991443	C	-1.140019	-0.634934	-2.044480

C	1.944652	-0.815111	-0.097858	C	2.139223	-0.748539	-0.050708	C	-2.104034	-0.675526	0.213881
C	2.260789	0.372571	2.331692	C	2.121083	0.756765	2.209737	C	-2.286606	0.167321	-2.372119
C	3.036457	0.040014	0.061034	C	3.206551	0.134152	0.139458	C	-3.227286	0.099454	-0.118981
H	1.788884	-1.288374	-1.054338	H	2.139801	-1.355107	-0.941374	H	-2.035120	-1.024130	1.233609
C	3.173327	0.632983	1.318795	C	3.169711	0.891443	1.309692	C	-3.260876	0.504976	-1.467105
H	2.401010	0.852136	3.296321	H	2.131060	1.359663	3.113794	H	-2.390141	0.515213	-3.397023
C	0.015611	-2.987637	-0.519207	C	0.155281	-2.811237	-0.631097	C	-0.068226	-2.525975	1.185293
H	1.063194	-3.302237	-0.459328	H	1.146316	-3.265072	-0.541259	H	-1.060827	-2.974249	1.247982
H	-0.582244	-3.876228	-0.319969	H	-0.569108	-3.622013	-0.533574	H	0.645924	-3.349087	1.278515
C	-0.359612	-2.526175	-1.950613	C	-0.027417	-2.161218	-2.015831	C	0.159927	-1.565919	2.370331
H	0.385363	-2.956577	-2.629471	H	0.726329	-2.547495	-2.709118	H	-0.572005	-1.777076	3.154248
H	-0.190847	-1.428232	-1.988476	H	0.138543	-1.083896	-1.942066	H	-0.025895	-0.539220	2.052451
C	-1.743593	-2.972706	-2.417077	C	-1.419556	-2.399939	-2.598465	C	1.564405	-1.655971	2.966292
H	-1.779966	-2.990041	-3.511994	H	-1.533198	-1.913200	-3.568985	H	1.685571	-0.960633	3.798757
H	-2.522492	-2.275690	-2.079348	H	-1.601086	-3.468477	-2.730422	H	1.761558	-2.663585	3.339776
C	-2.942032	-3.142045	0.759343	H	-2.183223	-2.011597	-1.924412	H	2.322340	-1.420614	2.218067
H	-2.753845	-3.637950	-0.206997	C	-2.604169	-3.183616	1.379313	C	2.588251	-3.355796	-0.973586
H	-2.254010	-3.562774	1.493817	H	-1.712531	-3.784230	1.461544	H	2.105552	-3.969810	-0.213428
H	-3.962967	-3.353466	1.077516	H	-3.536397	-3.645385	1.673895	H	2.035317	-3.502851	-1.903508
C	0.197796	-0.694665	3.268468	C	-0.062381	-0.168432	2.993095	H	3.602935	-3.726336	-1.115684
H	-0.818048	-0.446919	2.957406	H	-0.887445	0.470013	2.671697	C	-0.204451	-0.988493	-2.996466
H	0.191528	-1.740667	3.580719	H	-0.477688	-1.172038	3.088324	H	-0.325867	-0.659700	-4.019582
H	0.457505	-0.085146	4.133017	H	0.284297	0.167427	3.970950	H	0.697397	-1.528841	-2.757251
O	4.229935	1.487729	1.558991	O	4.189465	1.783401	1.580795	O	-4.335657	1.279521	-1.893891
H	4.150999	1.810946	2.458618	H	3.976492	2.231211	2.401884	H	-4.191003	1.457637	-2.825332
O	-4.853605	1.269103	-0.083399	O	-5.094702	0.761834	0.085350	O	5.099284	0.779198	-0.236698
H	-5.611409	0.683818	-0.024595	H	-5.755992	0.133430	0.382164	H	5.768851	0.134036	-0.471388
C	-2.290591	2.554332	-0.487935	C	-2.755989	2.277901	-0.697730	C	2.744267	2.381302	0.285701
C	-2.954672	2.809982	-1.851696	C	-3.576590	2.354338	-1.998706	C	3.573753	2.648989	1.552658
H	-4.023400	2.615165	-1.814862	H	-4.597136	2.015837	-1.834912	H	4.598710	2.303804	1.440662
H	-2.799708	3.849707	-2.150795	H	-3.604486	3.381377	-2.376593	H	3.587394	3.720555	1.766006
H	-2.512960	2.162506	-2.609996	H	-3.122681	1.718241	-2.760551	H	3.126935	2.137064	2.406897
C	-0.812213	2.937605	-0.616804	C	-1.352363	2.805040	-1.014303	C	1.337464	2.938427	0.525588
H	-0.273834	2.797726	0.321833	H	-0.715819	2.805210	-0.127743	H	0.695295	2.805129	-0.345191
H	-0.311816	2.350892	-1.388047	H	-0.863963	2.204164	-1.783087	H	0.855462	2.459305	1.378700
H	-0.741265	3.990980	-0.893584	H	-1.425663	3.831108	-1.381584	H	1.410433	4.006994	0.734966
C	-2.916632	3.470040	0.577143	C	-3.373904	3.229398	0.344167	C	3.347095	3.138883	-0.908856
H	-3.978670	3.270884	0.698397	H	-4.380301	2.915366	0.611345	H	4.372506	2.831813	-1.099746
H	-2.424353	3.318814	1.539364	H	-2.763618	3.234258	1.249136	H	2.755647	2.951224	-1.805880
H	-2.789199	4.516549	0.288901	H	-3.417765	4.250623	-0.047637	H	3.337467	4.212969	-0.708659
C	4.013097	0.323061	-1.083752	C	4.338768	0.270894	-0.882103	C	-4.316036	0.469068	0.884837
C	5.435282	-0.107415	-0.688061	C	5.685969	-0.093137	-0.236846	C	-5.680680	-0.096973	0.449448
H	6.122675	0.085888	-1.514799	H	6.487312	-0.000827	-0.974012	H	-6.457808	0.170814	1.172744
H	5.787403	0.431667	0.187784	H	5.913188	0.556392	0.604614	H	-5.966503	0.287727	-0.526831
H	5.457051	-1.176217	-0.470249	H	5.665770	-1.124700	0.118098	H	-5.627526	-1.185142	0.386708
C	3.995753	1.822256	-1.426998	C	4.388657	1.710570	-1.420960	C	-4.431645	1.997738	1.035159
H	4.293368	2.430413	-0.576518	H	4.569288	2.427916	-0.624659	H	-4.661683	2.464736	0.080549
H	4.682261	2.020039	-2.253398	H	5.187474	1.802820	-2.160755	H	-5.219137	2.255154	1.750903
H	2.994024	2.123899	-1.736285	H	3.443966	1.962379	-1.904922	H	-3.487536	2.407086	1.398865
C	3.631420	-0.445801	-2.353134	C	4.135343	-0.660552	-2.081694	C	-4.014644	-0.098472	2.276595
H	3.651820	-1.524344	-2.194111	H	4.126867	-1.709110	-1.781216	H	-3.959148	-1.188174	2.259814
H	2.636434	-0.175725	-2.708051	H	3.202525	-0.447259	-2.605064	H	-3.071119	0.284966	2.669344
H	4.348503	-0.207413	-3.140288	H	4.957280	-0.518846	-2.785371	H	-4.811696	0.189863	2.965591

**SW (C11·)**				**SW (C12·)**			
C	2.701146	0.897096	0.035187	C	2.645964	0.885458	-0.067446
C	1.509851	0.178572	0.000729	C	1.462893	0.151440	-0.032965
C	1.424735	-1.176158	-0.309325	C	1.391019	-1.228132	-0.213059
C	2.596866	-1.861528	-0.635481	C	2.568980	-1.926482	-0.483103

C	3.798797	-1.162859	-0.612555	C	3.761954	-1.213999	-0.543756
C	3.863497	0.182697	-0.277845	C	3.810908	0.156501	-0.333813
H	0.591372	0.705970	0.222858	H	0.530266	0.667395	0.140434
H	4.718288	-1.681702	-0.863282	H	4.686719	-1.741506	-0.757921
C	0.088360	-1.900688	-0.230013	C	0.058408	-1.944977	-0.046696
H	0.123686	-2.727181	-0.945172	H	0.096028	-2.850203	-0.659986
C	-1.077600	-1.020039	-0.661422	C	-1.113296	-1.119137	-0.562287
C	-1.126534	-0.522173	-1.970150	C	-1.185376	-0.795836	-1.922303
C	-2.118505	-0.695400	0.204401	C	-2.128260	-0.668396	0.278987
C	-2.201896	0.277597	-2.332144	C	-2.263507	-0.034168	-2.354832
C	-3.205440	0.110889	-0.133842	C	-3.216772	0.102415	-0.119446
H	-2.086458	-1.092969	1.204625	H	-2.129240	-0.902959	1.335328
C	-3.218619	0.600030	-1.443011	C	-3.253992	0.412436	-1.483822
H	-2.256918	0.661236	-3.345896	H	-2.340738	0.216323	-3.410135
C	-0.088729	-2.549680	1.160711	C	-0.110511	-2.428083	1.412715
H	-1.077158	-3.010763	1.194211	H	-1.107399	-2.860509	1.502091
H	0.626615	-3.370623	1.242727	H	0.598910	-3.242894	1.584785
C	0.115581	-1.617450	2.370522	C	0.103763	-1.364900	2.505676
H	-0.607125	-1.877956	3.146911	H	-0.634445	-1.512758	3.294884
H	-0.099697	-0.584792	2.090695	H	-0.100416	-0.371448	2.106362
C	1.522606	-1.689600	2.961897	C	1.507069	-1.393149	3.109192
H	1.620163	-1.025397	3.820839	H	1.619334	-0.627492	3.877996
H	1.746831	-2.703835	3.296563	H	1.706913	-2.362173	3.572274
H	2.275758	-1.402910	2.227470	H	2.271627	-1.221703	2.349524
C	2.594210	-3.316622	-1.027478	C	2.577782	-3.413210	-0.727369
H	2.121088	-3.940740	-0.269995	H	2.121364	-3.958640	0.098500
H	2.049060	-3.470904	-1.960883	H	2.014806	-3.663778	-1.628800
H	3.610083	-3.678260	-1.172655	H	3.595794	-3.778341	-0.853985
C	-0.059279	-0.821537	-2.989919	C	-0.145817	-1.245032	-2.918321
H	0.851867	-0.258069	-2.784281	H	0.794710	-0.705253	-2.791609
H	0.212790	-1.877832	-2.987608	H	0.081548	-2.307745	-2.808762
H	-0.404007	-0.564955	-3.989781	H	-0.497036	-1.082302	-3.936704
O	-4.254186	1.399874	-1.851193	O	-4.290437	1.164487	-1.993613
H	-4.109776	1.654505	-2.765275	H	-4.137066	1.283008	-2.932829
O	5.071664	0.828769	-0.258629	O	5.020270	0.812302	-0.396289
H	5.763321	0.204876	-0.489999	H	5.703799	0.166332	-0.585295
C	2.725669	2.391036	0.401422	C	2.667375	2.395264	0.184738
C	3.569896	2.599797	1.671817	C	3.536500	2.702294	1.416131
H	4.595826	2.273634	1.523509	H	4.563906	2.376107	1.274130
H	3.572065	3.657655	1.938937	H	3.535663	3.777499	1.607079
H	3.141491	2.037225	2.502774	H	3.130072	2.200541	2.295660
C	1.346044	2.909647	0.674175	C	1.262388	2.939352	0.462486
H	0.696568	3.193111	-0.141962	H	0.587986	2.777537	-0.378848
H	0.925420	2.858564	1.668358	H	0.817053	2.482456	1.346798
C	3.308565	3.209977	-0.766524	H	1.327930	4.013722	0.638598
H	4.327885	2.909282	-0.993680	C	3.219394	3.132861	-1.046233
H	2.697673	3.076297	-1.660386	H	4.239153	2.831541	-1.272699
H	3.304747	4.269217	-0.505471	H	2.594572	2.930057	-1.917327
C	-4.316603	0.437959	0.868148	H	3.208608	4.209501	-0.863940
C	-5.667146	-0.080596	0.345835	C	-4.276941	0.547099	0.953938
H	-6.449134	0.129906	1.077789	C	-5.638983	-0.010483	0.494070
H	-5.940044	0.391011	-0.594161	H	-6.403345	0.361032	1.176100
H	-5.625293	-1.159993	0.193853	H	-5.892790	0.266449	-0.531020
C	-4.389439	1.957193	1.094976	H	-5.627820	-1.100412	0.570641
H	-4.614945	2.489649	0.174912	C	-4.320010	2.088405	0.922672
H	-5.170221	2.181768	1.824099	H	-4.496654	2.493828	-0.075339
H	-3.441302	2.327996	1.487514	H	-5.108129	2.418197	1.598972
C	-4.062248	-0.219148	2.228926	H	-3.373451	2.483818	1.300022
H	-4.030057	-1.307090	2.153974	C	-4.035883	0.106535	2.380853

H	-3.128608	0.126471	2.675600	H	-4.035823	-0.987776	2.479959
H	-4.874392	0.044378	2.906637	H	-3.091381	0.503793	2.778247

Structure of L-ascorbic acid (Asc) and the corresponding radical species in the HAT mechanism optimized at the M05-2X/6-311++G(2d,2p) level of theory in gas phase.

Asc-HAT											
M05-2X/6-311++G(2d,2p)											
Asc				Asc (O1•)				Asc (O2•)			
C	0.26799200	0.20608800	-0.77718600	C	0.24372	0.30643	-0.88814	C	0.22043	0.22272	-0.81227
O	-0.17999700	-1.15426900	-0.64138500	O	-0.21485	-1.06211	-0.81627	O	-0.20671	-1.14278	-0.65919
C	-1.45256400	-1.15984800	-0.14929800	C	-1.39901	-1.12864	-0.17367	C	-1.46088	-1.23722	-0.10264
C	-1.89332800	0.21617900	0.02975700	C	-1.86489	0.22166	0.11448	C	-1.97965	0.17133	0.09599
C	-0.90029900	1.02254000	-0.32930600	C	-0.90214	1.16730	-0.35443	C	-0.94724	1.00623	-0.34775
O	-0.79793900	2.35777700	-0.32941000	O	-0.91528	2.37861	-0.34225	O	-0.97450	2.31586	-0.32589
H	-1.59258200	2.74597100	0.04828300	O	-3.00889	0.43661	0.71457	H	-1.82360	2.58567	0.05985
O	-3.11801100	0.52225500	0.50871300	H	-3.40489	-0.42808	0.90928	O	-3.06448	0.53081	0.55236
H	-3.57082700	-0.30977400	0.69128400	O	-1.99154	-2.13832	0.08952	O	-1.97489	-2.27419	0.14886
O	-2.07698400	-2.15359000	0.09191500	C	1.47209	0.48600	-0.02273	C	1.46032	0.45799	0.05031
C	1.50850900	0.40933800	0.08785300	H	1.78040	1.52980	-0.11144	H	1.73977	1.50606	-0.03307
H	1.78843900	1.45897900	0.03713300	C	2.60610	-0.41719	-0.45406	C	2.61058	-0.39786	-0.43714
C	2.65678300	-0.42941200	-0.43545400	H	2.27227	-1.45159	-0.41982	H	2.36663	-1.44927	-0.27871
H	2.42535100	-1.48453000	-0.28256200	H	2.92298	-0.16181	-1.46495	H	2.74690	-0.23184	-1.50728
H	2.76999300	-0.25126500	-1.50636700	O	1.08200	0.19291	1.30212	O	1.14453	0.23369	1.40357
O	1.21767000	0.14130300	1.43847100	H	1.88548	0.10458	1.82234	H	1.14644	-0.71107	1.57467
H	1.06982500	-0.80226800	1.53817900	O	3.65587	-0.18879	0.48117	O	3.76167	-0.01590	0.28943
O	3.82461500	-0.04784000	0.26657100	H	4.29417	-0.89944	0.43092	H	4.47711	-0.62089	0.09563
H	4.53223600	-0.65788100	0.06162400	H	0.45582	0.54184	-1.92596	H	0.44040	0.40239	-1.86329
H	0.50018200	0.38637800	-1.82476400								
Asc (O3•)				Asc (O4•)				Asc (C1•)			
C	0.25960	0.19879	-0.80422	C	0.31521	0.23151	-0.76821	C	0.19853	0.10620	0.15190
O	-0.18469	-1.15746	-0.66708	O	-0.10714	-1.13703	-0.64113	O	-0.27242	-1.17803	0.07802
C	-1.43610	-1.16534	-0.12802	C	-1.38472	-1.16836	-0.15625	C	-1.65501	-1.10939	-0.07311
C	-1.87185	0.21373	0.06862	C	-1.85018	0.19913	0.02669	C	-1.99708	0.28301	-0.09596
C	-0.89575	1.01979	-0.33132	C	-0.86963	1.02497	-0.32346	C	-0.83571	1.01710	0.04625
O	-0.78737	2.35770	-0.33975	O	-0.78940	2.36125	-0.31784	O	-0.67715	2.35752	0.07948
H	-1.57588	2.75120	0.04525	H	-1.59425	2.73617	0.05197	H	-1.53798	2.78141	0.04340
O	-3.07906	0.51471	0.58768	O	-3.08162	0.48313	0.49982	O	-3.24537	0.74984	-0.22942
H	-3.52178	-0.31982	0.78519	H	-3.52413	-0.35560	0.67694	H	-3.84177	-0.00616	-0.29616
O	-2.05883	-2.15500	0.12615	O	-1.98994	-2.17458	0.07700	O	-2.36938	-2.07460	-0.16581
C	1.50903	0.39609	0.05105	C	1.54762	0.44502	0.10494	C	1.65926	0.27362	0.35507
H	1.82146	1.44823	0.00737	H	1.81008	1.49955	0.06800	H	1.87414	1.33142	0.46880

C	2.70150	-0.44279	-0.43578	C	2.71396	-0.38175	-0.42446	C	2.42015	-0.25434	-0.85091
H	2.43703	-1.49639	-0.36875	H	2.48448	-1.45269	-0.32010	H	2.27872	-1.33500	-0.91256
H	2.90787	-0.19620	-1.47242	H	2.87134	-0.22158	-1.49725	H	2.01025	0.20342	-1.75234
O	1.28079	0.12484	1.37194	O	1.26883	0.15339	1.45097	O	2.07076	-0.34943	1.55777
O	3.85390	-0.13788	0.30818	H	1.07394	-0.78391	1.53188	H	1.87650	-1.28803	1.50022
H	3.64913	-0.26852	1.23689	O	3.88602	-0.17950	0.24952	O	3.78163	0.08292	-0.67721
H	0.48570	0.38449	-1.85241	H	0.55099	0.42177	-1.81367	H	4.29480	-0.29003	-1.39328
**Asc (C2$^{\bullet}$)**				**Asc (C3$^{\bullet}$)**							
C	0.25904	0.01733	-0.79449	C	0.31308	0.19451	-0.76174				
O	-0.33921	-1.30043	-0.55389	O	-0.13789	-1.16433	-0.61624				
C	-1.58308	-1.13530	-0.04239	C	-1.41876	-1.16355	-0.14875				
C	-1.87603	0.29006	0.05071	C	-1.86244	0.21446	0.00561				
C	-0.82364	0.97141	-0.38553	C	-0.86182	1.01631	-0.34207				
O	-0.60927	2.28938	-0.49380	O	-0.75801	2.35140	-0.35253				
H	-1.36755	2.76669	-0.14506	H	-1.55958	2.74353	0.00584				
O	-3.05638	0.74648	0.52214	O	-3.09610	0.52582	0.45782				
H	-3.58218	-0.02713	0.76043	H	-3.55289	-0.30452	0.63824				
O	-2.31860	-2.03045	0.26953	O	-2.04874	-2.15407	0.09288				
C	1.50885	0.17577	-0.03307	C	1.54175	0.40394	0.11738				
C	2.74343	-0.53694	-0.45555	H	1.83166	1.45037	0.05449				
H	2.63699	-1.61219	-0.27853	C	2.65588	-0.44460	-0.35621				
H	2.92177	-0.37429	-1.51720	H	2.49750	-1.47394	-0.63731				
O	1.35937	0.40608	1.28928	O	1.20579	0.18628	1.48645				
H	2.24342	0.42658	1.67648	H	1.05852	-0.75495	1.60893				
O	3.81256	-0.02172	0.33667	O	3.88616	-0.07247	0.08317				
H	4.52303	-0.66142	0.36707	H	4.53954	-0.72800	-0.16250				
H	0.46526	0.07299	-1.86065	H	0.56936	0.36005	-1.80541				

SET-PT (SET step) mechanism

**M05-2X/6-311++G(2d,2p)**			
**Asc$^{\bullet+}$**			
C	0.24285900	0.29269600	-0.99098800
O	-0.18156700	-1.08285600	-0.93477300
C	-1.29020500	-1.23071600	-0.19348700
C	-1.79106000	0.13053900	0.14519100
C	-0.89998800	1.05800100	-0.39709700
O	-0.91753500	2.34068000	-0.37246400
H	-1.67060800	2.71856000	0.10959900
O	-2.86023600	0.36058400	0.81917800
H	-3.28130100	-0.48043800	1.08685200
O	-1.83362300	-2.23365600	0.12671200
C	1.45154100	0.50665800	-0.08415400
H	1.77585400	1.54291700	-0.19601900
C	2.60647300	-0.42099600	-0.40568900
H	2.26674000	-1.45407000	-0.39059600
H	3.02095900	-0.17935300	-1.38372200
O	0.95802500	0.27339900	1.21803900
H	1.71663900	0.13855400	1.79941300
O	3.53150900	-0.16484500	0.63484300
H	4.24080100	-0.80938200	0.62394700
H	0.44061200	0.55967200	-2.02440400

SET-PT (PT step) mechanism

Asc_PT

M05-2X/6-311++G(2d,2p)

Asc$^{\bullet+}$

C	0.24285900	0.29269600	-0.99098800
O	-0.18156700	-1.08285600	-0.93477300
C	-1.29020500	-1.23071600	-0.19348700
C	-1.79106000	0.13053900	0.14519100
C	-0.89998800	1.05800100	-0.39709700
O	-0.91753500	2.34068000	-0.37246400
H	-1.67060800	2.71856000	0.10959900
O	-2.86023600	0.36058400	0.81917800
H	-3.28130100	-0.48043800	1.08685200
O	-1.83362300	-2.23365600	0.12671200
C	1.45154100	0.50665800	-0.08415400
H	1.77585400	1.54291700	-0.19601900
C	2.60647300	-0.42099600	-0.40568900
H	2.26674000	-1.45407000	-0.39059600
H	3.02095900	-0.17935300	-1.38372200
O	0.95802500	0.27339900	1.21803900
H	1.71663900	0.13855400	1.79941300
O	3.53150900	-0.16484500	0.63484300
H	4.24080100	-0.80938200	0.62394700
H	0.44061200	0.55967200	-2.02440400

Asc (O1$^{\bullet}$)

C	0.24374	0.30640	-0.88814
O	-0.21487	-1.06218	-0.81619
C	-1.39904	-1.12860	-0.17365
C	-1.86487	0.22171	0.11448
C	-0.90210	1.16731	-0.35448
O	-0.91514	2.37861	-0.34230
O	-3.00889	0.43664	0.71457
H	-3.40482	-0.42811	0.90928
O	-1.99170	-2.13822	0.08955
C	1.47212	0.48594	-0.02278
H	1.78053	1.52970	-0.11160
C	2.60608	-0.41736	-0.45403
H	2.27223	-1.45175	-0.41957
H	2.92293	-0.16217	-1.46497
O	1.08203	0.19302	1.30210
H	1.88549	0.10486	1.82236
O	3.65585	-0.18878	0.48114
H	4.29412	-0.89947	0.43107
H	0.45581	0.54170	-1.92598

Asc (O2$^{\bullet}$)

C	0.19412	0.21765	-0.88006
O	-0.20280	-1.15098	-0.70827
C	-1.41115	-1.26685	-0.06668
C	-1.95030	0.13286	0.14706
C	-0.96972	0.98515	-0.37469
O	-1.03015	2.29477	-0.37563
H	-1.86312	2.54797	0.05374
O	-3.01321	0.47597	0.66252
O	-1.89290	-2.31075	0.21697
C	1.45215	0.51201	-0.07896
H	1.75738	1.53631	-0.30667
C	2.57414	-0.43546	-0.44307
H	2.24934	-1.45735	-0.26585
H	2.84709	-0.30855	-1.49049
O	1.11365	0.38729	1.28280
H	1.93622	0.32728	1.77721
O	3.65768	-0.08612	0.41252
H	4.30220	-0.79300	0.42184
H	0.37726	0.39373	-1.93854

Asc (O3$^{\bullet}$)

C	0.25961	0.19873	-0.80395
O	-0.18493	-1.15746	-0.66698
C	-1.43642	-1.16511	-0.12814
C	-1.87188	0.21404	0.06879
C	-0.89557	1.01991	-0.33102
O	-0.78661	2.35778	-0.33935
H	-1.57593	2.75173	0.04353
O	-3.07917	0.51499	0.58763
H	-3.52228	-0.31959	0.78409
O	-2.05954	-2.15463	0.12565
C	1.50910	0.39574	0.05140
H	1.82074	1.44813	0.00886
C	2.70170	-0.44214	-0.43620
H	2.43758	-1.49595	-0.37068
H	2.90812	-0.19439	-1.47259
O	1.28086	0.12328	1.37212
O	3.85405	-0.13798	0.30804
H	3.64931	-0.26925	1.23670
H	0.48582	0.38460	-1.85208

Asc (O4$^{\bullet}$)

C	0.29093	0.23704	-0.84096
O	-0.08977	-1.13669	-0.69488
C	-1.32805	-1.20036	-0.12892
C	-1.82614	0.15626	0.06398
C	-0.89697	1.00638	-0.35577
O	-0.85575	2.34632	-0.37361
H	-1.64394	2.70036	0.04816
O	-3.03680	0.40448	0.60556
H	-3.43312	-0.44813	0.82177
O	-1.89608	-2.21829	0.14268
C	1.53867	0.52146	-0.02413
H	1.83319	1.55362	-0.22790
C	2.67140	-0.41972	-0.42598
H	2.31670	-1.45330	-0.28900
H	2.96711	-0.31064	-1.47230
O	1.22635	0.35009	1.33435
H	2.05593	0.25328	1.81132
O	3.74122	-0.31193	0.41995
H	0.49168	0.42664	-1.89379

Asc (C1$^{\bullet}$)

C	0.18549	0.12906	0.09424
O	-0.25296	-1.16543	0.13385
C	-1.63642	-1.14092	0.02585
C	-2.02070	0.23775	-0.08618
C	-0.87592	1.00659	-0.04465
O	-0.75100	2.34927	-0.12114
H	-1.62359	2.74786	-0.16165
O	-3.28617	0.65877	-0.20841
H	-3.86020	-0.11738	-0.19266
O	-2.33111	-2.12547	0.02477
C	1.63675	0.39071	0.20342
H	1.79729	1.45075	0.00550
C	2.41115	-0.42837	-0.81557
H	2.20477	-1.48328	-0.65112
H	2.11584	-0.14389	-1.82418
O	2.06277	0.05845	1.51363
H	3.02380	0.02113	1.48340
O	3.78434	-0.13270	-0.58143
H	4.33311	-0.78726	-1.01206

Asc (C2$^{\bullet}$)

C	0.25910	0.01754	-0.79504
O	-0.33940	-1.30049	-0.55470
C	-1.58286	-1.13522	-0.04263
C	-1.87578	0.29021	0.05076
C	-0.82346	0.97152	-0.38572
O	-0.60883	2.28958	-0.49378
H	-1.36721	2.76693	-0.14528
O	-3.05597	0.74629	0.52279
H	-3.58151	-0.02760	0.76089
O	-2.31848	-2.03019	0.26970
C	1.50869	0.17555	-0.03331
C	2.74335	-0.53715	-0.45517
H	2.63712	-1.61240	-0.27769

Asc (C3$^{\bullet}$)

C	0.28631	0.18971	-0.82958
O	-0.13207	-1.17222	-0.65927
C	-1.37766	-1.19255	-0.11467
C	-1.84513	0.17911	0.04438
C	-0.88731	0.99731	-0.37366
O	-0.81340	2.33583	-0.41457
H	-1.59881	2.71502	-0.01004
O	-3.05984	0.46515	0.55979
H	-3.47578	-0.37565	0.78550
O	-1.97907	-2.18985	0.16789
C	1.53542	0.45986	-0.01039
H	1.85677	1.48134	-0.22496
C	2.61505	-0.48456	-0.37109

H	2.92198	-0.37486	-1.51677	H	2.40458	-1.52470	-0.56117	
O	1.35855	0.40572	1.28896	O	1.16649	0.35758	1.36625	
H	2.24226	0.42644	1.67684	H	1.97374	0.31545	1.88264	
O	3.81232	-0.02149	0.33715	O	3.80954	-0.16841	0.21174	
H	4.52226	-0.66173	0.36887	H	4.43964	-0.87874	0.08569	
H	0.46537	0.07316	-1.86115	H	0.50657	0.34921	-1.88229	

SPLET (SP step) mechanism

M05-2X/6-311++G(2d,2p)											
Asc				Asc (O1$^-$)				Asc (O2$^-$)			
C	0.26799200	0.20608800	-0.77718600	C	0.24733	0.23601	-0.85709	C	0.20625	0.18319	-0.84541
O	-0.17999700	-1.15426900	-0.64138500	O	-0.21257	-1.11559	-0.74153	O	-0.17957	-1.18270	-0.64100
C	-1.45256400	-1.15984800	-0.14929800	C	-1.46307	-1.07608	-0.15119	C	-1.44322	-1.23613	-0.06351
C	-1.89332800	0.21617900	0.02975700	C	-1.84003	0.26016	0.11630	C	-1.95585	0.16412	0.13489
C	-0.90029900	1.02254000	-0.32930600	C	-0.88236	1.14594	-0.35093	C	-0.95863	0.94749	-0.35208
O	-0.79793900	2.35777700	-0.32941000	O	-0.77884	2.38325	-0.38407	O	-1.01871	2.31805	-0.40017
H	-1.59258200	2.74597100	0.04828300	O	-3.07520	0.52420	0.67263	H	-1.88788	2.50658	-0.01799
O	-3.11801100	0.52225500	0.50871300	H	-3.47794	-0.34487	0.77696	O	-3.07201	0.51303	0.62386
H	-3.57082700	-0.30977400	0.69128400	O	-2.09721	-2.09632	0.06823	O	-1.94496	-2.29614	0.17993
O	-2.07698400	-2.15359000	0.09191500	C	1.49852	0.44872	-0.02636	C	1.48758	0.48535	-0.07291
C	1.50850900	0.40933800	0.08785300	H	1.76975	1.50081	-0.12474	H	1.76032	1.52103	-0.29047
H	1.78843900	1.45897900	0.03713300	C	2.64287	-0.43355	-0.46919	C	2.62688	-0.42902	-0.45789
C	2.65678300	-0.42941200	-0.43545400	H	2.30897	-1.46987	-0.46932	H	2.31360	-1.46085	-0.31406
H	2.42535100	-1.48453000	-0.28256200	H	2.96074	-0.15609	-1.47044	H	2.89678	-0.27549	-1.49926
H	2.76999300	-0.25126500	-1.50636700	O	1.27282	0.14335	1.34731	O	1.29443	0.33204	1.32447
O	1.21767000	0.14130300	1.43847100	H	0.33259	0.25961	1.52744	H	0.34484	0.42537	1.48603
H	1.06982500	-0.80226800	1.53817900	O	3.76808	-0.27006	0.38901	O	3.78508	-0.14106	0.32051
O	3.82461500	-0.04784000	0.26657100	H	3.40998	-0.27938	1.28069	H	3.47363	-0.07618	1.22708
H	4.53223600	-0.65788100	0.06162400	H	0.45968	0.45198	-1.90234	H	0.40672	0.32384	-1.91073
H	0.50018200	0.38637800	-1.82476400								
Asc (O3$^-$)				Asc (O4$^-$)				Asc (C1$^-$)			
C	0.17861	0.01273	-1.06223	C	0.29939	0.45257	-0.87068	C	0.22611	0.19167	-0.14727
O	-0.66359	-1.14173	-1.00302	O	0.05510	-0.97230	-0.88096	O	-0.21788	-1.13734	-0.05991
C	-1.75957	-0.81200	-0.22740	C	-1.10285	-1.22487	-0.25449	C	-1.61272	-1.13893	-0.00393
C	-1.64518	0.51736	0.24820	C	-1.75487	0.03218	0.06615	C	-2.00936	0.19159	-0.01316
C	-0.46900	1.08091	-0.18375	C	-0.96816	1.02157	-0.33845	C	-0.87465	1.00022	-0.09752
O	0.11503	2.17733	0.02557	O	-1.12971	2.35667	-0.22862	O	-0.87624	2.37058	-0.11218
H	1.75613	1.60756	-0.38497	H	-1.82828	2.52328	0.40848	H	-1.79712	2.63796	-0.08823
O	-2.61794	1.03588	1.07528	O	-2.95604	0.06153	0.70798	O	-3.33081	0.62386	-0.04619
H	-3.24899	0.31590	1.18179	H	-3.15157	-0.86152	0.91106	H	-3.84051	-0.18629	-0.14666
O	-2.65491	-1.61285	-0.01390	O	-1.55638	-2.32643	-0.03546	O	-2.27720	-2.18832	0.00474
C	1.58987	-0.30114	-0.58804	C	1.47989	0.79257	0.08494	C	1.64435	0.43415	0.11441
H	2.05143	-1.03643	-1.24530	H	1.61652	1.88162	-0.06621	H	1.83790	1.50113	0.04885
C	1.63516	-0.82758	0.83440	C	2.78597	0.05790	-0.41851	C	2.56033	-0.31587	-0.83902
H	1.12883	-0.12581	1.49694	H	2.63090	-0.41529	-1.39186	H	2.25597	-1.36194	-0.87168
H	1.14941	-1.79508	0.90060	H	3.59902	0.78347	-0.51800	H	2.47401	0.10701	-1.83457

O	2.36591	0.88952	-0.67241	O	1.21577	0.43103	1.34909	O	2.04881	0.03763	1.46843
O	2.99532	-0.99184	1.22739	H	2.38620	-0.63877	1.23175	H	1.41764	-0.63811	1.72640
H	3.43707	-0.20023	0.90441	O	3.09553	-0.90307	0.55096	O	3.92281	-0.20088	-0.43478
H	0.22819	0.36186	-2.09178	H	0.51674	0.75628	-1.89303	H	3.89180	-0.28109	0.52391

Asc (C2$^-$)				Asc (C3$^-$)			
C	0.63906	-0.31074	0.11840	C	0.34677	0.10283	-0.74646
O	-1.80601	-2.04277	0.06037	O	-0.22079	-1.22616	-0.61216
C	-2.46018	-0.99766	-0.01485	C	-1.47125	-1.13339	-0.14629
C	-1.74797	0.35000	-0.02993	C	-1.83511	0.26655	-0.01533
C	-0.44381	0.63891	0.02786	C	-0.79317	1.00027	-0.38429
O	-0.07666	1.98757	-0.00377	O	-0.64699	2.33879	-0.42717
H	-0.89880	2.48426	-0.07342	H	-1.40574	2.74035	0.00238
O	-2.63367	1.38651	-0.11715	O	-3.06299	0.63595	0.44479
H	-3.47671	0.85267	-0.14151	H	-3.50587	-0.19287	0.66555
O	-3.71066	-0.83321	-0.08746	O	-2.19859	-2.06878	0.10682
C	1.93824	-0.01674	0.20172	C	1.56561	0.28326	0.15808
C	3.01915	-1.03325	0.34495	H	1.92806	1.30032	-0.02572
H	3.45512	-0.95955	1.34406	C	2.63951	-0.72223	-0.07993
H	2.60970	-2.02637	0.20780	H	2.49546	-1.21954	-1.03921
O	2.48161	1.24896	0.18826	O	1.08860	0.25738	1.52886
H	1.75304	1.87629	0.08690	H	1.51889	-0.55752	1.82469
O	4.05078	-0.84384	-0.62565	O	3.93877	-0.02886	-0.16009
H	4.12075	0.10509	-0.75261	H	4.42409	-0.34564	0.59283
H	0.32689	-1.34131	0.12306	H	0.64697	0.22464	-1.78353

SPLET (ETE step) mechanism

M05-2X/6-311++G(2d,2p)											
Asc (O1$^\bullet$)				Asc (O2$^\bullet$)				Asc (C1$^\bullet$)			
C	0.24788	0.18582	-0.89535	C	0.20508	0.17370	-0.86883	C	0.19382	0.10327	0.17473
O	-0.23767	-1.15702	-0.66607	O	-0.21867	-1.18119	-0.65471	O	-0.27398	-1.17898	0.05938
C	-1.45539	-1.13360	-0.09296	C	-1.44684	-1.25863	-0.05002	C	-1.65822	-1.10759	-0.09301
C	-1.88638	0.25212	0.05918	C	-1.96009	0.15815	0.11954	C	-2.00080	0.28510	-0.07603
C	-0.86152	1.12094	-0.42091	C	-0.94999	0.97495	-0.40337	C	-0.84062	1.01603	0.09366
O	-0.79204	2.33379	-0.41406	O	-0.97345	2.28937	-0.40546	O	-0.68229	2.35409	0.17079
O	-3.03902	0.55367	0.60044	H	-1.82387	2.56417	-0.02483	H	-1.54182	2.78083	0.13367
H	-3.47753	-0.27904	0.84072	O	-3.01823	0.54270	0.61460	O	-3.24821	0.75533	-0.19724
O	-2.09559	-2.09423	0.23261	O	-1.96182	-2.28234	0.24651	H	-3.84532	0.00269	-0.29034
C	1.51676	0.43937	-0.10114	C	1.47272	0.46854	-0.07014	O	-2.37004	-2.07056	-0.21154
H	1.89465	1.41873	-0.39074	H	1.79783	1.48073	-0.30979	C	1.65343	0.26887	0.34586
C	2.58147	-0.60535	-0.35992	C	2.58645	-0.49967	-0.41279	H	1.86188	1.29758	0.62210
H	2.20532	-1.58046	-0.05592	H	2.27028	-1.50919	-0.15847	C	2.41144	-0.07050	-0.93416
H	2.82206	-0.63551	-1.41852	H	2.80286	-0.45537	-1.47610	H	2.16427	-1.09135	-1.23316
O	1.23726	0.40599	1.29407	O	1.21971	0.33596	1.32037	H	2.11992	0.60708	-1.73047
H	0.98696	1.28287	1.58997	H	0.98338	1.18400	1.69664	O	2.14298	-0.54478	1.40900
O	3.76906	-0.27385	0.32818	O	3.76957	-0.13877	0.26746	H	1.75207	-1.41944	1.32914
H	3.54843	-0.21026	1.26012	H	3.58958	-0.20258	1.20781	O	3.79666	0.08583	-0.72952
H	0.42739	0.30101	-1.96041	H	0.39918	0.31027	-1.93185	H	4.01382	-0.35595	0.09575

Asc (C3$^\bullet$)			
C	0.28584	0.24837	-0.78172
O	-0.13958	-1.11911	-0.68836
C	-1.40781	-1.16132	-0.18465
C	-1.86413	0.20146	0.05491
C	-0.88662	1.03348	-0.28703
O	-0.80253	2.37006	-0.24150
H	-1.60605	2.73469	0.14119

O	-3.08626	0.47367	0.55742
H	-3.52948	-0.36957	0.71058
O	-2.01740	-2.17055	0.02312
C	1.53823	0.44507	0.06444
H	1.69484	1.52161	0.17606
C	2.71838	-0.21799	-0.54930
H	2.90312	-0.19884	-1.60873
O	1.34398	-0.03259	1.38928
H	1.11867	-0.96692	1.32279
O	3.83314	-0.30139	0.21546
H	3.55970	-0.24839	1.13862
H	0.50508	0.47216	-1.82384

Structures of santowhite (SW) and the corresponding radical species in the HAT mechanism optimized at the M05-2X/6-311++G(2d,2p) level of theory in gas phase.

M06-2X/6-311++G(2d,2p)											
SW				SW (O1•)				SW (O2•)			
C	-2.61591700	-0.93080400	-0.09151600	C	-2.54398	-0.95015	-0.12516	C	-2.64347	-0.93564	-0.07480
C	-1.45603100	-0.16633800	-0.02152400	C	-1.39812	-0.16958	-0.01870	C	-1.49046	-0.19439	-0.02736
C	-1.42088400	1.21609400	-0.16983100	C	-1.38778	1.21798	-0.11208	C	-1.44960	1.20019	-0.20089
C	-2.61061200	1.89040200	-0.44112500	C	-2.58601	1.88508	-0.36141	C	-2.64346	1.91222	-0.48980
C	-3.78048900	1.14894500	-0.52924500	C	-3.74219	1.12790	-0.48125	C	-3.80942	1.21020	-0.55799
C	-3.79464400	-0.22607100	-0.35190000	C	-3.73346	-0.25394	-0.35978	C	-3.88809	-0.21875	-0.35011
H	-0.51893700	-0.66543400	0.15158000	H	-0.45591	-0.66296	0.14420	H	-0.55071	-0.68750	0.14998
H	-4.71258800	1.65488200	-0.74306000	H	-4.68229	1.62628	-0.67617	H	-4.74634	1.69811	-0.77624
C	-0.11285800	1.96892600	0.01586300	C	-0.09275	1.98480	0.11069	C	-0.13870	1.93248	-0.02605
H	-0.17277600	2.87590900	-0.58452700	H	-0.16700	2.91373	-0.45408	H	-0.18782	2.83578	-0.63233
C	1.07312000	1.18558200	-0.52056300	C	1.08510	1.24173	-0.47711	C	1.04304	1.12987	-0.54400
C	1.17816600	0.95280100	-1.89525100	C	1.17734	1.10673	-1.89230	C	1.12833	0.83703	-1.90817
C	2.06556900	0.68020800	0.31090000	C	2.07384	0.67081	0.33842	C	2.05550	0.67783	0.29438
C	2.25321200	0.21198900	-2.35861600	C	2.21309	0.38850	-2.40555	C	2.21236	0.09989	-2.35631
C	3.15346600	-0.07146400	-0.13008800	C	3.13089	-0.06756	-0.14034	C	3.15101	-0.07019	-0.13103
H	1.98958700	0.87908400	1.36277600	H	1.98827	0.81654	1.39855	H	1.99124	0.91803	1.33837
C	3.21898200	-0.29989800	-1.50459800	C	3.22390	-0.24244	-1.58751	C	3.20239	-0.35298	-1.49679
H	2.34817900	0.02697200	-3.42042900	H	2.33053	0.25631	-3.46973	H	2.29547	-0.12824	-3.41049
C	0.04623100	2.44023500	1.47791800	C	0.07261	2.39311	1.59056	C	0.01129	2.42049	1.43534
H	1.03802800	2.87597700	1.58010000	H	1.06712	2.81844	1.70949	H	0.99884	2.86748	1.52360
H	-0.66012600	3.25199600	1.64409200	H	-0.62683	3.20387	1.78652	H	-0.70296	3.22649	1.59248
C	-0.19260800	1.37836300	2.56664400	C	-0.17473	1.29343	2.63875	C	-0.21149	1.36754	2.53551
H	0.51679200	1.53647400	3.37749800	H	0.52359	1.42994	3.46270	H	0.50180	1.54112	3.33932
H	0.00534500	0.38438600	2.17376500	H	0.03399	0.31329	2.21735	H	-0.00535	0.37097	2.15373
C	-1.60993200	1.41359800	3.13365900	C	-1.59916	1.30186	3.18868	C	-1.62478	1.40189	3.11190
H	-1.74158100	0.65693900	3.90331600	H	-1.73296	0.52218	3.93425	H	-1.75171	0.64409	3.88071
H	-1.81668700	2.38491900	3.57893500	H	-1.81835	2.25795	3.65978	H	-1.82854	2.37240	3.55948
H	-2.34528600	1.23481300	2.35343000	H	-2.32420	1.13989	2.39538	H	-2.36909	1.22514	2.33973
C	-2.65844700	3.37849600	-0.66311100	C	-2.65528	3.37959	-0.52338	C	-2.63555	3.39580	-0.74210
H	-2.26245800	3.92551500	0.18814300	H	-2.26904	3.89751	0.35040	H	-2.20803	3.94598	0.09154
H	-2.07181500	3.66007900	-1.53599700	H	-2.07250	3.70434	-1.38386	H	-2.05315	3.63666	-1.62947
H	-3.68011500	3.70530100	-0.82707300	H	-3.68115	3.69794	-0.67588	H	-3.64840	3.75039	-0.89837
C	0.17385500	1.49312200	-2.87588100	C	0.17525	1.75236	-2.80934	C	0.09218	1.30076	-2.89571
H	-0.82101300	1.10368500	-2.67505300	H	-0.82926	1.38426	-2.61831	H	-0.86786	0.82157	-2.71762
H	0.11731900	2.57883600	-2.81620500	H	0.16353	2.83243	-2.67421	H	-0.06298	2.37574	-2.82940
H	0.45268900	1.22808300	-3.89065300	H	0.43211	1.54598	-3.84260	H	0.40465	1.07021	-3.90891
O	4.25765500	-1.03458500	-2.02058700	O	4.13120	-0.89680	-2.13050	O	4.25033	-1.08223	-1.99595
H	4.14890800	-1.09937700	-2.97050300	O	-4.90675	-0.95098	-0.48135	H	4.13135	-1.19368	-2.94043
O	-4.98170000	-0.90840200	-0.44429400	H	-5.62231	-0.33484	-0.64574	O	-4.99227	-0.78557	-0.41940
H	-5.68479800	-0.28457500	-0.63106500	C	-2.49884	-2.47048	0.01469	C	-2.66136	-2.43490	0.15417
C	-2.59694400	-2.44476700	0.11110700	C	-3.36793	-2.91296	1.20151	C	-3.54191	-2.75670	1.37310
C	-3.47069200	-2.82251100	1.31674000	H	-4.40605	-2.63760	1.06143	H	-4.56412	-2.43924	1.20900
H	-4.50312100	-2.53032900	1.16817300	H	-3.30734	-3.99450	1.30894	H	-3.52746	-3.83099	1.54759
H	-3.43249400	-3.90010400	1.46696400	H	-3.00566	-2.45754	2.12108	H	-3.15344	-2.26187	2.26140
H	-3.09584600	-2.33830200	2.21627100	C	-1.07651	-2.96998	0.27778	C	-1.26034	-2.98331	0.42772
C	-1.18308600	-2.95783400	0.39204400	H	-0.40222	-2.72129	-0.53887	H	-0.58763	-2.81003	-0.40969
H	-0.50674600	-2.75355300	-0.43503000	H	-0.67217	-2.56494	1.20343	H	-0.82494	-2.54459	1.32338
H	-0.76952500	-2.51994500	1.29807600	H	-1.10222	-4.05292	0.37152	H	-1.33246	-4.05687	0.58368
H	-1.22739300	-4.03542700	0.53160000	C	-2.99001	-3.13902	-1.27741	C	-3.21638	-3.14782	-1.08949
C	-3.10365500	-3.15850500	-1.15049400	H	-4.01387	-2.86734	-1.50377	H	-4.23063	-2.83117	-1.29884
H	-4.12277000	-2.87717000	-1.38634400	H	-2.35721	-2.84964	-2.11358	H	-2.59169	-2.93954	-1.95601
H	-2.46779400	-2.91557700	-1.99924400	H	-2.93518	-4.22057	-1.16628	H	-3.20902	-4.22220	-0.91510
H	-3.06848700	-4.23542700	-0.99431600	C	4.16740	-0.69697	0.77043	C	4.22524	-0.55127	0.84283

C	4.20254700	-0.61554100	0.83832700	C	5.56422	-0.15518	0.42452	C	5.59293	0.02925	0.45449
C	5.58976100	-0.05557200	0.49092200	H	6.29346	-0.59849	1.10028	H	6.34308	-0.30843	1.16752
H	6.32084400	-0.43721600	1.20188800	H	5.83326	-0.39950	-0.59565	H	5.89323	-0.28585	-0.53732
H	5.89739100	-0.34177600	-0.50743100	H	5.59484	0.92512	0.55214	H	5.56117	1.11643	0.48091
H	5.58370200	1.03042100	0.55666700	C	4.14660	-2.22452	0.59400	C	4.28659	-2.08610	0.84266
C	4.22645800	-2.15021400	0.78020100	H	4.38980	-2.50030	-0.42466	H	4.54016	-2.47487	-0.13608
H	4.48522900	-2.50730200	-0.20921900	H	4.87743	-2.66977	1.26678	H	5.04160	-2.41710	1.55365
H	4.96189900	-2.52735700	1.48902700	H	3.16495	-2.62153	0.84631	H	3.32709	-2.50025	1.14561
H	3.25207200	-2.55071000	1.05267300	C	3.89498	-0.38903	2.24336	C	3.93006	-0.10589	2.27670
C	3.89917400	-0.21779400	2.28445000	H	3.92670	0.68019	2.44293	H	3.90826	0.97827	2.36563
H	3.90062700	0.86238800	2.41369300	H	2.93188	-0.77903	2.56700	H	2.98583	-0.50768	2.63875
H	2.94115100	-0.61010200	2.61923500	H	4.66596	-0.86352	2.84518	H	4.72064	-0.47740	2.92402
H	4.67250200	-0.63176600	2.92710800								
**SW (C1·)**				**SW (C2·)**				**SW (C3·)**			
C	-2.48973	-1.03482	-0.07275	C	-2.64427	-0.91257	-0.09431	C	-3.00361	-0.67042	0.27786
C	-1.46775	-0.12641	-0.01512	C	-1.47893	-0.15024	-0.02656	C	-1.70743	-0.19260	0.16145
C	-1.47798	1.23472	-0.17028	C	-1.42954	1.23126	-0.18444	C	-1.35092	0.98535	-0.51105
C	-2.73438	1.78531	-0.45100	C	-2.61573	1.92743	-0.46471	C	-2.36520	1.72056	-1.13659
C	-3.83345	0.93568	-0.53137	C	-3.73194	1.14331	-0.53218	C	-3.66924	1.25946	-1.03050
C	-3.72919	-0.43827	-0.34371	C	-3.82965	-0.21281	-0.36269	C	-3.99510	0.10585	-0.33584
H	-4.80536	1.35536	-0.75162	H	-0.54612	-0.65419	0.15286	H	-0.90516	-0.74309	0.62169
C	-0.21127	2.05159	-0.00743	C	-0.11877	1.98135	-0.00306	H	-4.46118	1.80652	-1.52586
H	-0.31090	2.94603	-0.62124	H	-0.17608	2.88246	-0.61259	C	0.05526	1.39637	-0.47730
C	0.98182	1.27485	-0.54007	C	1.06280	1.18609	-0.53071	C	1.10607	0.42970	-0.69015
C	1.08658	1.02942	-1.91206	C	1.17086	0.94509	-1.90365	C	0.99887	-0.64256	-1.60708
C	1.96902	0.77158	0.29813	C	2.04714	0.67698	0.30805	C	2.32185	0.55892	0.01328
C	2.15887	0.28000	-2.36764	C	2.24171	0.19325	-2.35862	C	2.06643	-1.51687	-1.71546
C	3.05003	0.00613	-0.13405	C	3.13026	-0.08625	-0.12453	C	3.39419	-0.31135	-0.08566
H	1.89124	0.97848	1.34802	H	1.96843	0.88233	1.35848	H	2.40720	1.38008	0.69889
C	3.11820	-0.23229	-1.50673	C	3.19969	-0.32199	-1.49774	C	3.23147	-1.37998	-0.97513
H	2.25482	0.08533	-3.42762	H	2.33944	0.00168	-3.41898	H	2.00415	-2.32780	-2.42934
C	-0.05807	2.53536	1.44945	C	0.03793	2.46588	1.45487	C	0.39642	2.75414	0.06670
H	0.92612	2.98917	1.54647	H	1.03245	2.89578	1.55475	H	1.40214	3.02412	-0.24564
H	-0.77997	3.33434	1.61294	H	-0.66344	3.28406	1.61009	H	-0.27075	3.50203	-0.35468
C	-0.27404	1.46561	2.53588	C	-0.21103	1.41725	2.55393	C	0.28839	2.86684	1.60429
H	0.45743	1.60803	3.32975	H	0.49617	1.58072	3.36558	H	0.84670	3.74867	1.91619
H	-0.09704	0.47536	2.12178	H	-0.01725	0.41795	2.17278	H	0.76715	2.00733	2.06964
C	-1.67661	1.50531	3.13706	C	-1.63029	1.46650	3.11505	C	-1.14848	2.98510	2.10412
H	-1.79597	0.74255	3.90255	H	-1.76861	0.71933	3.89270	H	-1.16977	3.13483	3.18065
H	-1.86812	2.47398	3.59485	H	-1.83374	2.44379	3.54856	H	-1.64368	3.83511	1.63759
H	-2.43070	1.33687	2.37204	H	-2.36407	1.28267	2.33457	H	-1.72649	2.09570	1.87358
C	-2.90624	3.26229	-0.68525	C	-2.67284	3.41335	-0.70112	C	-2.09487	2.93872	-1.97810
H	-2.55526	3.84221	0.16452	H	-2.28198	3.96554	0.14885	H	-2.17041	3.85495	-1.39534
H	-2.34166	3.58346	-1.55893	H	-2.08463	3.68536	-1.57550	H	-1.09963	2.89738	-2.41091
H	-3.95107	3.50298	-0.85205	H	-3.69693	3.72592	-0.87019	H	-2.81971	3.00257	-2.78462
C	0.08195	1.56045	-2.89753	C	0.17397	1.48732	-2.89074	C	-0.16704	-0.83219	-2.54129
H	-0.91238	1.17167	-2.69169	H	-0.82550	1.11114	-2.68753	H	-1.00539	-1.32729	-2.05736
H	0.02535	2.64675	-2.85104	H	0.13009	2.57417	-2.84247	H	-0.53058	0.12396	-2.90573
H	0.35906	1.28333	-3.90951	H	0.45058	1.20877	-3.90242	H	0.14167	-1.43301	-3.39172
O	4.15258	-0.97939	-2.01403	O	4.23435	-1.06757	-2.00523	O	4.24492	-2.28918	-1.13275
H	4.04270	-1.05515	-2.96297	H	4.13004	-1.13593	-2.95542	H	3.97220	-2.95104	-1.77012
O	-4.84084	-1.23453	-0.42742	O	-5.03143	-0.86330	-0.46002	O	-5.30440	-0.29379	-0.26105
H	-5.60865	-0.68468	-0.59201	H	-5.70664	-0.21137	-0.65860	H	-5.85090	0.34308	-0.72391
C	-2.26276	-2.52443	0.14142	C	-2.63630	-2.42639	0.11765	C	-3.32673	-1.96231	1.02563
C	-3.06805	-3.01408	1.35063	C	-3.51861	-2.78695	1.32181	C	-4.25881	-1.66963	2.21045
H	-4.13310	-2.88372	1.19397	H	-4.54749	-2.48519	1.16653	H	-5.20083	-1.24749	1.88171
H	-2.86598	-4.07112	1.51508	H	-3.49214	-3.86388	1.47872	H	-4.46167	-2.59468	2.74725
H	-2.77582	-2.46626	2.24411	H	-3.14310	-2.30076	2.21989	H	-3.78428	-0.97276	2.89813
C	-0.77736	-2.77109	0.41161	C	-1.22677	-2.94669	0.40556	C	-2.06414	-2.62117	1.58388

H	-0.16391	-2.42470	-0.41774	H	-0.54721	-2.75170	-0.42099	H	-1.36153	-2.87995	0.79451
H	-0.45425	-2.25023	1.31088	H	-0.81331	-2.50613	1.31019	H	-1.55994	-1.98124	2.30415
H	-0.60684	-3.83670	0.55028	H	-1.27928	-4.02316	0.55122	H	-2.34851	-3.53895	2.09293
C	-2.66942	-3.30706	-1.11279	C	-3.14681	-3.13990	-1.14215	C	-3.98790	-2.97076	0.07398
H	-3.72154	-3.17124	-1.33787	H	-4.16260	-2.84897	-1.38146	H	-4.91766	-2.58887	-0.32989
H	-2.08262	-2.97919	-1.96800	H	-2.50667	-2.90714	-1.99043	H	-3.31738	-3.20379	-0.75090
H	-2.48041	-4.36727	-0.95339	H	-3.12324	-4.21632	-0.98099	H	-4.19630	-3.89232	0.61461
C	4.08430	-0.55030	0.84321	C	4.17050	-0.63455	0.85102	C	4.67157	-0.11939	0.73015
C	5.48340	-0.01561	0.50433	C	5.56366	-0.08693	0.50765	C	5.87276	0.07062	-0.20751
H	6.20266	-0.40723	1.22196	H	6.28831	-0.47077	1.22397	H	6.77224	0.22855	0.38527
H	5.79347	-0.31046	-0.49074	H	5.87396	-0.38034	-0.48777	H	6.02464	-0.79454	-0.84136
H	5.49564	1.07057	0.56636	H	5.56575	0.99937	0.56815	H	5.72136	0.94396	-0.83831
C	4.08110	-2.08539	0.78982	C	4.18259	-2.16958	0.79911	C	4.90379	-1.33214	1.64322
H	4.34232	-2.44950	-0.19645	H	4.44211	-2.53252	-0.18799	H	5.01804	-2.24559	1.07251
H	4.80308	-2.47389	1.50641	H	4.91253	-2.54956	1.51206	H	5.80747	-1.17405	2.22954
H	3.09667	-2.46682	1.05374	H	3.20419	-2.56151	1.06965	H	4.06767	-1.45088	2.32902
C	3.77675	-0.14317	2.28583	C	3.86347	-0.22842	2.29401	C	4.59193	1.11774	1.62708
H	3.79455	0.93730	2.41182	H	3.87371	0.85222	2.41909	H	4.45934	2.02849	1.04712
H	2.80979	-0.51903	2.61381	H	2.90041	-0.61105	2.62557	H	3.78312	1.04021	2.35048
H	4.53845	-0.56752	2.93564	H	4.62996	-0.64649	2.94215	H	5.52534	1.20547	2.17795
**SW (C4•)**				**SW (C5•)**				**SW (C6•)**			
C	-2.63936	-0.88802	-0.07571	C	-2.63105	-0.91597	-0.07217	C	-2.97684	-0.20469	-0.17515
C	-1.46445	-0.14420	-0.05259	C	-1.46136	-0.16493	-0.02468	C	-1.92032	0.68291	0.01541
C	-1.41457	1.24018	-0.17457	C	-1.41061	1.21371	-0.20091	C	-0.98006	0.99282	-0.95814
C	-2.60447	1.93928	-0.36955	C	-2.59319	1.89765	-0.47836	C	-1.09098	0.40061	-2.21600
C	-3.79026	1.21919	-0.40625	C	-3.77262	1.16921	-0.54448	C	-2.14005	-0.47878	-2.43400
C	-3.81820	-0.15871	-0.25469	C	-3.80265	-0.20169	-0.33958	C	-3.06158	-0.78434	-1.44277
H	-0.52858	-0.66086	0.06406	H	-0.52931	-0.67184	0.15306	H	-1.81231	1.16029	0.97364
H	-4.72377	1.74426	-0.55899	H	-4.69981	1.68235	-0.76222	H	-2.24424	-0.94778	-3.40364
C	-0.09031	1.97146	-0.03591	C	-0.09372	1.95444	-0.03548	C	0.19607	1.91058	-0.64548
H	-0.15096	2.87976	-0.63446	H	-0.14393	2.85011	-0.65376	H	0.33139	2.54311	-1.52427
C	1.06703	1.16718	-0.59027	C	1.08217	1.14810	-0.56119	C	1.48836	1.12444	-0.45212
C	1.18798	0.88745	-1.96501	C	1.15238	0.86783	-1.93923	C	2.72096	1.74117	-0.66143
C	2.07047	0.65253	0.18141	C	2.09593	0.67679	0.26576	C	1.46409	-0.19856	-0.02848
C	2.26182	0.13205	-2.40861	C	2.23372	0.12921	-2.31725	C	3.87192	0.99837	-0.44479
C	3.16288	-0.10416	-0.16648	C	3.18759	-0.08120	-0.16561	C	2.60392	-0.96316	0.20372
C	3.23094	-0.36248	-1.54220	H	2.03661	0.90980	1.31133	H	0.49960	-0.65083	0.12028
H	2.37313	-0.09013	-3.45773	C	3.24618	-0.36351	-1.53477	C	3.82418	-0.32234	-0.02386
C	0.16316	2.42010	1.41923	C	0.07976	2.45184	1.41600	H	4.83761	1.45871	-0.60779
H	1.16275	2.84896	1.45190	H	1.07188	2.89014	1.50135	C	-0.06448	2.82241	0.51510
H	-0.53262	3.22279	1.65900	H	-0.62606	3.26584	1.57318	H	-0.68245	3.69111	0.34606
C	0.02534	1.32528	2.48791	C	-0.14916	1.40800	2.52377	C	0.41036	2.53748	1.89512
H	0.76678	1.49963	3.26523	H	0.56854	1.57820	3.32480	H	1.47588	2.77689	1.98546
H	0.26318	0.35548	2.05577	H	0.04447	0.40776	2.14469	H	0.35371	1.46415	2.08932
C	-1.36057	1.27733	3.12824	C	-1.56063	1.45463	3.10436	C	-0.37769	3.30845	2.95014
H	-1.41497	0.49535	3.88197	H	-1.68612	0.70950	3.88616	H	-0.00013	3.10854	3.94867
H	-1.58487	2.22550	3.61339	H	-1.76070	2.43273	3.53767	H	-0.31110	4.37892	2.77024
H	-2.13245	1.08661	2.38762	H	-2.30422	1.26637	2.33424	H	-1.42922	3.03049	2.92143
C	-2.63661	3.43213	-0.56017	C	-2.62486	3.38188	-0.72778	C	-0.10867	0.68964	-3.31752
H	-2.18592	3.95500	0.27900	H	-2.21694	3.94011	0.11045	H	-0.15347	1.73610	-3.61469
H	-2.09179	3.72075	-1.45745	H	-2.04132	3.64087	-1.60960	H	0.90956	0.48217	-2.99631
H	-3.65929	3.77993	-0.66340	H	-3.64370	3.71765	-0.89102	H	-0.32542	0.08422	-4.19174
C	0.17424	1.40191	-2.95007	C	0.13206	1.34452	-2.93733	C	2.82187	3.17160	-1.11448
H	-0.81351	1.00055	-2.73681	H	-0.83593	0.88715	-2.74975	H	2.40967	3.29461	-2.11496
H	0.10234	2.48708	-2.90162	H	0.00492	2.42331	-2.87524	H	2.26549	3.83070	-0.45142
H	0.45442	1.12537	-3.96100	H	0.44977	1.09583	-3.94359	H	3.85737	3.49569	-1.13554
O	4.22827	-1.09538	-2.12393	O	4.27355	-1.09668	-2.06842	O	4.99478	-1.01272	0.16984
H	4.84769	-1.38552	-1.45569	H	4.12039	-1.17793	-3.01202	H	5.73245	-0.43140	-0.02054
O	-5.02006	-0.81988	-0.29238	O	-4.99843	-0.87062	-0.41037	O	-4.07415	-1.67119	-1.71250

H	-5.72223	-0.18184	-0.42802	H	-5.69482	-0.24238	-0.60716	H	-3.98804	-1.97313	-2.61791
C	-2.63571	-2.40640	0.09218	C	-2.63004	-2.42555	0.16163	C	-3.96846	-0.53160	0.93931
C	-3.44455	-2.79727	1.33830	C	-3.49991	-2.76642	1.38101	C	-5.39369	-0.14907	0.51366
H	-4.47928	-2.48764	1.25533	H	-4.52924	-2.46266	1.23402	H	-5.70449	-0.69313	-0.36996
H	-3.41444	-3.87831	1.46394	H	-3.47559	-3.84124	1.55242	H	-6.08500	-0.37613	1.32349
H	-3.01201	-2.33741	2.22458	H	-3.11168	-2.26973	2.26798	H	-5.44962	0.91726	0.30534
C	-1.21714	-2.94914	0.27871	C	-1.22096	-2.95087	0.44348	C	-3.65095	0.23981	2.22195
H	-0.58600	-2.73526	-0.58111	H	-0.54806	-2.77256	-0.39233	H	-2.66382	-0.00734	2.60693
H	-0.74607	-2.54025	1.17019	H	-0.79570	-2.49983	1.33751	H	-3.70909	1.31513	2.06786
H	-1.27205	-4.02916	0.39463	H	-1.27810	-4.02466	0.60552	H	-4.38265	-0.03054	2.97953
C	-3.22812	-3.08194	-1.15313	C	-3.15495	-3.15886	-1.08113	C	-3.90388	-2.02945	1.27394
H	-4.25344	-2.77613	-1.32262	H	-4.17228	-2.86999	-1.31538	H	-4.16293	-2.63965	0.41684
H	-2.63789	-2.83165	-2.03217	H	-2.52257	-2.94190	-1.93946	H	-2.90164	-2.29701	1.60300
H	-3.20517	-4.16254	-1.02232	H	-3.13184	-4.23260	-0.90266	H	-4.59974	-2.25064	2.08155
C	4.17081	-0.58982	0.87062	C	4.26015	-0.58182	0.80209	C	2.51689	-2.41386	0.67467
C	5.56965	-0.02121	0.58708	C	5.63118	-0.01257	0.41034	C	3.22677	-2.57582	2.02711
H	6.26071	-0.35794	1.35729	H	6.38221	-0.36387	1.11595	H	3.13581	-3.60823	2.36025
H	5.97965	-0.31845	-0.37523	H	5.92154	-0.32330	-0.58609	H	4.27848	-2.32593	1.95774
H	5.53765	1.06507	0.60205	H	5.61076	1.07458	0.44493	H	2.76302	-1.93425	2.77344
C	4.20540	-2.12473	0.92343	C	4.30426	-2.11672	0.78489	C	3.15454	-3.34605	-0.36623
H	4.50516	-2.58924	-0.01301	H	4.54963	-2.49669	-0.19965	H	4.20221	-3.11585	-0.51851
H	4.90655	-2.44705	1.69075	H	5.05844	-2.46409	1.48901	H	3.07106	-4.37711	-0.02659
H	3.21956	-2.50847	1.17308	H	3.34116	-2.52336	1.08637	H	2.63448	-3.25650	-1.31758
C	3.74810	-0.09376	2.25511	C	3.97315	-0.14500	2.24000	C	1.06508	-2.85948	0.86274
H	3.70861	0.99257	2.28145	H	3.96297	0.93846	2.33711	H	0.55532	-2.26069	1.61453
H	2.76576	-0.47579	2.52067	H	3.02518	-0.53917	2.60013	H	0.50179	-2.80545	-0.06623
H	4.46653	-0.43417	2.99798	H	4.76150	-0.53059	2.88211	H	1.06123	-3.89378	1.19891
**SW (C7·)**				**SW (C8·)**				**SW (C9·)**			
C	-2.97991	-0.14374	-0.29774	C	-3.08784	-0.19805	-0.24122	C	-3.09560	-0.23928	-0.24570
C	-1.93614	0.63326	0.20080	C	-2.04459	0.67093	0.07320	C	-2.03842	0.65262	-0.03983
C	-0.95742	1.22554	-0.58770	C	-0.99499	0.98109	-0.78407	C	-0.99115	0.85383	-0.91942
C	-1.02541	1.05709	-1.97264	C	-0.99202	0.40976	-2.06034	C	-0.99444	0.12792	-2.14281
C	-2.05896	0.29529	-2.49382	C	-2.02331	-0.45190	-2.39906	C	-2.05966	-0.77197	-2.36544
C	-3.01436	-0.30088	-1.68396	C	-3.04569	-0.76375	-1.51549	C	-3.06981	-0.95800	-1.45187
H	-1.86692	0.77074	1.26459	H	-2.05777	1.13044	1.04276	H	-2.04485	1.21968	0.87087
H	-2.12478	0.15605	-3.56485	H	-2.03633	-0.89552	-3.38581	H	-2.07667	-1.33105	-3.29099
C	0.19204	2.01298	0.01251	C	0.13423	1.91498	-0.37777	C	0.13476	1.82986	-0.62929
H	0.29429	2.91573	-0.58696	H	0.19304	2.67533	-1.15558	H	0.18076	2.49649	-1.49039
C	1.50779	1.25559	-0.08477	C	1.47756	1.20190	-0.34034	C	1.48640	1.13967	-0.51203
C	2.71092	1.91830	-0.32014	C	2.65510	1.85561	-0.69662	C	2.66094	1.78030	-0.90112
C	1.52642	-0.12588	0.07868	C	1.55069	-0.12059	0.08478	C	1.57056	-0.13637	0.03394
C	3.87623	1.16512	-0.37115	C	3.85046	1.15566	-0.59869	C	3.86539	1.11649	-0.70731
C	2.67944	-0.90201	0.03989	C	2.73362	-0.84332	0.19258	C	2.76233	-0.82206	0.23836
H	0.57986	-0.61127	0.23895	H	0.62358	-0.60826	0.33084	H	0.64549	-0.61567	0.30251
C	3.87129	-0.20949	-0.19356	C	3.89991	-0.15850	-0.16226	C	3.92554	-0.14889	-0.14631
H	4.81881	1.66154	-0.56010	H	4.77409	1.64631	-0.87560	H	4.78746	1.59691	-1.00657
C	-0.06538	2.50008	1.45694	C	-0.11179	2.67454	0.94035	C	-0.11270	2.72787	0.60012
H	0.67488	3.28142	1.66719	H	0.53249	3.55161	0.95735	H	0.53151	3.60185	0.52057
H	-1.03780	2.99409	1.48800	H	-1.13479	3.04795	0.94482	H	-1.13410	3.10542	0.55851
C	0.00575	1.46940	2.52615	C	0.15419	1.88059	2.22105	C	0.15088	2.08172	1.96138
H	0.79627	0.73931	2.48834	H	1.23183	1.72091	2.32167	H	1.22311	1.95840	2.09418
C	-0.76313	1.65193	3.78511	H	-0.25865	0.87159	2.14594	H	-0.27163	1.08008	2.00184
H	-0.75962	0.75343	4.39512	C	-0.38169	2.55974	3.42525	C	-0.42327	2.92866	3.09160
H	-0.34970	2.46216	4.39582	H	-0.30958	2.09917	4.39493	H	-0.18915	2.50640	4.06479
H	-1.79939	1.92251	3.57762	H	-0.80342	3.54835	3.36140	H	-0.02243	3.93979	3.05731
C	-0.01685	1.67943	-2.89878	C	0.08499	0.69984	-3.06978	H	-1.50656	2.99999	3.00655
H	-0.04394	2.76592	-2.83101	H	0.25240	1.76979	-3.17272	C	-0.00689	0.27313	-3.12485
H	0.99230	1.35992	-2.64915	H	1.03103	0.25293	-2.77334	H	0.84077	0.91869	-2.99466
H	-0.22252	1.40371	-3.92803	H	-0.19373	0.30796	-4.04277	H	-0.07406	-0.29178	-4.03840

C	2.77743	3.40349	-0.55822	C	2.66722	3.27071	-1.21138	C	2.66287	3.14022	-1.54845
H	2.24225	3.67451	-1.46704	H	2.12674	3.34736	-2.15331	H	2.11642	3.12803	-2.49028
H	2.33595	3.96450	0.26095	H	2.20283	3.95915	-0.51103	H	2.19967	3.89119	-0.91484
H	3.80805	3.72467	-0.66863	H	3.68580	3.60286	-1.38351	H	3.67898	3.45823	-1.75796
O	5.05316	-0.90460	-0.25502	O	5.10968	-0.80197	-0.08376	O	5.14434	-0.75647	0.02647
H	5.76703	-0.28892	-0.42740	H	5.80103	-0.19598	-0.35426	H	5.83226	-0.16538	-0.28305
O	-4.00996	-1.05422	-2.25520	O	-4.03399	-1.63343	-1.90421	O	-4.07635	-1.85396	-1.71588
H	-3.88453	-1.06322	-3.20526	H	-3.84952	-1.93281	-2.79563	H	-3.90620	-2.26812	-2.56328
C	-4.01037	-0.79454	0.62342	C	-4.20593	-0.50933	0.75285	C	-4.20364	-0.42057	0.78695
C	-5.42199	-0.29303	0.28493	C	-5.56355	-0.08771	0.17097	C	-5.56531	-0.05769	0.17463
H	-5.70326	-0.54696	-0.72989	H	-5.78505	-0.61647	-0.74803	H	-5.80411	-0.69190	-0.67022
H	-6.13915	-0.74615	0.96757	H	-6.34906	-0.30300	0.89353	H	-6.34196	-0.17552	0.92853
H	-5.47480	0.78734	0.40231	H	-5.56887	0.98130	-0.03114	H	-5.56408	0.97900	-0.15553
C	-3.74653	-0.45772	2.09236	C	-4.01970	0.24435	2.07121	C	-3.99787	0.48022	2.00723
H	-2.76948	-0.80723	2.41969	H	-3.08933	-0.02811	2.56512	H	-3.06400	0.26036	2.52061
H	-3.81356	0.61259	2.27484	H	-4.03517	1.32220	1.92516	H	-4.01117	1.53413	1.73715
H	-4.50104	-0.94929	2.70194	H	-4.83966	-0.01460	2.73683	H	-4.81184	0.30525	2.70657
C	-3.94832	-2.32271	0.48250	C	-4.21814	-2.01031	1.07855	C	-4.22423	-1.87310	1.28845
H	-4.16999	-2.63809	-0.52998	H	-4.39083	-2.60865	0.19203	H	-4.41612	-2.56969	0.48169
H	-2.95784	-2.68390	0.75214	H	-3.26898	-2.30600	1.52075	H	-3.27158	-2.12490	1.74967
H	-4.67376	-2.77796	1.15489	H	-5.00956	-2.21746	1.79706	H	-5.00664	-1.98405	2.03733
C	2.63782	-2.41595	0.23983	C	2.75158	-2.29624	0.66425	C	2.79324	-2.22688	0.83719
C	3.47719	-2.80963	1.46452	C	3.58978	-2.42578	1.94446	C	3.59118	-2.22563	2.14932
H	3.42432	-3.88770	1.60675	H	3.57652	-3.46096	2.28189	H	3.58939	-3.22823	2.57374
H	4.51604	-2.52764	1.34389	H	4.61827	-2.12859	1.77902	H	4.61803	-1.91916	1.99089
H	3.08527	-2.32806	2.35795	H	3.16861	-1.80478	2.73237	H	3.13116	-1.54976	2.86725
C	3.16671	-3.13228	-1.01123	C	3.32715	-3.20164	-0.43490	C	3.42224	-3.21163	-0.15989
H	4.19562	-2.86476	-1.21921	H	4.34362	-2.92539	-0.68795	H	4.44213	-2.93767	-0.40145
H	3.11216	-4.20916	-0.86025	H	3.32326	-4.23425	-0.08971	H	3.42356	-4.21097	0.27206
H	2.55673	-2.87692	-1.87518	H	2.71324	-3.13892	-1.33101	H	2.83947	-3.23696	-1.07830
C	1.21083	-2.91206	0.48366	C	1.34365	-2.80231	0.98345	C	1.38576	-2.73467	1.15570
H	0.77871	-2.46972	1.37879	H	0.88169	-2.22790	1.78361	H	0.88797	-2.10487	1.88988
H	0.55900	-2.69796	-0.36056	H	0.69556	-2.76921	0.11042	H	0.76562	-2.78904	0.26358
H	1.23825	-3.99033	0.6224	H	1.41386	-3.83673	1.31135	H	1.46498	-3.73625	1.57183
**SW (C10•)**				**SW (C11•)**				**SW (C12•)**			
C	-3.09560	-0.23928	-0.24570	C	-2.62402	-0.95273	-0.08667	C	-2.60186	-0.92512	-0.09585
C	-2.03842	0.65262	-0.03983	C	-1.46432	-0.18915	-0.02363	C	-1.43884	-0.16602	-0.01932
C	-0.99115	0.85383	-0.91942	C	-1.43184	1.19201	-0.17862	C	-1.39598	1.21569	-0.17210
C	-0.99444	0.12792	-2.14281	C	-2.62555	1.86044	-0.45099	C	-2.58037	1.89470	-0.45487
C	-2.05966	-0.77197	-2.36544	C	-3.79519	1.11694	-0.52974	C	-3.75304	1.15858	-0.54966
C	-3.06981	-0.95800	-1.45187	C	-3.80716	-0.25725	-0.34341	C	-3.77513	-0.21573	-0.36794
H	-2.04485	1.21968	0.87087	H	-0.53222	-0.70268	0.15203	H	-0.50564	-0.66887	0.16358
H	-2.07667	-1.33105	-3.29099	H	-4.72864	1.62080	-0.74259	H	-4.68107	1.66826	-0.77205
C	0.13476	1.82986	-0.62929	C	-0.12687	1.95082	0.00245	C	-0.08624	1.96318	0.02161
H	0.18076	2.49649	-1.49039	H	-0.18965	2.85365	-0.60390	H	-0.13732	2.86857	-0.58200
C	1.48640	1.13967	-0.51203	C	1.06291	1.16879	-0.52715	C	1.10063	1.17393	-0.50350
C	2.66094	1.78030	-0.90112	C	1.17257	0.93037	-1.90041	C	1.21719	0.93841	-1.87761
C	1.57056	-0.13637	0.03394	C	2.05550	0.67196	0.30914	C	2.08616	0.66723	0.33439
C	3.86539	1.11649	-0.70731	C	2.25258	0.19316	-2.35783	C	2.29499	0.19656	-2.33390
C	2.76233	-0.82206	0.23836	C	3.14845	-0.07580	-0.12575	C	3.17481	-0.08408	-0.10183
H	0.64549	-0.61567	0.30251	H	1.97554	0.87461	1.35997	H	2.01888	0.85676	1.39172
C	3.92554	-0.14889	-0.14631	C	3.21890	-0.30964	-1.49908	C	3.25556	-0.31517	-1.47348
H	4.78746	1.59691	-1.00657	H	2.35130	0.00400	-3.41857	H	2.39661	0.01148	-3.39510
C	-0.11270	2.72787	0.60012	C	0.02839	2.43189	1.46181	C	0.06545	2.43768	1.48337
H	0.53151	3.60185	0.52057	H	1.01885	2.87093	1.56271	H	1.05991	2.86570	1.59133
H	-1.13410	3.10542	0.55851	H	-0.68040	3.24278	1.62190	H	-0.63603	3.25517	1.64218
C	0.15088	2.08172	1.96138	C	-0.20923	1.37573	2.55636	C	-0.18854	1.38095	2.57326
H	1.22311	1.95840	2.09418	H	0.49993	1.53909	3.36639	H	0.51350	1.53973	3.39024
H	-0.27163	1.08008	2.00184	H	-0.01080	0.37973	2.16896	H	0.01084	0.38502	2.18635

C	-0.42327	2.92866	3.09160	C	-1.62660	1.41297	3.12318	C	-1.61132	1.42358	3.12604
H	-0.18915	2.50640	4.06479	H	-1.75739	0.66100	3.89756	H	-1.75341	0.67016	3.89704
H	-0.02243	3.93979	3.05731	H	-1.83471	2.38681	3.56233	H	-1.81869	2.39723	3.56603
H	-1.50656	2.99999	3.00655	H	-2.36173	1.22833	2.34405	H	-2.33989	1.24499	2.33945
C	-0.00689	0.27313	-3.12485	C	-2.67757	3.34700	-0.68272	C	-2.61945	3.38242	-0.68110
H	0.84077	0.91869	-2.99466	H	-2.28133	3.90052	0.16418	H	-2.22672	3.92957	0.17158
H	-0.07406	-0.29178	-4.03840	H	-2.09310	3.62408	-1.55848	H	-2.02541	3.65899	-1.55057
C	2.66287	3.14022	-1.54845	H	-3.70030	3.67026	-0.84703	H	-3.63835	3.71377	-0.85302
H	2.11642	3.12803	-2.49028	C	0.16814	1.46170	-2.88582	C	0.22077	1.47880	-2.86636
H	2.19967	3.89119	-0.91484	H	-0.82672	1.07426	-2.68128	H	-0.77634	1.09245	-2.67095
H	3.67898	3.45823	-1.75796	H	0.11188	2.54797	-2.83631	H	0.16668	2.56480	-2.80982
O	5.14434	-0.75647	0.02647	H	0.44672	1.18723	-3.89813	H	0.50559	1.21040	-3.87856
H	5.83226	-0.16538	-0.28305	O	4.26289	-1.04106	-2.00915	O	4.30039	-1.04892	-1.97809
O	-4.07635	-1.85396	-1.71588	H	4.15776	-1.10922	-2.95923	H	4.20210	-1.11458	-2.92912
H	-3.90620	-2.26812	-2.56328	O	-4.99184	-0.94526	-0.42167	O	-4.96471	-0.89267	-0.46728
C	-4.20364	-0.42057	0.78695	H	-5.69980	-0.32590	-0.60508	H	-5.66318	-0.26603	-0.66180
C	-5.56531	-0.05769	0.17463	C	-2.58155	-2.47303	0.12676	C	-2.59215	-2.43844	0.11217
H	-5.80411	-0.69190	-0.67022	C	-3.43379	-2.84874	1.35102	C	-3.47764	-2.80753	1.31187
H	-6.34196	-0.17552	0.92853	H	-4.46919	-2.56242	1.21129	H	-4.50726	-2.51047	1.15370
H	-5.56408	0.97900	-0.15553	H	-3.38346	-3.92392	1.50933	H	-3.44622	-3.88478	1.46614
C	-3.99787	0.48022	2.00723	H	-3.04862	-2.35088	2.23830	H	-3.10776	-2.32205	2.21277
H	-3.06400	0.26036	2.52061	C	-1.18569	-2.94508	0.37642	C	-1.18327	-2.95766	0.40628
H	-4.01117	1.53413	1.73715	H	-0.52195	-3.15339	-0.44616	H	-0.49946	-2.75981	-0.41619
H	-4.81184	0.30525	2.70657	H	-0.79732	-3.00611	1.37932	H	-0.77468	-2.51847	1.31392
C	-4.22423	-1.87310	1.28845	C	-3.10294	-3.19774	-1.12683	H	-1.23427	-4.03450	0.54935
H	-4.41612	-2.56969	0.48169	H	-4.12895	-2.92354	-1.34068	C	-3.09214	-3.15394	-1.15114
H	-3.27158	-2.12490	1.74967	H	-2.48539	-2.94592	-1.98609	H	-4.10759	-2.86779	-1.39673
H	-5.00664	-1.98405	2.03733	H	-3.05090	-4.27278	-0.96855	H	-2.44770	-2.91758	-1.99526
C	2.79324	-2.22688	0.83719	C	4.19764	-0.61052	0.84774	H	-3.06426	-4.23048	-0.99090
C	3.59118	-2.22563	2.14932	C	5.58294	-0.04490	0.50185	C	4.21403	-0.62112	0.89388
H	3.58939	-3.22823	2.57374	H	6.31413	-0.42035	1.21601	C	5.60390	-0.05066	0.56242
H	4.61803	-1.91916	1.99089	H	5.89449	-0.33312	-0.49470	H	6.32792	-0.42372	1.28385
H	3.13116	-1.54976	2.86725	H	5.57127	1.04128	0.56365	H	5.91960	-0.33957	-0.43280
C	3.42224	-3.21163	-0.15989	C	4.22956	-2.14527	0.79585	H	5.58503	1.03523	0.62265
H	4.44213	-2.93767	-0.40145	H	4.49222	-2.50494	-0.19163	C	4.24458	-2.15889	0.83690
H	3.42356	-4.21097	0.27206	H	4.96537	-2.51591	1.50772	H	4.51735	-2.50879	-0.15143
H	2.83947	-3.23696	-1.07830	H	3.25662	-2.54951	1.06787	H	4.97027	-2.53260	1.55626
C	1.38576	-2.73467	1.15570	C	3.88825	-0.20856	2.29142	H	3.26660	-2.56026	1.09358
H	0.88797	-2.10487	1.88988	H	3.88400	0.87214	2.41638	C	3.88079	-0.22494	2.29568
H	0.76562	-2.78904	0.26358	H	2.93116	-0.60423	2.62491	H	4.23981	0.70500	2.70429
H	1.46498	-3.73625	1.57183	H	4.66179	-0.61620	2.93788	H	3.22765	-0.83449	2.89806

SET-PT (SET step)

M05-2X/6-311++G(2d,2p)			
SW •+			
C	2.44020600	1.00898900	-0.11449800
C	1.34862700	0.16872100	0.04606400
C	1.40466300	-1.22021000	-0.08706300
C	2.61629800	-1.82722600	-0.42868000
C	3.71943800	-1.00906400	-0.59021900
C	3.64743100	0.37443900	-0.43824800
H	0.40503800	0.61291200	0.30995900
H	4.67382200	-1.45251500	-0.83707100
C	0.15948500	-2.05370900	0.17312400
H	0.27032800	-2.97140200	-0.40686700
C	-0.99814900	-1.32715100	-0.43426100
C	-1.07112900	-1.18410100	-1.86730800
C	-1.99451700	-0.74412600	0.36708300
C	-2.09554400	-0.44508800	-2.38270000
C	-3.02713600	0.01692300	-0.12316100
H	-1.92575000	-0.89491300	1.42671600
C	-3.05213000	0.16539800	-1.55317800
H	-2.18842900	-0.32074400	-3.45180400
C	-0.01147600	-2.47599800	1.64502300
H	-1.00613200	-2.90511700	1.75578900
H	0.68392000	-3.29237100	1.82763700
C	0.24081400	-1.39399100	2.70883900
H	-0.41573100	-1.58414100	3.55539100
H	-0.02184100	-0.40843500	2.32939100
C	1.68540300	-1.36625500	3.20225300
H	1.81965000	-0.60082700	3.96114700
H	1.95189300	-2.32399000	3.64275700
H	2.37724500	-1.16126800	2.38987700
C	2.75181600	-3.31423800	-0.60854400
H	2.40174200	-3.85557400	0.26669200
H	2.18038800	-3.66299700	-1.46722400
H	3.78893200	-3.58346000	-0.77315600
C	-0.09201100	-1.84555000	-2.78548500
H	0.91150100	-1.46432100	-2.62060500
H	-0.07384700	-2.91923100	-2.61246600
H	-0.36918600	-1.67330100	-3.81874700
O	-4.01749200	0.89044000	-2.08756000
H	-3.94603800	0.92676100	-3.04857200
O	4.76250000	1.12665600	-0.60285400
H	5.51955200	0.56508000	-0.78391900
C	2.33215400	2.52038100	0.07223600
C	3.23666800	2.97234500	1.22886800
H	4.28040700	2.76707400	1.02616300
H	3.12019400	4.04364200	1.37666800
H	2.95288100	2.46831400	2.15037700
C	0.90175100	2.94203600	0.41649200
H	0.20067600	2.67513700	-0.37318400
H	0.56598700	2.50459200	1.35523600
H	0.87860000	4.02237000	0.52911200
C	2.72738300	3.24386400	-1.22394200
H	3.75129800	3.03251900	-1.50580500
H	2.07111700	2.94635500	-2.03928600
H	2.62559400	4.31721100	-1.07934900
C	-4.06215900	0.66876700	0.77484800

C	-5.46991400	0.15004400	0.42776700
H	-6.17696800	0.58800800	1.12742700
H	-5.77558700	0.42224500	-0.57450600
H	-5.51742200	-0.93115100	0.53311700
C	-4.00053600	2.19943500	0.61349300
H	-4.26192600	2.52000200	-0.38704000
H	-4.70831600	2.64492800	1.30771700
H	-3.00775400	2.57050700	0.85837100
C	-3.79627900	0.34468300	2.24600600
H	-3.85582800	-0.72336200	2.44252400
H	-2.82854400	0.71602400	2.57636800
H	-4.55846900	0.83276600	2.84563200

SET-PT (PT step) mechanism

M05-2X/6-311++G(2d,2p)											
SW $^{\bullet+}$				SW (O1$^{\bullet}$)				SW (O2$^{\bullet}$)			
C	2.44020600	1.00898900	-0.11449800	C	2.54453	0.94961	-0.12528	C	2.64317	0.93580	-0.07473
C	1.34862700	0.16872100	0.04606400	C	1.39844	0.16937	-0.01904	C	1.49030	0.19437	-0.02724
C	1.40466300	-1.22021000	-0.08706300	C	1.38774	-1.21822	-0.11223	C	1.44964	-1.20022	-0.20084
C	2.61629800	-1.82722600	-0.42868000	C	2.58585	-1.88568	-0.36109	C	2.64359	-1.91203	-0.48988
C	3.71943800	-1.00906400	-0.59021900	C	3.74227	-1.12882	-0.48073	C	3.80943	-1.20981	-0.55818
C	3.64743100	0.37443900	-0.43824800	C	3.73389	0.25304	-0.35952	C	3.88787	0.21914	-0.35027
H	0.40503800	0.61291200	0.30995900	H	0.45631	0.66302	0.14355	H	0.55050	0.68734	0.15025
H	4.67382200	-1.45251500	-0.83707100	H	4.68229	-1.62751	-0.67532	H	4.74640	-1.69755	-0.77655
C	0.15948500	-2.05370900	0.17312400	C	0.09244	-1.98453	0.11056	C	0.13884	-1.93271	-0.02597
H	0.27032800	-2.97140200	-0.40686700	H	0.16632	-2.91367	-0.45391	H	0.18809	-2.83599	-0.63226
C	-0.99814900	-1.32715100	-0.43426100	C	-1.08526	-1.24123	-0.47733	C	-1.04293	-1.13016	-0.54398
C	-1.07112900	-1.18410100	-1.86730800	C	-1.17724	-1.10579	-1.89245	C	-1.12811	-0.83729	-1.90813
C	-1.99451700	-0.74412600	0.36708300	C	-2.07424	-0.67076	0.33823	C	-2.05543	-0.67807	0.29436
C	-2.09554400	-0.44508800	-2.38270000	C	-2.21307	-0.38759	-2.40565	C	-2.21207	-0.10008	-2.35634
C	-3.02713600	0.01692300	-0.12316100	C	-3.13133	0.06759	-0.14046	C	-3.15084	0.07005	-0.13108
H	-1.92575000	-0.89491300	1.42671600	H	-1.98882	-0.81680	1.39832	H	-1.99122	-0.91835	1.33833
C	-3.05213000	0.16539800	-1.55317800	C	-3.22419	0.24282	-1.58757	C	-3.20208	0.35286	-1.49685
H	-2.18842900	-0.32074400	-3.45180400	H	-2.33036	-0.25509	-3.46982	H	-2.29513	0.12807	-3.41051
C	-0.01147600	-2.47599800	1.64502300	C	-0.07291	-2.39226	1.59059	C	-0.01120	-2.42075	1.43540
H	-1.00613200	-2.90511700	1.75578900	H	-1.06727	-2.81789	1.70971	H	-0.99877	-2.86769	1.52357
H	0.68392000	-3.29237100	1.82763700	H	0.62681	-3.20269	1.78692	H	0.70300	-3.22680	1.59256
C	0.24081400	-1.39399100	2.70883900	C	0.17406	-1.29195	2.63823	C	0.21159	-1.36786	2.53559
H	-0.41573100	-1.58414100	3.55539100	H	-0.52448	-1.42806	3.46207	H	-0.50165	-1.54155	3.33944
H	-0.02184100	-0.40843500	2.32939100	H	-0.03466	-0.31207	2.21626	H	0.00533	-0.37128	2.15389
C	1.68540300	-1.36625500	3.20225300	C	1.59833	-1.29998	3.18856	C	1.62490	-1.40212	3.11196
H	1.81965000	-0.60082700	3.96114700	H	1.73198	-0.51968	3.93352	H	1.75174	-0.64438	3.88085
H	1.95189300	-2.32399000	3.64275700	H	1.81735	-2.25569	3.66051	H	1.82874	-2.37265	3.55946
H	2.37724500	-1.16126800	2.38987700	H	2.32361	-1.13869	2.39533	H	2.36919	-1.22523	2.33983
C	2.75181600	-3.31423800	-0.60854400	C	2.65483	-3.38027	-0.52258	C	2.63591	-3.39561	-0.74216
H	2.40174200	-3.85557400	0.26669200	H	2.26893	-3.89782	0.35157	H	2.20895	-3.94585	0.09173
H	2.18038800	-3.66299700	-1.46722400	H	2.07159	-3.70526	-1.38265	H	2.05311	-3.63666	-1.62922
H	3.78893200	-3.58346000	-0.77315600	H	3.68057	-3.69880	-0.67547	H	3.64876	-3.74996	-0.89894
C	-0.09201100	-1.84555000	-2.78548500	C	-0.17485	-1.75075	-2.80965	C	-0.09187	-1.30100	-2.89558
H	0.91150100	-1.46432100	-2.62060500	H	0.82922	-1.38083	-2.61979	H	0.86792	-0.82113	-2.71788
H	-0.07384700	-2.91923100	-2.61246600	H	-0.16118	-2.83065	-2.67349	H	0.06395	-2.37584	-2.82867
H	-0.36918600	-1.67330100	-3.81874700	H	-0.43288	-1.54579	-3.84290	H	-0.40464	-1.07121	-3.90887
O	-4.01749200	0.89044000	-2.08756000	O	-4.13168	0.89699	-2.13056	O	-4.24995	1.08223	-1.99606
H	-3.94603800	0.92676100	-3.04857200	O	4.90743	0.94975	-0.48083	H	-4.13055	1.19424	-2.94042
O	4.76250000	1.12665600	-0.60285400	H	5.62272	0.33341	-0.64561	O	4.99193	0.78616	-0.41978
H	5.51955200	0.56508000	-0.78391900	C	2.49985	2.46996	0.01460	C	2.66084	2.43505	0.15436
C	2.33215400	2.52038100	0.07223600	C	3.36893	2.91208	1.20158	C	3.54150	2.75693	1.37317

C	3.23666800	2.97234500	1.22886800	H	4.40698	2.63642	1.06161	H	4.56372	2.43955	1.20896
H	4.28040700	2.76707400	1.02616300	H	3.30863	3.99362	1.30909	H	3.52702	3.83123	1.54762
H	3.12019400	4.04364200	1.37666800	H	3.00641	2.45665	2.12105	H	3.15315	2.26211	2.26153
H	2.95288100	2.46831400	2.15037700	C	1.07765	2.96990	0.27759	C	1.25974	2.98314	0.42816
C	0.90175100	2.94203600	0.41649200	H	0.40341	2.72182	-0.53928	H	0.58692	2.80968	-0.40912
H	0.20067600	2.67513700	-0.37318400	H	0.67298	2.56461	1.20299	H	0.82461	2.54434	1.32391
H	0.56598700	2.50459200	1.35523600	H	1.10374	4.05279	0.37177	H	1.33165	4.05672	0.58408
H	0.87860000	4.02237000	0.52911200	C	2.99140	3.13840	-1.27739	C	3.21550	3.14815	-1.08936
C	2.72738300	3.24386400	-1.22394200	H	4.01524	2.86646	-1.50358	H	4.22979	2.83174	-1.29888
H	3.75129800	3.03251900	-1.50580500	H	2.35868	2.84921	-2.11368	H	2.59073	2.93977	-1.95579
H	2.07111700	2.94635500	-2.03928600	H	2.93684	4.21997	-1.16626	H	3.20792	4.22252	-0.91492
H	2.62559400	4.31721100	-1.07934900	C	-4.16802	0.69660	0.77042	C	-4.22509	0.55123	0.84270
C	-4.06215900	0.66876700	0.77484800	C	-5.56474	0.15464	0.42437	C	-5.59284	-0.02904	0.45423
C	-5.46991400	0.15004400	0.42776700	H	-6.29406	0.59767	1.10021	H	-6.34298	0.30875	1.16724
H	-6.17696800	0.58800800	1.12742700	H	-5.83380	0.39910	-0.59576	H	-5.89302	0.28619	-0.53757
H	-5.77558700	0.42224500	-0.57450600	H	-5.59517	-0.92569	0.55176	H	-5.56128	-1.11622	0.48060
H	-5.51742200	-0.93115100	0.53311700	C	-4.14750	2.22420	0.59427	C	-4.28618	2.08607	0.84263
C	-4.00053600	2.19943500	0.61349300	H	-4.39095	2.50013	-0.42428	H	-4.53964	2.47493	-0.13611
H	-4.26192600	2.52000200	-0.38704000	H	-4.87826	2.66921	1.26727	H	-5.04116	2.41715	1.55361
H	-4.70831600	2.64492800	1.30771700	H	-3.16586	2.62130	0.84646	H	-3.32661	2.50003	1.14563
H	-3.00775400	2.57050700	0.85837100	C	-3.89561	0.38845	2.24330	C	-3.93009	0.10572	2.27658
C	-3.79627900	0.34468300	2.24600600	H	-3.92722	-0.68080	2.44269	H	-3.90846	-0.97844	2.36545
H	-3.85582800	-0.72336200	2.44252400	H	-2.93256	0.77851	2.56701	H	-2.98584	0.50736	2.63873
H	-2.82854400	0.71602400	2.57636800	H	-4.66666	0.86276	2.84516	H	-4.72067	0.47730	2.92386
H	-4.55846900	0.83276600	2.84563200								
**SW (C1·)**				**SW (C2·)**				**SW (C3·)**			
C	2.48992	1.03468	-0.07260	C	2.64399	0.91281	-0.09449	C	3.00310	0.67070	0.27789
C	1.46783	0.12639	-0.01496	C	1.47877	0.15030	-0.02672	C	1.70707	0.19251	0.16116
C	1.47790	-1.23473	-0.17024	C	1.42957	-1.23122	-0.18446	C	1.35108	-0.98581	-0.51095
C	2.73423	-1.78543	-0.45110	C	2.61589	-1.92723	-0.46464	C	2.36581	-1.72110	-1.13570
C	3.83338	-0.93592	-0.53153	C	3.73198	-1.14295	-0.53212	C	3.66968	-1.25961	-1.02931
C	3.72928	0.43802	-0.34376	C	3.82948	0.21320	-0.36274	C	3.99500	-0.10557	-0.33510
H	4.80522	-1.35569	-0.75192	H	0.54588	0.65414	0.15264	H	0.90450	0.74301	0.62086
C	0.21115	-2.05153	-0.00746	C	0.11892	-1.98149	-0.00298	H	4.46194	-1.80672	-1.52412
H	0.31076	-2.94592	-0.62133	H	0.17633	-2.88258	-0.61250	C	-0.05509	-1.39695	-0.47752
C	-0.98189	-1.27470	-0.54012	C	-1.06277	-1.18638	-0.53063	C	-1.10586	-0.43022	-0.69054
C	-1.08648	-1.02907	-1.91208	C	-1.17104	-0.94571	-1.90361	C	-0.99858	0.64198	-1.60746
C	-1.96917	-0.77153	0.29804	C	-2.04697	-0.67705	0.30816	C	-2.32164	-0.55921	0.01294
C	-2.15871	-0.27956	-2.36767	C	-2.24199	-0.19399	-2.35859	C	-2.06603	1.51645	-1.71583
C	-3.05013	-0.00602	-0.13414	C	-3.13013	0.08611	-0.12442	C	-3.39382	0.31125	-0.08592
H	-1.89147	-0.97856	1.34791	H	-1.96809	-0.88217	1.35862	H	-2.40712	-1.38036	0.69856
C	-3.11811	0.23262	-1.50680	C	-3.19979	0.32148	-1.49768	C	-3.23104	1.37982	-0.97542
H	-2.25454	-0.08474	-3.42764	H	-2.33996	-0.00274	-3.41897	H	-2.00371	2.32728	-2.42982
C	0.05783	-2.53537	1.44938	C	-0.03775	-2.46606	1.45494	C	-0.39640	-2.75470	0.06641
H	-0.92639	-2.98915	1.54629	H	-1.03236	-2.89576	1.55485	H	-1.40210	-3.02463	-0.24607
H	0.77969	-3.33438	1.61290	H	0.66344	-3.28441	1.61007	H	0.27073	-3.50265	-0.35494
C	0.27376	-1.46566	2.53586	C	0.21149	-1.41755	2.55403	C	-0.28862	-2.86748	1.60396
H	-0.45779	-1.60806	3.32967	H	-0.49556	-1.58101	3.36581	H	-0.84604	-3.74998	1.91556
H	0.09685	-0.47539	2.12178	H	0.01773	-0.41821	2.17298	H	-0.76850	-2.00859	2.06930
C	1.67627	-1.50546	3.13717	C	1.63084	-1.46690	3.11492	C	1.14822	-2.98414	2.10422
H	1.79560	-0.74271	3.90268	H	1.76921	-0.71996	3.89278	H	1.16932	-3.13430	3.18069
H	1.86769	-2.47414	3.59497	H	1.83441	-2.44431	3.54813	H	1.64472	-3.83329	1.63751
H	2.43045	-1.33706	2.37222	H	2.36451	-1.28281	2.33441	H	1.72515	-2.09387	1.87428
C	2.90589	-3.26240	-0.68556	C	2.67322	-3.41318	-0.70075	C	2.09619	-2.93981	-1.97662
H	2.55423	-3.84244	0.16384	H	2.28346	-3.96521	0.14985	H	2.17095	-3.85558	-1.39305
H	2.34180	-3.58322	-1.55969	H	2.08414	-3.68568	-1.57438	H	1.10144	-2.89864	-2.41053
H	3.95077	-3.50329	-0.85176	H	3.69722	-3.72550	-0.87083	H	2.82189	-3.00441	-2.78231
C	-0.08176	-1.55996	-2.89753	C	-0.17427	-1.48810	-2.89073	C	0.16741	0.83157	-2.54159
H	0.91248	-1.17085	-2.69182	H	0.82517	-1.11161	-2.68793	H	1.00524	1.32774	-2.05782

H	-0.02483	-2.64624	-2.85089	H	-0.13013	-2.57492	-2.84205	H	0.53178	-0.12463	-2.90503
H	-0.35902	-1.28308	-3.90954	H	-0.45119	-1.20999	-3.90245	H	-0.14152	1.43141	-3.39262
O	-4.15242	0.97984	-2.01412	O	-4.23458	1.06694	-2.00524	O	-4.24440	2.28914	-1.13304
H	-4.04227	1.05598	-2.96299	H	-4.13015	1.13536	-2.95541	H	-3.97112	2.95165	-1.76948
O	4.84100	1.23419	-0.42756	O	5.03117	0.86386	-0.46001	O	5.30418	0.29446	-0.26013
H	5.60874	0.68427	-0.59219	H	5.70647	0.21207	-0.65873	H	5.85096	-0.34246	-0.72259
C	2.26317	2.52431	0.14174	C	2.63578	2.42665	0.11735	C	3.32554	1.96303	1.02520
C	3.06876	3.01380	1.35081	C	3.51795	2.78746	1.32155	C	4.25733	1.67122	2.21046
H	4.13377	2.88340	1.19391	H	4.54691	2.48597	1.16631	H	5.19963	1.24935	1.88220
H	2.86678	4.07083	1.51540	H	3.49120	3.86438	1.47843	H	4.45962	2.59658	2.74696
H	2.77669	2.46591	2.24431	H	3.14254	2.30118	2.21962	H	3.78287	0.97442	2.89826
C	0.77786	2.77114	0.41228	C	1.22614	2.94678	0.40505	C	2.06249	2.62157	1.58275
H	0.16417	2.42485	-0.41695	H	0.54669	2.75162	-0.42153	H	1.36003	2.87968	0.79302
H	0.45491	2.25027	1.31160	H	0.81264	2.50628	1.30970	H	1.55835	1.98169	2.30310
H	0.60749	3.83675	0.55103	H	1.27849	4.02328	0.55060	H	2.34628	3.53968	2.09151
C	2.66966	3.30697	-1.11252	C	3.14633	3.14011	-1.14246	C	3.98661	2.97133	0.07335
H	3.72170	3.17103	-1.33785	H	4.16218	2.84927	-1.38164	H	4.91668	2.58965	-0.33001
H	2.08260	2.97921	-1.96760	H	2.50631	2.90722	-1.99080	H	3.31630	3.20372	-0.75188
H	2.48081	4.36719	-0.95302	H	3.12263	4.21654	-0.98138	H	4.19442	3.89321	0.61366
C	-4.08453	0.55024	0.84307	C	-4.17012	0.63481	0.85118	C	-4.67109	0.11947	0.73010
C	-5.48358	0.01562	0.50389	C	-5.56342	0.08730	0.50825	C	-5.87248	-0.07058	-0.20727
H	-6.20295	0.40707	1.22151	H	-6.28789	0.47154	1.22454	H	-6.77186	-0.22825	0.38572
H	-5.79353	0.31069	-0.49116	H	-5.87386	0.38037	-0.48723	H	-6.02440	0.79444	-0.84129
H	-5.49582	-1.07057	0.56567	H	-5.56568	-0.99897	0.56918	H	-5.72132	-0.94409	-0.83790
C	-4.08132	2.08534	0.79000	C	-4.18194	2.16982	0.79886	C	-4.90305	1.33230	1.64316
H	-4.34246	2.44966	-0.19620	H	-4.44160	2.53253	-0.18829	H	-5.01756	2.24570	1.07241
H	-4.80337	2.47369	1.50661	H	-4.91166	2.55015	1.51186	H	-5.80652	1.17423	2.22980
H	-3.09692	2.46672	1.05409	H	-3.20340	2.56164	1.06909	H	-4.06669	1.45114	2.32865
C	-3.77721	0.14283	2.28566	C	-3.86289	0.22903	2.29423	C	-4.59130	-1.11758	1.62711
H	-3.79503	-0.93767	2.41142	H	-3.87333	-0.85157	2.41963	H	-4.45891	-2.02840	1.04721
H	-2.81031	0.51862	2.61388	H	-2.89969	0.61155	2.62550	H	-3.78227	-1.04002	2.35028
H	-4.53902	0.56703	2.9354	H	-4.62918	0.64744	2.94239	H	-5.52458	-1.20521	2.17823
**SW (C4•)**				**SW (C5•)**				**SW (C6•)**			
C	2.63006	0.89351	-0.08737	C	2.63091	0.91598	-0.07202	C	2.49542	1.07179	0.04752
C	1.45932	0.14371	-0.05140	C	1.46128	0.16490	-0.02439	C	1.38406	0.23912	-0.04902
C	1.41589	-1.24165	-0.16561	C	1.41055	-1.21373	-0.20086	C	1.44935	-1.11347	-0.36079
C	2.60802	-1.93564	-0.36515	C	2.59309	-1.89758	-0.47861	C	2.69639	-1.68810	-0.60340
C	3.78960	-1.20942	-0.41566	C	3.77250	-1.16909	-0.54485	C	3.81936	-0.87858	-0.51876
C	3.81129	0.16947	-0.27230	C	3.80250	0.20177	-0.33975	C	3.73098	0.46875	-0.20057
H	0.52207	0.65638	0.07174	H	0.52924	0.67172	0.15363	H	0.40784	0.65847	0.11955
H	4.72470	-1.73046	-0.57247	H	4.69966	-1.68221	-0.76277	H	4.79534	-1.30626	-0.70714
C	0.09627	-1.97885	-0.01409	C	0.09374	-1.95453	-0.03540	C	0.18616	-1.96863	-0.40168
H	0.15853	-2.89132	-0.60612	H	0.14397	-2.85013	-0.65379	H	0.31566	-2.69701	-1.20145
C	-1.06662	-1.18372	-0.57010	C	-1.08213	-1.14815	-0.56119	C	-1.03923	-1.14031	-0.77469
C	-1.19668	-0.92470	-1.94477	C	-1.15208	-0.86770	-1.93914	C	-1.20987	-0.70183	-2.08797
C	-2.06483	-0.65646	0.20424	C	-2.09600	-0.67687	0.26572	C	-1.97034	-0.76509	0.18441
C	-2.27680	-0.17247	-2.38689	C	-2.23344	-0.12905	-2.31724	C	-2.30541	0.09775	-2.37286
C	-3.15822	0.09601	-0.14156	C	-3.18752	0.08119	-0.16567	C	-3.07267	0.04684	-0.07412
C	-3.23749	0.33535	-1.51853	H	-2.03678	-0.91004	1.31127	H	-1.81800	-1.12389	1.18641
H	-2.37950	0.02547	-3.44495	C	-3.24595	0.36362	-1.53487	C	-3.21741	0.47158	-1.39626
C	-0.14993	-2.41741	1.44533	C	-0.07982	-2.45210	1.41597	H	-2.45643	0.44600	-3.38612
H	-1.14804	-2.84900	1.48497	H	-1.07200	-2.89029	1.50126	C	0.02018	-2.74440	0.87108
H	0.54937	-3.21626	1.68788	H	0.62587	-3.26624	1.57306	H	-0.40450	-3.73416	0.80668
C	-0.01220	-1.31480	2.50580	C	0.14923	-1.40844	2.52385	C	0.36165	-2.20460	2.21601
H	-0.74817	-1.48879	3.28816	H	-0.56827	-1.57886	3.32501	H	-0.43839	-2.44361	2.91899
H	-0.25979	-0.34926	2.06975	H	-0.04463	-0.40817	2.14497	H	0.44104	-1.11986	2.17183
C	1.37702	-1.25429	3.13787	C	1.56078	-1.45494	3.10427	C	1.68071	-2.77155	2.76734
H	1.43014	-0.46758	3.88670	H	1.68611	-0.71001	3.88629	H	1.86650	-2.40468	3.77377
H	1.61003	-2.19807	3.62747	H	1.76108	-2.43311	3.53729	H	1.64525	-3.85787	2.79921

H	2.14407	-1.06288	2.39239	H	2.30431	-1.26630	2.33420	H	2.51055	-2.47297	2.13288
C	2.64651	-3.42958	-0.54557	C	2.62466	-3.38176	-0.72834	C	2.84326	-3.14392	-0.95136
H	2.20331	-3.94838	0.30003	H	2.21594	-3.94015	0.10941	H	2.36591	-3.77302	-0.20303
H	2.09778	-3.72751	-1.43749	H	2.04173	-3.64036	-1.61070	H	2.37761	-3.36493	-1.91063
H	3.67026	-3.77309	-0.65238	H	3.64354	-3.71773	-0.89089	H	3.89114	-3.41877	-1.01561
C	-0.19274	-1.45339	-2.93221	C	-0.13144	-1.34390	-2.93713	C	-0.24219	-1.07055	-3.17857
H	0.80052	-1.06424	-2.72219	H	0.83596	-0.88497	-2.75030	H	0.77014	-0.76898	-2.91899
H	-0.13509	-2.53930	-2.88189	H	-0.00267	-2.42245	-2.87405	H	-0.23283	-2.14656	-3.34416
H	-0.47038	-1.17631	-3.94401	H	-0.44986	-1.09660	-3.94350	H	-0.51717	-0.59044	-4.11203
O	-4.28269	1.07740	-2.00334	O	-4.27328	1.09686	-2.06851	O	-4.28038	1.27089	-1.73606
H	-4.19550	1.15607	-2.95478	H	-4.11996	1.17838	-3.01207	H	-4.22580	1.47777	-2.67019
O	5.00908	0.83693	-0.32401	O	4.99819	0.87081	-0.41062	O	4.87531	1.22324	-0.13177
H	5.71436	0.20140	-0.45517	H	5.69469	0.24268	-0.60740	H	5.62925	0.66016	-0.31311
C	2.61967	2.41240	0.07562	C	2.62991	2.42554	0.16199	C	2.36328	2.55284	0.39644
C	3.43640	2.81135	1.31407	C	3.49989	2.76621	1.38134	C	3.13174	2.86317	1.68966
H	4.47211	2.50712	1.22387	H	4.52922	2.46254	1.23418	H	4.18920	2.65301	1.58481
H	3.40132	3.89255	1.43676	H	3.47553	3.84099	1.55301	H	3.00938	3.91608	1.93761
H	3.01315	2.35197	2.20504	H	3.11179	2.26930	2.26825	H	2.73720	2.27061	2.51243
C	1.19989	2.94826	0.27226	C	1.22084	2.95084	0.44403	C	0.90315	2.95082	0.62321
H	0.56265	2.72853	-0.58161	H	0.54784	2.77250	-0.39169	H	0.29676	2.78792	-0.26517
H	0.73757	2.54001	1.16847	H	0.79572	2.49980	1.33813	H	0.46070	2.40252	1.45230
H	1.24988	4.02888	0.38445	H	1.27799	4.02463	0.60605	H	0.86688	4.01049	0.86498
C	3.19863	3.08724	-1.17631	C	3.15469	3.15905	-1.08071	C	2.90266	3.41625	-0.75351
H	4.22430	2.78673	-1.35307	H	4.17195	2.87018	-1.31519	H	3.95193	3.22120	-0.93977
H	2.60291	2.83084	-2.04995	H	2.52215	2.94228	-1.93898	H	2.34274	3.21853	-1.66508
H	3.17062	4.16811	-1.04872	H	3.13163	4.23275	-0.90203	H	2.78405	4.46856	-0.50084
C	-4.16005	0.60552	0.88581	C	-4.26020	0.58174	0.80194	C	-4.05203	0.45347	1.02520
C	-5.55943	0.05662	0.58295	C	-5.63116	0.01244	0.41003	C	-5.46434	-0.04946	0.69265
H	-6.25579	0.40646	1.34323	H	-6.38227	0.36371	1.11557	H	-6.14599	0.23328	1.49312
H	-5.91167	0.38601	-0.38800	H	-5.92143	0.32316	-0.58643	H	-5.82865	0.37137	-0.23663
H	-5.55035	-1.03098	0.60111	H	-5.61072	-1.07470	0.44462	H	-5.46587	-1.13421	0.60946
C	-4.18588	2.13874	0.88240	C	-4.30434	2.11664	0.78485	C	-4.06097	1.98163	1.18078
H	-4.49667	2.52546	-0.08176	H	-4.54953	2.49673	-0.19969	H	-4.38214	2.47236	0.27006
H	-4.88384	2.49068	1.64026	H	-5.05865	2.46394	1.48887	H	-4.74152	2.25943	1.98387
H	-3.19908	2.53283	1.11603	H	-3.34130	2.52327	1.08655	H	-3.06540	2.33839	1.43754
C	-3.74423	0.13062	2.27834	C	-3.97330	0.14486	2.23984	C	-3.66082	-0.14203	2.37899
H	-3.71595	-0.95591	2.32749	H	-3.96311	-0.93860	2.33691	H	-3.66270	-1.22954	2.35578
H	-2.75957	0.50936	2.54263	H	-3.02539	0.53905	2.60007	H	-2.67972	0.20011	2.70158
H	-4.46218	0.49148	3.01193	H	-4.76174	0.53040	2.88189	H	-4.38774	0.17865	3.12147
**SW (C7$^{\bullet}$)**				**SW (C8$^{\bullet}$)**				**SW (C9$^{\bullet}$)**			
C	2.54553	0.89773	-0.30515	C	2.51867	0.98540	-0.13759	C	2.64342	0.89659	0.04596
C	1.41437	0.13550	-0.03903	C	1.39873	0.16845	-0.02671	C	1.47317	0.14278	-0.04712
C	1.41839	-1.24988	0.07980	C	1.43509	-1.22060	-0.08424	C	1.42718	-1.19838	-0.38318
C	2.61591	-1.93741	-0.09898	C	2.65944	-1.84913	-0.30579	C	2.64324	-1.86964	-0.67556
C	3.75868	-1.20004	-0.38253	C	3.79031	-1.05606	-0.43612	C	3.83560	-1.11243	-0.59587
C	3.73454	0.18253	-0.47976	C	3.73367	0.32637	-0.34554	C	3.84088	0.21508	-0.24471
H	0.47190	0.63510	0.10023	H	0.43716	0.62955	0.11434	H	0.53367	0.62876	0.14797
H	4.69802	-1.71579	-0.53161	H	4.74883	-1.52667	-0.61086	H	4.77355	-1.60258	-0.81775
C	0.14375	-1.96194	0.49604	C	0.16122	-2.02237	0.13979	C	0.10732	-1.94636	-0.37093
H	0.21486	-2.99657	0.16636	H	0.25622	-2.94610	-0.42830	H	0.15627	-2.70516	-1.15034
C	-1.07577	-1.37159	-0.18352	C	-1.05054	-1.30114	-0.42433	C	-1.06371	-1.05211	-0.74035
C	-1.27119	-1.58857	-1.54984	C	-1.20792	-1.21030	-1.80972	C	-1.14452	-0.52298	-2.03238
C	-1.99673	-0.58699	0.49866	C	-1.99653	-0.68944	0.38956	C	-2.06999	-0.73799	0.16542
C	-2.36774	-1.00139	-2.16019	C	-2.29357	-0.50696	-2.30585	C	-2.21489	0.30178	-2.33995
C	-3.10007	0.02477	-0.09355	C	-3.09080	0.03197	-0.08440	C	-3.15124	0.09473	-0.11781
H	-1.83929	-0.43357	1.54827	H	-1.87542	-0.77265	1.45326	H	-2.01099	-1.16179	1.14946
C	-3.26235	-0.20536	-1.46006	C	-3.21484	0.10901	-1.47203	C	-3.19516	0.61661	-1.41081
H	-2.53356	-1.16387	-3.21705	H	-2.43053	-0.43275	-3.37661	H	-2.29414	0.71226	-3.33803
C	0.05592	-2.02432	2.05295	C	0.01130	-2.44770	1.61873	C	-0.06548	-2.70886	0.96045

H	-0.92268	-2.42488	2.31339	H	-1.01604	-2.76687	1.78032	H	-1.05537	-3.16035	0.95913
H	0.79778	-2.75981	2.36985	H	0.63468	-3.32247	1.79104	H	0.64854	-3.53084	0.96240
C	0.32368	-0.75649	2.79079	C	0.41263	-1.38364	2.66384	C	0.15369	-1.88367	2.24165
H	-0.49968	-0.16284	3.15156	H	-0.10261	-1.63419	3.59487	H	-0.57563	-2.18907	2.99038
C	1.69580	-0.41911	3.25402	H	0.05772	-0.40216	2.36178	H	-0.02888	-0.82956	2.04526
H	1.78953	0.63553	3.49876	C	1.87806	-1.33326	2.90434	C	1.55659	-2.04346	2.82312
H	1.95662	-0.98633	4.15468	H	2.46422	-0.46361	2.66779	H	1.66567	-1.46998	3.74024
H	2.43938	-0.66210	2.49621	H	2.38795	-2.21217	3.26286	H	1.75602	-3.08821	3.05307
C	2.70219	-3.43742	-0.00342	C	2.78345	-3.34434	-0.41606	H	2.31178	-1.70414	2.11932
H	2.35758	-3.79774	0.96272	H	2.43259	-3.83912	0.48578	C	2.71728	-3.22383	-1.02364
H	2.09068	-3.91321	-0.76806	H	2.19738	-3.72513	-1.25074	H	1.84787	-3.84956	-1.09296
H	3.72660	-3.76866	-0.13895	H	3.81813	-3.62975	-0.57551	H	3.67346	-3.67250	-1.22886
C	-0.33314	-2.43886	-2.36130	C	-0.24000	-1.85501	-2.76349	C	-0.11473	-0.81997	-3.08785
H	0.68473	-2.06022	-2.30481	H	0.77052	-1.48687	-2.60235	H	0.83671	-0.34963	-2.85077
H	-0.32354	-3.46480	-1.99670	H	-0.22159	-2.93527	-2.62914	H	0.07012	-1.88848	-3.17235
H	-0.63863	-2.45675	-3.40255	H	-0.52344	-1.65029	-3.79081	H	-0.44820	-0.45668	-4.05469
O	-4.32267	0.36313	-2.12134	O	-4.26705	0.79955	-2.02014	O	-4.22765	1.44622	-1.77104
H	-4.27900	0.11499	-3.04592	H	-4.19906	0.76012	-2.97527	H	-4.10603	1.71416	-2.68303
O	4.89379	0.86304	-0.75734	O	4.88568	1.06151	-0.47269	O	5.02695	0.90220	-0.17820
H	5.61177	0.23348	-0.83859	H	5.62315	0.46383	-0.60401	H	5.74356	0.30369	-0.39462
C	2.48764	2.42157	-0.38785	C	2.41962	2.50581	-0.02897	C	2.61945	2.37036	0.43738
C	3.40491	3.04009	0.67766	C	3.25733	3.00157	1.15931	C	3.42753	2.58611	1.72650
H	4.43951	2.75808	0.52513	H	4.30598	2.75894	1.03759	H	4.46637	2.30823	1.59820
H	3.32953	4.12535	0.63292	H	3.15842	4.08243	1.24543	H	3.38018	3.63673	2.00805
H	3.09671	2.71643	1.66981	H	2.89866	2.55210	2.08337	H	3.00460	1.99599	2.53699
C	1.07213	2.94398	-0.13119	C	0.97782	2.96106	0.20700	C	1.19459	2.86137	0.70263
H	0.36356	2.57063	-0.86733	H	0.32232	2.66842	-0.61031	H	0.56522	2.77122	-0.17990
H	0.71892	2.67219	0.86166	H	0.57696	2.56155	1.13652	H	0.72779	2.31881	1.52189
H	1.08557	4.02945	-0.19783	H	0.96467	4.04624	0.27641	H	1.23773	3.91228	0.97855
C	2.91051	2.89568	-1.78562	C	2.90476	3.16665	-1.32716	C	3.19830	3.23067	-0.69659
H	3.92280	2.58903	-2.02067	H	3.93969	2.92391	-1.53565	H	4.22926	2.97396	-0.90700
H	2.23775	2.49001	-2.53816	H	2.29260	2.84000	-2.16505	H	2.61192	3.09951	-1.60356
H	2.85734	3.98228	-1.82950	H	2.81314	4.24797	-1.23804	H	3.15282	4.27991	-0.40949
C	-4.05656	0.90831	0.70493	C	-4.08749	0.70167	0.86020	C	-4.21738	0.41654	0.92819
C	-5.48341	0.34371	0.64667	C	-5.49471	0.12659	0.64041	C	-5.59618	-0.05660	0.44429
H	-6.14451	0.97250	1.24090	H	-6.19158	0.60309	1.32797	H	-6.34166	0.16435	1.20643
H	-5.85771	0.31169	-0.36923	H	-5.83955	0.29382	-0.37260	H	-5.88507	0.43647	-0.47583
H	-5.50656	-0.66251	1.05944	H	-5.49700	-0.94320	0.83863	H	-5.58635	-1.13152	0.27695
C	-4.03461	2.33881	0.14593	C	-4.09654	2.21967	0.62530	C	-4.24705	1.92773	1.20151
H	-4.35318	2.36536	-0.88935	H	-4.39422	2.46415	-0.38717	H	-4.48922	2.48928	0.30733
H	-4.70371	2.96715	0.73171	H	-4.79587	2.68819	1.31596	H	-4.99692	2.14304	1.96103
H	-3.03012	2.75208	0.21355	H	-3.10660	2.63280	0.80872	H	-3.27969	2.26063	1.57216
C	-3.65571	0.98738	2.17961	C	-3.72247	0.46927	2.32762	C	-3.93393	-0.28294	2.25928
H	-3.67412	0.00871	2.65454	H	-3.72893	-0.58848	2.58193	H	-3.93233	-1.36557	2.15255
H	-2.66583	1.42156	2.30513	H	-2.74630	0.88381	2.57061	H	-2.98249	0.02999	2.68419
H	-4.36796	1.62438	2.69855	H	-4.46122	0.96696	2.95116	H	-4.71850	-0.01775	2.96394
**SW (C10·)**				**SW (C11·)**				**SW (C12·)**			
C	2.67305	0.89692	-0.03908	C	2.62424	0.95259	-0.08633	C	2.60187	0.92512	-0.09563
C	1.49018	0.16580	-0.05431	C	1.46444	0.18916	-0.02355	C	1.43885	0.16600	-0.01914
C	1.42017	-1.20092	-0.29969	C	1.43177	-1.19194	-0.17901	C	1.39599	-1.21568	-0.17214
C	2.59651	-1.89066	-0.59042	C	2.62540	-1.86045	-0.45162	C	2.58036	-1.89464	-0.45507
C	3.78924	-1.18095	-0.59499	C	3.79514	-1.11709	-0.53008	C	3.75302	-1.15850	-0.54989
C	3.83807	0.17632	-0.31750	C	3.80730	0.25704	-0.34325	C	3.77511	0.21579	-0.36799
H	0.56293	0.67871	0.13140	H	0.53241	0.70274	0.15229	H	0.50567	0.66883	0.16392
H	4.71206	-1.69884	-0.81999	H	4.72853	-1.62100	-0.74308	H	4.68104	-1.66814	-0.77243
C	0.09202	-1.93199	-0.18122	C	0.12673	-1.95065	0.00178	C	0.08623	-1.96321	0.02140
H	0.12463	-2.78363	-0.86049	H	0.18940	-2.85325	-0.60492	H	0.13737	-2.86851	-0.58235
C	-1.08201	-1.08430	-0.63533	C	-1.06298	-1.16832	-0.52756	C	-1.10058	-1.17386	-0.50367
C	-1.17869	-0.69637	-2.00070	C	-1.17240	-0.92906	-1.90069	C	-1.21696	-0.93797	-1.87773

C	-2.08570	-0.68898	0.22904	C	-2.05573	-0.67203	0.30887	C	-2.08620	-0.66738	0.33424
C	-2.29668	0.07801	-2.38171	C	-2.25246	-0.19175	-2.35785	C	-2.29468	-0.19596	-2.33396
C	-3.19192	0.08626	-0.13431	C	-3.14868	0.07588	-0.12577	C	-3.17475	0.08412	-0.10192
H	-2.01389	-0.99697	1.25419	H	-1.97589	-0.87522	1.35960	H	-2.01910	-0.85725	1.39152
C	-3.26678	0.45994	-1.48601	C	-3.21898	0.31041	-1.49898	C	-3.25534	0.31558	-1.47351
H	-2.38833	0.37702	-3.41689	H	-2.35104	-0.00202	-3.41851	H	-2.39617	-0.01063	-3.39512
C	-0.07271	-2.52437	1.23560	C	-0.02865	-2.43230	1.46092	C	-0.06556	-2.43795	1.48306
H	-1.06600	-2.96340	1.30096	H	-1.01916	-2.87125	1.56162	H	-1.06002	-2.86599	1.59089
H	0.62899	-3.35045	1.33464	H	0.68002	-3.24334	1.62075	H	0.63593	-3.25545	1.64180
C	0.17090	-1.55479	2.40633	C	0.20907	-1.37659	2.55590	C	0.18832	-1.38139	2.57314
H	-0.54359	-1.76934	3.19968	H	-0.50005	-1.54030	3.36590	H	-0.51381	-1.54029	3.39001
H	-0.01586	-0.53073	2.09224	H	0.01062	-0.38043	2.16894	H	-0.01101	-0.38540	2.18635
C	1.58527	-1.64771	2.97458	C	1.62648	-1.41411	3.12263	C	1.61105	-1.42412	3.12604
H	1.71825	-0.95759	3.80410	H	1.75732	-0.66244	3.89729	H	1.75309	-0.67080	3.89716
H	1.78296	-2.65373	3.33981	H	1.83455	-2.38812	3.56140	H	1.81837	-2.39782	3.56593
H	2.32526	-1.41075	2.21458	H	2.36156	-1.22920	2.34353	H	2.33968	-1.24545	2.33954
C	2.61073	-3.36177	-0.91117	C	2.67721	-3.34694	-0.68386	C	2.61942	-3.38231	-0.68162
H	2.20682	-3.95555	-0.09584	H	2.28064	-3.90069	0.16273	H	2.22609	-3.92964	0.17066
H	2.01529	-3.57777	-1.79675	H	2.09292	-3.62360	-1.55988	H	2.02590	-3.65859	-1.55154
H	3.62469	-3.69823	-1.10122	H	3.69992	-3.67032	-0.84802	H	3.63839	-3.71373	-0.85298
C	-0.23227	-1.05363	-2.96813	C	-0.16763	-1.45942	-2.88627	C	-0.22040	-1.47809	-2.86648
H	-0.36837	-0.74415	-3.98970	H	0.82679	-1.07058	-2.68224	H	0.77658	-1.09136	-2.67116
H	0.65492	-1.60602	-2.72277	H	-0.10984	-2.54559	-2.83645	H	-0.16590	-2.56407	-2.80982
O	-4.33200	1.21548	-1.91036	H	-0.44695	-1.18566	-3.89858	H	-0.50537	-1.20990	-3.87868
H	-4.23444	1.38783	-2.84811	O	-4.26307	1.04188	-2.00882	O	-4.30010	1.04948	-1.97805
O	5.04686	0.82619	-0.32694	H	-4.15759	1.11089	-2.95880	H	-4.20181	1.11521	-2.92907
H	5.73640	0.19543	-0.53890	O	4.99208	0.94489	-0.42118	O	4.96469	0.89272	-0.46742
C	2.69219	2.39374	0.26526	H	5.69995	0.32551	-0.60488	H	5.66308	0.26610	-0.66224
C	3.53987	2.66334	1.51740	C	2.58205	2.47281	0.12767	C	2.59215	2.43840	0.11269
H	4.56661	2.34676	1.37966	C	3.43410	2.84784	1.35227	C	3.47786	2.80735	1.31227
H	3.53091	3.72986	1.73635	H	4.46948	2.56132	1.21266	H	4.50748	2.51042	1.15383
H	3.12276	2.13517	2.37258	H	3.38402	3.92297	1.51099	H	3.44637	3.88456	1.46676
C	1.28616	2.93046	0.54037	H	3.04863	2.34973	2.23928	H	3.10823	2.32165	2.21316
H	0.62989	2.80215	-0.31746	C	1.18625	2.94505	0.37716	C	1.18329	2.95747	0.40716
H	0.83353	2.44623	1.40305	H	0.52263	3.15349	-0.44550	H	0.49932	2.75972	-0.41519
H	1.35756	3.99453	0.75284	H	0.79771	3.00633	1.37998	H	0.77491	2.51808	1.31481
C	3.25676	3.17307	-0.93121	C	3.10386	3.19793	-1.12551	H	1.23424	4.03429	0.55043
H	4.27513	2.87905	-1.15419	H	4.12988	2.92366	-1.33924	C	3.09182	3.15416	-1.15061
H	2.64229	3.00284	-1.81269	H	2.48647	2.94653	-1.98501	H	4.10722	2.86808	-1.39649
H	3.24473	4.23830	-0.70617	H	3.05194	4.27291	-0.96681	H	2.44719	2.91795	-1.99462
C	-4.24949	0.50243	0.88316	C	-4.19803	0.61002	0.84786	H	3.06396	4.23066	-0.99015
C	-5.62269	-0.05924	0.48378	C	-5.58325	0.04450	0.50150	C	-4.21414	0.62085	0.89378
H	-6.36133	0.23516	1.22744	H	-6.31456	0.41953	1.21575	C	-5.60388	0.05026	0.56208
H	-5.93919	0.31064	-0.48365	H	-5.89467	0.33322	-0.49495	H	-6.32803	0.42315	1.28346
H	-5.58774	-1.14603	0.44845	H	-5.57154	-1.04172	0.56271	H	-5.91949	0.33925	-0.43314
C	-4.31567	2.03521	0.97556	C	-4.23007	2.14480	0.79669	H	-5.58491	-1.03564	0.62218
H	-4.58523	2.48024	0.02580	H	-4.49255	2.50488	-0.19069	C	-4.24493	2.15864	0.83709
H	-5.06062	2.31943	1.71691	H	-4.96606	2.51505	1.50857	H	-4.51768	2.50869	-0.15119
H	-3.35283	2.43382	1.28838	H	-3.25722	2.54901	1.06909	H	-4.97074	2.53209	1.55647
C	-3.93008	-0.02496	2.28382	C	-3.88878	0.20738	2.29138	H	-3.26704	2.56013	1.09392
H	-3.90533	-1.11226	2.30891	H	-3.88447	-0.87338	2.41580	C	-3.88092	0.22456	2.29554
H	-2.98033	0.35676	2.65282	H	-2.93176	0.60294	2.62517	H	-4.24070	-0.70493	2.70451
H	-4.71091	0.30694	2.96376	H	-4.66243	0.61465	2.93793	H	-3.22711	0.83359	2.89772

SPLET (SP step) mechanism

M05-2X/6-311++G(2d,2p)											
SW				SW ($O1^{-}$)				SW ($O2^{-}$)			
C	-2.61591700	-0.93080400	-0.09151600	C	2.47510	1.00935	-0.08941	C	2.69290	0.89911	-0.03719
C	-1.45603100	-0.16633800	-0.02152400	C	1.37071	0.16425	-0.04054	C	1.51439	0.17432	-0.00456
C	-1.42088400	1.21609400	-0.16983100	C	1.43482	-1.21870	-0.17313	C	1.42801	-1.20130	-0.23201
C	-2.61061200	1.89040200	-0.44112500	C	2.67622	-1.80385	-0.41698	C	2.61263	-1.88045	-0.54195
C	-3.78048900	1.14894500	-0.52924500	C	3.79452	-0.98212	-0.48367	C	3.80581	-1.18607	-0.58961
C	-3.79464400	-0.22607100	-0.35190000	C	3.70666	0.39029	-0.31327	C	3.93273	0.21611	-0.33871
H	-0.51893700	-0.66543400	0.15158000	H	0.38814	0.58462	0.08845	H	0.59201	0.69495	0.20316
H	-4.71258800	1.65488200	-0.74306000	H	4.76509	-1.42177	-0.67817	H	4.72302	-1.70454	-0.83121
C	-0.11285800	1.96892600	0.01586300	C	0.16290	-2.04514	-0.02366	C	0.10704	-1.93474	-0.08096
H	-0.17277600	2.87590900	-0.58452700	H	0.29053	-2.93777	-0.63630	H	0.13890	-2.80788	-0.73571
C	1.07312000	1.18558200	-0.52056300	C	-1.05702	-1.30835	-0.54344	C	-1.06716	-1.10565	-0.57467
C	1.17816600	0.95280100	-1.89525100	C	-1.19316	-1.07336	-1.92063	C	-1.13969	-0.74296	-1.92558
C	2.06556900	0.68020800	0.31090000	C	-2.02451	-0.75179	0.29562	C	-2.08931	-0.68613	0.26789
C	2.25321200	0.21198900	-2.35861600	C	-2.24222	-0.30874	-2.38668	C	-2.22155	0.01125	-2.35030
C	3.15346600	-0.07146400	-0.13008800	C	-3.08547	0.02812	-0.13923	C	-3.18026	0.08700	-0.13395
H	1.98958700	0.87908400	1.36277600	H	-1.93988	-0.93859	1.35272	H	-2.02912	-0.96962	1.30138
C	3.21898200	-0.29989800	-1.50459800	C	-3.23544	0.29233	-1.55117	C	-3.22059	0.42613	-1.48291
H	2.34817900	0.02697200	-3.42042900	H	-2.35366	-0.13477	-3.44788	H	-2.29062	0.29161	-3.39397
C	0.04623100	2.44023500	1.47791800	C	0.02222	-2.55506	1.43017	C	-0.08148	-2.49416	1.34558
H	1.03802800	2.87597700	1.58010000	H	-0.97141	-2.98802	1.51966	H	-1.07978	-2.92595	1.42760
H	-0.66012600	3.25199600	1.64409200	H	0.73197	-3.36906	1.58373	H	0.61831	-3.32022	1.46402
C	-0.19260800	1.37836300	2.56664400	C	0.25167	-1.51815	2.54408	C	0.17386	-1.49589	2.48850
H	0.51679200	1.53647400	3.37749800	H	-0.45201	-1.70427	3.35451	H	-0.52429	-1.69546	3.30273
H	0.00534500	0.38438600	2.17376500	H	0.03394	-0.52119	2.17237	H	-0.02121	-0.48251	2.14625
C	-1.60993200	1.41359800	3.13365900	C	1.67246	-1.54239	3.10387	C	1.60309	-1.56041	3.02225
H	-1.74158100	0.65693900	3.90331600	H	1.79365	-0.80941	3.89909	H	1.75252	-0.84486	3.82832
H	-1.81668700	2.38491900	3.57893500	H	1.90475	-2.52482	3.51356	H	1.81929	-2.55583	3.40909
H	-2.34528600	1.23481300	2.35343000	H	2.39855	-1.32008	2.32585	H	2.31389	-1.33691	2.23143
C	-2.65844700	3.37849600	-0.66311100	C	2.83039	-3.28561	-0.63262	C	2.61347	-3.35963	-0.84778
H	-2.26245800	3.92551500	0.18814300	H	2.46687	-3.85359	0.21991	H	2.20599	-3.94914	-0.02806
H	-2.07181500	3.66007900	-1.53599700	H	2.26361	-3.60762	-1.50466	H	2.01857	-3.58306	-1.73429
H	-3.68011500	3.70530100	-0.82707300	H	3.87416	-3.54087	-0.79191	H	3.62826	-3.69854	-1.03340
C	0.17385500	1.49312200	-2.87588100	C	-0.21131	-1.65875	-2.90693	C	-0.08398	-1.14329	-2.91743
H	-0.82101300	1.10368500	-2.67505300	H	0.80650	-1.32477	-2.70911	H	0.86148	-0.65931	-2.68559
H	0.11731900	2.57883600	-2.81620500	H	-0.21022	-2.74884	-2.86449	H	0.09626	-2.21601	-2.88592
H	0.45268900	1.22808300	-3.89065300	H	-0.47854	-1.36397	-3.91740	H	-0.39040	-0.87652	-3.92520
O	4.25765500	-1.03458500	-2.02058700	O	-4.17187	0.99503	-2.04201	O	-4.26958	1.18159	-1.97253
H	4.14890800	-1.09937700	-2.97050300	O	4.85541	1.15420	-0.38361	H	-4.09422	1.36405	-2.89640
O	-4.98170000	-0.90840200	-0.44429400	H	5.59227	0.56581	-0.55093	O	5.05926	0.79590	-0.39243
H	-5.68479800	-0.28457500	-0.63106500	C	2.33371	2.52071	0.08101	C	2.72233	2.39821	0.23913
C	-2.59694400	-2.44476700	0.11110700	C	3.14589	2.99128	1.29669	C	3.61853	2.67662	1.45556
C	-3.47069200	-2.82251100	1.31674000	H	4.20200	2.77915	1.17677	H	4.61212	2.28193	1.27300
H	-4.50312100	-2.53032900	1.16817300	H	3.01633	4.06545	1.42482	H	3.67599	3.75146	1.63935
H	-3.43249400	-3.90010400	1.46696400	H	2.78871	2.49419	2.19653	H	3.19981	2.19715	2.33982
H	-3.09584600	-2.33830200	2.21627100	C	0.87695	2.92670	0.31450	C	1.33665	2.96955	0.54161
C	-1.18308600	-2.95783400	0.39204400	H	0.23733	2.64833	-0.51934	H	0.65291	2.82755	-0.29372
H	-0.50674600	-2.75355300	-0.43503000	H	0.47483	2.47512	1.21867	H	0.90018	2.50956	1.42694
H	-0.76952500	-2.51994500	1.29807600	H	0.83306	4.00802	0.42908	H	1.42936	4.03940	0.72721
H	-1.22739300	-4.03542700	0.53160000	C	2.81778	3.24391	-1.18424	C	3.28062	3.14292	-0.98269
C	-3.10365500	-3.15850500	-1.15049400	H	3.86111	3.03257	-1.38867	H	4.26181	2.75225	-1.22957
H	-4.12277000	-2.87717000	-1.38634400	H	2.22177	2.93376	-2.03954	H	2.61512	3.00751	-1.83458
H	-2.46779400	-2.91557700	-1.99924400	H	2.69707	4.31900	-1.05497	H	3.35308	4.21094	-0.76770
H	-3.06848700	-4.23542700	-0.99431600	C	-4.09655	0.62248	0.83544	C	-4.25478	0.54196	0.85260
C	4.20254700	-0.61554100	0.83832700	C	-5.50597	0.11129	0.50076	C	-5.62683	-0.00983	0.43950

C	5.58976100	-0.05557200	0.49092200	H	-6.23936	0.57375	1.16472	H	-6.38120	0.31554	1.15551
H	6.32084400	-0.43721600	1.20188800	H	-5.74292	0.35119	-0.53010	H	-5.91140	0.33617	-0.54714
H	5.89739100	-0.34177600	-0.50743100	H	-5.55062	-0.96875	0.63657	H	-5.60480	-1.09777	0.43438
H	5.58370200	1.03042100	0.55666700	C	-4.07538	2.15469	0.72442	C	-4.30320	2.07629	0.90706
C	4.22645800	-2.15021400	0.78020100	H	-4.27881	2.44576	-0.30046	H	-4.54663	2.49804	-0.06085
H	4.48522900	-2.50730200	-0.20921900	H	-4.82602	2.58920	1.38788	H	-5.05813	2.39229	1.62666
H	4.96189900	-2.52735700	1.48902700	H	-3.09588	2.53311	1.01621	H	-3.33932	2.46785	1.22474
H	3.25207200	-2.55071000	1.05267300	C	-3.80312	0.25765	2.29127	C	-3.96981	0.04495	2.27149
C	3.89917400	-0.21779400	2.28445000	H	-3.83808	-0.81942	2.44800	H	-3.95429	-1.04171	2.32018
H	3.90062700	0.86238800	2.41369300	H	-2.82634	0.62179	2.60726	H	-3.02136	0.42280	2.64646
H	2.94115100	-0.61010200	2.61923500	H	-4.55787	0.71681	2.92930	H	-4.76008	0.40119	2.92947
H	4.67250200	-0.63176600	2.92710800								
**SW (C1^{-})**				**SW (C2^{-})**				**SW (C3^{-})**			
C	2.72393	0.82363	-0.04300	C	2.68782	0.87477	-0.06017	C	3.06329	0.63006	0.27154
C	1.45963	0.21446	0.02602	C	1.48473	0.16765	-0.02031	C	1.75565	0.19312	0.11543
C	1.40149	-1.17075	-0.20171	C	1.42795	-1.20416	-0.22440	C	1.35265	-1.00603	-0.52010
C	2.52249	-1.94678	-0.51218	C	2.61525	-1.90068	-0.51870	C	2.40751	-1.79672	-1.04184
C	3.75305	-1.30527	-0.58814	C	3.85323	-1.25890	-0.57693	C	3.72236	-1.38690	-0.86297
C	3.85202	0.05836	-0.35502	C	3.82723	0.10896	-0.32917	C	4.06507	-0.21118	-0.22123
H	4.64582	-1.86992	-0.83255	H	0.56388	0.69323	0.16962	H	0.95903	0.79157	0.52214
C	0.05471	-1.88081	-0.04029	C	0.10005	-1.93438	-0.06039	H	4.51688	-1.99492	-1.28263
H	0.05712	-2.78325	-0.65688	H	0.13560	-2.81739	-0.69874	C	-0.05151	-1.36157	-0.57530
C	-1.10569	-1.04772	-0.55054	C	-1.07846	-1.11723	-0.55862	C	-1.07281	-0.35692	-0.65175
C	-1.16784	-0.69347	-1.90217	C	-1.16704	-0.79287	-1.91834	C	-0.95936	0.84289	-1.42138
C	-2.15130	-0.64832	0.27132	C	-2.08936	-0.67243	0.28443	C	-2.34164	-0.54255	-0.03289
C	-2.25997	0.03182	-2.35047	C	-2.24960	-0.04388	-2.35024	C	-2.01216	1.74365	-1.44169
C	-3.25080	0.10021	-0.15087	C	-3.18236	0.09397	-0.12543	C	-3.40717	0.34892	-0.07386
H	-2.09478	-0.91357	1.30940	H	-2.01796	-0.92988	1.32402	H	-2.47821	-1.44173	0.53478
C	-3.27991	0.43018	-1.50165	C	-3.23604	0.39905	-1.48209	C	-3.21047	1.53418	-0.78114
H	-2.31707	0.30681	-3.39682	H	-2.33003	0.20856	-3.40022	H	-1.91095	2.63797	-2.04793
C	-0.12439	-2.36855	1.41201	C	-0.06977	-2.46644	1.37828	C	-0.47148	-2.74547	-0.13444
H	-1.10898	-2.82718	1.51246	H	-1.06507	-2.90170	1.47367	H	-1.47096	-2.95563	-0.52003
H	0.59991	-3.16577	1.57986	H	0.63620	-3.28523	1.50445	H	0.17073	-3.51371	-0.56284
C	0.08705	-1.28882	2.49041	C	0.18521	-1.44953	2.50534	C	-0.47720	-2.99192	1.39021
H	-0.67660	-1.38863	3.26387	H	-0.50952	-1.64120	3.32457	H	-1.01198	-3.92294	1.60072
H	-0.01449	-0.30799	2.03007	H	-0.01547	-0.44189	2.15011	H	-1.02106	-2.19015	1.88722
C	1.47023	-1.36772	3.13100	C	1.61665	-1.50144	3.03513	C	0.92806	-3.07782	1.97932
H	1.59979	-0.59019	3.88199	H	1.76863	-0.76594	3.82301	H	0.89829	-3.29834	3.04566
H	1.62216	-2.33464	3.61197	H	1.83334	-2.48685	3.44600	H	1.49987	-3.86480	1.48732
H	2.23585	-1.23364	2.37058	H	2.32638	-1.30275	2.23665	H	1.46390	-2.14481	1.83448
C	2.46418	-3.43133	-0.79136	C	2.56354	-3.38879	-0.80879	C	2.14836	-3.01337	-1.88285
H	2.00491	-3.98439	0.02530	H	2.12734	-3.96793	0.00617	H	2.03735	-3.92275	-1.29133
H	1.88427	-3.64434	-1.68975	H	1.98102	-3.60717	-1.70751	H	1.22244	-2.87345	-2.43991
H	3.46579	-3.82801	-0.94189	H	3.57990	-3.72915	-0.97046	H	2.96875	-3.17161	-2.58009
C	-0.07393	-1.04557	-2.86890	C	-0.12634	-1.23298	-2.90878	C	0.20676	1.10741	-2.32932
H	0.81088	-0.45068	-2.65255	H	0.83820	-0.78953	-2.67454	H	1.07105	1.52294	-1.81147
H	0.21713	-2.08946	-2.77650	H	0.01075	-2.31218	-2.87809	H	0.54143	0.16968	-2.77122
H	-0.39551	-0.86371	-3.89153	H	-0.41836	-0.95286	-3.91704	H	-0.08560	1.79661	-3.11916
O	-4.34035	1.16122	-2.01504	O	-4.28616	1.14888	-1.97897	O	-4.22410	2.49599	-0.86522
H	-4.13601	1.36103	-2.92904	H	-4.12732	1.29342	-2.91246	H	-3.87204	3.22418	-1.37669
O	5.11738	0.64291	-0.45210	O	5.06453	0.75328	-0.37118	O	5.41034	0.14325	-0.09863
H	5.73243	-0.05359	-0.68448	H	5.63964	0.00280	-0.57784	H	5.92329	-0.57096	-0.47654
C	2.87252	2.33223	0.22796	C	2.73892	2.38339	0.18407	C	3.39081	1.96087	0.94842
C	3.81017	2.57889	1.41966	C	3.61065	2.68602	1.41165	C	4.26169	1.73086	2.19179
H	4.80942	2.20399	1.22806	H	4.61734	2.31113	1.26910	H	5.19769	1.24962	1.93364
H	3.87044	3.64839	1.63002	H	3.64892	3.76306	1.58419	H	4.47651	2.68599	2.67240
H	3.41359	2.07847	2.30180	H	3.18375	2.20965	2.29277	H	3.73026	1.10064	2.90208
C	1.52215	2.95510	0.57656	C	1.35241	2.97295	0.45302	C	2.12969	2.69834	1.40159
H	0.80865	2.80784	-0.22761	H	0.68066	2.81507	-0.38824	H	1.46669	2.90876	0.56534

H	1.09719	2.48333	1.45735	H	0.90097	2.53610	1.34161	H	1.57604	2.12590	2.14146
H	1.66486	4.02264	0.76061	H	1.44995	4.04607	0.61525	H	2.42293	3.64526	1.85284
C	3.40643	3.05924	-1.01502	C	3.31369	3.10125	-1.04526	C	4.12485	2.87814	-0.04108
H	4.38579	2.69320	-1.30302	H	4.30631	2.73183	-1.27484	H	5.04903	2.42750	-0.38412
H	2.71886	2.90821	-1.84568	H	2.67023	2.93097	-1.90683	H	3.48972	3.07157	-0.90370
H	3.47651	4.13068	-0.81832	H	3.36463	4.17527	-0.85838	H	4.35325	3.83017	0.43921
C	-4.34148	0.55109	0.81978	C	-4.24523	0.57674	0.86064	C	-4.73114	0.05020	0.63105
C	-5.70449	-0.01639	0.39847	C	-5.62434	0.02366	0.47335	C	-5.87198	0.01033	-0.39691
H	-6.47016	0.30919	1.10295	H	-6.36924	0.36746	1.19074	H	-6.81561	-0.20511	0.10603
H	-5.98056	0.31700	-0.59506	H	-5.91810	0.35086	-0.51700	H	-5.96045	0.95573	-0.91971
H	-5.67229	-1.10423	0.40418	H	-5.60778	-1.06423	0.49014	H	-5.68295	-0.77458	-1.12672
C	-4.40447	2.08541	0.86021	C	-4.28555	2.11205	0.88130	C	-5.02428	1.12344	1.68979
H	-4.64531	2.49465	-0.11395	H	-4.53499	2.51337	-0.09385	H	-5.09363	2.10763	1.24167
H	-5.16675	2.40217	1.57240	H	-5.03278	2.44805	1.59996	H	-5.96510	0.89711	2.19304
H	-3.44471	2.48639	1.17803	H	-3.31704	2.50552	1.18223	H	-4.22945	1.13458	2.43293
C	-4.06631	0.07035	2.24621	C	-3.94982	0.10951	2.28752	C	-4.71674	-1.30184	1.34670
H	-4.03829	-1.01571	2.30378	H	-3.93847	-0.97584	2.35960	H	-4.53801	-2.11870	0.65176
H	-3.12520	0.46166	2.62526	H	-2.99641	0.49074	2.64603	H	-3.95453	-1.33527	2.12175
H	-4.86771	0.42345	2.89308	H	-4.73257	0.48347	2.94474	H	-5.68723	-1.45847	1.81604
**SW (C4$^-$)**				**SW (C5$^-$)**				**SW (C6$^-$)**			
C	2.40563	1.00572	-0.34529	C	2.48676	1.00979	-0.07872	C	2.66411	0.87280	-0.10501
C	1.33730	0.16567	-0.04997	C	1.37743	0.17062	-0.03423	C	1.46767	0.15677	-0.07018
C	1.43031	-1.22075	0.02403	C	1.43249	-1.20972	-0.19032	C	1.36920	-1.22621	-0.22658
C	2.66002	-1.81838	-0.24548	C	2.66815	-1.79855	-0.45299	C	2.54868	-1.93267	-0.49709
C	3.74046	-1.00244	-0.56149	C	3.79153	-0.98368	-0.50871	C	3.74513	-1.23840	-0.56791
C	3.62570	0.37733	-0.60374	C	3.71306	0.38656	-0.31577	C	3.81586	0.13261	-0.36252
H	0.36297	0.57905	0.15155	H	0.39880	0.59597	0.10552	H	0.54868	0.68924	0.10223
H	4.70249	-1.45256	-0.77460	H	4.75801	-1.42599	-0.71667	H	4.65997	-1.78024	-0.77836
C	0.20795	-2.02072	0.44192	C	0.16125	-2.03332	-0.04298	C	0.05051	-1.94701	0.03048
H	0.32871	-3.02252	0.03329	H	0.27321	-2.91215	-0.67598	H	0.06788	-2.87053	-0.54893
C	-1.07212	-1.42934	-0.15658	C	-1.05760	-1.27810	-0.55205	C	-1.11111	-1.12698	-0.53984
C	-1.37287	-1.77617	-1.47787	C	-1.20414	-1.04334	-1.93522	C	-1.19647	-0.81301	-1.89722
C	-1.84567	-0.53276	0.59702	C	-2.00626	-0.75615	0.31639	C	-2.12338	-0.68964	0.30250
C	-2.51399	-1.22923	-2.04979	C	-2.24777	-0.27720	-2.45249	C	-2.28999	-0.07914	-2.34218
C	-2.98135	0.01721	-0.02223	C	-3.07838	0.02196	-0.13190	C	-3.21837	0.06704	-0.11296
C	-3.30879	-0.34964	-1.33018	H	-1.91532	-0.95283	1.36913	H	-2.02041	-1.02270	1.32254
H	-2.78665	-1.48349	-3.06808	C	-3.13226	0.23699	-1.51083	C	-3.27654	0.36138	-1.47446
C	0.12761	-2.20877	1.97691	C	0.03239	-2.56797	1.40311	H	-2.38266	0.15108	-3.39721
H	-0.90074	-2.49336	2.18210	H	-0.96130	-3.00024	1.49602	C	-0.09160	-2.36082	1.48658
H	0.77081	-3.04620	2.26295	H	0.74069	-3.38726	1.53404	H	0.37010	-3.32851	1.67386
C	0.48650	-1.00981	2.86212	C	0.27779	-1.55347	2.53426	C	0.27527	-1.34198	2.54717
H	-0.06726	-1.10668	3.79618	H	-0.41787	-1.75231	3.34888	H	-0.35974	-1.46157	3.43377
H	0.11099	-0.10831	2.38899	H	0.06123	-0.54869	2.18323	H	0.05855	-0.33303	2.18404
C	1.97537	-0.89804	3.19071	C	1.70431	-1.59340	3.07850	C	1.73459	-1.35987	3.04828
H	2.16228	-0.06524	3.86737	H	1.83631	-0.87697	3.88705	H	1.90232	-0.64367	3.85950
H	2.33050	-1.80750	3.67730	H	1.93809	-2.58463	3.46553	H	1.97154	-2.35460	3.42493
H	2.57337	-0.74224	2.29676	H	2.42215	-1.35733	2.29705	H	2.42764	-1.13564	2.24058
C	2.84683	-3.31102	-0.18547	C	2.80686	-3.27507	-0.70860	C	2.53997	-3.42555	-0.66115
H	2.57829	-3.69776	0.79478	H	2.44188	-3.86284	0.12984	H	2.11260	-3.88721	0.22743
H	2.21669	-3.81351	-0.91722	H	2.23120	-3.56400	-1.58641	H	1.92943	-3.72918	-1.51026
H	3.88150	-3.57531	-0.38684	H	3.84710	-3.53624	-0.88104	H	3.54802	-3.80332	-0.81217
C	-0.51004	-2.69944	-2.30520	C	-0.20954	-1.65661	-2.90118	C	-0.15297	-1.25418	-2.88788
H	0.52359	-2.35592	-2.32784	H	0.81454	-1.33566	-2.70471	H	0.76324	-0.67891	-2.77334
H	-0.50831	-3.71359	-1.90491	H	-0.22209	-2.74899	-2.85630	H	0.10953	-2.29848	-2.73474
H	-0.87474	-2.74629	-3.32892	H	-0.48817	-1.35213	-3.90352	H	-0.51862	-1.13427	-3.90498
O	-4.44596	0.16305	-1.96232	O	-4.16303	1.02919	-2.02108	O	-4.33918	1.09776	-1.97887
H	-4.46570	-0.20727	-2.84536	H	-3.95469	0.98766	-2.96543	H	-4.18910	1.21273	-2.91752
O	4.73900	1.13867	-0.91360	O	4.86716	1.14376	-0.37798	O	5.05203	0.76508	-0.43288
H	5.47581	0.54093	-1.04297	H	5.59695	0.55346	-0.56804	H	5.71723	0.08246	-0.52313

C	2.24054	2.52396	-0.37561	C	2.35435	2.52052	0.10305	C	2.70729	2.37980	0.14562
C	3.15471	3.17294	0.67397	C	3.17344	2.98020	1.31808	C	3.57283	2.67665	1.37949
H	4.19927	2.95830	0.47806	H	4.22779	2.76236	1.19293	H	4.59242	2.33862	1.23559
H	3.01019	4.25312	0.66334	H	3.05098	4.05457	1.45227	H	3.58097	3.75007	1.57089
H	2.90089	2.80245	1.66506	H	2.81698	2.48036	2.21676	H	3.15821	2.17278	2.25012
C	0.80212	2.93575	-0.05432	C	0.90022	2.93220	0.34352	C	1.31394	2.94894	0.42057
H	0.09556	2.52869	-0.77348	H	0.25793	2.65977	-0.49037	H	0.64467	2.79899	-0.42328
H	0.49991	2.60459	0.93639	H	0.50001	2.47776	1.24725	H	0.86591	2.49408	1.30063
H	0.73664	4.02209	-0.08672	H	0.86213	4.01330	0.46351	H	1.40403	4.01950	0.59839
C	2.57735	3.06975	-1.77097	C	2.83645	3.24841	-1.16034	C	3.27192	3.11379	-1.07882
H	3.60107	2.84689	-2.04952	H	3.87811	3.03340	-1.36945	H	4.27870	2.78450	-1.30782
H	1.90944	2.63272	-2.51017	H	2.23607	2.94448	-2.01471	H	2.63927	2.93048	-1.94517
H	2.43875	4.15073	-1.78107	H	2.72128	4.32349	-1.02492	H	3.28881	4.18683	-0.88649
C	-3.86065	1.03241	0.73142	C	-4.10691	0.60379	0.83899	C	-4.28968	0.53581	0.87045
C	-5.28625	0.48791	0.90803	C	-5.51319	0.09582	0.48970	C	-5.65868	-0.04442	0.48667
H	-5.89347	1.20375	1.46534	H	-6.24548	0.52619	1.17528	H	-6.40919	0.28402	1.20613
H	-5.76092	0.29761	-0.04847	H	-5.77498	0.36482	-0.52701	H	-5.96130	0.27589	-0.50379
H	-5.25086	-0.44357	1.47060	H	-5.54827	-0.98837	0.58254	H	-5.61765	-1.13148	0.50490
C	-3.90532	2.37435	-0.01513	C	-4.08324	2.13773	0.77200	C	-4.36170	2.06979	0.88723
H	-4.33323	2.26665	-1.00571	H	-4.29919	2.47974	-0.23331	H	-4.61770	2.46471	-0.08941
H	-4.50123	3.09452	0.54867	H	-4.82263	2.55272	1.45943	H	-5.11575	2.39310	1.60532
H	-2.89494	2.76925	-0.11274	H	-3.09907	2.50464	1.05974	H	-3.40100	2.48177	1.18944
C	-3.30069	1.30842	2.12566	C	-3.82629	0.20656	2.29033	C	-3.97720	0.07302	2.29480
H	-3.23400	0.39228	2.70368	H	-3.85476	-0.87362	2.41950	H	-3.93480	-1.01111	2.36220
H	-2.29343	1.71011	2.06325	H	-2.85570	0.56930	2.62367	H	-3.02666	0.46936	2.64339
H	-3.95255	2.02447	2.63171	H	-4.59021	0.64529	2.93160	H	-4.76350	0.43040	2.95844
**SW (C7^{-})**				**SW (C8^{-})**				**SW (C9^{-})**			
C	2.67989	0.77853	-0.34001	C	2.44699	1.09839	0.08299	C	2.71569	0.84558	0.16267
C	1.48407	0.10878	-0.08772	C	1.35962	0.24788	-0.10648	C	1.52173	0.12626	0.02235
C	1.39573	-1.26475	0.12625	C	1.46196	-1.10268	-0.41873	C	1.41658	-1.16364	-0.45428
C	2.55405	-2.03146	0.02842	C	2.73809	-1.65899	-0.54463	C	2.58814	-1.89355	-0.87482
C	3.74937	-1.39426	-0.27882	C	3.83607	-0.82162	-0.39420	C	3.80643	-1.15355	-0.71975
C	3.81953	-0.01841	-0.43721	C	3.70438	0.52039	-0.08487	C	3.85821	0.12693	-0.23159
H	0.56909	0.66619	-0.02404	H	0.37064	0.65297	0.00436	H	0.59920	0.61701	0.29101
H	4.65834	-1.97712	-0.36285	H	4.83037	-1.23965	-0.49000	H	4.73488	-1.63477	-1.00531
C	0.08715	-1.84947	0.58632	C	0.21486	-1.96148	-0.60874	C	0.07170	-1.85147	-0.53862
H	0.09427	-2.92185	0.37589	H	0.38180	-2.54462	-1.51559	H	0.11332	-2.49706	-1.41941
C	-1.10169	-1.27372	-0.16285	C	-0.99903	-1.09558	-0.88865	C	-1.06710	-0.88619	-0.80604
C	-1.24875	-1.48324	-1.53696	C	-1.18247	-0.51677	-2.14596	C	-1.06590	-0.13015	-1.98568
C	-2.08035	-0.53707	0.49976	C	-1.92265	-0.80718	0.10983	C	-2.13548	-0.72918	0.06851
C	-2.34909	-0.93450	-2.18065	C	-2.27616	0.31409	-2.34271	C	-2.12762	0.73037	-2.21830
C	-3.18904	0.03080	-0.12842	C	-3.01940	0.03741	-0.05677	C	-3.20644	0.14317	-0.13732
H	-1.94820	-0.38850	1.55853	H	-1.75081	-1.25453	1.07188	H	-2.13041	-1.31668	0.96746
C	-3.29517	-0.18388	-1.50134	C	-3.17552	0.59347	-1.32601	C	-3.17317	0.87682	-1.31948
H	-2.47511	-1.09395	-3.24477	H	-2.43011	0.76737	-3.31430	H	-2.14200	1.31212	-3.13218
C	-0.05288	-1.67781	2.14863	C	-0.00618	-2.98551	0.53348	C	-0.15941	-2.79143	0.66151
H	-1.00608	-2.16860	2.38218	H	-1.04980	-3.30189	0.47253	H	-1.14958	-3.24101	0.57790
H	0.71217	-2.32295	2.59157	H	0.59597	-3.86884	0.34422	H	0.56237	-3.60031	0.56747
C	0.09814	-0.30217	2.75708	C	0.36483	-2.51927	1.96251	C	0.03084	-2.13246	2.03923
H	-0.21653	0.50435	2.09738	H	-0.40064	-2.90985	2.63716	H	-0.73078	-2.49530	2.73159
C	1.49716	-0.04256	3.28846	H	0.24508	-1.41858	1.98246	H	-0.10647	-1.05632	1.95231
H	1.59573	0.99244	3.62177	C	1.72268	-3.01135	2.45051	C	1.41584	-2.40014	2.62316
H	1.71663	-0.66310	4.16293	H	1.75031	-3.02709	3.54156	H	1.54197	-1.90651	3.58540
H	2.32998	-0.22450	2.57626	H	2.53330	-2.36183	2.10836	H	1.56863	-3.46903	2.76444
C	2.54554	-3.51988	0.25653	C	2.95267	-3.12799	-0.73674	H	2.18195	-2.03777	1.94288
H	2.13337	-3.75986	1.23343	H	2.74779	-3.62760	0.21996	C	2.58119	-3.17912	-1.38567
H	1.93827	-4.02986	-0.48989	H	2.28331	-3.54492	-1.48608	H	1.69057	-3.77766	-1.45268
H	3.55517	-3.91762	0.20255	H	3.97844	-3.33137	-1.03400	H	3.50861	-3.63813	-1.68922
C	-0.25675	-2.28984	-2.32988	C	-0.21861	-0.76055	-3.27507	C	0.05256	-0.20649	-2.98841

H	0.71873	-1.80939	-2.33669	H	0.79776	-0.52257	-2.97012	H	0.87987	0.42749	-2.67611
H	-0.12245	-3.27711	-1.89202	H	-0.23056	-1.80567	-3.57955	H	0.45701	-1.21220	-3.06989
H	-0.59723	-2.41266	-3.35478	H	-0.47826	-0.15261	-4.13720	H	-0.29654	0.11879	-3.96589
O	-4.35839	0.35457	-2.20579	O	-4.24129	1.43620	-1.58087	O	-4.19579	1.76279	-1.61129
H	-4.24077	0.12677	-3.12837	H	-4.16891	1.73557	-2.48772	H	-3.97729	2.19716	-2.43641
O	5.04749	0.56505	-0.70539	O	4.84192	1.29420	0.06394	O	5.10053	0.74869	-0.12417
H	5.70966	-0.12536	-0.66681	H	5.59822	0.70928	0.00577	H	5.74995	0.10854	-0.41722
C	2.73588	2.30047	-0.45656	C	2.26440	2.56623	0.46452	C	2.77258	2.27315	0.69299
C	3.62577	2.86738	0.66002	C	2.93318	2.84564	1.81888	C	3.60574	2.34675	1.98343
H	4.64339	2.50351	0.57581	H	3.99980	2.65944	1.77669	H	4.62035	2.00783	1.80663
H	3.63604	3.95615	0.60135	H	2.76888	3.88609	2.09992	H	3.63590	3.37218	2.35756
H	3.22891	2.57341	1.62909	H	2.49990	2.20646	2.58474	H	3.15702	1.71198	2.74519
C	1.34890	2.92534	-0.29287	C	0.78534	2.93502	0.60246	C	1.37601	2.80150	1.02721
H	0.66403	2.58762	-1.06759	H	0.24383	2.78708	-0.32944	H	0.73282	2.80662	0.14938
H	0.92074	2.68513	0.67744	H	0.30035	2.34920	1.37993	H	0.89918	2.20083	1.79897
H	1.44351	4.00734	-0.37053	H	0.71271	3.98678	0.87408	H	1.46181	3.82361	1.39470
C	3.27933	2.72229	-1.82908	C	2.86824	3.47877	-0.61293	C	3.37681	3.22581	-0.35235
H	4.27980	2.33923	-1.99414	H	3.92794	3.29024	-0.74057	H	4.37742	2.91281	-0.62822
H	2.62825	2.34958	-2.61761	H	2.36725	3.31204	-1.56454	H	2.75605	3.22916	-1.24657
H	3.30525	3.81043	-1.89177	H	2.72930	4.52203	-0.32883	H	3.42089	4.24240	0.04368
C	-4.20863	0.86052	0.65103	C	-3.97749	0.34632	1.09231	C	-4.33826	0.28547	0.87916
C	-5.61337	0.25402	0.51666	C	-5.40144	-0.10466	0.73578	C	-5.68093	-0.09676	0.23969
H	-6.31776	0.84227	1.10448	H	-6.06991	0.11100	1.56889	H	-6.47752	-0.00354	0.97783
H	-5.94782	0.23930	-0.51430	H	-5.77034	0.40362	-0.14742	H	-5.91200	0.54176	-0.60492
H	-5.61566	-0.76413	0.90029	H	-5.41612	-1.17721	0.55390	H	-5.64899	-1.12915	-0.10276
C	-4.21390	2.30449	0.12750	C	-3.96489	1.85245	1.39436	C	-4.40134	1.72865	1.40100
H	-4.47786	2.34508	-0.92371	H	-4.28346	2.43192	0.53550	H	-4.58932	2.43022	0.59682
H	-4.93587	2.89347	0.69301	H	-4.63556	2.06211	2.22718	H	-5.20039	1.81650	2.13704
H	-3.23009	2.74939	0.26027	H	-2.96150	2.16609	1.67510	H	-3.46108	1.99204	1.88038
C	-3.86995	0.90924	2.14215	C	-3.57201	-0.38182	2.37538	C	-4.13181	-0.62796	2.08921
H	-3.88078	-0.08247	2.58771	H	-3.58983	-1.46149	2.24732	H	-4.11375	-1.67660	1.80070
H	-2.88956	1.33869	2.33120	H	-2.57710	-0.09396	2.70598	H	-3.20743	-0.39726	2.61350
H	-4.61801	1.51866	2.64742	H	-4.27980	-0.12188	3.16028	H	-4.95963	-0.48190	2.78079
**SW ($C10^{-}$)**				**SW ($C11^{-}$)**				**SW ($C12^{-}$)**			
C	2.71569	0.84558	0.16267	C	2.34086	1.07766	-0.32980	C	2.56302	0.93978	-0.12577
C	1.52173	0.12626	0.02235	C	1.31617	0.17638	-0.08493	C	1.41404	0.16024	-0.03639
C	1.41658	-1.16364	-0.45428	C	1.46319	-1.20536	-0.01719	C	1.39955	-1.22652	-0.14354
C	2.58814	-1.89355	-0.87482	C	2.71882	-1.75694	-0.25886	C	2.60056	-1.88716	-0.39608
C	3.80643	-1.15355	-0.71975	C	3.76501	-0.88594	-0.53960	C	3.76015	-1.13112	-0.50623
C	3.85821	0.12693	-0.23159	C	3.59015	0.49383	-0.56765	C	3.75198	0.24753	-0.36595
H	0.59920	0.61701	0.29101	H	0.35047	0.63220	0.07238	H	0.46590	0.64362	0.12202
H	4.73488	-1.63477	-1.00531	H	4.75046	-1.29316	-0.73495	H	4.70079	-1.62870	-0.70605
C	0.07170	-1.85147	-0.53862	C	0.27528	-2.06161	0.39842	C	0.09876	-1.98532	0.06439
H	0.11332	-2.49706	-1.41941	H	0.42105	-3.06128	-0.00794	H	0.17042	-2.91171	-0.50529
C	-1.06710	-0.88619	-0.80604	C	-0.99996	-1.52115	-0.22052	C	-1.09414	-1.22437	-0.49027
C	-1.06590	-0.13015	-1.98568	C	-1.23557	-1.70715	-1.58345	C	-1.21521	-1.05383	-1.87134
C	-2.13548	-0.72918	0.06851	C	-1.89565	-0.74538	0.50356	C	-2.06651	-0.66768	0.33368
C	-2.12762	0.73037	-2.21830	C	-2.35156	-1.11020	-2.14796	C	-2.29223	-0.31983	-2.34502
C	-3.20644	0.14317	-0.13732	C	-2.99337	-0.09029	-0.04977	C	-3.15367	0.07937	-0.10667
H	-2.13041	-1.31668	0.96746	H	-1.69874	-0.60098	1.54840	H	-2.03132	-0.78631	1.40520
C	-3.17317	0.87682	-1.31948	C	-3.20591	-0.30526	-1.41043	C	-3.23515	0.24299	-1.49145
H	-2.14200	1.31212	-3.13218	H	-2.55000	-1.24896	-3.20305	H	-2.40474	-0.18190	-3.41440
C	-0.15941	-2.79143	0.66151	C	0.20819	-2.23315	1.93497	C	-0.06094	-2.40730	1.54285
H	-1.14958	-3.24101	0.57790	H	-0.77165	-2.64257	2.17766	H	-1.06417	-2.81079	1.65520
H	0.56237	-3.60031	0.56747	H	0.93614	-2.99464	2.21575	H	0.63089	-3.22763	1.73803
C	0.03084	-2.13246	2.03923	C	0.48947	-0.98287	2.78873	C	0.18668	-1.31610	2.59784
H	-0.73078	-2.49530	2.73159	H	-0.13369	-1.02237	3.68223	H	-0.52066	-1.44878	3.41322
H	-0.10647	-1.05632	1.95231	H	0.21421	-0.07856	2.25304	H	-0.02914	-0.33654	2.18225
C	1.41584	-2.40014	2.62316	C	1.95249	-0.86343	3.20901	C	1.60913	-1.33119	3.15362

H	1.54197	-1.90651	3.58540	H	2.10707	0.01957	3.82431	H	1.74076	-0.55609	3.90563
H	1.56863	-3.46903	2.76444	H	2.25823	-1.73809	3.78309	H	1.82605	-2.29132	3.62062
H	2.18195	-2.03777	1.94288	H	2.59723	-0.78062	2.33844	H	2.34129	-1.16631	2.36631
C	2.58119	-3.17912	-1.38567	C	2.96777	-3.24299	-0.22093	C	2.66872	-3.38071	-0.57036
H	1.69057	-3.77766	-1.45268	H	2.72564	-3.66570	0.75235	H	2.27780	-3.90165	0.29949
H	3.50861	-3.63813	-1.68922	H	2.36397	-3.76488	-0.96277	H	2.08175	-3.69624	-1.43138
C	0.05256	-0.20649	-2.98841	H	4.01228	-3.45731	-0.42884	H	3.69528	-3.69946	-0.72515
H	0.87987	0.42749	-2.67611	C	-0.29751	-2.50704	-2.44501	C	-0.23055	-1.64859	-2.84396
H	0.45701	-1.21220	-3.06989	H	0.70968	-2.09857	-2.39243	H	0.77150	-1.25228	-2.69130
H	-0.29654	0.11879	-3.96589	H	-0.25012	-3.54513	-2.11859	H	-0.17166	-2.73117	-2.73230
O	-4.19579	1.76279	-1.61129	H	-0.62551	-2.49272	-3.48016	H	-0.53199	-1.43532	-3.86570
H	-3.97729	2.19716	-2.43641	O	-4.27417	0.29693	-2.04138	O	-4.27031	0.96761	-2.04805
O	5.10053	0.74869	-0.12417	H	-4.15422	0.20010	-2.98662	H	-4.14985	0.97052	-2.99794
H	5.74995	0.10854	-0.41722	O	4.68421	1.28801	-0.84381	O	4.93751	0.94393	-0.47740
C	2.77258	2.27315	0.69299	H	5.44144	0.71553	-0.96832	H	5.64006	0.31376	-0.64084
C	3.60574	2.34675	1.98343	C	2.02056	2.61305	-0.30215	C	2.51630	2.45772	0.04075
H	4.62035	2.00783	1.80663	C	2.93324	3.23782	0.76849	C	3.37804	2.87781	1.24102
H	3.63590	3.37218	2.35756	H	3.98719	3.01724	0.61053	H	4.41660	2.60018	1.10368
H	3.15702	1.71198	2.74519	H	2.77053	4.31224	0.75360	H	3.31858	3.95795	1.36816
C	1.37601	2.80150	1.02721	H	2.63735	2.86861	1.75046	H	3.00539	2.40628	2.14796
H	0.73282	2.80662	0.14938	C	0.59306	2.99984	0.00477	C	1.09204	2.95172	0.30299
H	0.89918	2.20083	1.79897	H	-0.10980	2.61651	-0.74086	H	0.42209	2.71404	-0.51979
H	1.46181	3.82361	1.39470	H	0.27993	2.68099	1.00560	H	0.67757	2.52880	1.21492
C	3.37681	3.22581	-0.35235	C	2.39280	3.18007	-1.68373	H	1.11568	4.03356	0.41630
H	4.37742	2.91281	-0.62822	H	3.41231	2.94006	-1.98092	C	3.01858	3.15020	-1.23435
H	2.75605	3.22916	-1.24657	H	1.70370	2.78464	-2.42939	H	4.04363	2.87934	-1.45913
H	3.42089	4.24240	0.04368	H	2.25985	4.25807	-1.64651	H	2.38960	2.87610	-2.07865
C	-4.33826	0.28547	0.87916	C	-3.84132	0.88164	0.76782	H	2.96354	4.23028	-1.10372
C	-5.68093	-0.09676	0.23969	C	-5.31768	0.46092	0.75822	C	-4.15721	0.66000	0.95129
H	-6.47752	-0.00354	0.97783	H	-5.89762	1.16925	1.34891	C	-5.54946	0.10046	0.60621
H	-5.91200	0.54176	-0.60492	H	-5.71866	0.43915	-0.24814	H	-6.26742	0.56483	1.27706
H	-5.64899	-1.12915	-0.10276	H	-5.43124	-0.52753	1.20074	H	-5.83661	0.27847	-0.42836
C	-4.40134	1.72865	1.40100	C	-3.68286	2.29728	0.18960	H	-5.56044	-0.97221	0.79756
H	-4.58932	2.43022	0.59682	H	-4.04191	2.34700	-0.83198	C	-4.16247	2.18892	0.76897
H	-5.20039	1.81650	2.13704	H	-4.25802	2.99868	0.79358	H	-4.37212	2.49662	-0.25366
H	-3.46108	1.99204	1.88038	H	-2.63773	2.60065	0.20893	H	-4.91050	2.59878	1.44250
C	-4.13181	-0.62796	2.08921	C	-3.38622	0.93880	2.22790	H	-3.19236	2.58691	1.06689
H	-4.11375	-1.67660	1.80070	H	-3.46188	-0.03293	2.71330	C	-3.86918	0.35653	2.40068
H	-3.20743	-0.39726	2.61350	H	-2.36415	1.30094	2.31053	H	-3.89978	-0.71541	2.61794
H	-4.95963	-0.48190	2.78079	H	-4.03073	1.63391	2.76204	H	-2.91497	0.77605	2.73488

SPLET (ETE step) mechanism

M05-2X/6-311++G(2d,2p)											
SW (O1•)				SW (O2•)				SW (C1•)			
C	2.54437	0.94979	-0.12507	C	2.64341	0.93568	-0.07404	C	2.48946	1.03498	-0.07281
C	1.39836	0.16942	-0.01894	C	1.49043	0.19436	-0.02726	C	1.46761	0.12643	-0.01507
C	1.38782	-1.21815	-0.11227	C	1.44963	-1.20010	-0.20176	C	1.47802	-1.23471	-0.17014
C	2.58600	-1.88545	-0.36126	C	2.64357	-1.91188	-0.49090	C	2.73449	-1.78513	-0.45087
C	3.74234	-1.12845	-0.48081	C	3.80950	-1.20978	-0.55856	C	3.83343	-0.93536	-0.53139
C	3.73380	0.25339	-0.35939	C	3.88807	0.21907	-0.34992	C	3.72898	0.43859	-0.34383
H	0.45616	0.66295	0.14364	H	0.55064	0.68732	0.15034	H	4.80538	-1.35490	-0.75170
H	4.68241	-1.62700	-0.67550	H	4.74645	-1.69752	-0.77697	C	0.21142	-2.05174	-0.00725
C	0.09258	-1.98467	0.11023	C	0.13870	-1.93254	-0.02760	H	0.31116	-2.94618	-0.62103
H	0.16664	-2.91363	-0.45454	H	0.18783	-2.83534	-0.63462	C	-0.98173	-1.27513	-0.53997
C	-1.08513	-1.24132	-0.47750	C	-1.04297	-1.12938	-0.54495	C	-1.08653	-1.02992	-1.91197
C	-1.17721	-1.10576	-1.89262	C	-1.12791	-0.83493	-1.90877	C	-1.96888	-0.77169	0.29820
C	-2.07403	-0.67086	0.33815	C	-2.05566	-0.67834	0.29372	C	-2.15883	-0.28055	-2.36763
C	-2.21297	-0.38738	-2.40571	C	-2.21189	-0.09733	-2.35635	C	-3.04987	-0.00627	-0.13404
C	-3.13109	0.06762	-0.14046	C	-3.15106	0.07017	-0.13108	H	-1.89104	-0.97842	1.34812
H	-1.98856	-0.81708	1.39822	H	-1.99164	-0.91978	1.33743	C	-3.11810	0.23191	-1.50678
C	-3.22392	0.24305	-1.58753	C	-3.20212	0.35450	-1.49654	H	-2.25481	-0.08603	-3.42764
H	-2.33027	-0.25478	-3.46985	H	-2.29476	0.13200	-3.41028	C	0.05823	-2.53546	1.44964
C	-0.07286	-2.39284	1.59014	C	-0.01143	-2.42179	1.43334	H	-0.92596	-2.98927	1.54668
H	-1.06723	-2.81854	1.70901	H	-0.99902	-2.86875	1.52115	H	0.78013	-3.33444	1.61316
H	0.62689	-3.20328	1.78633	H	0.70272	-3.22800	1.58985	C	0.27422	-1.46570	2.53606
C	0.17389	-1.29279	2.63811	C	0.21145	-1.36982	2.53441	H	-0.45719	-1.60818	3.32998
H	-0.52497	-1.42894	3.46167	H	-0.50159	-1.54428	3.33826	H	0.09711	-0.47546	2.12199
H	-0.03452	-0.31279	2.21626	H	0.00498	-0.37293	2.15361	C	1.67682	-1.50531	3.13714
C	1.59797	-1.30113	3.18894	C	1.62490	-1.40442	3.11039	H	1.79618	-0.74256	3.90264
H	1.73141	-0.52115	3.93428	H	1.75192	-0.64724	3.87980	H	1.86844	-2.47397	3.59490
H	1.81681	-2.25705	3.66056	H	1.82890	-2.37528	3.55712	H	2.43086	-1.33679	2.37207
H	2.32352	-1.13953	2.39602	H	2.36897	-1.22694	2.33818	C	2.90653	-3.26211	-0.68500
C	2.65508	-3.37997	-0.52325	C	2.63578	-3.39535	-0.74387	H	2.55559	-3.84199	0.16480
H	2.26835	-3.89791	0.35029	H	2.20824	-3.94591	0.08950	H	2.34202	-3.58341	-1.55868
H	2.07265	-3.70453	-1.38403	H	2.05348	-3.63589	-1.63139	H	3.95139	-3.50270	-0.85176
H	3.68097	-3.69848	-0.67530	H	3.64868	-3.74979	-0.90022	C	-0.08197	-1.56113	-2.89741
C	-0.17501	-1.75099	-2.80982	C	-0.09136	-1.29718	-2.89658	H	0.91245	-1.17260	-2.69148
H	0.82941	-1.38266	-2.61879	H	0.86783	-0.81603	-2.71908	H	-0.02563	-2.64745	-2.85100
H	-0.16297	-2.83107	-2.67482	H	0.06597	-2.37179	-2.82985	H	-0.35894	-1.28388	-3.90939
H	-0.43197	-1.54454	-3.84305	H	-0.40473	-1.06767	-3.90975	O	-4.15248	0.97896	-2.01416
O	-4.13136	0.89743	-2.13037	O	-4.25001	1.08426	-1.99512	H	-4.04274	1.05440	-2.96314
O	4.90723	0.95027	-0.48069	H	-4.13069	1.19696	-2.93941	O	4.84049	1.23501	-0.42775
H	5.62269	0.33403	-0.64514	O	4.99218	0.78602	-0.41900	H	5.60841	0.68525	-0.59208
C	2.49948	2.47013	0.01487	C	2.66122	2.43476	0.15609	C	2.26228	2.52458	0.14126
C	3.36854	2.91236	1.20182	C	3.54178	2.75569	1.37522	C	3.06763	3.01450	1.35032
H	4.40663	2.63684	1.06185	H	4.56399	2.43834	1.21087	H	4.13268	2.88435	1.19352
H	3.30809	3.99390	1.30933	H	3.52738	3.82987	1.55045	H	2.86536	4.07151	1.51471
H	3.00608	2.45690	2.12130	H	3.15331	2.26026	2.26318	H	2.77563	2.46669	2.24389
C	1.07722	2.96985	0.27791	C	1.26016	2.98283	0.43013	C	0.77687	2.77104	0.41162
H	0.40298	2.72159	-0.53892	H	0.58741	2.81007	-0.40735	H	0.16335	2.42444	-0.41759
H	0.67266	2.56456	1.20336	H	0.82487	2.54341	1.32551	H	0.45398	2.25023	1.31100
H	1.10313	4.05275	0.37201	H	1.33218	4.05628	0.58685	H	0.60620	3.83663	0.55019
C	2.99091	3.13865	-1.27713	C	3.21606	3.14870	-1.08707	C	2.66866	3.30714	-1.11309
H	4.01479	2.86691	-1.50332	H	4.23033	2.83230	-1.29674	H	3.72076	3.17144	-1.33830
H	2.35824	2.84930	-2.11341	H	2.59132	2.94102	-1.95369	H	2.08178	2.97908	-1.96818
H	2.93612	4.22021	-1.16603	H	3.20861	4.22295	-0.91186	H	2.47951	4.36734	-0.95379
C	-4.16775	0.69659	0.77046	C	-4.22554	0.55015	0.84307	C	-4.08401	0.55044	0.84319
C	-5.56450	0.15483	0.42426	C	-5.59311	-0.03002	0.45383	C	-5.48322	0.01590	0.50454

H	-6.29383	0.59780	1.10015	H	-6.34344	0.30689	1.16705	H	-6.20234	0.40770	1.22220
H	-5.83349	0.39952	-0.59584	H	-5.89320	0.28613	-0.53771	H	-5.79336	0.31068	-0.49053
H	-5.59502	-0.92553	0.55146	H	-5.56133	-1.11722	0.47910	H	-5.49561	-1.07027	0.56669
C	-4.14703	2.22420	0.59456	C	-4.28697	2.08497	0.84453	C	-4.08057	2.08552	0.78957
H	-4.39026	2.50029	-0.42401	H	-4.54037	2.47475	-0.13386	H	-4.34185	2.44952	-0.19672
H	-4.87787	2.66920	1.26750	H	-5.04214	2.41518	1.55572	H	-4.80241	2.47425	1.50618
H	-3.16539	2.62114	0.84698	H	-3.32755	2.49885	1.14809	H	-3.09605	2.46684	1.05332
C	-3.89543	0.38814	2.24329	C	-3.93064	0.10327	2.27653	C	-3.77640	0.14349	2.28586
H	-3.92712	-0.68114	2.44247	H	-3.90888	-0.98099	2.36433	H	-3.79429	-0.93697	2.41199
H	-2.93236	0.77805	2.56713	H	-2.98647	0.50466	2.63918	H	-2.80939	0.51930	2.61373
H	-4.66646	0.86238	2.84524	H	-4.72135	0.47410	2.92409	H	-4.53803	0.56799	2.93565
**SW (C2·)**				**SW (C3·)**				**SW (C4·)**			
C	2.64404	0.91277	-0.09438	C	-3.00355	-0.67047	0.27782	C	2.63107	0.89287	-0.08649
C	1.47880	0.15027	-0.02656	C	-1.70739	-0.19269	0.16125	C	1.45990	0.14367	-0.05164
C	1.42960	-1.23123	-0.18434	C	-1.35099	0.98534	-0.51117	C	1.41576	-1.24156	-0.16708
C	2.61591	-1.92727	-0.46460	C	-2.36531	1.72058	-1.13656	C	2.60757	-1.93602	-0.36687
C	3.73198	-1.14299	-0.53219	C	-3.66936	1.25952	-1.03031	C	3.78957	-1.21039	-0.41624
C	3.82949	0.21316	-0.36276	C	-3.99512	0.10590	-0.33566	C	3.81198	0.16835	-0.27154
H	0.54593	0.65412	0.15282	H	-0.90505	-0.74321	0.62134	H	0.52288	0.65673	0.07161
C	0.11892	-1.98150	-0.00297	H	-4.46134	1.80661	-1.52558	H	4.72444	-1.73179	-0.57320
H	0.17636	-2.88257	-0.61251	C	0.05520	1.39643	-0.47735	C	0.09574	-1.97815	-0.01627
C	-1.06272	-1.18634	-0.53065	C	1.10606	0.42979	-0.69026	H	0.15750	-2.89024	-0.60894
C	-1.17089	-0.94555	-1.90363	C	0.99911	-0.64219	-1.60755	C	-1.06684	-1.18204	-0.57156
C	-2.04699	-0.67710	0.30809	C	2.32170	0.55885	0.01345	C	-1.19641	-0.92050	-1.94582
C	-2.24179	-0.19380	-2.35862	C	2.06684	-1.51623	-1.71618	C	-2.06555	-0.65645	0.20330
C	-3.13012	0.08611	-0.12452	C	3.39418	-0.31121	-0.08568	C	-2.27678	-0.16810	-2.38702
H	-1.96823	-0.88231	1.35855	H	2.40682	1.37972	0.69944	C	-3.15909	0.09623	-0.14155
C	-3.19969	0.32160	-1.49776	C	3.23180	-1.37943	-0.97570	C	-3.23809	0.33771	-1.51816
H	-2.33966	-0.00245	-3.41901	H	2.00478	-2.32690	-2.43038	H	-2.37924	0.03152	-3.44478
C	-0.03778	-2.46609	1.45494	C	0.39627	2.75410	0.06693	C	-0.15059	-2.41774	1.44283
H	-1.03232	-2.89598	1.55478	H	1.40199	3.02419	-0.24534	H	-1.14881	-2.84911	1.48215
H	0.66356	-3.28431	1.61013	H	-0.27095	3.50202	-0.35434	H	0.54850	-3.21696	1.68473
C	0.21118	-1.41754	2.55408	C	0.28824	2.86661	1.60451	C	-0.01257	-1.31600	2.50417
H	-0.49598	-1.58108	3.36574	H	0.84599	3.74881	1.91637	H	-0.74879	-1.49027	3.28622
H	0.01737	-0.41822	2.17300	H	0.76765	2.00743	2.06981	H	-0.25964	-0.35001	2.06879
C	1.63046	-1.46678	3.11515	C	-1.14866	2.98386	2.10449	C	1.37654	-1.25657	3.13655
H	1.76875	-0.71970	3.89290	H	-1.16994	3.13393	3.18096	H	1.42980	-0.47062	3.88616
H	1.83399	-2.44411	3.54855	H	-1.64465	3.83330	1.63773	H	1.60915	-2.20091	3.62527
H	2.36421	-1.28281	2.33469	H	-1.72596	2.09391	1.87431	H	2.14378	-1.06472	2.39137
C	2.67316	-3.41318	-0.70093	C	-2.09505	2.93875	-1.97810	C	2.64542	-3.42983	-0.54855
H	2.28203	-3.96535	0.14892	H	-2.17071	3.85498	-1.39539	H	2.20287	-3.94916	0.29708
H	2.08526	-3.68526	-1.57551	H	-1.09980	2.89744	-2.41084	H	2.09579	-3.72694	-1.44017
H	3.69731	-3.72574	-0.86965	H	-2.81983	3.00248	-2.78469	H	3.66897	-3.77355	-0.65657
C	-0.17405	-1.48791	-2.89070	C	-0.16678	-0.83176	-2.54178	C	-0.19152	-1.44632	-2.93383
H	0.82545	-1.11175	-2.68755	H	-1.00523	-1.32665	-2.05782	H	0.80044	-1.05309	-2.72523
H	-0.13022	-2.57476	-2.84234	H	-0.53014	0.12441	-2.90638	H	-0.12942	-2.53192	-2.88254
H	-0.45066	-1.20941	-3.90240	H	0.14184	-1.43276	-3.39211	H	-0.47132	-1.17128	-3.94560
O	-4.23440	1.06708	-2.00534	O	4.24543	-2.28836	-1.13365	O	-4.28360	1.07983	-2.00210
H	-4.13018	1.13517	-2.95556	H	3.97288	-2.94992	-1.77139	H	-4.19598	1.16043	-2.95334
O	5.03117	0.86381	-0.46021	O	-5.30441	-0.29375	-0.26069	O	5.01020	0.83514	-0.32200
H	5.70647	0.21195	-0.65869	H	-5.85103	0.34347	-0.72293	H	5.71513	0.19931	-0.45366
C	2.63580	2.42659	0.11750	C	-3.32658	-1.96244	1.02548	C	2.62139	2.41163	0.07785
C	3.51800	2.78740	1.32169	C	-4.25858	-1.66995	2.21041	C	3.43758	2.80902	1.31716
H	4.54698	2.48598	1.16639	H	-5.20068	-1.24791	1.88177	H	4.47318	2.50429	1.22729
H	3.49119	3.86431	1.47862	H	-4.46128	-2.59506	2.74716	H	3.40303	3.89013	1.44080
H	3.14267	2.30105	2.21976	H	-3.78410	-0.97306	2.89810	H	3.01357	2.34906	2.20746
C	1.22615	2.94663	0.40527	C	-2.06388	-2.62123	1.58357	C	1.20176	2.94803	0.27409
H	0.54666	2.75138	-0.42126	H	-1.36130	-2.87981	0.79411	H	0.56491	2.72926	-0.58031
H	0.81274	2.50612	1.30997	H	-1.55971	-1.98132	2.30387	H	0.73872	2.53930	1.16972

H	1.27842	4.02313	0.55078	H	-2.34811	-3.53909	2.09253	H	1.25222	4.02853	0.38717
C	3.14628	3.14013	-1.14230	C	-3.98774	-2.97083	0.07378	C	3.20144	3.08733	-1.17312
H	4.16214	2.84937	-1.38153	H	-4.91758	-2.58896	-0.32995	H	4.22705	2.78647	-1.34956
H	2.50625	2.90723	-1.99064	H	-3.31731	-3.20372	-0.75120	H	2.60610	2.83207	-2.04736
H	3.12251	4.21655	-0.98118	H	-4.19601	-3.89246	0.61434	H	3.17389	4.16809	-1.04452
C	-4.17015	0.63475	0.85107	C	4.67132	-0.11947	0.73057	C	-4.16138	0.60372	0.88636
C	-5.56344	0.08727	0.50807	C	5.87273	0.07136	-0.20663	C	-5.56038	0.05447	0.58236
H	-6.28793	0.47149	1.22435	H	6.77199	0.22919	0.38652	H	-6.25718	0.40291	1.34287
H	-5.87385	0.38038	-0.48740	H	6.02504	-0.79340	-0.84091	H	-5.91247	0.38498	-0.38829
H	-5.56573	-0.99901	0.56896	H	5.72130	0.94502	-0.83698	H	-5.55070	-1.03314	0.59903
C	-4.18196	2.16977	0.79880	C	4.90352	-1.33274	1.64298	C	-4.18807	2.13694	0.88509
H	-4.44159	2.53251	-0.18834	H	5.01812	-2.24582	1.07173	H	-4.49880	2.52481	-0.07863
H	-4.91170	2.55008	1.51179	H	5.80701	-1.17483	2.22964	H	-4.88642	2.48743	1.64325
H	-3.20343	2.56158	1.06907	H	4.06724	-1.45207	2.32847	H	-3.20155	2.53127	1.11950
C	-3.86295	0.22890	2.29412	C	4.59117	1.11709	1.62823	C	-3.74556	0.12708	2.27828
H	-3.87336	-0.85170	2.41945	H	4.45872	2.02819	1.04878	H	-3.71684	-0.95951	2.32591
H	-2.89977	0.61143	2.62543	H	3.78205	1.03902	2.35124	H	-2.76108	0.50583	2.54319
H	-4.62928	0.64726	2.94227	H	5.52434	1.20458	2.17955	H	-4.46375	0.48663	3.01226
**SW (C5•)**				**SW (C6•)**				**SW (C7•)**			
C	2.63100	0.91592	-0.07192	C	2.49530	1.07180	0.04761	C	2.54530	0.89786	-0.30523
C	1.46134	0.16489	-0.02439	C	1.38396	0.23912	-0.04915	C	1.41424	0.13554	-0.03894
C	1.41054	-1.21371	-0.20099	C	1.44931	-1.11346	-0.36101	C	1.41840	-1.24984	0.07993
C	2.59305	-1.89759	-0.47880	C	2.69639	-1.68804	-0.60350	C	2.61595	-1.93726	-0.09899
C	3.77250	-1.16915	-0.54494	C	3.81935	-0.87852	-0.51856	C	3.75862	-1.19980	-0.38274
C	3.80256	0.20169	-0.33969	C	3.73091	0.46877	-0.20027	C	3.73434	0.18278	-0.48002
H	0.52932	0.67174	0.15367	H	0.40772	0.65843	0.11935	H	0.47176	0.63508	0.10043
H	4.69964	-1.68230	-0.76288	H	4.79537	-1.30617	-0.70685	H	4.69799	-1.71547	-0.53196
C	0.09369	-1.95448	-0.03561	C	0.18619	-1.96869	-0.40196	C	0.14388	-1.96204	0.49630
H	0.14390	-2.85003	-0.65406	H	0.31576	-2.69705	-1.20173	H	0.21510	-2.99668	0.16667
C	-1.08215	-1.14802	-0.56132	C	-1.03921	-1.14042	-0.77488	C	-1.07569	-1.37187	-0.18330
C	-1.15202	-0.86729	-1.93922	C	-1.20975	-0.70161	-2.08808	C	-1.27123	-1.58918	-1.54954
C	-2.09608	-0.67693	0.26563	C	-1.97035	-0.76536	0.18425	C	-1.99653	-0.58704	0.49877
C	-2.23338	-0.12860	-2.31725	C	-2.30524	0.09808	-2.37287	C	-2.36777	-1.00205	-2.15997
C	-3.18761	0.08119	-0.16570	C	-3.07260	0.04671	-0.07417	C	-3.09984	0.02471	-0.09352
H	-2.03689	-0.91030	1.31113	H	-1.81811	-1.12443	1.18619	H	-1.83899	-0.43338	1.54834
C	-3.24595	0.36386	-1.53484	C	-3.21726	0.47171	-1.39623	C	-3.26224	-0.20575	-1.45996
C	-0.07986	-2.45218	1.41572	H	-2.45621	0.44653	-3.38608	H	-2.53363	-1.16473	-3.21679
H	-1.07201	-2.89043	1.50098	C	0.02049	-2.74453	0.87077	C	0.05602	-2.02434	2.05320
H	0.62586	-3.26629	1.57275	H	-0.40348	-3.73460	0.80639	H	-0.92261	-2.42482	2.31363
C	0.14913	-1.40859	2.52371	C	0.36153	-2.20461	2.21575	H	0.79785	-2.75982	2.37019
H	-0.56844	-1.57905	3.32480	H	-0.43874	-2.44349	2.91852	C	0.32377	-0.75647	2.79099
H	-0.04469	-0.40830	2.14487	H	0.44102	-1.11989	2.17148	H	-0.49954	-0.16278	3.15177
C	1.56064	-1.45517	3.10419	C	1.68038	-2.77165	2.76744	C	1.69594	-0.41909	3.25411
H	1.68598	-0.71030	3.88626	H	1.86601	-2.40479	3.77392	H	1.78996	0.63573	3.49793
H	1.76090	-2.43339	3.53715	H	1.64481	-3.85797	2.79932	H	1.95647	-0.98557	4.15532
H	2.30421	-1.26651	2.33416	H	2.51039	-2.47315	2.13315	H	2.43955	-0.66297	2.49663
C	2.62457	-3.38175	-0.72868	C	2.84334	-3.14378	-0.95178	C	2.70236	-3.43725	-0.00328
H	2.21589	-3.94023	0.10902	H	2.36704	-3.77316	-0.20300	H	2.35814	-3.79746	0.96305
H	2.04161	-3.64025	-1.61105	H	2.37665	-3.36482	-1.91053	H	2.09056	-3.91318	-0.76761
H	3.64345	-3.71772	-0.89132	H	3.89123	-3.41827	-1.01727	H	3.72673	-3.76845	-0.13918
C	-0.13123	-1.34314	-2.93724	C	-0.24186	-1.06997	-3.17861	C	-0.33326	-2.43972	-2.36084
H	0.83593	-0.88364	-2.75061	H	0.77032	-0.76793	-2.91894	H	0.68463	-2.06110	-2.30447
H	-0.00185	-2.42160	-2.87397	H	-0.23196	-2.14598	-3.34411	H	-0.32368	-3.46558	-1.99602
H	-0.44989	-1.09621	-3.94363	H	-0.51696	-0.59006	-4.11214	H	-0.63879	-2.45784	-3.40208
O	-4.27328	1.09718	-2.06842	O	-4.28024	1.27107	-1.73592	O	-4.32254	0.36270	-2.12132
H	-4.11999	1.17871	-3.01198	H	-4.22549	1.47825	-2.66997	H	-4.27901	0.11425	-3.04582
O	4.99830	0.87066	-0.41043	O	4.87523	1.22327	-0.13118	O	4.89347	0.86337	-0.75791
H	5.69474	0.24253	-0.60741	H	5.62919	0.66025	-0.31261	H	5.61152	0.23390	-0.83916
C	2.63004	2.42546	0.16225	C	2.36302	2.55283	0.39654	C	2.48717	2.42168	-0.38798

C	3.49996	2.76596	1.38170	C	3.13131	2.86318	1.68986	C	3.40477	3.04044	0.67710
H	4.52930	2.46229	1.23455	H	4.18882	2.65320	1.58508	H	4.43935	2.75860	0.52420
H	3.47561	3.84072	1.55350	H	3.00876	3.91605	1.93790	H	3.32918	4.12568	0.63232
H	3.11179	2.26895	2.26852	H	2.73679	2.27048	2.51253	H	3.09702	2.71681	1.66942
C	1.22097	2.95076	0.44425	C	0.90282	2.95069	0.62315	C	1.07167	2.94384	-0.13076
H	0.54803	2.77254	-0.39154	H	0.29659	2.78785	-0.26534	H	0.36287	2.57037	-0.86662
H	0.79577	2.49963	1.33827	H	0.46029	2.40225	1.45211	H	0.71892	2.67199	0.86224
H	1.27813	4.02453	0.60641	H	0.86645	4.01032	0.86503	H	1.08490	4.02932	-0.19741
C	3.15492	3.15909	-1.08034	C	2.90246	3.41633	-0.75332	C	2.90938	2.89575	-1.78597
H	4.17219	2.87023	-1.31479	H	3.95173	3.22130	-0.93955	H	3.92162	2.58922	-2.02142
H	2.52243	2.94243	-1.93868	H	2.34257	3.21868	-1.66493	H	2.23636	2.48990	-2.53820
H	3.13187	4.23278	-0.90153	H	2.78381	4.46861	-0.50057	H	2.85603	3.98233	-1.82994
C	-4.26033	0.58156	0.80195	C	-4.05191	0.45324	1.02522	C	-4.05614	0.90859	0.70480
C	-5.63128	0.01233	0.40991	C	-5.46428	-0.04947	0.69262	C	-5.48309	0.34418	0.64679
H	-6.38241	0.36350	1.11548	H	-6.14586	0.23313	1.49319	H	-6.14401	0.97313	1.24105
H	-5.92150	0.32320	-0.58651	H	-5.82860	0.37161	-0.23654	H	-5.85752	0.31210	-0.36905
H	-5.61085	-1.07482	0.44434	H	-5.46589	-1.13420	0.60916	H	-5.50632	-0.66198	1.05968
C	-4.30448	2.11646	0.78509	C	-4.06064	1.98138	1.18103	C	-4.03399	2.33893	0.14544
H	-4.54963	2.49668	-0.19940	H	-4.38175	2.47229	0.27039	H	-4.35259	2.36527	-0.88984
H	-5.05884	2.46365	1.48912	H	-4.74116	2.25916	1.98417	H	-4.70294	2.96753	0.73109
H	-3.34147	2.52307	1.08691	H	-3.06502	2.33796	1.43783	H	-3.02942	2.75204	0.21291
C	-3.97347	0.14445	2.23980	C	-3.66072	-0.14253	2.37891	C	-3.65515	0.98795	2.17942
H	-3.96331	-0.93903	2.33670	H	-3.66260	-1.23003	2.35550	H	-3.67366	0.00939	2.65457
H	-3.02554	0.53856	2.60009	H	-2.67962	0.19953	2.70158	H	-2.66522	1.42203	2.30475
H	-4.76189	0.52992	2.88191	H	-4.38763	0.17801	3.12146	H	-4.36728	1.62516	2.69828
**SW (C8$^\bullet$)**				**SW (C9$^\bullet$)**				**SW (C10$^\bullet$)**			
C	-2.51862	0.98544	0.13746	C	-2.64330	0.89672	-0.04596	C	2.67317	0.89687	-0.03865
C	-1.39871	0.16844	0.02661	C	-1.47310	0.14283	0.04711	C	1.49025	0.16583	-0.05401
C	-1.43511	-1.22060	0.08428	C	-1.42719	-1.19834	0.38315	C	1.42013	-1.20078	-0.29993
C	-2.65947	-1.84907	0.30591	C	-2.64329	-1.86953	0.67556	C	2.59640	-1.89050	-0.59098
C	-3.79032	-1.05595	0.43617	C	-3.83559	-1.11222	0.59584	C	3.78919	-1.18087	-0.59536
C	-3.73364	0.32646	0.34546	C	-3.84079	0.21532	0.24465	C	3.83813	0.17630	-0.31741
H	-0.43712	0.62949	-0.11451	H	-0.53360	0.62881	-0.14798	H	0.56305	0.67871	0.13195
H	-4.74886	-1.52652	0.61095	H	-4.77358	-1.60232	0.81771	H	4.71196	-1.69873	-0.82064
C	-0.16124	-2.02242	-0.13960	C	-0.10739	-1.94639	0.37087	C	0.09193	-1.93181	-0.18187
H	-0.25626	-2.94607	0.42861	H	-0.15636	-2.70517	1.15033	H	0.12444	-2.78316	-0.86151
C	1.05050	-1.30111	0.42446	C	1.06368	-1.05219	0.74026	C	-1.08205	-1.08386	-0.63568
C	1.20780	-1.21006	1.80984	C	1.14460	-0.52325	2.03235	C	-1.17866	-0.69527	-2.00086
C	1.99654	-0.68955	-0.38948	C	2.06989	-0.73794	-0.16554	C	-2.08583	-0.68891	0.22881
C	2.29344	-0.50665	2.30593	C	2.21498	0.30148	2.33997	C	-2.29654	0.07938	-2.38158
C	3.09078	0.03193	0.08443	C	3.15117	0.09473	0.11775	C	-3.19199	0.08649	-0.13428
H	1.87547	-0.77291	-1.45317	H	2.01084	-1.16161	-1.14963	H	-2.01406	-0.99731	1.25385
C	3.21475	0.10918	1.47205	C	3.19521	0.61643	1.41082	C	-3.26677	0.46086	-1.48579
H	2.43035	-0.43229	3.37668	H	2.29426	0.71187	3.33808	H	-2.38807	0.37895	-3.41661
C	-0.01127	-2.44794	-1.61848	C	0.06527	-2.70905	-0.96043	C	-0.07303	-2.52476	1.23468
H	1.01607	-2.76711	-1.78001	H	1.05511	-3.16068	-0.95917	H	-1.06650	-2.96337	1.29988
H	-0.63464	-3.32274	-1.79070	H	-0.64886	-3.53093	-0.96223	H	0.62833	-3.35118	1.33330
C	-0.41258	-1.38401	-2.66374	C	-0.15388	-1.88401	-2.24173	C	0.17106	-1.55581	2.40582
H	0.10269	-1.63467	-3.59472	H	0.57552	-2.18943	-2.99038	H	-0.54330	-1.77061	3.19921
H	-0.05768	-0.40250	-2.36178	H	0.02860	-0.82987	-2.04543	H	-0.01553	-0.53155	2.09228
C	-1.87800	-1.33367	-2.90427	C	-1.55673	-2.04398	-2.82322	C	1.58552	-1.64935	2.97375
H	-2.46406	-0.46376	-2.66844	H	-1.66594	-1.47049	-3.74033	H	1.71880	-0.95957	3.80351
H	-2.38795	-2.21278	-3.26220	H	-1.75601	-3.08876	-3.05320	H	1.78294	-2.65557	3.33860
C	-2.78352	-3.34426	0.41638	H	-2.31195	-1.70478	-2.11940	H	2.32548	-1.41234	2.21376
H	-2.43247	-3.83919	-0.48531	C	-2.71741	-3.22375	1.02367	C	2.61048	-3.36148	-0.91231
H	-2.19763	-3.72492	1.25125	H	-1.84806	-3.84958	1.09293	H	2.20645	-3.95553	-0.09724
H	-3.81824	-3.62965	0.57565	H	-3.67360	-3.67241	1.22889	H	2.01508	-3.57705	-1.79803
C	0.23981	-1.85458	2.76367	C	0.11491	-0.82050	3.08785	H	3.62442	-3.69795	-1.10243
H	-0.77071	-1.48655	2.60227	H	-0.83659	-0.35026	2.85089	C	-0.23214	-1.05211	-2.96850

H	0.22149	-2.93488	2.62961	H	-0.06980	-1.88905	3.17220	H	0.65488	-1.60485	-2.72338
H	0.52309	-1.64956	3.79097	H	0.44842	-0.45731	4.05473	H	-0.36821	-0.74204	-3.98989
O	4.26695	0.79979	2.02011	O	4.22766	1.44607	1.77104	O	-4.33201	1.21652	-1.90980
H	4.19871	0.76080	2.97524	H	4.10668	1.71307	2.68338	H	-4.23435	1.38956	-2.84741
O	-4.88563	1.06165	0.47254	O	-5.02680	0.90249	0.17808	O	5.04694	0.82610	-0.32668
H	-5.62313	0.46400	0.60388	H	-5.74339	0.30406	0.39476	H	5.73646	0.19538	-0.53883
C	-2.41951	2.50585	0.02874	C	-2.61928	2.37054	-0.43725	C	2.69246	2.39362	0.26608
C	-3.25721	3.00157	-1.15956	C	-3.42740	2.58650	-1.72631	C	3.54036	2.66286	1.51814
H	-4.30586	2.75896	-1.03783	H	-4.46624	2.30862	-1.59800	H	4.56707	2.34633	1.38012
H	-3.15827	4.08242	-1.24574	H	-3.38003	3.63716	-2.00775	H	3.53142	3.72931	1.73741
H	-2.89854	2.55204	-2.08361	H	-3.00451	1.99646	-2.53687	H	3.12340	2.13443	2.37324
C	-0.97769	2.96103	-0.20725	C	-1.19442	2.86159	-0.70246	C	1.28650	2.93038	0.54153
H	-0.32222	2.66846	0.61011	H	-0.56503	2.77136	0.18004	H	0.63012	2.80241	-0.31625
H	-0.57683	2.56140	-1.13671	H	-0.72762	2.31912	-1.52178	H	0.83395	2.44592	1.40412
H	-0.96451	4.04620	-0.27678	H	-1.23757	3.91253	-0.97828	H	1.35802	3.99437	0.75433
C	-2.90463	3.16678	1.32689	C	-3.19816	3.23067	0.69685	C	3.25689	3.17319	-0.93029
H	-3.93957	2.92408	1.53540	H	-4.22911	2.97384	0.90718	H	4.27515	2.87906	-1.15359
H	-2.29247	2.84016	2.16480	H	-2.61178	3.09933	1.60380	H	2.64219	3.00332	-1.81168
H	-2.81297	4.24809	1.23770	H	-3.15274	4.27998	0.40997	H	3.24509	4.23837	-0.70496
C	4.08749	0.70151	-0.86023	C	4.21726	0.41669	-0.92825	C	-4.24972	0.50200	0.88328
C	5.49474	0.12655	-0.64025	C	5.59607	-0.05656	-0.44448	C	-5.62273	-0.05976	0.48334
H	6.19161	0.60296	-1.32788	H	6.34154	0.16448	-1.20661	H	-6.36157	0.23392	1.22709
H	5.83952	0.29401	0.37274	H	5.88500	0.43638	0.47570	H	-5.93923	0.31071	-0.48388
H	5.49711	-0.94328	-0.83824	H	5.58621	-1.13150	-0.27728	H	-5.58749	-1.14651	0.44725
C	4.09645	2.21955	-0.62562	C	4.24697	1.92793	-1.20131	C	-4.31627	2.03469	0.97655
H	4.39411	2.46425	0.38681	H	4.48918	2.48933	-0.30704	H	-4.58571	2.48030	0.02704
H	4.79576	2.68798	-1.31636	H	4.99683	2.14335	-1.96081	H	-5.06145	2.31825	1.71792
H	3.10649	2.63259	-0.80912	H	3.27961	2.26091	-1.57187	H	-3.35359	2.43334	1.28986
C	3.72258	0.46881	-2.32763	C	3.93373	-0.28254	-2.25946	C	-3.93034	-0.02615	2.28366
H	3.72907	-0.58899	-2.58173	H	3.93206	-1.36520	-2.15291	H	-3.90531	-1.11345	2.30810
H	2.74642	0.88328	-2.57077	H	2.98230	0.03052	-2.68429	H	-2.98072	0.35558	2.65300
H	4.46137	0.96638	-2.95122	H	4.71830	-0.01729	-2.96410	H	-4.71134	0.30513	2.96370
**SW (C11·)**				**SW (C12·)**							
C	2.62439	0.95248	-0.08616	C	2.60198	0.92501	-0.09562				
C	1.46452	0.18915	-0.02338	C	1.43890	0.16599	-0.01931				
C	1.43175	-1.19196	-0.17890	C	1.39595	-1.21569	-0.17233				
C	2.62529	-1.86054	-0.45162	C	2.58030	-1.89475	-0.45517				
C	3.79508	-1.11727	-0.53019	C	3.75302	-1.15870	-0.54980				
C	3.80736	0.25683	-0.34332	C	3.77520	0.21557	-0.36781				
H	0.53255	0.70280	0.15250	H	0.50573	0.66886	0.16367				
H	4.72838	-1.62129	-0.74336	H	4.68103	-1.66840	-0.77226				
C	0.12665	-1.95055	0.00193	C	0.08616	-1.96312	0.02124				
H	0.18921	-2.85314	-0.60479	H	0.13718	-2.86842	-0.58250				
C	-1.06299	-1.16811	-0.52745	C	-1.10063	-1.17369	-0.50375				
C	-1.17238	-0.92894	-1.90060	C	-1.21699	-0.93762	-1.87779				
C	-2.05575	-0.67178	0.30893	C	-2.08630	-0.66735	0.33420				
C	-2.25244	-0.19165	-2.35782	C	-2.29475	-0.19562	-2.33395				
C	-3.14872	0.07605	-0.12576	C	-3.17488	0.08414	-0.10187				
H	-1.97592	-0.87491	1.35968	H	-2.01918	-0.85733	1.39146				
C	-3.21901	0.31050	-1.49900	C	-3.25544	0.31576	-1.47345				
H	-2.35099	-0.00195	-3.41848	H	-2.39623	-0.01019	-3.39509				
C	-0.02881	-2.43212	1.46108	C	-0.06557	-2.43784	1.48291				
H	-1.01941	-2.87085	1.56178	H	-1.06001	-2.86592	1.59079				
H	0.67966	-3.24334	1.62095	H	0.63594	-3.25533	1.64164				
C	0.20910	-1.37642	2.55600	C	0.18832	-1.38127	2.57297				
H	-0.49994	-1.54002	3.36609	H	-0.51377	-1.54019	3.38988				
H	0.01069	-0.38025	2.16903	H	-0.01109	-0.38529	2.18618				
C	1.62654	-1.41398	3.12265	C	1.61106	-1.42389	3.12585				
H	1.75734	-0.66250	3.89751	H	1.75298	-0.67066	3.89707				

H	1.83472	-2.38808	3.56119	H	1.81851	-2.39762	3.56561
H	2.36159	-1.22883	2.34359	H	2.33969	-1.24501	2.33939
C	2.67701	-3.34705	-0.68390	C	2.61927	-3.38242	-0.68171
H	2.28075	-3.90078	0.16285	H	2.22661	-3.92974	0.17090
H	2.09240	-3.62373	-1.55969	H	2.02511	-3.65879	-1.55117
H	3.69965	-3.67044	-0.84843	H	3.63813	-3.71379	-0.85382
C	-0.16762	-1.45937	-2.88614	C	-0.22036	-1.47749	-2.86661
H	0.82683	-1.07060	-2.68209	H	0.77648	-1.09024	-2.67157
H	-0.10989	-2.54555	-2.83628	H	-0.16530	-2.56342	-2.80972
H	-0.44690	-1.18561	-3.89845	H	-0.50565	-1.20966	-3.87882
O	-4.26310	1.04192	-2.00888	O	-4.30023	1.04965	-1.97792
H	-4.15790	1.11046	-2.95892	H	-4.20178	1.11573	-2.92890
O	4.99214	0.94464	-0.42159	O	4.96485	0.89243	-0.46701
H	5.70000	0.32517	-0.60505	H	5.66326	0.26575	-0.66155
C	2.58235	2.47273	0.12774	C	2.59238	2.43829	0.11271
C	3.43516	2.84802	1.35175	C	3.47798	2.80713	1.31241
H	4.47045	2.56153	1.21149	H	4.50758	2.51006	1.15412
H	3.38509	3.92317	1.51032	H	3.44661	3.88436	1.46687
H	3.05029	2.35004	2.23910	H	3.10816	2.32151	2.21326
C	1.18674	2.94504	0.37822	C	1.18354	2.95750	0.40701
H	0.52335	3.15581	-0.44400	H	0.49965	2.75985	-0.41545
H	0.79843	3.00415	1.38125	H	0.77500	2.51813	1.31459
C	3.10340	3.19758	-1.12587	H	1.23458	4.03431	0.55031
H	4.12917	2.92295	-1.34033	C	3.09232	3.15399	-1.15051
H	2.48528	2.94625	-1.98488	H	4.10775	2.86784	-1.39620
H	3.05194	4.27260	-0.96729	H	2.44782	2.91781	-1.99463
C	-4.19824	0.61004	0.84776	H	3.06450	4.23050	-0.99007
C	-5.58327	0.04411	0.50129	C	-4.21431	0.62070	0.89385
H	-6.31476	0.41896	1.21545	C	-5.60402	0.05011	0.56202
H	-5.89466	0.33271	-0.49520	H	-6.32821	0.42281	1.28347
H	-5.57123	-1.04210	0.56256	H	-5.91964	0.33927	-0.43315
C	-4.23066	2.14480	0.79649	H	-5.58498	-1.03580	0.62194
H	-4.49313	2.50479	-0.19092	C	-4.24513	2.15848	0.83739
H	-4.96682	2.51489	1.50828	H	-4.51784	2.50868	-0.15085
H	-3.25794	2.54927	1.06899	H	-4.97097	2.53182	1.55680
C	-3.88901	0.20758	2.29133	H	-3.26725	2.55994	1.09433
H	-3.88445	-0.87317	2.41584	C	-3.88117	0.22417	2.29558
H	-2.93210	0.60340	2.62517	H	-4.24073	-0.70558	2.70415
H	-4.66280	0.61473	2.93779	H	-3.22753	0.83323	2.89791
